# Supplementary material for: Accessing five oxidation states of uranium in a retained ligand framework
Source: Nat Commun. 2023 Aug 3;14:4657. doi: 10.1038/s41467-023-40403-w (PMC10400547; doi:10.1038/s41467-023-40403-w)
Supplement: Supplementary file 1 — Supplementary Information [file 41467_2023_40403_MOESM1_ESM.pdf]

Supplementary Information for

Accessing Five Oxidation States of Uranium in a Retained Ligand Framework

Chong Deng, Jiefeng Liang, Rong Sun, Yi Wang, Peng-Xiang Fu, Bing-Wu Wang, Song Gao, Wenliang Huang*

Correspondence to: wlhuang@pku.edu.cn

Table of Contents

1. Experimental Procedures 2

1.1 General Considerations 2

1.2 Synthetic Details 3

1.3 Trapping Experiment 11

2. X-ray Crystallography 13

3. Infrared (IR) Spectra 29

4. Electrochemical Measurement 41

5. Nuclear Magnetic Resonance (NMR) Spectra 55

6. Ultraviolet–Visible–Near-Infrared (UV-Vis-NIR) Spectra 79

7. X-ray Photoelectron Spectroscopy (XPS) 94

8. SQUID Measurement 102

9. Electronic Paramagnetic Resonance (EPR) Spectra 109

10. Density Functional Theory (DFT) Calculations 112

11. References 162

1. Experimental Procedures

1.1. General Considerations

All experiments were performed under a dry argon atmosphere using standard Schlenk techniques or in a nitrogen filled Vigor glove box unless otherwise specified. Celite (purchased from Sigma-Aldrich), neutral alumina and 4 Å molecular sieves (purchased from Sinopharm) were dried under dynamic vacuum at 250 °C for at least 48 h prior to use. All solvents were purchased from Acros, Alfa Aesar, Honeywell or Fisher. Solvents including *n*-pentane, hexanes, diethyl ether (Et_2O), toluene, and methylene chloride (CH_2Cl_2) were collected from a Vigor YJC-5 Solvent Purification System under argon, transferred to the glove box without exposure to air, and stored over activated molecular sieves. Tetrahydrofuran (THF) was refluxed over sodium hydride for 72 h and distilled from sodium-benzophenone ketyl before use. Deuterated solvents including benzene-d_6 (C_6D_6), tetrahydrofuran-d_8 (C_4D_8O), pyridine-d_5 (C_5D_5N), and dichloromethane-d_2 (CD_2Cl_2) were obtained from Cambridge Isotope Laboratories, degassed three times using freeze-pump-thaw method and stored over activated molecular sieves for one week prior to use.

1,3,5-Tris(2-bromophenyl)benzene[1], benzyl potassium (KBn)[2], $UI_3(solv)_x$ (solv = THF, $x = 4$; or solv = 1,4-dioxane, $x = 1.5$)[3-5], and potassium graphite (KC_8)[6] were prepared following published procedures. 4,7,13,16,21,24-Hexaoxa-1,10-diazabicyclo[8.8.8]hexacosane (2.2.2-cryptand, crypt) was purchased from Amethyst, dissolved in THF, passed through neutral alumina, and dried under reduced pressure before use. Cobaltocene (Cp_2Co, Cp = cyclopentadienyl) was purchased from Macklin, dissolved in toluene, passed through neutral alumina, and recrystallized at –35 °C before use. [TBA][PF_6] (TBA = $^nBu_4N$) was purchased from Sigma-Aldrich and recrystallized three times from acetone before use. All other reagents were purchased from commercial vendors and used as received.

^1H, ^{13}C, and ^{19}F NMR spectra were recorded on Bruker Avance 400 MHz or 600 MHz spectrometers at room temperature. FWHM (full width at half maximum) values are given for broad peaks. Two-dimensional (2D) NMR (^1H–^{13}C correlation) spectra were recorded on a Bruker Avance 500 MHz spectrometer. Chemical shifts are referenced internally to the residual proteo-solvent signal. CHN analyses were performed on a Vario EL elemental analyzer at the Analytical Center of Peking University. The single crystal X-ray diffraction data were collected at 180 K using a Rigaku Oxford diffractometer equipped with a CCD collector using Mo Kα radiation, namely XtaLAB PRO 007HF(Mo). Electrochemical measurements were performed on a CHI730E instrument using a three-electrode system containing a glassy carbon working electrode, a platinum wire counter electrode, and a platinum wire reference electrode. The internal standard Fc^* (Cp^*_2Fe, Cp^* = pentamethylcyclopentadienyl) was added for each solution, while the internal resistance was measured and automatically compensated for each cyclic voltammetry experiment. The UV-Vis-NIR absorption spectra were recorded with a Shimadzu UV3600Plus instrument. The temperature dependent magnetic susceptibilities were measured under a DC field of 1 kOe in the temperature range of 2–298 K (or 2–300 K) with a Quantum Design MPMS-XL5

superconducting quantum interference device (SQUID) magnetometer at Peking University or a Quantum Design MPMS3 magnetometer at Beijing Normal University. The samples (15–20 mg each) were packed in capsules with N-grease and parafilm covered to protect them from air and moisture. The background of sample holders (capsule, parafilm and N-grease) and Pascal correction were considered when the diamagnetic correction was carried on the data. Continuous-wave electron paramagnetic resonance (CW-EPR) experiments were performed on a Bruker Elexsys E580 spectrometer, operating at the X-band (ω = 9.35 GHz) with a low-temperature environment achieved by using an Oxford Instruments ESR900 and CF935 liquid helium cryostat. The EPR spectrum simulation was performed with the *EasySpin* toolbox (http://www.easyspin.org/) based on MATLAB[7]. XPS measurements were carried out at 298 K on an AXIS Supra X-ray Photoelectron Spectrometer at the Analytical Center of Peking University. Fourier transform infrared (FTIR) spectra in the range of 4000–400 cm^{-1} were recorded on a Bruker Tensor 27 spectrometer using a KBr pellet (for solid samples) or a KBr cell (for solution samples) at the Analytical Center of Peking University.

Caution! *Depleted uranium (primary isotope ^{238}U) is a weak α-emitter (4.197 MeV) with a half-life of 4.47 × 10^9 years; manipulations and reactions should be carried out in monitored fume hoods or in an inert atmosphere glovebox in a radiation laboratory equipped with α- and β-counting equipment.*

1.2. Synthetic Details

1.2.1. Synthesis of 1,3,5-tris[2-(1-adamantylamino)phenyl]benzene, $H_3(^{Ad}TPBN_3)$

A 75 mL thick-walled Schlenk tube was charged with 1,3,5-tris(2-bromophenyl)benzene (2.000 g, 3.68 mmol, 1.0 equiv.), 1-adamantylamine (1.840 g, 12.2 mmol, 3.3 equiv.), $Pd_2(dba)_3$ (0.253 g, 0.276 mmol, 0.075 equiv., dba = dibenzylideneacetone), XPhos (0.395 g, 0.829 mmol, 0.225 equiv., 2-dicyclohexylphosphino-2′,4′,6′-triisopropylbiphenyl, CAS No. 564483-18-7), sodium *tert*-butoxide (1.700 g, 17.7 mmol, 4.8 equiv.), and toluene (25 mL). The resulting suspension was vigorously stirred at 80 °C for three days. After cooled to room temperature, the reaction mixture was filtered through a Celite-padded medium-porosity fritted filter, washed with toluene (ca. 3 mL × 3) until the elute was nearly colorless. The filtrate was collected, and the volatiles were removed under reduced pressure to give a brown oil. The crude product was purified by chromatography (eluent: 3% ethyl acetate in *n*-hexane) and then washed with cold *n*-pentane to yield $H_3(^{Ad}TPBN_3)$ as an off-white solid (1.940 g, 70%). Single crystals of $H_3(^{Ad}TPBN_3)$ suitable for X-ray crystallography were grown from a Et_2O solution at 4 °C. ^1H NMR (400 MHz, $CDCl_3$) δ, ppm: 7.36 (s, 3H, C*H* of the anchor ring), 7.19 (td, *J* = 7.6, 1.4 Hz, 3H, C*H* of side rings), 7.12 (dd, *J* = 7.5, 1.2 Hz, 3H, C*H* of side rings), 7.07 (d, *J* = 8.1 Hz, 3H, C*H* of side rings), 6.79 (t, *J* = 7.4 Hz, 3H, C*H* of side rings), 3.88 (s, 3H, N*H*), 2.07 (br s, FWHM = 10.4 Hz, 9H, Ad), 1.84 (m, 18 H, Ad), 1.63 (m, 18 H, Ad). ^1H NMR (400 MHz, C_6D_6) δ, ppm: 7.66 (s, 3H, C*H* of the anchor ring), 7.29 (dd, *J* = 7.5, 1.4 Hz, 3H, C*H* of side rings), 7.14–7.21 (m, 6H, C*H* of side rings, overlapped with C_6D_5H), 6.82 (td, *J* = 7.2, 1.4 Hz, 3H, C*H* of side rings), 4.12 (s, 3H, N*H*), 1.88 (br s,

FWHM = 12.6 Hz, 9H, Ad), 1.84 (m, 18 H, Ad), 1.47 (m, 18 H, Ad). ^{1}H NMR (400 MHz, C_4D_8O) δ, ppm: 7.33 (s, 3H, C*H* of the anchor ring), 7.05–7.12 (m, 9H, C*H* of side rings), 6.70 (t, *J* = 7.1 Hz, C*H* of side rings), 6.82 (td, *J* = 7.2, 1.4 Hz, 3H, C*H* of side rings), 3.86 (s, 3H, N*H*), 2.04 (br s, FWHM = 11.2 Hz, 9H, Ad), 1.88 (s, 18 H, Ad), 1.67 (m, 18 H, Ad). ^{13}C NMR (100 MHz, $CDCl_3$) δ, ppm: 143.69 (*C*N of side rings), 141.44 (C_{ipso} of the anchor ring), 130.43, 129.97, 129.61, 128.17, 117.51, 116.36 (*C*H of the anchor ring, C_{ipso} and *C*H of side rings), 52.28 (*C*N of Ad), 43.55 (CH_2 of Ad), 36.59 (CH_2 of Ad), 29.84 (*C*H of Ad). ^{13}C NMR (100 MHz, C_6D_6) δ, ppm: 144.16 (*C*N of side rings), 142.41 (C_{ipso} of the anchor ring), 130.87, 130.14, 130.09, 128.67, 118.03, 116.69 (*C*H of the anchor ring, C_{ipso} and *C*H of side rings), 52.25 (*C*N of Ad), 43.71 (CH_2 of Ad), 36.66 (CH_2 of Ad), 30.12 (*C*H of Ad). ^{13}C NMR (100 MHz, C_4D_8O) δ, ppm: 144.68 (*C*N of side rings), 142.82 (C_{ipso} of the anchor ring), 131.05, 130.93, 130.47, 128.93, 118.30, 117.27 (*C*H of the anchor ring, C_{ipso} and *C*H of side rings), 52.91 (*C*N of Ad), 44.50 (CH_2 of Ad), 37.51 (CH_2 of Ad), 31.12 (*C*H of Ad). Anal. (%): Calcd. for $C_{55}H_{65.5}N_3O_{0.25}$, M_w = 772.65, as in the formula of 1,3,5-[2-(1-AdNH)C_6H_4]$_3$$C_6H_3$·0.25$C_4H_{10}O$ (Et_2O): C, 85.50; H, 8.55; N, 5.44. Found: C, 85.47; H, 8.49; N, 5.46. FTIR (KBr) $\tilde{\nu}$, cm^{-1}: 481 (w), 743 (m), 1089 (m), 1127 (w), 1184 (w), 1249 (w), 1284 (w), 1309 (w), 1355 (w), 1409 (w), 1450 (m), 1507 (m), 1578 (m), 1603 (w), 2848 (m), 2904 (s), 3422 (m).

1.2.2. Synthesis of the tripotassium salt K_3(^{Ad}TPBN$_3$)

To a pre-cooled (–78 °C) suspension of H_3(^{Ad}TPBN$_3$) (1.000 g, 1.33 mmol, 1.0 equiv.) in Et_2O (40 mL), solid KBn (0.540 g, 4.15 mmol, 3.1 equiv.) was added in small portions when keeping cold (–78 °C). The mixture was warmed to room temperature and stirred for three hours. After removing the volatiles under reduced pressure, the remaining solid was transferred to a medium-porosity fritted filter with the help of hexanes (10 mL) and then washed with addition hexanes (10 mL × 2) and dried under reduced pressure. K_3(^{Ad}TPBN$_3$) was obtained as a yellow solid (1.090 g, 95%). K_3(^{Ad}TPBN$_3$) is almost insoluble in aromatic solvents (e.g., benzene, toluene) and moderately soluble in THF. ^{1}H NMR (400 MHz, C_4D_8O) δ, ppm: 6.81 (s, 3H, C*H* of the anchor ring), 6.50–6.57 (m, 6H, C*H* of side rings), 6.29 (d, *J* = 8.3 Hz, 3H, C*H* of side rings), 5.49 (d, *J* = 6.7 Hz, 3H, C*H* of side rings), 2.00 (br s, FWHM = 12.2 Hz, 9H, Ad), 1.88 (s, 18 H, Ad), 1.65 (s, 18 H, Ad). ^{13}C NMR (100 MHz, C_4D_8O) δ, ppm: 160.32 (*C*N of side rings), 149.55 (C_{ipso} of the anchor ring), 131.88, 130.36, 129.85, 128.32, 113.11, 100.97 (*C*H of the anchor ring, C_{ipso} and *C*H of side rings), 53.88 (*C*N of Ad), 44.84 (CH_2 of Ad), 38.89 (CH_2 of Ad), 31.96 (*C*H of Ad). Anal. (%): Calcd. for $C_{57}H_{67}K_3N_3$, M_w = 911.48, as in the formula of 1,3,5-[2-(1-AdN(K))C_6H_4]$_3$$C_6H_3$·0.5$C_6H_{14}$ (*n*-hexane): C, 75.11; H, 7.41; N, 4.61. Found: C, 74.75; H, 7.64; N, 4.38. FTIR (KBr) $\tilde{\nu}$, cm^{-1}: 613 (w), 720 (w), 743 (w), 847 (w), 900 (w), 1055 (m), 1088 (w), 1142 (w), 1181 (w), 1301 (m), 1334 (w), 1355 (w), 1366 (w), 1406 (w), 1439 (m), 1468 (m), 1514 (w), 1579 (m), 2848 (m), 2903 (s).

1.2.3. Synthesis of (^{Ad}TPBN$_3$)U (1)

A 50 mL round-bottom flask was charged with $K_3(^{Ad}TPBN_3)$ (0.384 g, 0.44 mmol, 1.0 equiv.) and THF (20 mL). A second 50 mL round-bottom flask was charged with $UI_3(THF)_4$ (0.401 g, 0.44 mmol, 1 equiv.) and pre-cooled (–78 °C) THF (10 mL). Both mixtures were cooled at –78 °C for at least 15 minutes. The cold suspension of $K_3(^{Ad}TPBN_3)$ in THF was added to the cold THF solution of $UI_3(THF)_4$ dropwise with stirring. After 15 minutes kept at –78 °C, the reaction mixture was warmed to room temperature and stirred for an hour. The volatiles were removed under reduced pressure, the residues were extracted into toluene (ca. 20 mL) and filtered through a Celite-padded coarse-porosity fritted filter. The filtrate was collected and the volatiles were removed under reduced pressure. The three-step procedure (extraction, filtration, and drying) was repeated with toluene (ca. 20 mL) to remove residual KI. Then the remaining solid was extracted completely into Et_2O (ca. 30 mL) and filtered through a Celite pad. The filtrate was concentrated to ca. 10 mL, and stored at –35 °C for two days. The product precipitated out from solution, and was washed with cold *n*-pentane and dried under reduced pressure to yield **3** as a black crystalline solid (0.284 g, 65%). Identical yield and purity of the product could be afforded when using UI_3(1,4-dioxane)$_{1.5}$ as the uranium starting material instead of $UI_3(THF)_4$. Single crystals of **3** suitable for X-ray crystallography were grown from a Et_2O solution at –35 °C. ^{1}H NMR (400 MHz, C_6D_6) δ, ppm: 12.39 (s, 3H, C*H* of side rings), 11.21 (s, 3H, C*H* of side rings), 6.90 (br s, FWHM ≈ 300 Hz, 9H, Ad, overlapped with C_6D_5H), 4.29 (br s, FWHM ≈ 160 Hz, 3H, C*H* of the anchor ring, overlapped with others), 4.10 (s, 3H, C*H* of side rings), 4.03 (s, 3H, C*H* of side rings), 0.64 (s, 9H, Ad), –0.02 (s, 9H, Ad), –0.17 (s, 9H, Ad), –12.40 (br s, FWHM = 320 Hz, 9H, Ad). Anal. (%): Calcd. for $C_{55.25}H_{63}N_3U$, M_w = 1007.16, as in the formula of (1,3,5-[2-(1-AdN)C_6H_4]$_3C_6H_3$)U·0.25C_5H_{12} (*n*-pentane): C, 65.89; H, 6.31; N, 4.17. Found: C, 66.16; H, 6.41; N, 4.21. FTIR (KBr) $\tilde{\nu}$, cm^{-1}: 743 (m), 891 (m), 1035 (w), 1068 (w), 1095 (w), 1135 (w), 1268 (w), 1286 (w), 1308 (w), 1356 (w), 1384 (m), 1408 (w), 1451 (m), 1511 (m), 1580 (m), 1602 (w), 2849 (m), 2904 (s).

1.2.4. Synthesis of [K(crypt)][(^{Ad}TPBN$_3$)U] (2)

1 (0.100 g, 0.101 mmol, 1.0 equiv.) and 2.2.2-cryptand (0.038 g, 0.10 mmol, 1.0 equiv.) were weighed in a vial. THF (5 mL) was added to dissolve the mixture and the solution was cooled down to –78 °C for 15 minutes. KC_8 (0.016 g, 0.12 mmol, 1.2 equiv.) was added in small portions when keeping cold. The reaction mixture was warmed to room temperature and rigorously stirred for an hour. The dark-brown/black suspension was then filtered through Celite and washed with THF (ca. 1 mL × 3). The filtrate was concentrated to 2 mL, layered with hexanes (2 mL), and stored at –35 °C overnight. After removing the supernatant by filtration and washing with cold hexanes, the product was collected and dried on a medium frit to yield **2** as a brownish red solid (0.128 g, 90%). Single crystals of **2** suitable for X-ray crystallography were grown from a THF/Et_2O/hexanes solution at room temperature. ^{1}H NMR (400 MHz, C_6D_6) δ, ppm: 16.78 (d, *J* = 8.4 Hz, 3H, C*H* of side rings), 8.19 (t, *J* = 7.0 Hz, 3H, C*H* of side rings), 7.59 (t, *J* = 7.6 Hz, 3H, C*H* of side rings), 4.56 (br s, FWHM = 110 Hz, 9H, Ad), 2.55 (s, 12H, OC*H*$_2$C*H*$_2$O of [K(crypt)]$^+$), 2.41 (t, *J* = 4.0 Hz, 12H, NCH$_2$C*H*$_2$O of

[K(crypt)]$^+$), 1.46 (t, J = 4.0 Hz, 12H, NCH_2CH$_2$O of [K(crypt)]$^+$), 0.91 (br s, FWHM = 11.7 Hz, 9H, Ad), 0.61 (m, 9H, Ad), 0.21–0.25 (m, 12H, CH of side rings (3H) & Ad (9H)), –9.63 (br s, FWHM = 180 Hz, 9H, Ad), –71.98 (s, 3H, CH of the anchor ring). ^{1}H NMR (400 MHz, C_4D_8O) δ, ppm: 16.37 (d, J = 8.4 Hz, 3H, CH of side rings), 7.75 (t, J = 7.2 Hz, 3H, CH of side rings), 7.45 (t, J = 7.8 Hz, 3H, CH of side rings), 3.91 (br s, FWHM = 65 Hz, 9H, Ad), 3.32 (s, 12H, OCH_2CH_2O of [K(crypt)]$^+$), 3.24 (t, J = 4.4 Hz, 12H, NCH$_2$CH_2O of [K(crypt)]$^+$), 2.25 (t, J = 4.5 Hz, 12H, NCH_2CH$_2$O of [K(crypt)]$^+$), 0.66 (br s, FWHM = 11.6 Hz, 9H, Ad), 0.54 (m, 9H, Ad), 0.09 (m, 9H, Ad), –0.17 (d, J = 6.6 Hz, 3H, CH of side rings), –10.64 (br s, FWHM = 160 Hz, 9H, Ad), –73.35 (s, 3H, CH of the anchor ring). ^{1}H NMR (400 MHz, C_5D_5N) δ, ppm: 16.90 (d, J = 8.6 Hz, 3H, CH of side rings), 8.19 (t, J = 7.0 Hz, 3H, CH of side rings), 7.80 (t, J = 7.8 Hz, 3H, CH of side rings), 4.32 (br s, FWHM = 45 Hz, 9H, Ad), 3.35 (s, 12H, OCH_2CH_2O of [K(crypt)]$^+$), 3.28 (t, J = 4.4 Hz, 12H, NCH$_2$CH_2O of [K(crypt)]$^+$), 2.28 (t, J = 4.5 Hz, 12H, NCH_2CH$_2$O of [K(crypt)]$^+$), 0.73 (br s, FWHM = 13.4 Hz, 9H, Ad), 0.55–0.50 (m, 12 H, Ad (9H) & CH of side rings (3H)), 0.07 (m, 9H, Ad), –10.30 (br s, FWHM = 300 Hz, 9H, Ad), –72.30 (s, 3H, CH of the anchor ring). Anal. (%): Calcd. for $C_{74}H_{100}KN_5O_{6.5}U$, M_w = 1440.77, as in the formula of [K($C_{18}H_{36}N_2O_6$)][(1,3,5-[2-(1-AdN)C_6H_4]$_3C_6H_3$)U]·0.5C_4H_8O (THF): C, 61.69; H, 7.00; N, 4.86. Found: C, 61.90; H, 7.35; N, 4.80. FTIR (KBr) $\tilde{\nu}$, cm^{-1}: 743 (m), 845 (w), 902 (m), 948 (m), 1078 (m), 1104 (s), 1133 (m), 1260 (m), 1288 (m), 1355 (m), 1384 (m), 1449 (m), 1511 (w), 1580 (w), 2849 (m), 2902 (s).

1.2.5. Synthesis of (^{Ad}TPBN$_3$)UO (3)

Route A: 2e-Oxidation of (^{Ad}TPBN$_3$)U (**1**)

Preparation scale using pyridine-*N*-oxide as the oxidant: To a stirring solution of **1** (100 mg, 0.101 mmol, 1.0 equiv.) in toluene (5 mL), a solution of pyridine-*N*-oxide (9.6 mg, 0.101 mmol, 1.0 equiv.) in toluene (2 mL) was added at room temperature. After 10 minutes, the volatiles were removed under reduced pressure. The remaining solid was washed with hexanes (0.5 mL) and *n*-pentane (3 mL), and dried under reduced pressure to yield **3** as a black solid in an almost quantitative yield (101 mg, 99%). Single crystals of **3** suitable for X-ray crystallography were grown from a toluene/*n*-pentane solution at –35 °C (co-crystallized with one equivalent of toluene, Supplementary Fig. 5) or from a benzene/*n*-pentane solution at room temperature (Supplementary Fig. 6). ^{1}H NMR (400 MHz, C_6D_6) δ, ppm: 13.69 (s, 3H, CH of the anchor ring), 10.32 (d, J = 7.4 Hz, 3H, CH of side rings), 5.12 (t, J =7.3 Hz, 3H, CH of side rings), 4.09 (t, J = 7.2 Hz, 3H, CH of side rings), 0.55 (m, 9H, Ad), 0.31 (br s, FWHM = 12.7 Hz, 9H, Ad), 0.09 (m, 9H, Ad), –1.40 (d, J = 8.1 Hz, CH of side rings), –6.31 (br s, FWHM = 140 Hz, 9H, Ad), –7.25 (br s, FWHM = 31.1 Hz, 9H, Ad). Anal. (%): Calcd. for $C_{61}H_{68}N_3OU$, M_w = 1097.26, as in the formula of (1,3,5-[2-(1-AdN)C_6H_4]$_3C_6H_3$)UO·C_7H_8 (toluene): C, 66.77; H, 6.25; N, 3.83. Found: C, 66.88; H, 6.64; N, 3.85. FTIR (KBr) $\tilde{\nu}$, cm^{-1}: 612 (w), 642 (w), 716 (w), 728 (w), 743 (m), 759 (w), 783 (m), 844 (m), 889 (w), 937 (w), 1045 (w), 1075 (m), 1100 (w), 1119 (w), 1227 (s), 1279 (w), 1302 (w), 1354 (w), 1384 (m), 1445 (m), 1466 (w), 1591 (m), 2847 (m), 2903 (s).

NMR scale using N_2O as the oxidant: **1** (5.0 mg, 5.1 μmol, 1.0 equiv.) was dissolved in C_6D_6 (0.5 mL), and transferred to a J-Young tube. Upon freezing the solution, the headspace gas was removed under reduced pressure using the standard Schlenk technique. The tube was then warmed to about 10 °C, and filled with N_2O (*ca.* 15 equiv.). ^{1}H NMR spectroscopy showed the full consumption of **1**, and the formation of **3** as the major uranium product along with a small amount of the pro-ligand H_3(^{Ad}TPBN$_3$).

Route B: 1e-Oxidation of (^{Ad}TPBN$_3$)UI (**7**)

A 20 mL vial was charged with **7** (110 mg, 0.100 mmol, 1.0 equiv.) and THF (5 mL). A second 20 mL vial was charged with $AgNO_2$ (23 mg, 0.150 mmol, 1.5 equiv.) and THF (3 mL). Both mixtures were cooled at –35 °C for 15 minutes. In dark, the suspension of $AgNO_2$ in THF was added to the solution of **7** with stirring. The reaction mixture was warmed to room temperature and stirred for 2.5 hours in dark. After filtration through Celite, the volatiles were removed under reduced pressure. The remaining solid was extracted into toluene and filtered through Celite to get rid of residual salts. The volatiles were removed under reduced pressure again. After triturated with *n*-pentane (2 mL), the product was dried under reduced pressure to yield **3** as a black solid (94 mg, 95%).

Route C: 1e-Oxidation of [K(crypt)][(^{Ad}TPBN$_3$)UO] (**4**) (NMR scale)

To a saturated solution of **4** (5.0 mg, 3.5 μmol, 1.0 equiv.) in pyridine-d_5 (0.5 mL), solid AgOTf (0.9 mg, 3.5 μmol, 1 equiv.; OTf = CF_3SO_3) was added at room temperature. The solution color turned from brownish yellow to black immediately. ^{1}H NMR (400 MHz, C_5D_5N) spectroscopy revealed the quantitative formation of **3** and free 2.2.2-cryptand in a 1:1 molar ratio (Supplementary Fig. 53). ^{1}H NMR assignment for *in situ* formed **3** (δ, ppm): 13.77 (s, 3H, C*H* of the anchor ring), 10.58 (d, *J* = 7.3 Hz, 3H, C*H* of side rings), 5.29 (t, *J* =7.6 Hz, 3H, C*H* of side rings), 4.19 (t, *J* = 7.2 Hz, 3H, C*H* of side rings), 0.44 (m, 9H, Ad), 0.23 (br s, FWHM = 12.6 Hz, 9H, Ad), –0.01 (m, 9H, Ad), –1.41 (d, *J* = 8.3 Hz, C*H* of side rings), –6.42 (br s, FWHM = 180 Hz, 9H, Ad), –7.38 (br s, FWHM = 31.0 Hz, 9H, Ad). The resulting suspension was transferred to a vial. The volatiles were removed under reduced pressure, and the remaining solid was extracted into toluene. After filtered through Celite, the volatiles were removed under reduced pressure again. The remaining solid was washed with hexanes and dried under reduced pressure to yield **3** as a black solid (3.4 mg, 96%).

Route D: 1e-Reduction of [(^{Ad}TPBN$_3$)UO][SbF$_6$] (**5**) (NMR scale)

In a J-Young tube, Cp_2Co (1.0 mg, 5.3 μmol, 1.2 equiv.) was added to a suspension of **5** (5.4 mg, 4.4 μmol, 1.0 equiv.) in C_6D_6 (0.5 mL). Within 10 minutes at room temperature, ^{1}H NMR spectroscopy revealed the full consumption of **5**, and the formation of **3** as the major uranium product along with a small amount of the pro-ligand H_3(^{Ad}TPBN$_3$), and some excess of Cp_2Co. The reaction mixture was then filtered to remove insoluble by-products and transferred to a vial. After removing the volatiles under reduced pressure, the remaining solid was washed with cold hexanes and dried under reduced pressure to yield **3** as a black solid (2.7 mg, 61%).

1.2.6. Synthesis of [K(crypt)][(^{Ad}TPBN$_3$)UO] (4)

Route A: 1e-Reduction of (^{Ad}TPBN$_3$)UO (**3**)

To a pre-cooled (–78 °C) solution of **3** (0.043 g, 0.043 mmol, 1.0 equiv.) and 2.2.2-cryptand (0.016 g, 0.043 mmol, 1.0 equiv.) in THF (3 mL), KC_8 (0.007 g, 0.052 mmol, 1.2 equiv.) was added in small portions with stirring. The mixture was warmed to room temperature and stirred for 30 minutes. The suspension was filtered through a Celite pad, which was washed with THF until the elute was nearly colorless (or pale greenish yellow). The filtrate was concentrated to 2 mL, layered with hexanes (4 mL), and stored at –35 °C overnight. The product precipitated out from solution, and was washed with cold Et_2O and dried under reduced pressure to yield **4** as a greenish yellow solid (44 mg, 72%). ^{1}H NMR (400 MHz, C_5D_5N) δ, ppm: 62.12 (br s, FWHM = 320 Hz, 3H, C*H* of the anchor ring), 28.77 (s, 3H, C*H* of side rings), 3.82 (s, 12H, OC*H*$_2$C*H*$_2$O of [K(crypt)]$^+$), 3.78 (t, *J* = 4.7 Hz, 12H, NCH$_2$C*H*$_2$O of [K(crypt)]$^+$), 2.78 (m, 15H, NC*H*$_2$CH$_2$O of [K(crypt)]$^+$ (12H) & C*H* of side rings (3H)), –1.92 (br s, FWHM = 340 Hz, 9H, Ad), –5.55 (br s, FWHM = 39.7 Hz, 9H, Ad), –8.58 (s, 3H, C*H* of side rings), –9.37 (br s, FWHM = 140 Hz, 9H, Ad), –35.96 (s, 3H, C*H* of side rings), –46.34 (br s, FWHM = 680 Hz, 9H, Ad). One peak (9H) of Ad groups was not found in the range of –500 to +500 ppm, probably due to signal broadening. Notably, splitting of C*H* peaks of side rings could not be observed under this NMR experiment condition. The spectrum recorded in THF-*d*$_8$ (C_4D_8O) shows nearly identical chemical shifts for a set of peaks corresponding to the anion [(^{Ad}TPBN$_3$)UO]$^-$, but with a much lower intensity due to the low solubility of the crystalline product. Single crystals of **4** suitable for X-ray crystallography were grown from a THF/hexanes solution at room temperature. Anal. (%): Calcd. for $C_{72}H_{96}KN_5O_7U$, M_w = 1420.72, as in the formula of [K($C_{18}H_{36}N_2O_6$)][(1,3,5-[2-(1-AdN)C_6H_4]$_3C_6H_3$)UO]: C, 60.87; H, 6.81; N, 4.93. Found: C, 60.81; H, 6.90; N, 4.97. FTIR (KBr) $\tilde{\nu}$, cm^{-1}: 635 (w), 737 (m), 750 (m), 831 (w), 847 (w), 933 (m), 949 (m), 1082 (s), 1101 (s), 1130 (m), 1181 (w), 1239 (m), 1257 (m), 1276 (m), 1286 (m), 1357 (m), 1385 (w), 1440 (m), 1510 (w), 1582 (m), 1602 (w), 2849 (m), 2866 (m), 2883 (s), 2897 (s), 2956 (w).

Route B: 2e-Oxidation of [K(crypt)][(^{Ad}TPBN$_3$)U] (**2**)

NMR scale using N_2O as the oxidant: **2** (7.8 mg, 5.6 μmol, 1.0 equiv.) was dissolved in THF-*d*$_8$ (0.5 mL), and transferred to a J-Young tube. Upon freezing the solution, the headspace gas was removed under reduced pressure using the standard Schlenk technique. The tube was then warmed to ca. 0 °C, and filled with anhydrous N_2O (*ca.* 15 equiv.). ^{1}H NMR spectroscopy showed the full consumption of **2** and the quantitative formation of **4**. The resulting solution was transferred to a vial in the glove box, layered with Et_2O (1 mL) and hexanes (3 mL), and stayed undisturbed at room temperature. After 24 hours, the product precipitated out from solution, and was washed with hexanes and dried under reduced pressure to yield **4** as a greenish yellow solid (5.9 mg, 75%).

Preparation scale using pyridine-*N*-oxide as the oxidant: To a pre-cooled (–35 °C) solution of **2** (52 mg, 0.037 mmol, 1.0 equiv.) in THF (2 mL), a cold solution of pyridine-*N*-oxide (3.5 mg, 0.037 mmol, 1.0 equiv.) in THF (1 mL) was added dropwise. After stirring for 10 minutes, the volatiles were removed under reduced pressure. The remaining solid was triturated with hexanes (3 mL) at room temperature, and dried

under reduced pressure to yield **4** as a greenish yellow solid in an almost quantitative yield (52 mg, 99%).

Route C: 1e-Oxidation of (^{Ad}TPBN$_3$)U (**1**)

To a pre-cooled (–35 °C) solid mixture of **1** (50 mg, 0.051 mmol, 1.0 equiv.) and anhydrous KNO_2 (5.2 mg, 0.061 mmol, 1.2 equiv.) in a 20 mL vial, a pre-cooled (–35 °C) THF (5 mL) solution of 2.2.2-cryptand (19 mg, 0.051 mmol, 1.0 equiv.) was added dropwise. The mixture was then warmed to room temperature and vigorously stirred for 48 h. The resulting dark-red solution was filtered through Celite. The filtrate was concentrated to *ca.* 4 mL, layered with hexanes (6 mL), and stored at –35 °C for two days. The product precipitated out from solution, and was washed with toluene and hexanes and dried under reduced pressure to yield **4** as a greenish yellow solid (58 mg, 81%).

1.2.7. Synthesis of [Cp$^*_2$Co][(^{Ad}TPBN$_3$)UO] (4′)

To a pre-cooled (–35 °C) solution of **3** (0.044 g, 0.044 mmol, 1.0 equiv.) in THF (3 mL), the cold solution of Cp$^*_2$Co (0.017 g, 0.052 mmol, 1.2 equiv.) in THF (2 mL) was added dropwise. The mixture was warmed to room temperature and stirred for 20 minutes. The suspension was filtered through a Celite pad. The filtrate was concentrated to 2 mL, layered with hexanes (6 mL), and stored at –35 °C for two days. The product precipitated out from solution, and was washed with cold hexanes and dried under reduced pressure to yield **4′** as a dark brown solid (54 mg, 91%). Single crystals of **4′** suitable for X-ray crystallography were grown from a THF/hexanes solution at room temperature. ^{1}H NMR (400 MHz, C_5D_5N) δ, ppm: 61.96 (br s, FWHM = 280 Hz, 3H, C*H* of the anchor ring), 28.86 (s, 3H, C*H* of side rings), 2.70 (s, 3H, C*H* of side rings), 2.64 (s, 30H, C*H*$_3$ of [Cp$^*_2$Co]$^+$), –1.96 (br s, FWHM = 320 Hz, 9H, Ad), –5.61 (br s, FWHM = 43.9 Hz, 9H, Ad), –8.62 (s, 3H, C*H* of side rings), –9.40 (br s, FWHM = 126 Hz, 9H, Ad), –35.95 (s, 3H, C*H* of side rings), –46.49 (br s, FWHM = 720 Hz, 9H, Ad). One peak (9H) of Ad groups was not found in the range of –500 to +500 ppm probably due to signal broadening. Besides, splitting of C*H* peaks of side rings could not be observed under this NMR experiment condition. Anal. (%): Calcd. for $C_{74}H_{90}CoN_3OU$, M_w = 1334.52, as in the formula of [$(C_5Me_5)_2Co$][(1,3,5-[2-(1-AdN)C_6H_4]$_3C_6H_3$)UO]: C, 66.60; H, 6.80; N, 3.15. Found: C, 66.86; H, 7.03; N, 2.82. FTIR (KBr) $\tilde{\nu}$, cm^{-1}: 636 (w), 743 (m), 848 (w), 904 (m), 940 (m), 1026 (s), 1068 (s), 1140 (m), 1181 (m), 1244 (m), 1286 (m), 1307 (w), 1356 (w), 1384 (w), 1408 (w), 1450 (m), 1467 (w), 1510 (w), 1580 (m), 1602 (w), 2848 (m), 2903 (s).

1.2.8. Synthesis of [(^{Ad}TPBN$_3$)UO][SbF_6] (5)

To a stirring cold (–35 °C) solution of **3** (100 mg, 0.100 mmol, 1.0 equiv.) in CH_2Cl_2 (8 mL), a cold solution of $AgSbF_6$ (35 mg, 0.100 mmol, 1.0 equiv.) in CH_2Cl_2 (2 mL) was added dropwise in dark (to avoid light). After five minutes, the reaction mixture was filtered through a Celite-padded coarse-porosity fritted filter, and washed with cold CH_2Cl_2 (2 mL × 3). The volatiles were removed under reduced pressure. The resulting purple black solid was washed with toluene (1 mL), dissolved in CH_2Cl_2 (3 mL), and filtered through a Celite pad. The filtrate was layered with *n*-pentane (3 mL)

and stored at –35 °C overnight. Black crystals (co-crystallized with CH_2Cl_2) suitable for X-ray crystallography precipitated out from solution, washed with cold Et_2O, and dried under reduced pressure to yield **5** as a black solid (106 mg, 86%). ^{1}H NMR (CD_2Cl_2, 500 MHz, 298 K) δ, ppm: 10.60 (td, J = 7.8, 1.4 Hz, 3H, CH of side rings), 10.55 (dd, J = 7.7, 1.5 Hz, 3H, CH of side rings), 9.14 (s, 3H, CH of the anchor ring), 4.64 (br s, FWHM = 12.4 Hz, 9H, Ad), 1.64 (m, 9H, Ad), 1.49 (m, 9H, Ad), 1.47 (t, J = 7.5 Hz, 3H, CH of side rings), 0.62 (m, 9H, Ad), 0.40 (d, J = 8.4 Hz, 3H, CH of side rings), 0.05 (m, 9 H, Ad). ^{13}C NMR (125 MHz, CD_2Cl_2) δ, ppm: 199.12, 172.60, 163.50, 155.20, 138.08, 108.19, 101.52 (C_{aryl}), 77.25 (C_{Ad}), 68.69 (C_{aryl}), 36.79, 33.46, 12.21 (C_{Ad}). Detailed assignments are listed in the following section (Supplementary Figs. 55–56). Several peaks of ^{1}H and ^{13}C with unusual chemical shifts show a strong influence from spin-orbit coupling (SOC) effects on the NMR chemical shift of heavy atom-containing systems[8-10]. ^{19}F NMR (565 MHz, CD_2Cl_2, 298 K) δ, ppm: –124±14 (m: sextet ($J_{121Sb-19F}$ = 1947 Hz) & octet ($J_{123Sb-19F}$ = 1054 Hz), [SbF_6]$^-$). Anal. (%): Calcd. for $C_{55}H_{62}Cl_2F_6N_3OSbU$, M_w = 1325.80, as in the formula of [(1,3,5-[2-(1-AdN)C_6H_4]$_3$$C_6H_3$)UO][$SbF_6$]·$CH_2Cl_2$: C, 49.83; H, 4.71; N, 3.17. Found: C, 49.93; H, 4.99; N, 3.12. FTIR (KBr) ṽ, cm^{-1}: 433 (w), 503 (w), 547 (w), 586 (w), 657 (vs, SbF_6), 730 (m), 762 (m), 789 (m), 844 (w), 896 (w), 921 (w), 974 (w), 1059 (s), 1102 (m), 1122 (w), 1201 (m), 1299 (s), 1344 (w), 1385 (w), 1450 (m), 1555 (w), 1589 (w), 2851 (m), 2907 (s), 3059 (w).

1.2.9. Synthesis of (^{Ad}TPBN$_3$)UF (6)

Cold toluene (–35 °C, 5 mL) was added to a mixture of **1** (0.077 g, 0.077 mmol, 1.0 equiv.) and AgF (0.015 g, 0.12 mmol, 1.5 equiv.) with stirring. After stirring in dark over three days at room temperature, the suspension was filtered through a Celite-padded coarse-porosity fritted filter. The filtrate was collected and the volatiles were removed under reduced pressure. The oily residues were triturated with n-pentane (ca. 5 mL). The suspension was transferred to a medium-porosity fritted filter, washed with cold n-pentane (5 mL × 3), and dried under reduced pressure. **6** was obtained as a dark-red solid (0.068 g, 87%). Single crystals of **6** suitable for X-ray crystallography were grown from a hexanes/n-pentane solution at room temperature. ^{1}H NMR (400 MHz, C_6D_6) δ, ppm: 32.36 (s, 3H, CH of the anchor ring), 19.92 (d, J = 7.8 Hz, 3H, CH of side rings), 2.56 (t, J = 5.8 Hz, 3H, CH of side rings), –1.79 (br s, FWHM = 56.8 Hz, 9H, Ad), –3.14 (t, J = 6.2 Hz, 3H, CH of side rings), –3.70 (br s, FWHM = 25.7 Hz, 9H, Ad), –5.10 (br s, FWHM = 27.9 Hz, 9H, Ad), –23.35 (d, J = 8.0 Hz, 3H, CH of side rings), –25.02 (br s, FWHM = 160 Hz, 9H, Ad), –31.02 (br s, FWHM = 700 Hz, 9H, Ad). ^{19}F NMR (376 MHz, C_6D_6) δ, ppm: no signal could be observed in the range of –1100 to +900 ppm. Anal. (%): Calcd. for $C_{54}H_{60}FN_3U$, M_w = 1008.12, as in the formula of (1,3,5-[2-(1-AdN)C_6H_4]$_3$$C_6H_3$)UF: C, 64.34; H, 6.00; N, 4.17. Found: C, 64.40; H, 6.02; N, 3.88. FTIR (KBr) ṽ, cm^{-1}: 478 (w), 492 (w), 613 (w), 646 (w), 666 (w), 715 (w), 743 (m), 760 (w), 782 (w), 797 (w), 825 (w), 844 (m), 892 (w), 937 (w), 983 (w), 1046 (w), 1076 (m), 1100 (w), 1119 (w), 1182 (w), 1226 (m), 1279 (w), 1303 (w), 1355 (w), 1385 (w), 1445 (m), 1466 (w), 1511 (w), 1591 (m), 2848 (m), 2903 (s).

1.2.10. Synthesis of (^{Ad}TPBN$_3$)UI (7)

To a solution of **1** (0.103 g, 0.104 mmol, 1.0 equiv.) in toluene (5 mL), a solution of 1,2-diiodoethane (0.015 g, 0.053 mmol, 0.5 equiv.) in toluene (1 mL) was added dropwise with stirring. The resulting dark red solution was stirred at temperature for 10 minutes. The volatiles were then removed under reduced pressure. The remaining solid was washed with cold *n*-pentane (2 mL × 3) and dried under reduced pressure to yield **7** as a brownish red solid (0.110 g, 95%). Single crystals of **7** suitable for X-ray crystallography were grown from an *n*-pentane solution at room temperature. ^{1}H NMR (400 MHz, C_6D_6) δ, ppm: 8.56 (t, J = 7.5 Hz, 3H, C*H* of side rings), 8.03 (d, J = 7.3 Hz, 3H, C*H* of side rings), 5.03 (t, J = 7.3 Hz, 3H, C*H* of side rings), 3.39 (d, J = 8.1 Hz, 3H, C*H* of side rings), 2.34 (br s, FWHM = 17.0 Hz, 9H, Ad), 1.39 (s, 3H, C*H* of the anchor ring), 0.86–1.00 (m, 18H, Ad), –0.81 (br s, FWHM = 48.0 Hz, 9H, Ad), –2.31 (br s, FWHM = 520 Hz, 9H, Ad). Anal. (%): Calcd. for $C_{56.5}H_{66}IN_3U$, M_w = 1152.10, as in the formula of (1,3,5-[2-(1-AdN)C_6H_4]$_3C_6H_3$)UI·0.5C_5H_{12} (*n*-pentane): C, 58.90; H, 5.77; N, 3.65. Found: C, 59.10; H, 5.76; N, 3.44. FTIR (KBr) $\tilde{\nu}$, cm^{-1}: 427 (w), 475 (w), 494 (w), 613 (w), 641 (w), 664 (w), 712 (w), 729 (w), 745 (w), 763 (m), 783 (w), 797 (w), 844 (m), 860 (w), 891 (w), 929 (w), 1065 (m), 1101 (w), 1198 (m), 1220 (m), 1276 (w), 1289 (w), 1301 (w), 1354 (w), 1399 (w), 1447 (m), 1467 (m), 1592 (m), 2487 (m), 2903 (s).

1.3. Trapping Experiment

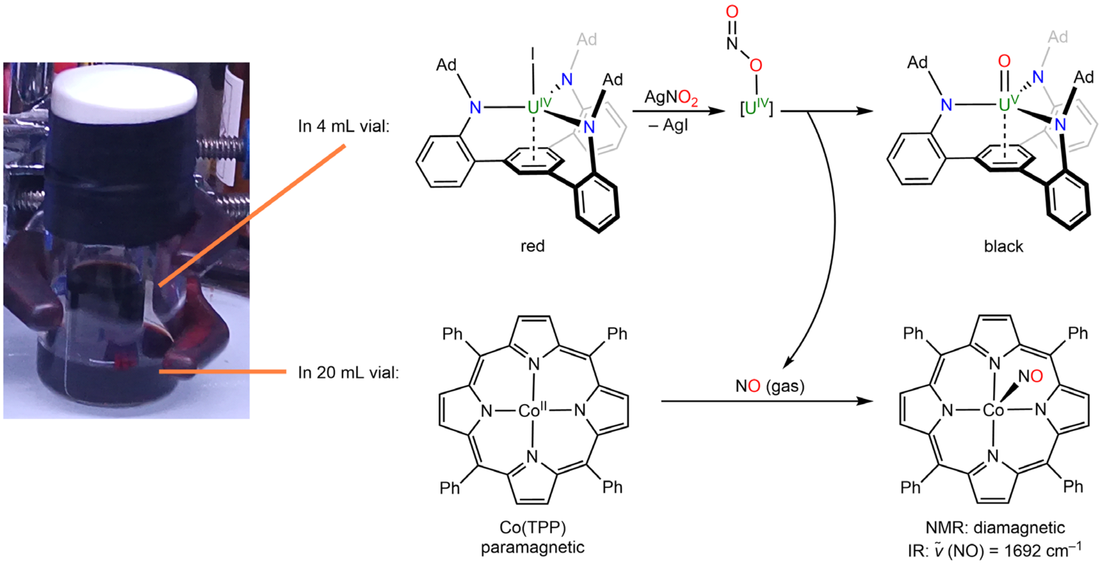

Supplementary Fig. 1. Representation of the apparatus (left) and the chemical equations (right) relevant to the trapping experiment.

In a 20 mL vial, Co(TPP) (cobalt(II) 5,10,15,20-tetraphenylporphyrin; 7.0 mg, 0.010 mmol, 0.8 equiv.) was dissolved in THF (1.5 mL). In a 4 mL vial (fits inside the 20 mL vial), the uranium(IV) iodide **7** (14.5 mg, 0.013 mmol, 1.0 equiv.) and $AgNO_2$ (2.0 mg, 0.014 mmol, 1.1 equiv.) were weighted and a stirring bar was added. The 4 mL vial was placed inside the 20 mL vial, then added cold THF (–35 °C, 2 mL), and

the 20 mL vial was sealed as the above figure shows. The red mixture in the 4 mL vial was stirred steadily at room temperature, while the 20 mL vial was kept undisturbed.

After three hours, the mixture in the 4 mL vial turned black. By taking an aliquot of the outer solution for ^{1}H NMR spectroscopic measurement (Supplementary Fig. 65), we found that the paramagnetic peaks of Co(TPP) (range: 9.6–16.5 ppm in $CDCl_3$, 400 MHz)[11,12] were not observed. Instead, the major peaks were in the diamagnetic region and the chemical shifts are consistent with the reported values for the NO-bound complex, Co(TPP)(NO)[11,12]. In addition, an aliquot of the outer solution was dried and analysed by IR vibrational spectroscopy (KBr disk), and the characteristic peak of N–O stretching of Co(TPP)(NO) was observed at $\tilde{\nu}$(NO) = 1697 cm^{-1} (Supplementary Fig. 25), matching well with the literature values[13,14].

The possible chemical reactions involved in this trapping experiment was illustrated in the above figure. The results support the release of NO upon the transformation from **7** to **3**, and are consistent with a proposed mechanism involving the formation of a [U–ONO] intermediate[15].

2. X-ray Crystallography

2.1. General. All structures were solved by the intrinsic phasing method with SHELXT[16] and refined by full-matrix least-squares procedures utilizing SHELXL[17,18] within Olex2 crystallographic software package[19]. The PLATON[20] routine SQUEEZE[21] was used in the structure refinement of **1**, **2**·0.5THF, and **3**·C_7H_8.

2.2. Details of restraints and SQUEEZE

All hydrogen atoms were refined isotropically with riding coordinates using appropriate HFIX constraints. All non-hydrogen atoms were refined anisotropically. Other refinement description and the SQUEEZE information are detailed below for each single crystal data reported in this work.

H_3($^{Ad}TPBN_3$): There is no restraint. No solvent mask was used.

($^{Ad}TPBN_3$)U (**1**): There is no restraint. A solvent mask was calculated and 149 electrons were found in a volume of 541 Å^3 in a void per unit cell. This is consistent with the presence of 1.8[$C_4H_{10}O$] (Et_2O) per Formula Unit, which account for 151 electrons per unit cell.

[K(crypt)][($^{Ad}TPBN_3$)U]·0.5THF (**2**·0.5THF): There is no restraint. A solvent mask was calculated and 181 electrons were found in a volume of 949 Å^3 in 5 voids per unit cell. This is consistent with the presence of total 2.43[C_4H_8O] (THF) per Formula Unit, which account for 194 electrons per unit cell.

($^{Ad}TPBN_3$)UO·C_7H_8 (**3**·C_7H_8): There is no restraint. A solvent mask was calculated and 78 electrons were found in a volume of 414 Å^3 in a void per unit cell. This is consistent with the presence of 0.5[C_5H_{12}] (*n*-pentane) per Formula Unit, which account for 84 electrons per unit cell.

($^{Ad}TPBN_3$)UO (**3**): There is no restraint. No solvent mask was used.

[K(crypt)][($^{Ad}TPBN_3$)UO] (**4**): The crystal asymmetric unit contains 1/3 of the molecule. There is no restraint. No solvent mask was used.

[Cp^*_2Co][($^{Ad}TPBN_3$)UO]·THF (**4′**·THF): For better modeling, there are a few restraints used in the refinement. The similarity restraint SIMU was applied for C_{methyl} atoms of the Cp^* rings (C63–C74). The similarity restraint SADI was applied for the C–C bonds of the anchoring arene (C1–C6) with sigma of 0.02. The rigid bond restraint DELU was set for the U–O bond with sigma of 0.001. No solvent mask was used.

[($^{Ad}TPBN_3$)UO][SbF_6]·2CH_2Cl_2 (**5**·2CH_2Cl_2): The disordered fluorine atoms of the counter anion $[SbF_6]^-$ are modeled by two parts with total occupancy of 1, and converged at the occupancy of 0.54 and 0.46 for each part, respectively. No solvent mask was used.

($^{Ad}TPBN_3$)UF (**6**): There is no restraint. No solvent mask was used.

($^{Ad}TPBN_3$)UI (**7**): There is no restraint. No solvent mask was used.

XPhos: There is no restraint. No solvent mask was used.

2.3. CCDC Deposition. All crystal structures reported in this work have been deposited to the Cambridge Crystallographic Data Center (CCDC) with the following deposition numbers:

CCDC-2245010: $H_3(^{Ad}TPBN_3)$
CCDC-2245011: $(^{Ad}TPBN_3)U$ (**1**)
CCDC-2245012: $[K(crypt)][(^{Ad}TPBN_3)U]\cdot 0.5THF$ (**2**·0.5THF)
CCDC-2245013: $(^{Ad}TPBN_3)UO\cdot C_7H_8$ (**3**·C_7H_8)
CCDC-2245014: $(^{Ad}TPBN_3)UO$ (**3**)
CCDC-2245015: $[K(crypt)][(^{Ad}TPBN_3)UO]$ (**4**)
CCDC-2245016: $[Cp^*_2Co][(^{Ad}TPBN_3)UO]\cdot THF$ (**4′**·THF)
CCDC-2245017: $[(^{Ad}TPBN_3)UO][SbF_6]\cdot 2CH_2Cl_2$ (**5**·$2CH_2Cl_2$)
CCDC-2245018: $(^{Ad}TPBN_3)UF$ (**6**)
CCDC-2245019: $(^{Ad}TPBN_3)UI$ (**7**)
CCDC-2245020: XPhos

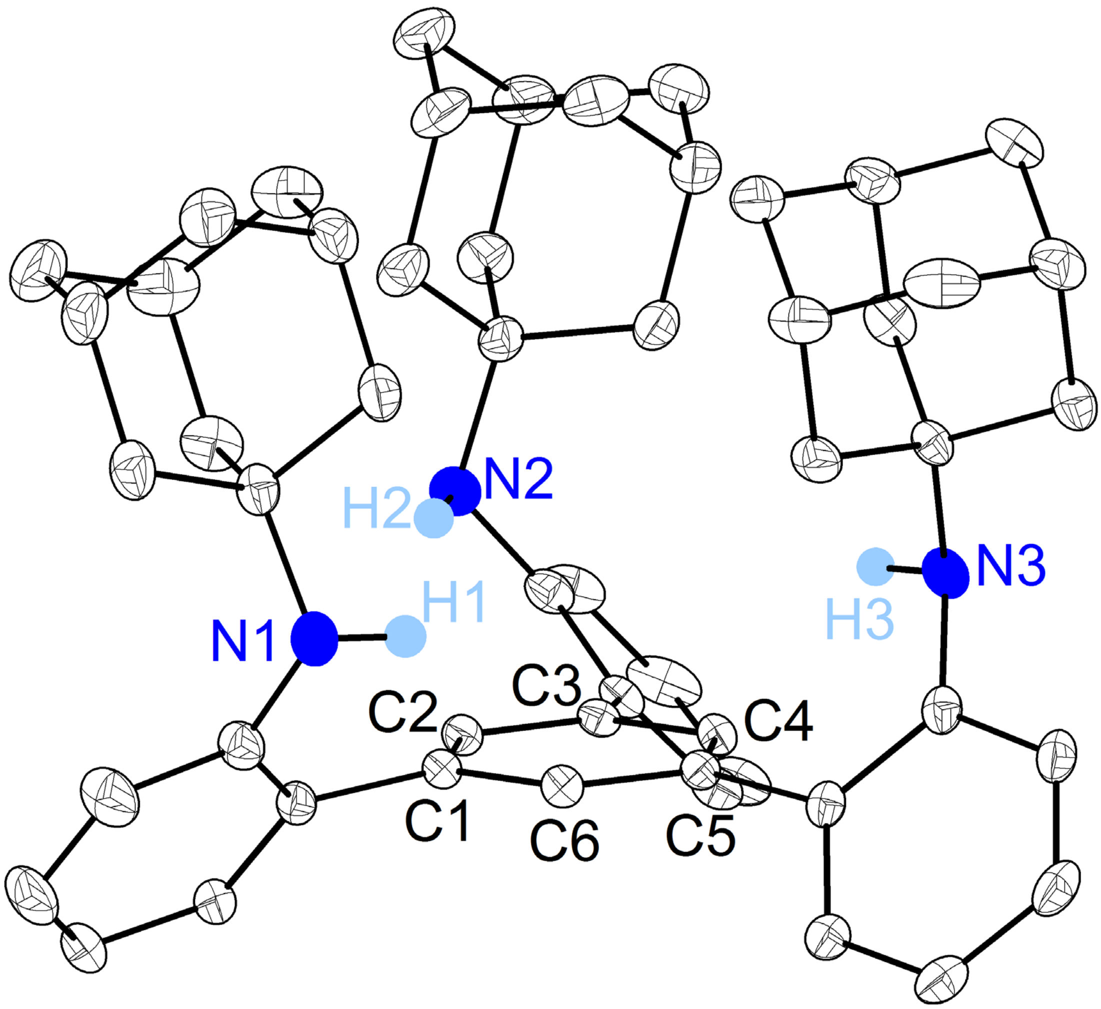

Supplementary Fig. 2. Representation of H_3(^{Ad}TPBN_3) with thermal ellipsoids set at 50% probability. Hydrogen (except for N*H*) atoms were omitted for clarity. Selected distances (Å) and angles (°): C1–C2 1.3964(12), C2–C3 1.3988(18), C3–C4 1.3979(21), C4–C5 1.3943(12), C5–C6 1.3993(19), C6–C1 1.3979(21); three dihedral angles between side rings and the anchor ring 51.813(31), 49.831(35), 54.214(33).

Single crystals suitable for X-ray crystallography were grown from a Et_2O solution. A total of 23506 reflections (–17 ≤ h ≤ 17, –18 ≤ k ≤ 17, –13 ≤ l ≤ 19) were collected at T =180.15 K with $2\theta_{max}$ = 58.744°, of which 10198 were unique. The residual peak and hole electron density were 0.27 and –0.21 eÅ^{-3}. The least-squares refinement converged normally with residuals of R_1 = 0.0454 and GOF = 1.038. Crystal and refinement data for H_3(^{Ad}TPBN_3): formula $C_{54}H_{63}N_3$, space group P–1, a = 13.0156(4) Å, b = 13.4914(3) Å, c = 14.0146(3) Å, α = 91.216(2)°, β = 106.490(2)°, γ = 116.398°, V = 2082.30(10) Å^3, Z = 2, μ = 0.069 mm^{-1}, F(000) = 816.0, R_1 = 0.0622 and wR_2 = 0.1202 (based on all data).

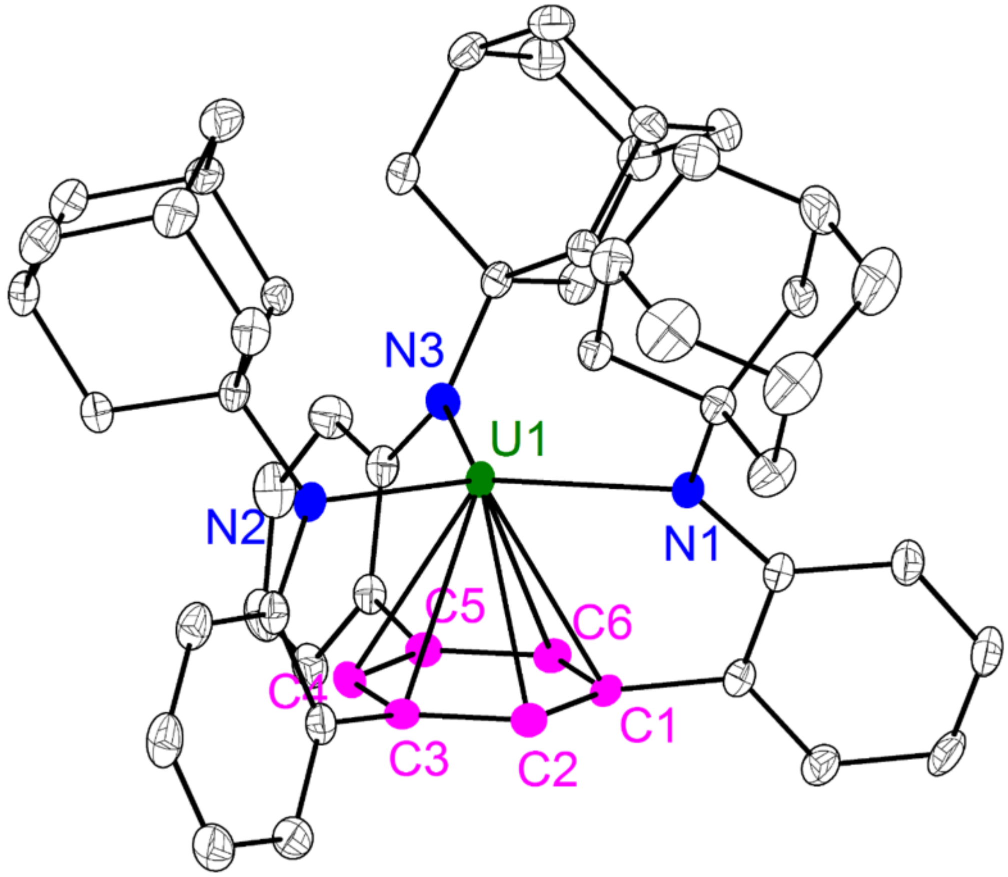

Supplementary Fig. 3. Representation of ($^{Ad}TPBN_3$)U (**1**) with thermal ellipsoids set at 50% probability. Hydrogen atoms were omitted for clarity. Selected distances (Å) and angles (°): U1–N1 2.4133(26), U1–N2 2.4291(24), U1–N3 2.4264(23), U1–C1 2.7325(29), U1–C2 2.7418(30), U1–C3 2.7336(30), U1–C4 2.7282(32), U1–C5 2.7220(22), U1–C6 2.7196(26), U1–$C_{centroid}$ 2.3385(3), U1–$3N_{plane}$ –0.1704(3) (U–$3N_{plane}$: the distance of uranium to the plane defined by three amide nitrogen atoms), C1–C2 1.4173(40), C2–C3 1.3923(52), C3–C4 1.4196(45), C4–C5 1.398(4), C5–C6 1.4131(46), C6–C1 1.4069(45); N1–U1–N2 119.854(78), N2–U1–N3 119.586(77), N3–U1–N1 119.09(8).

Single crystals suitable for X-ray crystallography were grown from a Et_2O solution. A total of 51089 reflections ($-15 \le h \le 16$, $-20 \le k \le 20$, $-20 \le l \le 21$) were collected at T = 180.15 K with $2\theta_{max}$ = 60.396°, of which 13156 were unique. The residual peak and hole electron density were 2.25 and –1.10 eÅ^{-3}. The least-squares refinement converged normally with residuals of R_1 = 0.0316 and GOF = 1.035. Crystal and refinement data for ($^{Ad}TPBN_3$)U: formula $C_{54}H_{60}N_3U$, space group P–1, a = 11.6503(2) Å, b = 14.5422(2) Å, c = 15.3316(3) Å, α = 94.2250(10)°, β = 105.144(2)°, γ = 90.4100(10)°, V = 2499.53(8) Å^3, Z = 2, μ = 3.282 mm^{-1}, F(000) = 994.0, R_1 = 0.0406 and wR_2 = 0.0754 (based on all data).

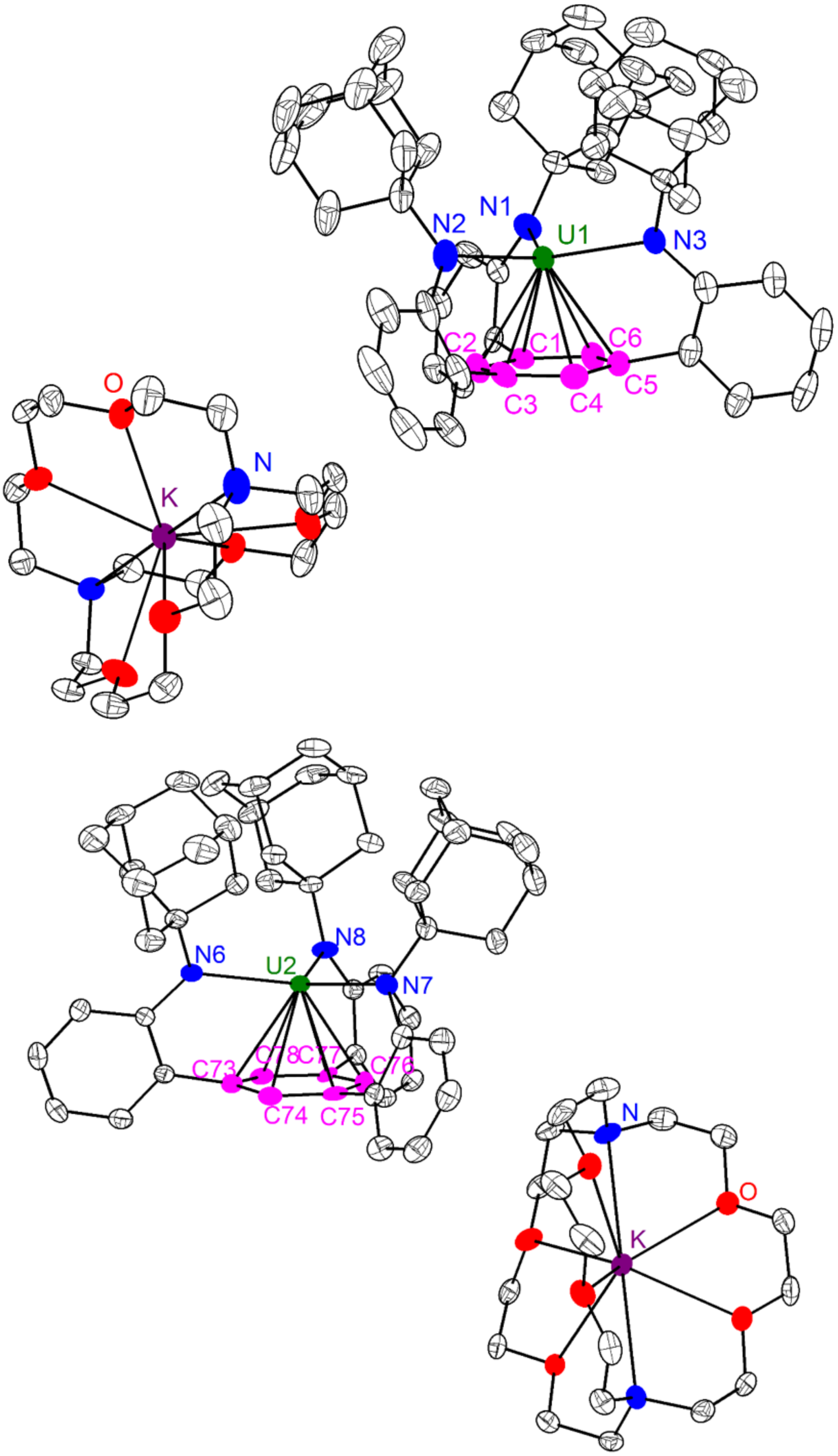

Supplementary Fig. 4 (top & bottom). Representation of $[K(crypt)][(^{Ad}TPBN_3)U]\cdot 0.5THF$ (**2**·0.5THF) with thermal ellipsoids set at 50% probability. Hydrogen atoms and co-crystallized molecules of THF were omitted for clarity. Selected distances (Å) and angles (°): For independent molecule 1 (top): U1–

N1 2.4723(27), U1–N2 2.4622(26), U1–N3 2.4745(31), U1–C1 2.5906(39), U1–C2 2.5943(44), U1–C3 2.5884(39), U1–C4 2.6335(33), U1–C5 2.5730(32), U1–C6 2.5790(38), U1–$C_{centroid}$ 2.1710(5), U1–3N_{plane} –0.3516(5), C1–C2 1.4081(54), C2–C3 1.4460(49), C3–C4 1.4087(54), C4–C5 1.3882(55), C5–C6 1.4344(51), C6–C1 1.4267(55); N1–U1–N2 118.676(91), N2–U1–N3 115.885(89), N3–U1–N1 119.460(94). For independent molecule 2 (bottom): U2–N6 2.4669(26), U2–N7 2.5037(28), U2–N8 2.4822(25), U2–C73 2.5945(40), U2–C74 2.6383(40), U2–C75 2.5947(36), U2–C76 2.5637(36), U2–C77 2.5936(31), U2–C78 2.6058(33), U2–$C_{centroid}$ 2.1790(5), U2–3N_{plane} –0.3261(5), C73–C74 1.4225(42), C74–C75 1.4017(55), C75–C76 1.4312(48), C76–C77 1.4171(41), C77–C78 1.4057(47), C78–C73 1.4196(48); N6–U2–N7 118.566(89), N7–U2–N8 117.750(94), N8–U2–N6 118.596(88).

Single crystals suitable for X-ray crystallography were grown from a THF/Et_2O/hexanes solution. A total of 106512 reflections (–16 ≤ h ≤ 17, –31 ≤ k ≤ 31, –34 ≤ l ≤ 33) were collected at T = 180.15 K with $2\theta_{max}$ = 59.158°, of which 36454 were unique. The residual peak and hole electron density were 1.51 and –1.50 $eÅ^{-3}$. The least-squares refinement converged normally with residuals of R_1 = 0.0398 and GOF = 1.026. Crystal and refinement data for 2[K(crypt)][($^{Ad}TPBN_3$)U]·THF: formula $C_{148}H_{200}K_2N_{10}O_{13}U_2$, space group P–1, a = 12.9095(2) Å, b = 23.2609(3) Å, c = 25.3412(4) Å, α = 105.0300(10)°, β = 90.3280(10)°, γ = 100.6780(10)°, V = 7210.43(19) $Å^3$, Z = 2, μ = 2.362 mm^{-1}, F(000) = 2968.0, R_1 = 0.0606 and wR_2 = 0.1045 (based on all data).

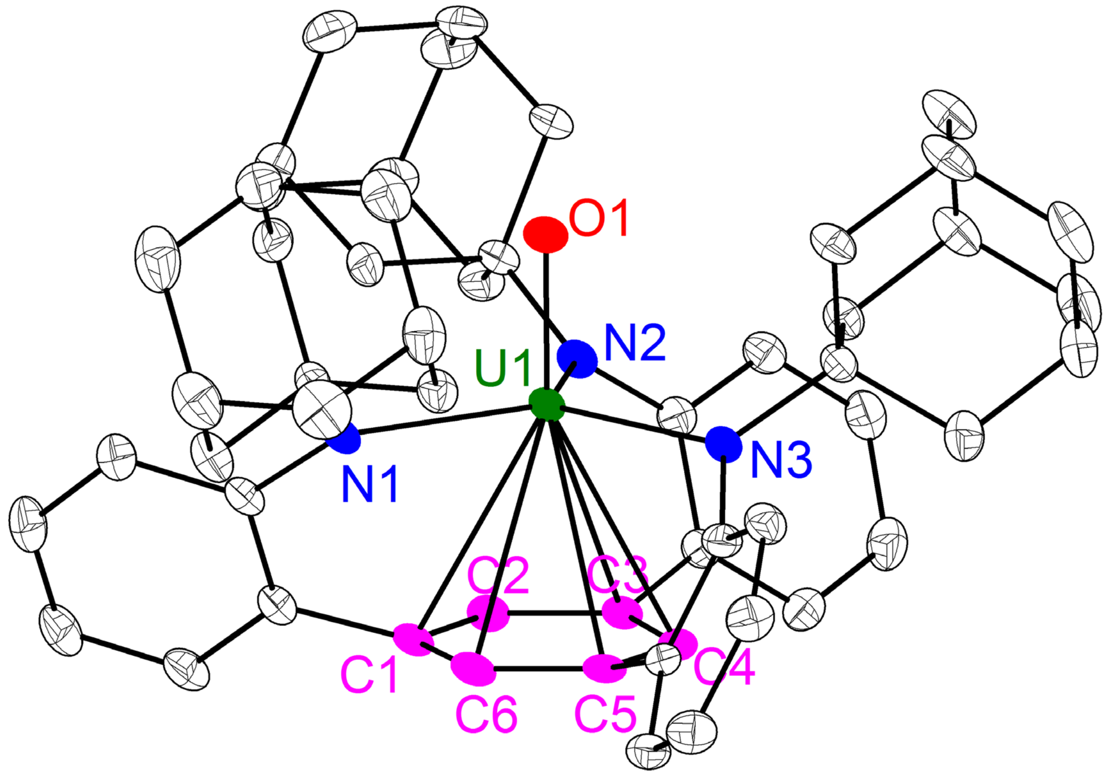

Supplementary Fig. 5. Representation of (^{Ad}TPBN$_3$)UO·C_7H_8 (**3**·C_7H_8) with thermal ellipsoids set at 50% probability. Hydrogen atoms and co-crystallized molecules of toluene were omitted for clarity. Selected distances (Å) and angles (°): U1–O1 1.8360(26), U1–N1 2.3424(24), U1–N2 2.3121(28), U1–N3 2.3113(26), U1–C1 2.9305(33), U1–C2 2.9077(37), U1–C3 2.8853(39), U1–C4 2.9149(35), U1–C5 2.9151(31), U1–C6 2.9371(31), U1–$C_{centroid}$ 2.5563(4), U1–3N_{plane} +0.0931(4), C1–C2 1.3898(43), C2–C3 1.4146(40), C3–C4 1.3913(41), C4–C5 1.4075(44), C5–C6 1.3855(40), C6–C1 1.4196(41); N1–U1–N2 120.032(98), N2–U1–N3 118.348(93), N3–U1–N1 121.141(95).

Single crystals suitable for X-ray crystallography were grown from a toluene/*n*-pentane solution. A total of 62485 reflections (–20 ≤ h ≤ 19, –17 ≤ k ≤ 16, –28 ≤ l ≤ 28) were collected at T = 180.15 K with $2\theta_{max}$ = 52.746°, of which 10342 were unique. The residual peak and hole electron density were 2.09 and –0.77 eÅ^{-3}. The least-squares refinement converged normally with residuals of R_1 = 0.0289 and GOF = 1.026. Crystal and refinement data for (^{Ad}TPBN$_3$)UO·C_7H_8: formula $C_{61}H_{68}N_3OU$, space group $P2_1/n$, a = 16.6702(3) Å, b = 13.7504(3) Å, c = 23.1478(6) Å, β = 107.260(2)°, V = 5067.0(2) Å^3, Z = 4, μ = 3.247 mm^{-1}, F(000) = 2220.0, R_1 = 0.0407 and wR_2 = 0.0717 (based on all data).

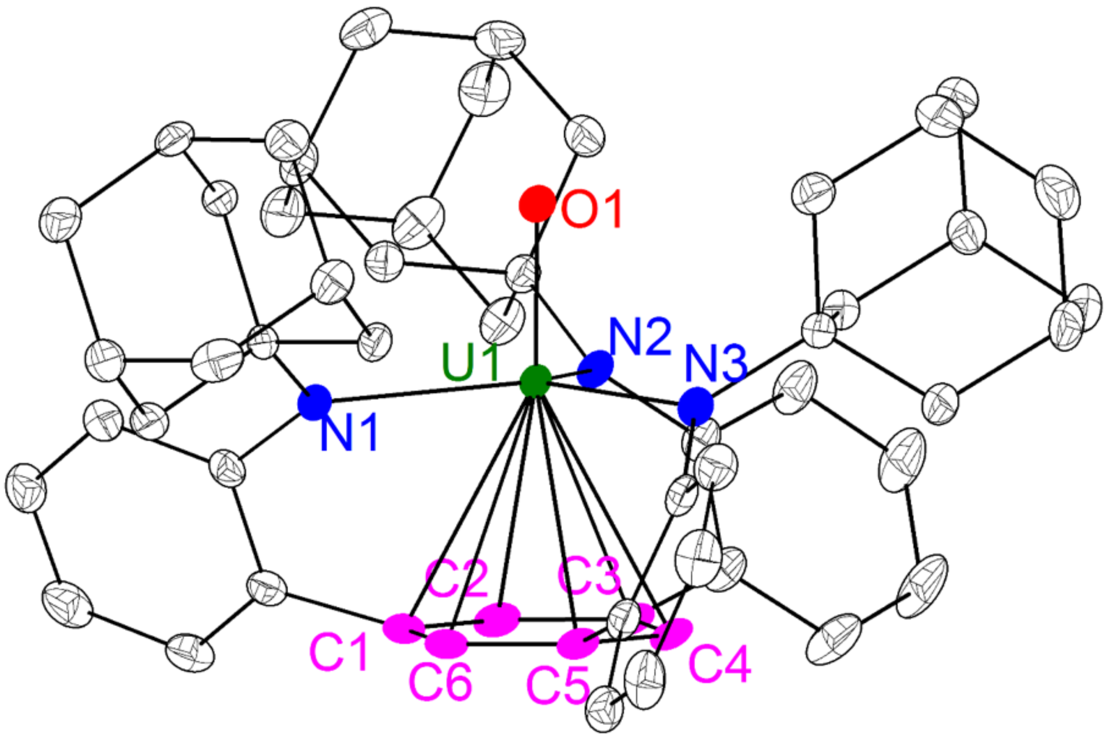

Supplementary Fig. 6. Representation of ($^{Ad}TPBN_3$)UO (**3**) with thermal ellipsoids set at 50% probability. Hydrogen atoms were omitted for clarity. Selected distances (Å) and angles (°): U1–O1 1.8287(17), U1–N1 2.3209(23), U1–N2 2.3271(25), U1–N3 2.3023(21), U1–C1 2.9177(24), U1–C2 2.9389(23), U1–C3 2.9263(27), U1–C4 2.9256(24), U1–C5 2.9027(25), U1–C6 2.9187(26), U1–$C_{centroid}$ 2.5654(4), U1–$3N_{plane}$ +0.1223(4), C1–C2 1.3826(41), C2–C3 1.4085(37), C3–C4 1.3829(40), C4–C5 1.4217(40), C5–C6 1.3829(34), C6–C1 1.4111(33); N1–U1–N2 118.34(8), N2–U1–N3 119.366(78), N3–U1–N1 121.464(79).

Single crystals suitable for X-ray crystallography were grown from a benzene/*n*-pentane solution. A total of 31639 reflections (–15 ≤ h ≤ 14, –25 ≤ k ≤ 23, –27 ≤ l ≤ 21) were collected at T = 180.15 K with $2\theta_{max}$ = 58.81°, of which 10717 were unique. The residual peak and hole electron density were 1.69 and –0.54 eÅ^{-3}. The least-squares refinement converged normally with residuals of R_1 = 0.0251 and GOF = 1.044. Crystal and refinement data for ($^{Ad}TPBN_3$)UO: formula $C_{54}H_{60}N_3OU$, space group $P2_1/c$, a = 10.9609(2) Å, b = 18.5423(3) Å, c = 21.1803(5) Å, β = 100.713(2)°, V = 4229.66(15) Å^3, Z = 4, μ = 3.882 mm^{-1}, F(000) = 2020.0, R_1 = 0.0369 and wR_2 = 0.0583 (based on all data).

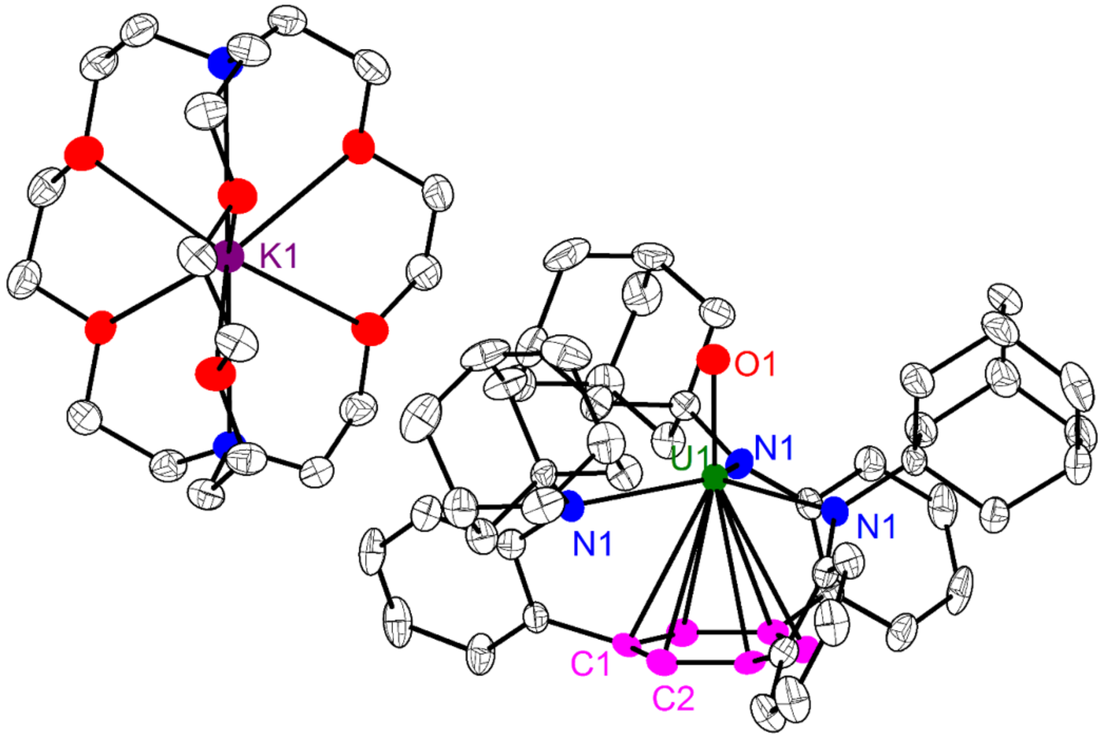

Supplementary Fig. 7. Representation of [K(crypt)][(^{Ad}TPBN$_3$)UO] (**4**) with thermal ellipsoids set at 50% probability. Hydrogen atoms were omitted for clarity. Selected distances (Å) and angles (°): U1–O1 1.8736(37), U1–N1 2.4214(43), U1–C1 3.0226(35), U1–C2 3.0336(36), U1–C$_{centroid}$ 2.6857(4), U1–3N$_{plane}$ +0.2531(4), C1–C2 1.4168(57), 1.3809(56); N1–U1–N1' 118.921(129).

Single crystals suitable for X-ray crystallography were grown from a THF/hexanes solution. A total of 6274 reflections (–17 ≤ h ≤ 22, –23 ≤ k ≤ 9, –23 ≤ l ≤ 24) were collected at T = 180.15 K with 2θ_{max} = 59.056°, of which 4511 were unique. The residual peak and hole electron density were 1.76 and –0.96 eÅ^{-3}. The least-squares refinement converged normally with residuals of R_1 = 0.0205 and GOF = 1.036. Crystal and refinement data for [K(crypt)][(^{Ad}TPBN$_3$)UO]: formula $C_{72}H_{96}KN_5O_7U$, space group $R3$, a = 17.4587(4) Å, c = 18.5858(6) Å, V = 4906.1(3) Å^3, Z = 3, μ = 2.603 mm^{-1}, $F(000)$ = 2190.0, R_1 = 0.0205 and wR_2 = 0.0448 (based on all data).

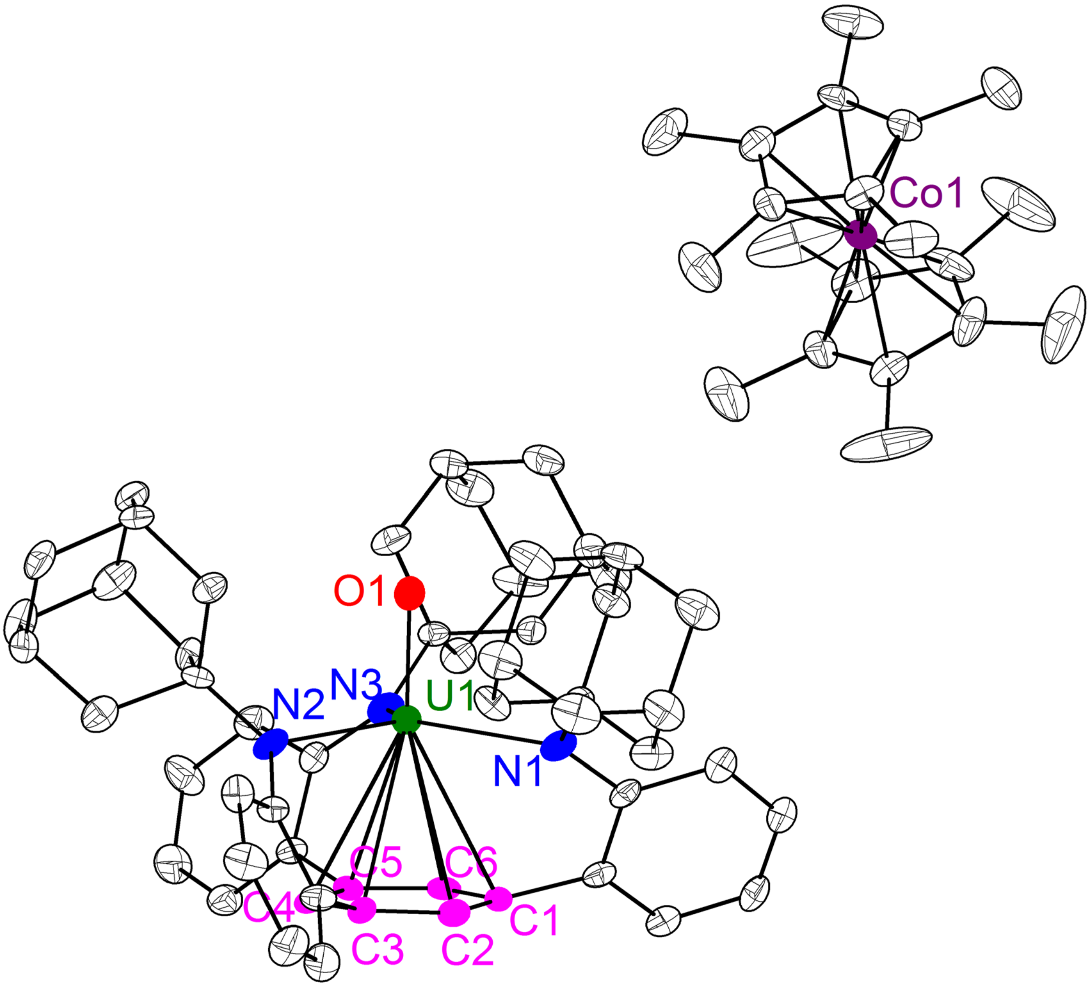

Supplementary Fig. 8. Representation of [Cp*$_2$Co][(^{Ad}TPBN$_3$)UO]·THF (**4′**·THF) with thermal ellipsoids set at 50% probability. Hydrogen atoms were omitted for clarity. Selected distances (Å) and angles (°): U1–O1 1.8508(71), U1–N1 2.4323(84), U1–N2 2.4524(86), U1–N3 2.4181(81), U1–C1 2.9523(94), U1–C2 2.9913(108), U1–C3 2.9603(95), U1–C4 3.0001(93), U1–C5 3.0027(91), U1–C6 2.9666(95), U1–C$_{centroid}$ 2.6348(4), U1–3N$_{plane}$ +0.1890(4), C1–C2 1.4086(149), C2–C3 1.3696(143), C3–C4 1.3968(134), C4–C5 1.3899(120), C5–C6 1.3906(131), C6–C1 1.3869(148); N1–U1–N2 118.510(284), N2–U1–N3 118.943(278), N3–U1–N1 120.755(277).

Single crystals suitable for X-ray crystallography were grown from a THF/hexanes solution. A total of 54028 reflections (–15 ≤ h ≤ 15, –31 ≤ k ≤ 31, –21 ≤ l ≤ 21) were collected at T = 180.00(10) K with 2θ_{max} = 50.052, of which 11650 were unique. The residual peak and hole electron density were 4.30 and –1.54 eÅ^{-3}. The least-squares refinement converged normally with residuals of R_1 = 0.0765 and GOF = 1.104. Crystal and refinement data for [Cp*$_2$Co][(^{Ad}TPBN$_3$)UO]·THF: formula $C_{78}H_{98}CoN_3O_2U$, space group $P2_1/n$, a = 13.3624(5) Å, b = 26.7946(8) Å, c = 18.4675(7) Å, β = 90.015(3)°, V = 6612.1(4) Å^3, Z = 4, μ = 2.746 mm^{-1}, F(000) = 2888.0, R_1 = 0.1074 and wR_2 = 0.1769 (based on all data).

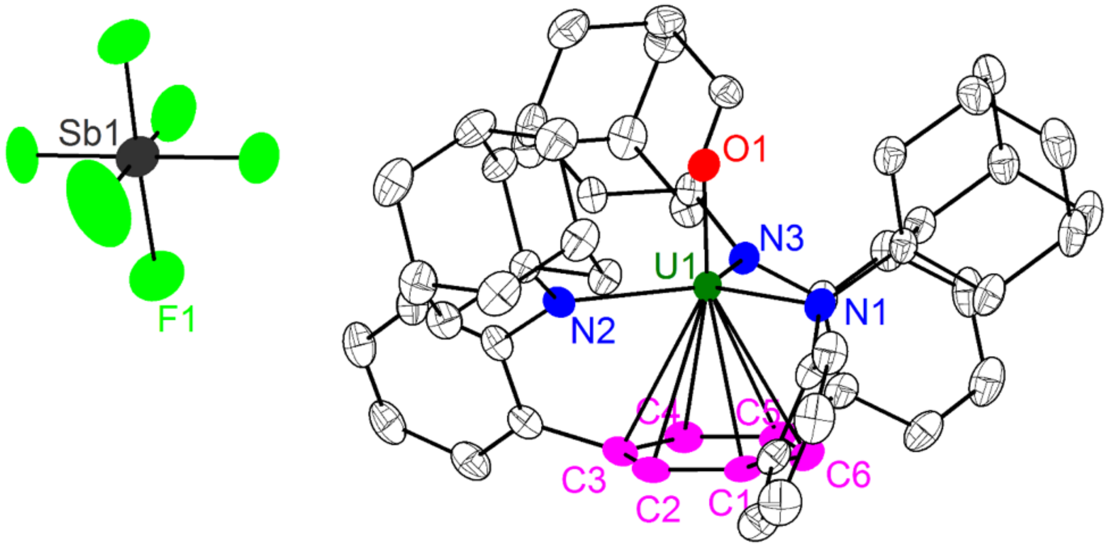

Supplementary Fig. 9. Representation of [(^{Ad}TPBN$_3$)UO][SbF$_6$]·2CH$_2$Cl$_2$ (**5**·2CH$_2$Cl$_2$) with thermal ellipsoids set at 50% probability. Hydrogen atoms, one set of disordered fluorine atoms of [SbF$_6$]$^-$, and co-crystallized molecules of CH$_2$Cl$_2$ were omitted for clarity. Selected distances (Å) and angles (°): U1–O1 1.8178(19), U1–N1 2.2753(34), U1–N2 2.2697(31), U1–N3 2.2755(27), U1–C1 2.8555(32), U1–C2 2.8684(35), U1–C3 2.8439(39), U1–C4 2.8608(37), U1–C5 2.8518(28), U1–C6 2.8809(28), U1–C$_{centroid}$ 2.4925(3), U1–3N$_{plane}$ +0.0364(3), C1–C2 1.3895(62), C2–C3 1.4182(63), C3–C4 1.3950(51), C4–C5 1.4159(62), C5–C6 1.3888(63), C6–C1 1.4117(51); N1–U1–N2 120.799(102), N2–U1–N3 119.42(10), N3–U1–N1 119.705(100).

Single crystals suitable for X-ray crystallography were grown from a CH$_2$Cl$_2$/*n*-pentane solution. A total of 32598 reflections (–16 ≤ h ≤ 16, –17 ≤ k ≤ 17, –15 ≤ l ≤ 19) were collected at T = 180.15 K with $2\theta_{max}$ = 52.744°, of which 10820 were unique. The residual peak and hole electron density were 1.84 and –1.90 eÅ^{-3}. The least-squares refinement converged normally with residuals of R_1 = 0.0280 and GOF = 1.053. Crystal and refinement data for [(^{Ad}TPBN$_3$)UO][SbF$_6$]·2CH$_2$Cl$_2$: formula C$_{56}$H$_{64}$Cl$_4$F$_6$N$_3$OSbU, space group P–1, a = 13.4938(4) Å, b = 14.1867(4) Å, c = 15.4238(4) Å, α = 66.506(3)°, β = 82.626(2)°, γ = 78.965(2)°, V = 2653.34(14) Å^3, Z = 2, μ = 3.823 mm^{-1}, F(000) = 1388.0, R_1 = 0.0329 and wR_2 = 0.0664 (based on all data).

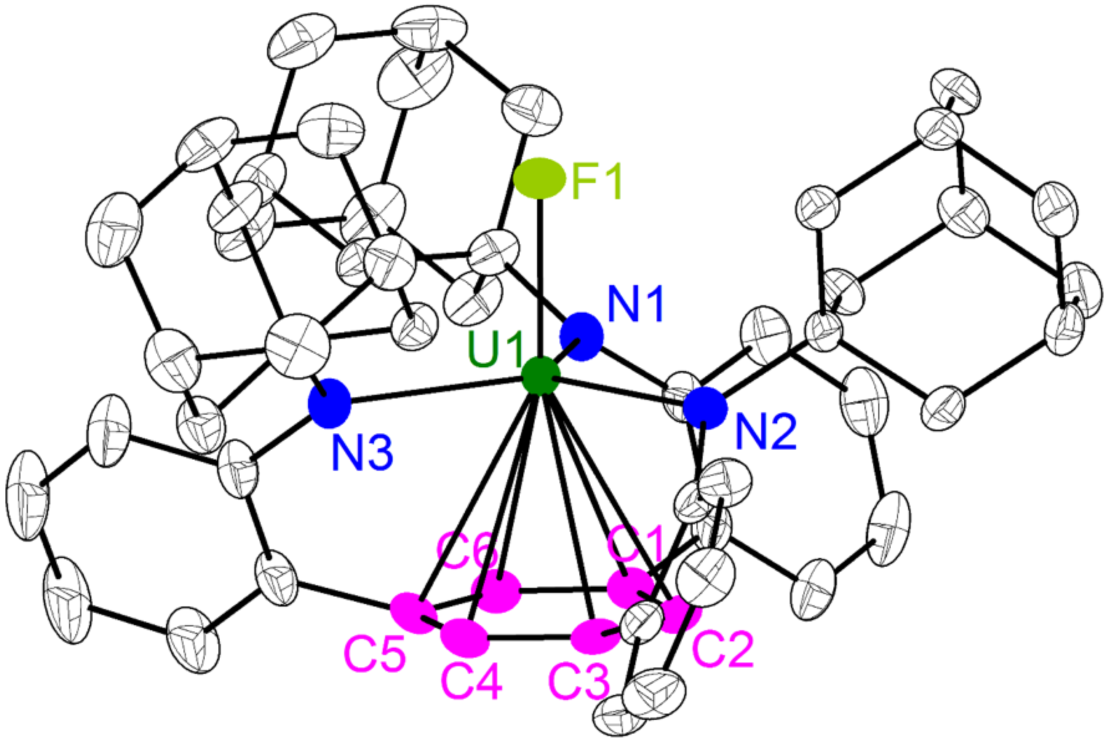

Supplementary Fig. 10. Representation of ($^{Ad}TPBN_3$)UF (**6**) with thermal ellipsoids set at 50% probability. Hydrogen atoms were omitted for clarity. Selected distances (Å) and angles (°): U1–F1 2.0897(17), U1–N1 2.3092(22), U1–N2 2.3233(25), U1–N3 2.3377(26), U1–C1 2.8717(29), U1–C2 2.8926(31), U1–C3 2.8932(29), U1–C4 2.9088(31), U1–C5 2.8996(31), U1–C6 2.8941(30), U1–$C_{centroid}$ 2.5324(4), U1–3N_{plane} +0.0700(4), C1–C2 1.3859(41), C2–C3 1.4108(47), C3–C4 1.3882(48), C4–C5 1.4078(41), C5–C6 1.3846(47), C6–C1 1.4203(49); N1–U1–N2 121.466(86), N2–U1–N3 118.901(86), N3–U1–N1 119.362(80).

Single crystals suitable for X-ray crystallography were grown from a hexanes/*n*-pentane solution. A total of 32272 reflections (–14 ≤ h ≤ 14, –18 ≤ k ≤ 25, –27 ≤ l ≤ 29) were collected at T = 180.15 K with $2\theta_{max}$ = 58.828°, of which 10710 were unique. The residual peak and hole electron density were 2.97 and –0.50 eÅ^{-3}. The least-squares refinement converged normally with residuals of R_1 = 0.0289 and GOF = 1.042. Crystal and refinement data for ($^{Ad}TPBN_3$)UF: formula $C_{54}H_{60}FN_3U$, space group $P2_1/c$, a = 10.9969(2) Å, b = 18.5897(4) Å, c = 21.2481(4) Å, β = 101.153(2)°, V = 4261.69(15) Å^3, Z = 4, μ = 3.855 mm^{-1}, F(000) = 2024.0, R_1 = 0.0386 and wR_2 = 0.0648 (based on all data).

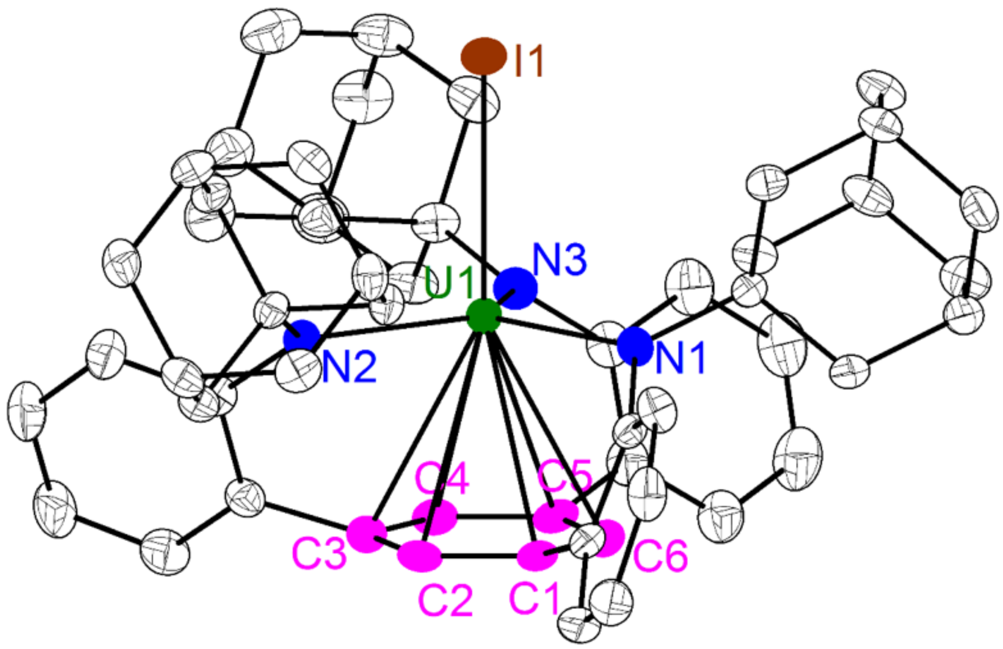

Supplementary Fig. 11. Representation of (^{Ad}TPBN$_3$)UI (**7**) with thermal ellipsoids set at 50% probability. Hydrogen atoms were omitted for clarity. Selected distances (Å) and angles (°): U1–I1 3.0846(5), U1–N1 2.2794(31), U1–N2 2.2893(32), U1–N3 2.3097(32), U1–C1 2.9775(39), U1–C2 2.9800(42), U1–C3 2.9696(42), U1–C4 2.9842(39), U1–C5 2.9802(40), U1–C6 2.9945(40), U1–C$_{centroid}$ 2.6318(4), U1–3N$_{plane}$ +0.1070(4), C1–C2 1.3840(56), C2–C3 1.4128(53), C3–C4 1.3883(58), C4–C5 1.4162(57), C5–C6 1.3860(54), C6–C1 1.4136(59); N1–U1–N2 119.499(110), N2–U1–N3 120.197(112), N3–U1–N1 119.656(113).

Single crystals suitable for X-ray crystallography were grown from an *n*-pentane solution. A total of 34203 reflections (–27 ≤ h ≤ 28, –9 ≤ k ≤ 14, –24 ≤ l ≤ 26) were collected at T = 180.15 K with $2\theta_{max}$ = 58.706°, of which 10932 were unique. The residual peak and hole electron density were 1.55 and –1.76 eÅ^{-3}. The least-squares refinement converged normally with residuals of R_1 = 0.0353 and GOF = 1.026. Crystal and refinement data for (^{Ad}TPBN$_3$)UI: formula $C_{54}H_{60}IN_3U$, space group $P2_1/c$, a = 20.7007(5) Å, b = 10.8830(3) Å, c = 19.4004(5) Å, β = 92.280(2)°, V = 4367.2(2) Å^3, Z = 4, μ = 4.465 mm^{-1}, F(000) = 2200.0, R_1 = 0.0498 and wR_2 = 0.0665 (based on all data).

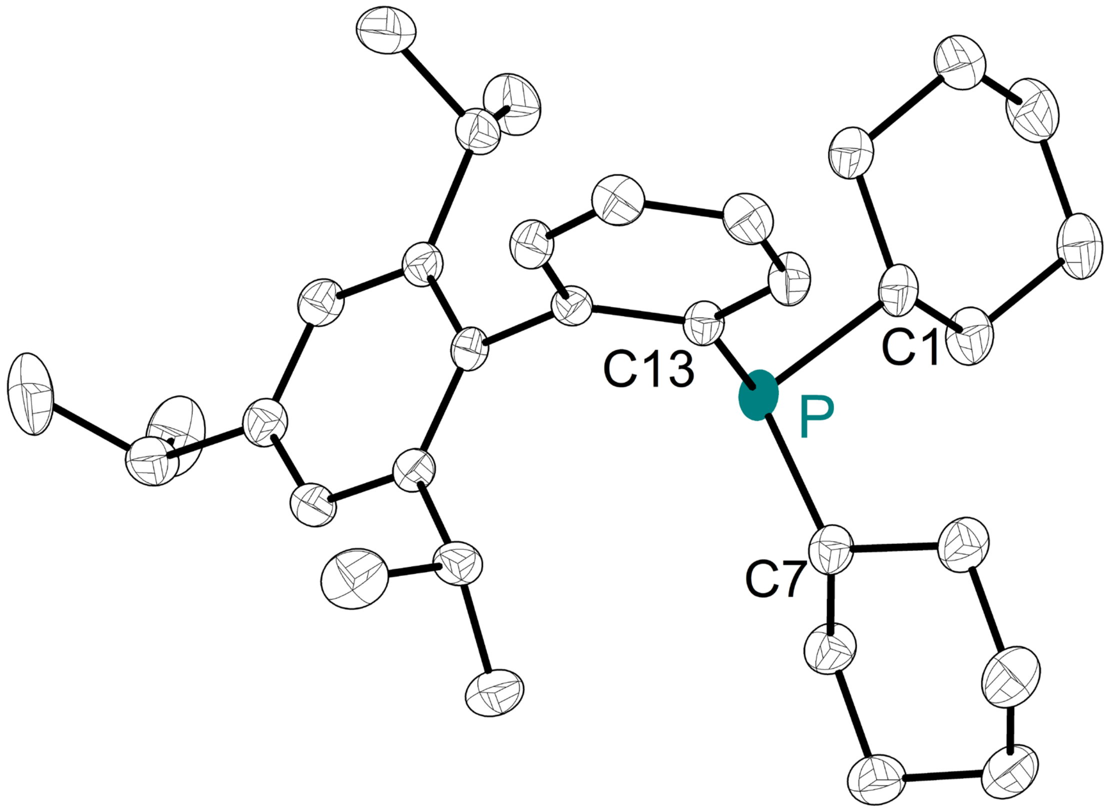

Supplementary Fig. 12. Representation of XPhos (2-dicyclohexylphosphino-2′,4′,6′-triisopropylbiphenyl, CAS No. 564483-18-7) with thermal ellipsoids set at 50% probability. Hydrogen atoms were omitted for clarity. Selected distances (Å) and angles (°): P1–C1 1.8664(10), P1–C7 1.8819(12), P1–C13 1.8481(12); C1–P1–C7 104.623(50), C7–P1–C13 101.938(48), C13–P1–C1 100.333(49); dihedral angle between the two phenyl rings 89.157(33).

Single crystals suitable for X-ray crystallography were grown from *n*-hexane washing (during the tripodal ligand synthesis process) at –20 °C. A total of 34637 reflections ($-14 \leq h \leq 14$, $-26 \leq k \leq 24$, $-17 \leq l \leq 20$) were collected at T = 180.00(10) K with $2\theta_{max}$ = 60.792°, of which 7725 were unique. The residual peak and hole electron density were 0.34 and –0.18 eÅ^{-3}. The least-squares refinement converged normally with residuals of R_1 = 0.0401 and GOF = 1.031. Crystal and refinement data for XPhos: formula $C_{33}H_{49}P$, space group $P2_1/c$, a = 10.9278(3) Å, b = 18.6308(6) Å, c = 14.7813(5) Å, β = 104.640(3)°, V = 2911.67(16) Å^3, Z = 4, μ = 0.113 mm^{-1}, F(000) = 1048.0, R_1 = 0.0488 and wR_2 = 0.1061 (based on all data).

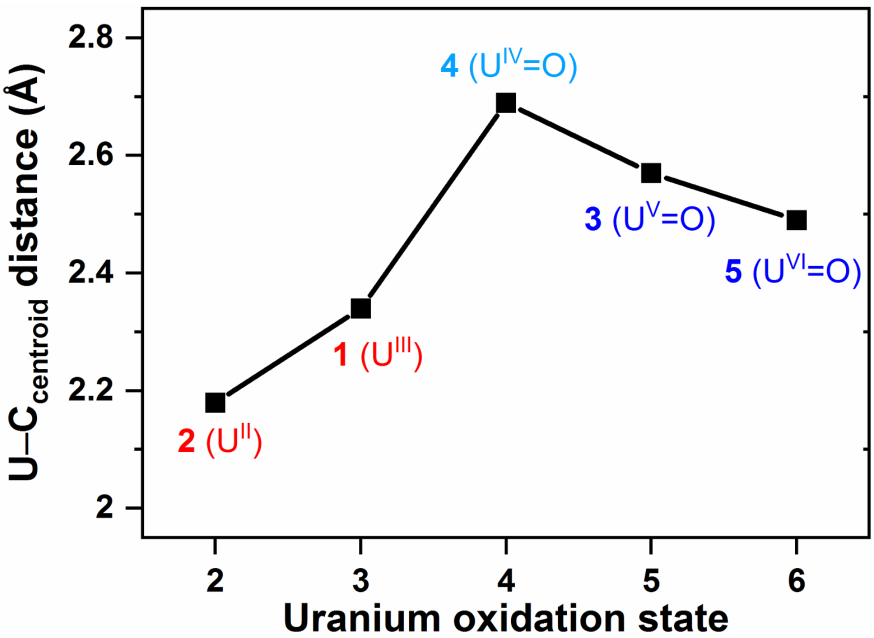

Supplementary Fig. 13. The plot of U–C$_{centroid}$ distances versus oxidation states of uranium for **1**–**5**.

Supplementary Table 1. Selected metrical parameters of **1**–**7**.

	**2**[a]	**1**	**6/7**	**4**	**3**	**5**
U oxidation states	II	III	IV	IV	V	VI
avg. U–N	2.477(3)	2.423(2)	2.323(2)/2.293(3)	2.421(4)	2.317(3)	2.274(3)
U–$C_{centroid}$	2.18	2.34	2.53/2.63	2.69	2.57	2.49
avg. C–C_{arene}	1.417(6)	1.408(5)	1.400(5)/1.400(6)	1.399(6)	1.398(4)	1.403(6)
avg. U–C_{arene}	2.596(4)	2.730(3)	2.893(3)/2.981(4)	3.028(4)	2.922(3)	2.860(4)
U–$3N_{plane}$[b]	–0.34	–0.17	0.07/0.11	0.25	0.12	0.04
U–X[c]	NA	NA	2.090(2)/3.085(1)	1.874(4)	1.829(2)	1.818(2)

Note: All distances in Å. [a] The average of two crystallographically independent molecules in an asymmetric unit; [b] U–$3N_{plane}$ is defined as the distance of the uranium ion to the plane of the three nitrogen atoms (positive values for above the plane and negative values for below the plane); [c] X = O for **3**–**5**, X = F for **6**, and X = I for **7**.

3. IR Spectra

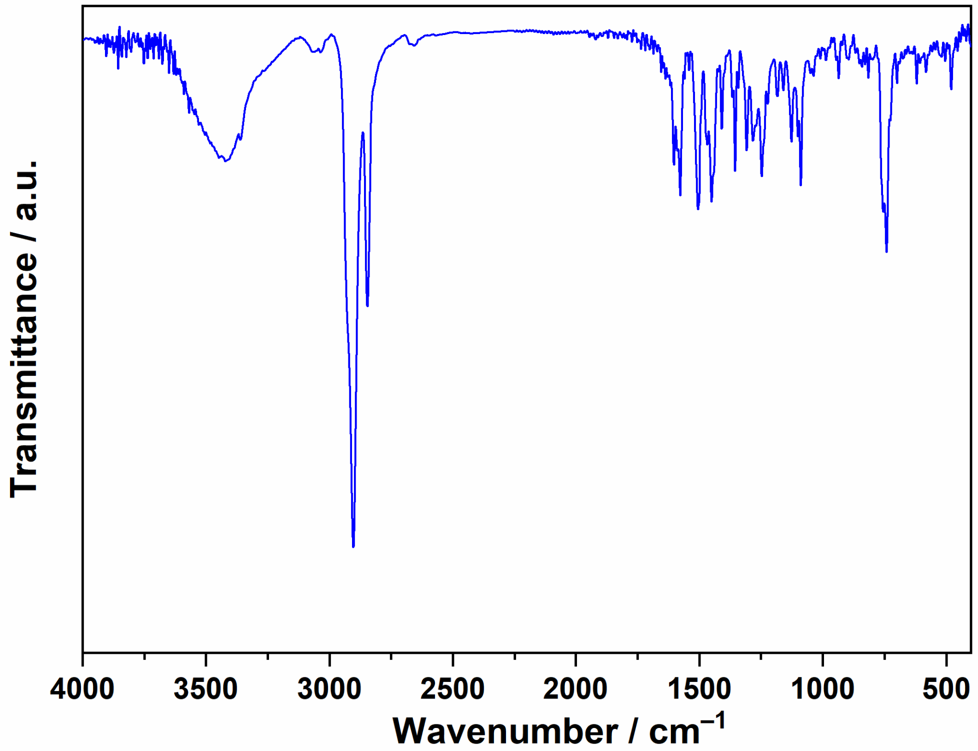

Supplementary Fig. 14. IR spectrum of H_3(^{Ad}TPBN_3) in KBr, $\tilde{\nu}$ / cm^{-1}: 481 (w), 743 (m), 1089 (m), 1127 (w), 1184 (w), 1249 (w), 1284 (w), 1309 (w), 1355 (w), 1409 (w), 1450 (m), 1507 (m), 1578 (m), 1603 (w), 2848 (m), 2904 (s), 3422 (m).

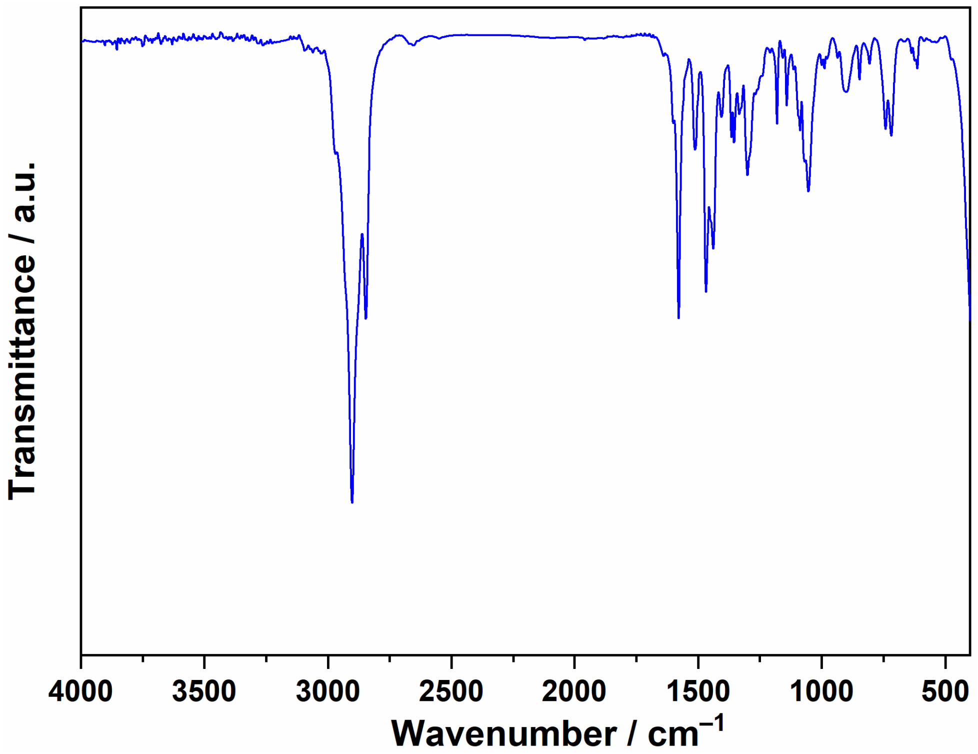

Supplementary Fig. 15. IR spectrum of $K_3(^{Ad}TPBN_3)$ in KBr, $\tilde{\nu}$ / cm^{-1}: 613 (w), 720 (w), 743 (w), 847 (w), 900 (w), 1055 (m), 1088 (w), 1142 (w), 1181 (w), 1301 (m), 1334 (w), 1355 (w), 1366 (w), 1406 (w), 1439 (m), 1468 (m), 1514 (w), 1579 (m), 2848 (m), 2903 (s).

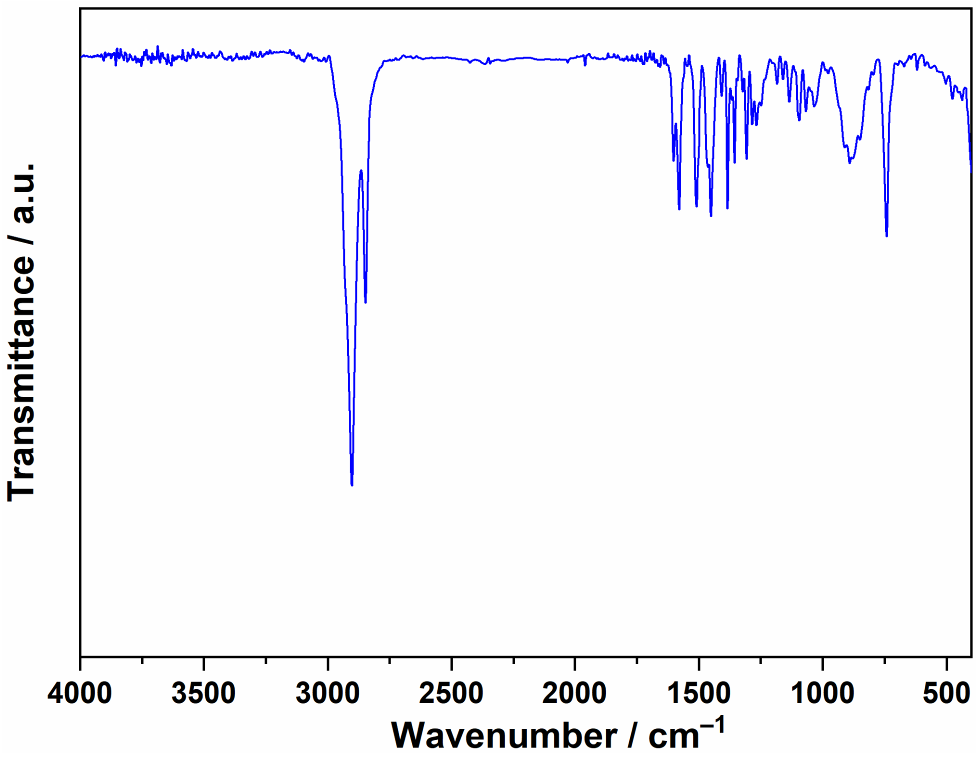

Supplementary Fig. 16. IR spectrum of (^{Ad}TPBN$_3$)U (**1**) in KBr, $\tilde{\nu}$ / cm^{-1}: 743 (m), 891 (m), 1035 (w), 1068 (w), 1095 (w), 1135 (w), 1268 (w), 1286 (w), 1308 (w), 1356 (w), 1384 (m), 1408 (w), 1451 (m), 1511 (m), 1580 (m), 1602 (w), 2849 (m), 2904 (s).

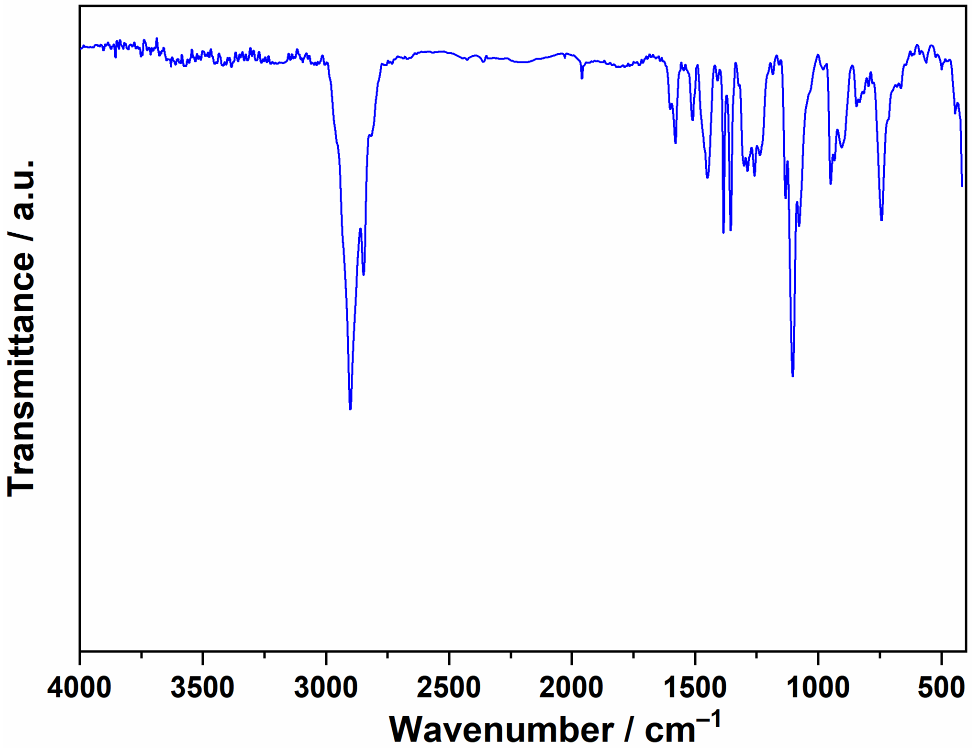

Supplementary Fig. 17. IR spectrum of [K(crypt)][(^{Ad}TPBN$_3$)U] (**2**) in KBr, $\tilde{\nu}$ / cm^{-1}: 743 (m), 845 (w), 902 (m), 948 (m), 1078 (m), 1104 (s), 1133 (m), 1260 (m), 1288 (m), 1355 (m), 1384 (m), 1449 (m), 1511 (w), 1580 (w), 2849 (m), 2902 (s).

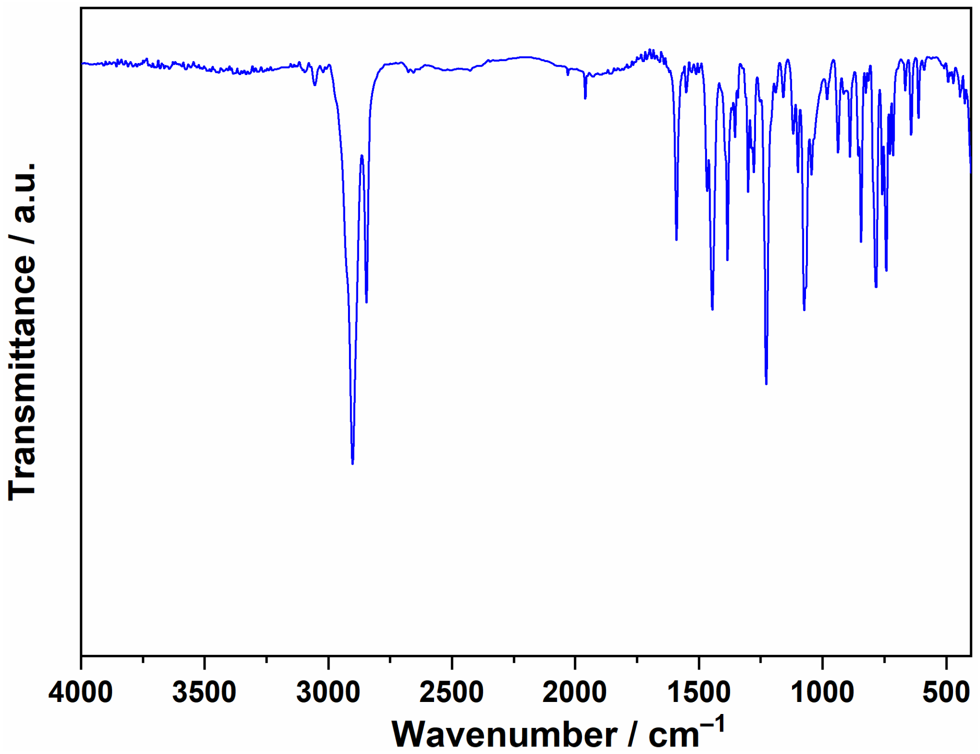

Supplementary Fig. 18. IR spectrum of (^{Ad}TPBN$_3$)UO (**3**) in KBr, $\tilde{\nu}$ / cm^{-1}: 612 (w), 642 (w), 716 (w), 728 (w), 743 (m), 759 (w), 783 (m), 844 (m), 889 (w), 937 (w), 1045 (w), 1075 (m), 1100 (w), 1119 (w), 1227 (s), 1279 (w), 1302 (w), 1354 (w), 1384 (m), 1445 (m), 1466 (w), 1591 (m), 2847 (m), 2903 (s).

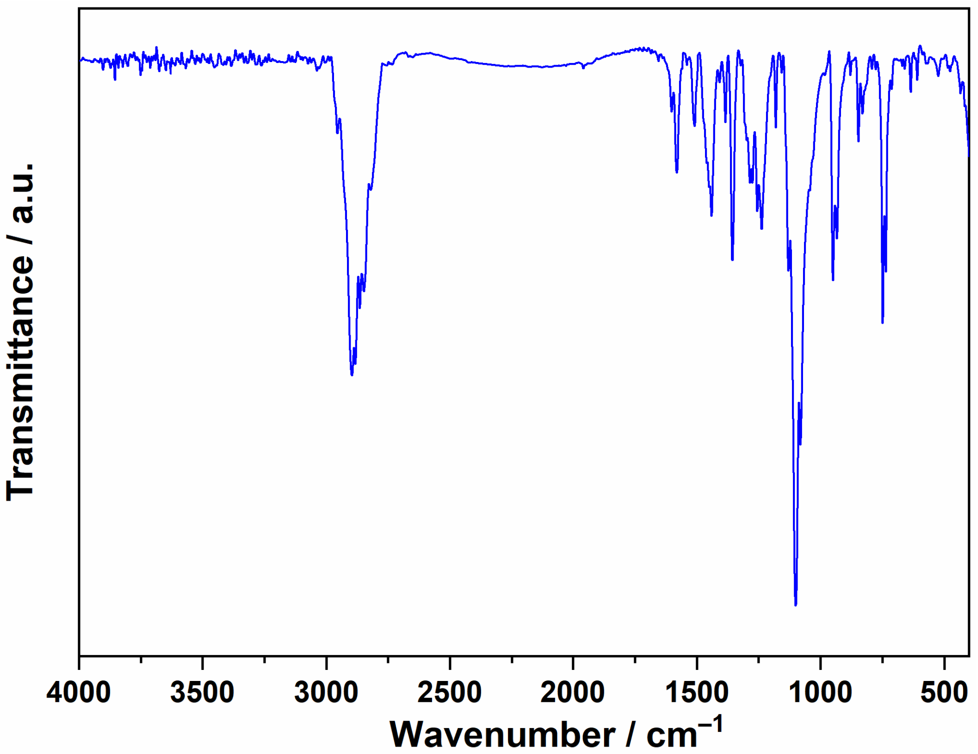

Supplementary Fig. 19. IR spectrum of [K(crypt)][(^{Ad}TPBN$_3$)UO] (**4**) in KBr, $\tilde{\nu}$ / cm^{-1}: 635 (w), 737 (m), 750 (m), 831 (w), 847 (w), 933 (m), 949 (m), 1082 (s), 1101 (s), 1130 (m), 1181 (w), 1239 (m), 1257 (m), 1276 (m), 1286 (m), 1357 (m), 1385 (w), 1440 (m), 1510 (w), 1582 (m), 1602 (w), 2849 (m), 2866 (m), 2883 (s), 2897 (s), 2956 (w).

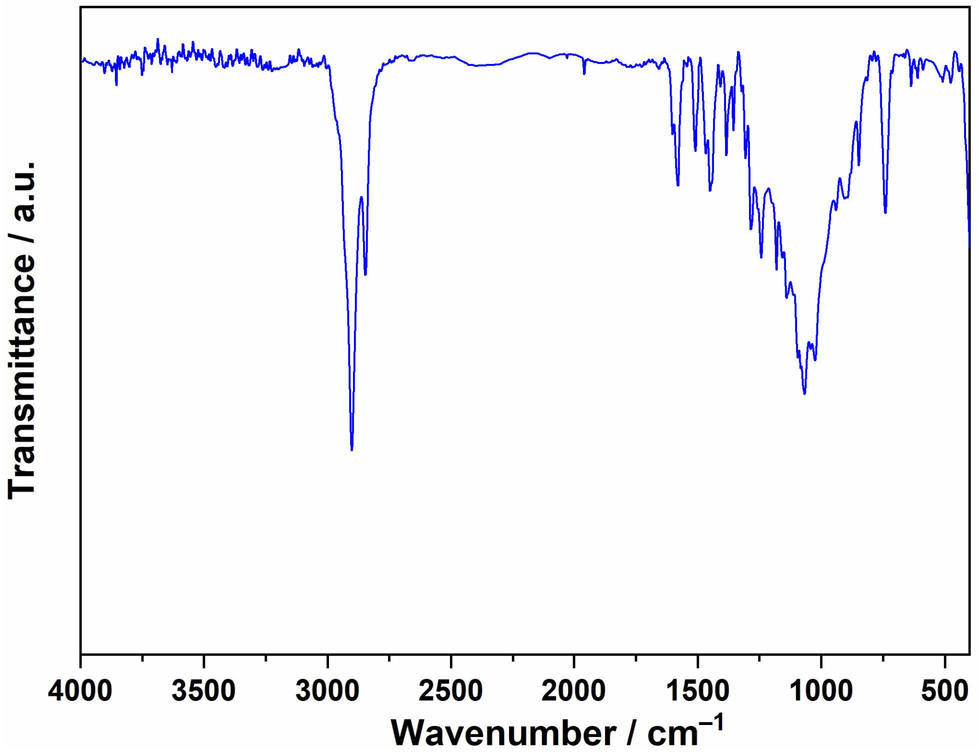

Supplementary Fig. 20. IR spectrum of [Cp*$_2$Co][(^{Ad}TPBN$_3$)UO] (**4′**) in KBr, $\tilde{v}$ / cm^{-1}: 636 (w), 743 (m), 848 (w), 904 (m), 940 (m), 1026 (s), 1068 (s), 1140 (m), 1181 (m), 1244 (m), 1286 (m), 1307 (w), 1356 (w), 1384 (w), 1408 (w), 1450 (m), 1467 (w), 1510 (w), 1580 (m), 1602 (w), 2848 (m), 2903 (s).

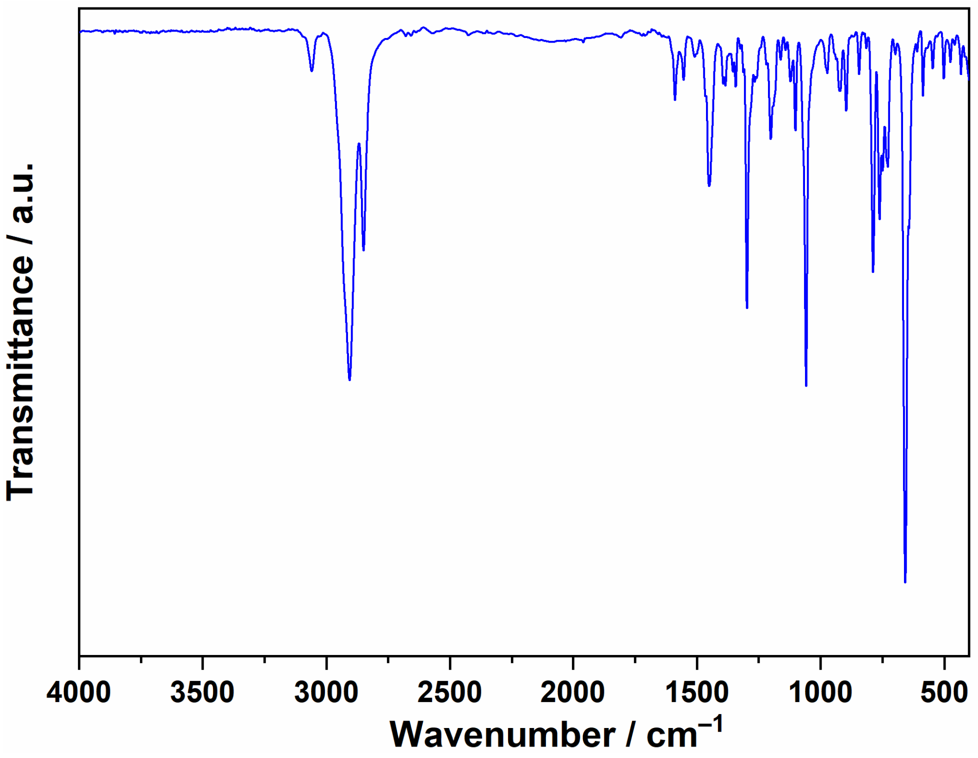

Supplementary Fig. 21. IR spectrum of [(^{Ad}TPBN$_3$)UO][SbF$_6$] (**5**) in KBr, $\tilde{\nu}$ / cm^{-1}: 433 (w), 503 (w), 547 (w), 586 (w), 657 (vs, SbF$_6$), 730 (m), 762 (m), 789 (m), 844 (w), 896 (w), 921 (w), 974 (w), 1059 (s), 1102 (m), 1122 (w), 1201 (m), 1299 (s), 1344 (w), 1385 (w), 1450 (m), 1555 (w), 1589 (w), 2851 (m), 2907 (s), 3059 (w).

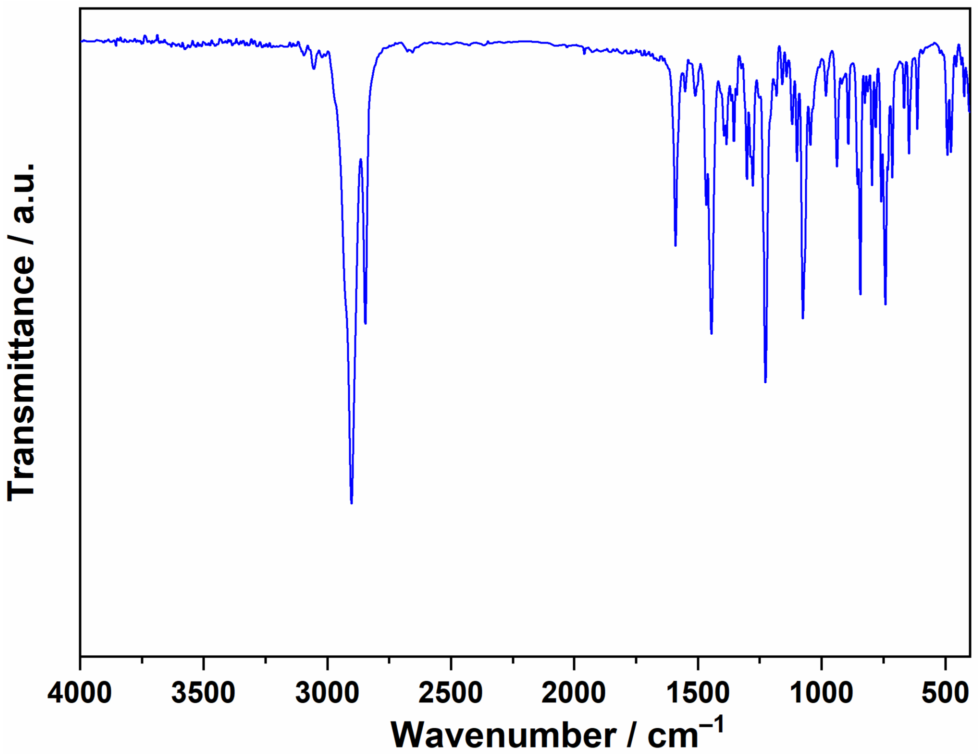

Supplementary Fig. 22. IR spectrum of (^{Ad}TPBN$_3$)UF (**6**) in KBr, $\tilde{\nu}$ / cm^{-1}: 478 (w), 492 (w), 613 (w), 646 (w), 666 (w), 715 (w), 743 (m), 760 (w), 782 (w), 797 (w), 825 (w), 844 (m), 892 (w), 937 (w), 983 (w), 1046 (w), 1076 (m), 1100 (w), 1119 (w), 1182 (w), 1226 (m), 1279 (w), 1303 (w), 1355 (w), 1385 (w), 1445 (m), 1466 (w), 1511 (w), 1591 (m), 2848 (m), 2903 (s).

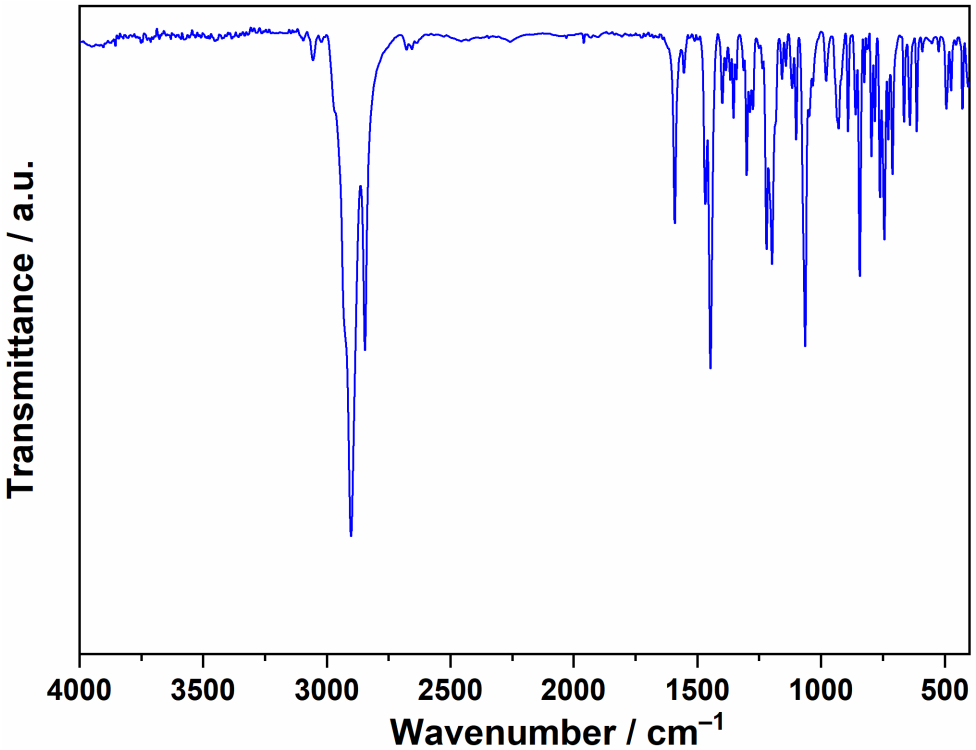

Supplementary Fig. 23. IR spectrum of (^{Ad}TPBN$_3$)UI (**7**) in KBr, $\tilde{\nu}$ / cm^{-1}: 427 (w), 475 (w), 494 (w), 613 (w), 641 (w), 664 (w), 712 (w), 729 (w), 745 (w), 763 (m), 783 (w), 797 (w), 844 (m), 860 (w), 891 (w), 929 (w), 1065 (m), 1101 (w), 1198 (m), 1220 (m), 1276 (w), 1289 (w), 1301 (w), 1354 (w), 1399 (w), 1447 (m), 1467 (m), 1592 (m), 2487 (m), 2903 (s).

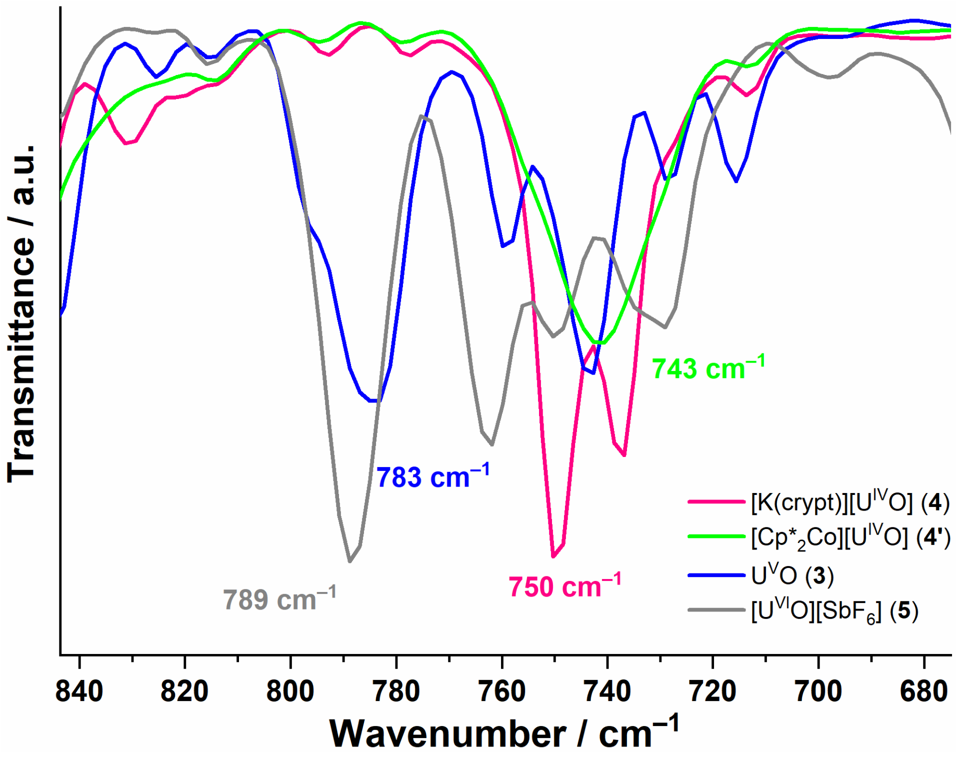

Supplementary Fig. 24. IR spectra (840–680 cm^{-1} region) of the uranium(IV–VI) oxo complexes [K(crypt)][(^{Ad}TPBN$_3$)UO] (**4**), [Cp$^*_2$Co][(^{Ad}TPBN$_3$)UO] (**4′**), (^{Ad}TPBN$_3$)UO (**3**), and [(^{Ad}TPBN$_3$)UO][SbF$_6$] (**5**) in KBr. The peaks ($\tilde{\nu}$ in cm^{-1}) tentatively assigned to the U–O stretching are labeled in different colours (**4**, pink; **4′**, green; **3**, blue; **5**, gray). The trend of wavenumbers for U–O stretching is consistent with the strength of U–O bond as the oxidation state of uranium increases from +4 to +6.

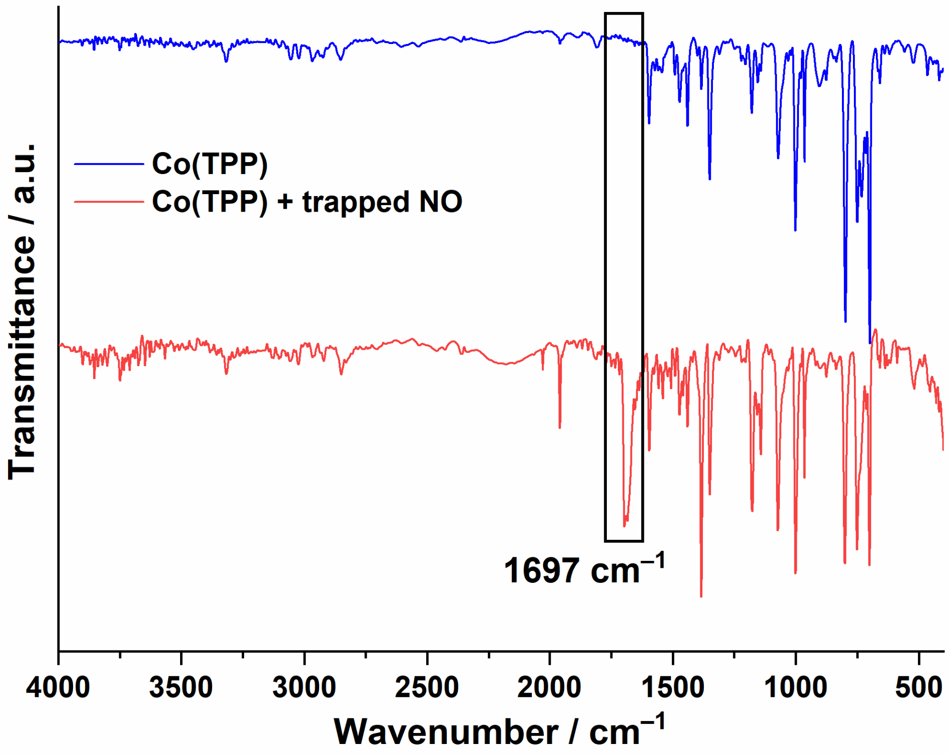

Supplementary Fig. 25. IR spectra of Co(TPP) (blue), and Co(TPP)(NO) (red) formed in the trapping experiment (see Supplementary section 1.3 for details). The diagnostic N–O stretching peak of Co(TPP)(NO) is highlighted at $\tilde{\nu}$(NO) = 1697 cm^{-1}.

4. Electrochemical Measurement

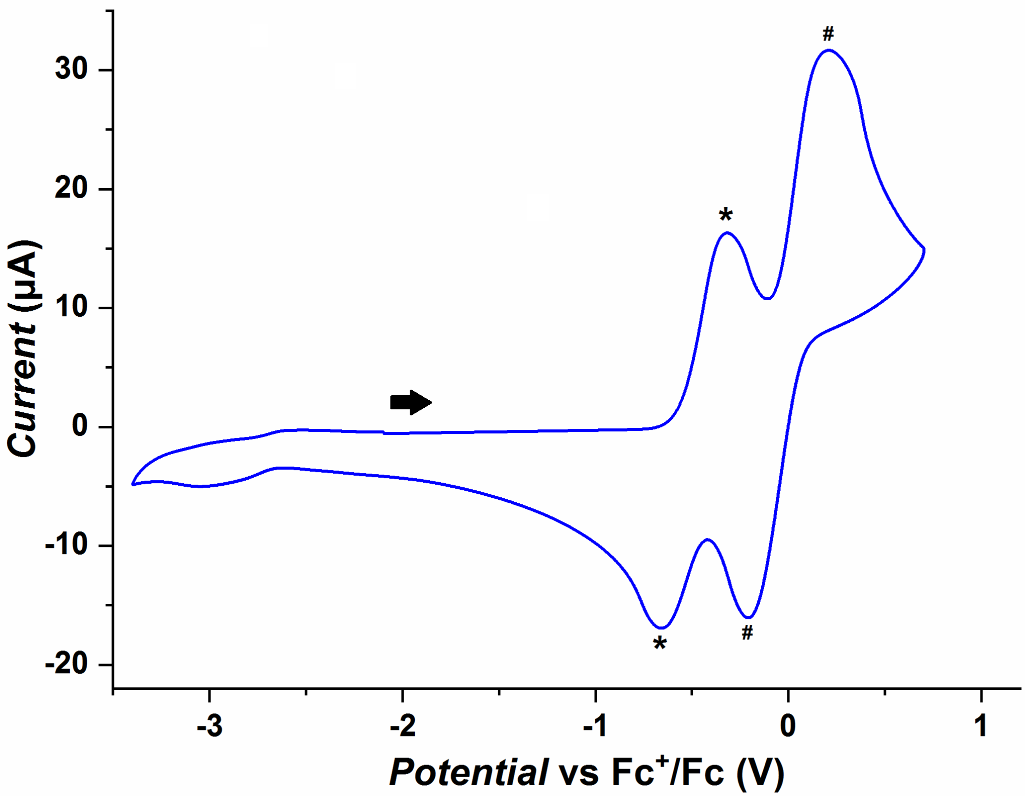

Supplementary Fig. 26. Cyclic voltammogram of Fc^* (1 mM, marked with asterisk) and Fc (Cp_2Fe, 1 mM, marked with "#") in the experimental cell at v = 50 mV/s in a THF solution of [TBA][PF_6] (0.1 mol/L). Fc^{*+}/Fc^* was determined to have a potential of –0.488 V vs Fc^+/Fc under these conditions.

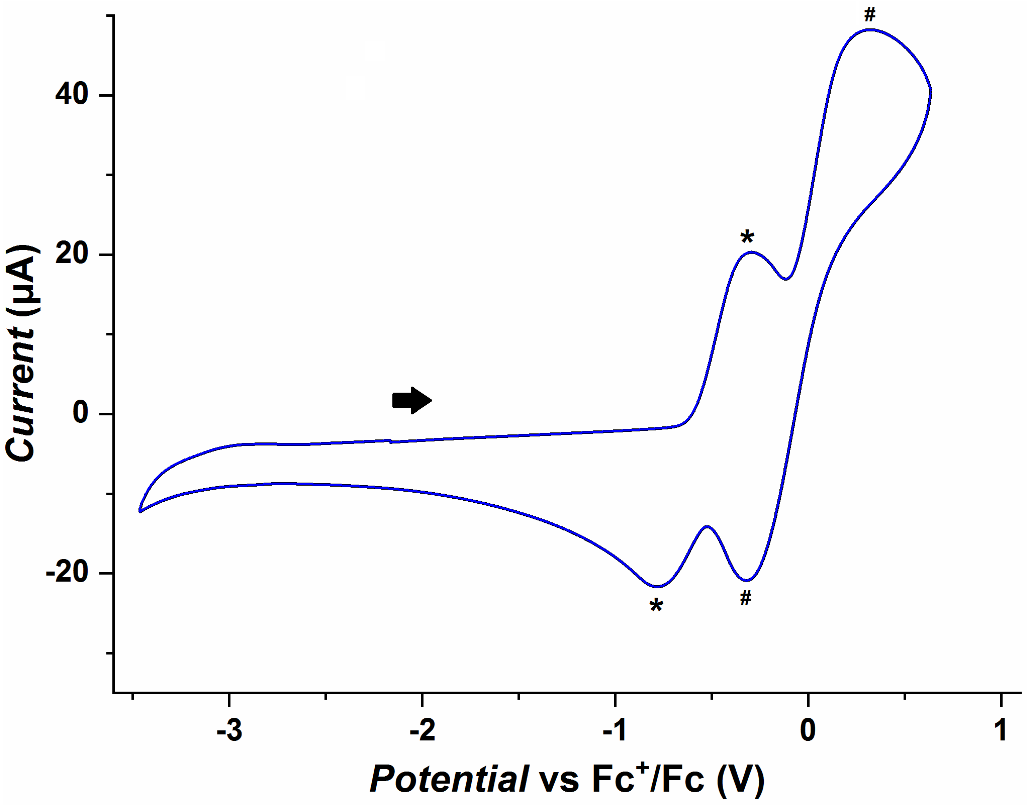

Supplementary Fig. 27. Cyclic voltammogram of Fc* (1 mM, marked with asterisk) and Fc (1 mM, marked with "#") in the experimental cell at v = 200 mV/s in a THF solution of [TBA][PF_6] (0.1 mol/L). Fc^{*+}/Fc^{*} was determined to have a potential of –0.538 V vs Fc^{+}/Fc under these conditions.

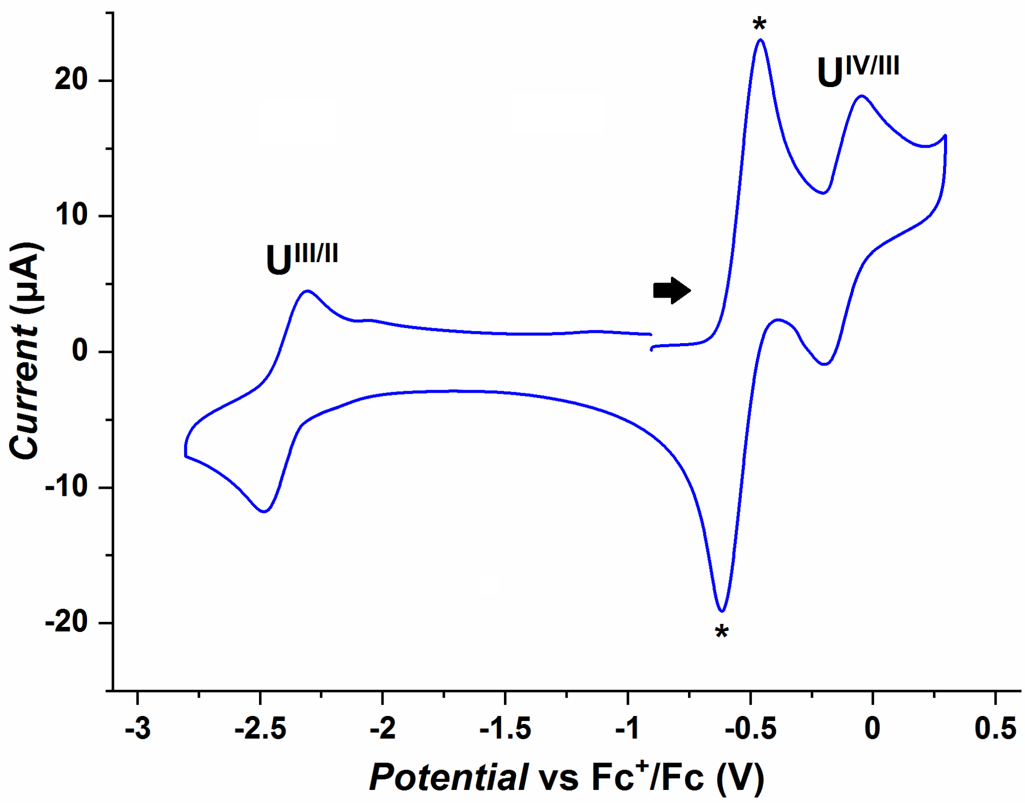

Supplementary Fig. 28. Cyclic voltammogram of (^{Ad}TPBN$_3$)U (**1**) (1 mM) recorded scanning anodically at v = 200 mV/s in a THF solution of [TBA][PF$_6$] (0.1 M). The event marked with asterisk is due to the internal standard Fc* (1 mM). Two reversible events are centered at –2.396 V (E_{pa}: –2.308 V, E_{pc}: –2.484 V) and –0.122 V (E_{pa}: –0.045 V, E_{pc}: –0.200 V), and assigned for the redox pairs of U^{III}/U^{II} and U^{IV}/U^{III}, respectively.

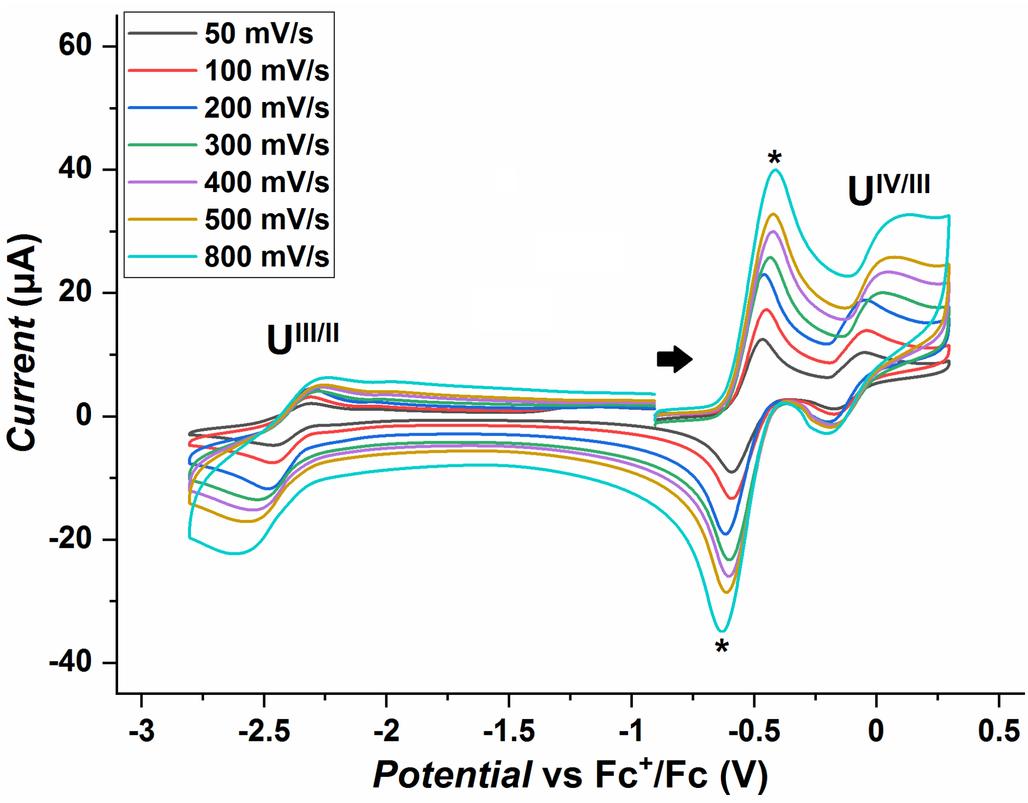

Supplementary Fig. 29. Cyclic voltammogram of (^{Ad}TPBN$_3$)U (**1**) (1 mM) recorded scanning anodically at v = 50, 100, 200, 300, 400, 500, 800 mV/s in a THF solution of [TBA][PF_6] (0.1 M). The event marked with asterisk is due to the internal standard Fc* (1 mM). The two uranium-centered redox events are identified and labelled accordingly.

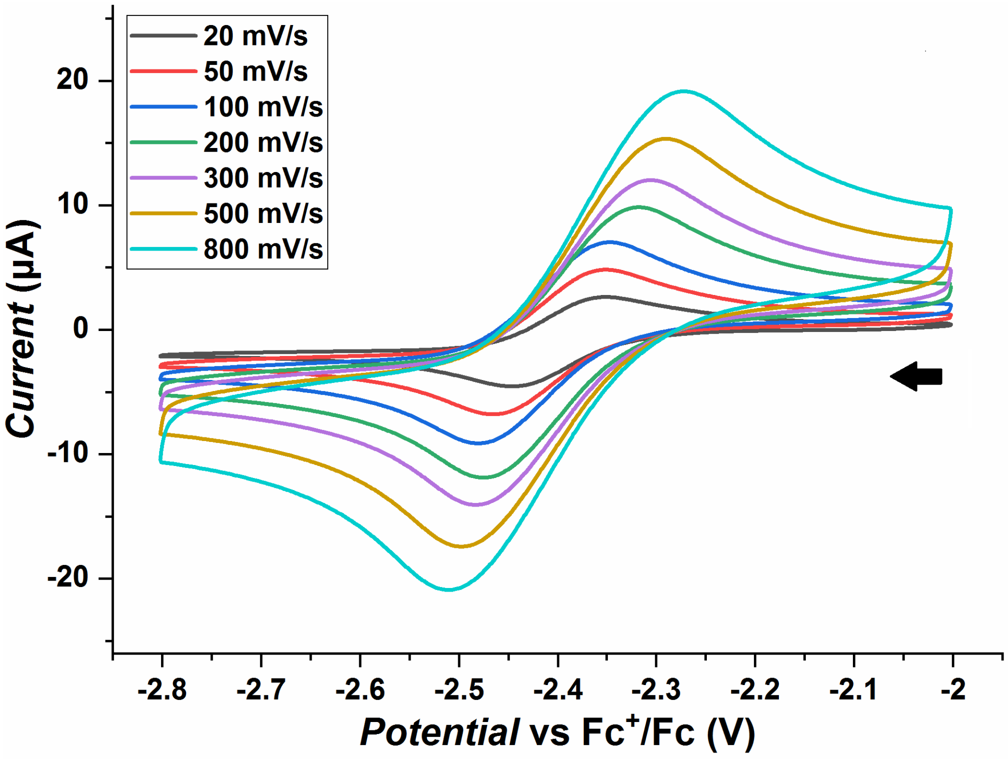

Supplementary Fig. 30. Cyclic voltammogram of the redox event at $E_{1/2}$ = –2.396 V vs Fc^+/Fc of ($^{Ad}TPBN_3$)U (**1**) (1 mM) recorded scanning cathodically at different scan rates in a THF solution of [TBA][PF_6] (0.1 M).

Supplementary Table 2. Electrochemical parameters of the reduction of **1** (Supplementary Fig. 30).

Scan rate (V/s)	E_{pa} (V)*	E_{pc} (V)*	$E_{1/2}$ (V)*	ΔE_p (V)*	i_{pa} (μA)	i_{pc} (μA)	$\|i_{pa}/i_{pc}\|$
0.020	–2.351	–2.447	–2.399	–0.096	4.09	–4.31	0.95
0.050	–2.351	–2.466	–2.4085	–0.115	6.36	–6.56	0.97
0.100	–2.348	–2.481	–2.4145	–0.133	8.67	–8.84	0.98
0.200	–2.318	–2.474	–2.396	–0.156	11.36	–11.47	0.99
0.300	–2.306	–2.483	–2.3945	–0.177	13.42	–13.42	1.00
0.500	–2.290	–2.498	–2.394	–0.208	16.40	–16.24	1.01
0.800	–2.272	–2.510	–2.391	–0.238	19.40	–18.95	1.02

* All referenced to the Fc^+/Fc standard.

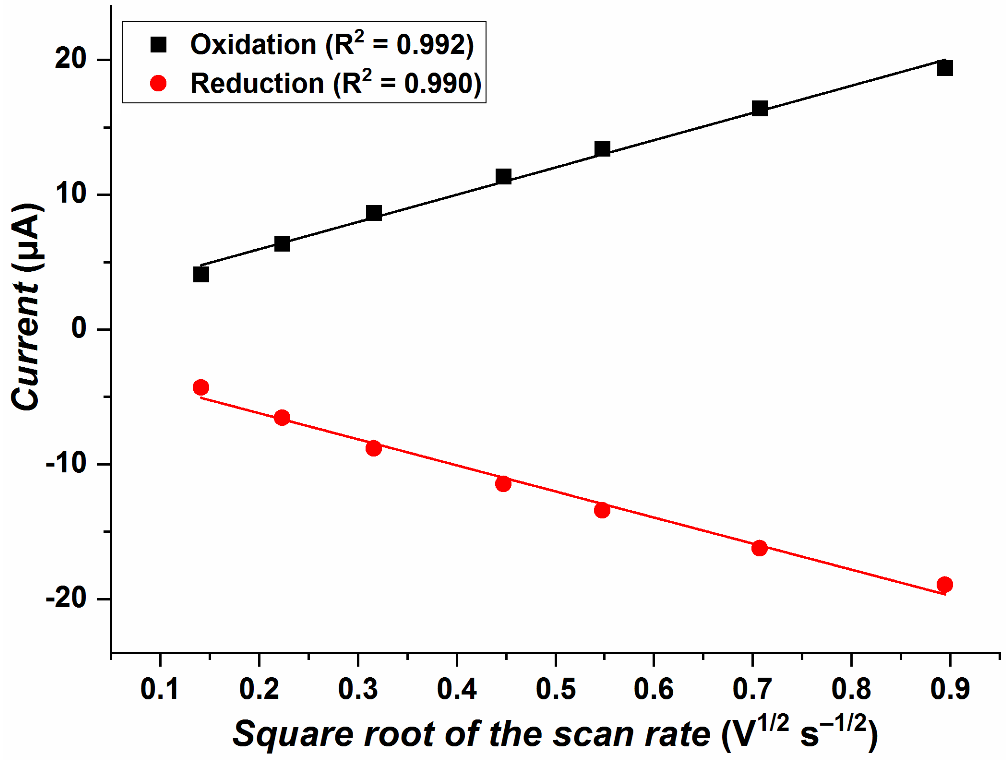

Supplementary Fig. 31. The Randles-Ševčík plots of the positive peak currents (i_{pa}) and negative peak currents (i_{pc}) of the redox event at $E_{1/2}$ = –2.396 V vs Fc^+/Fc of **1** (Supplementary Fig. 30).

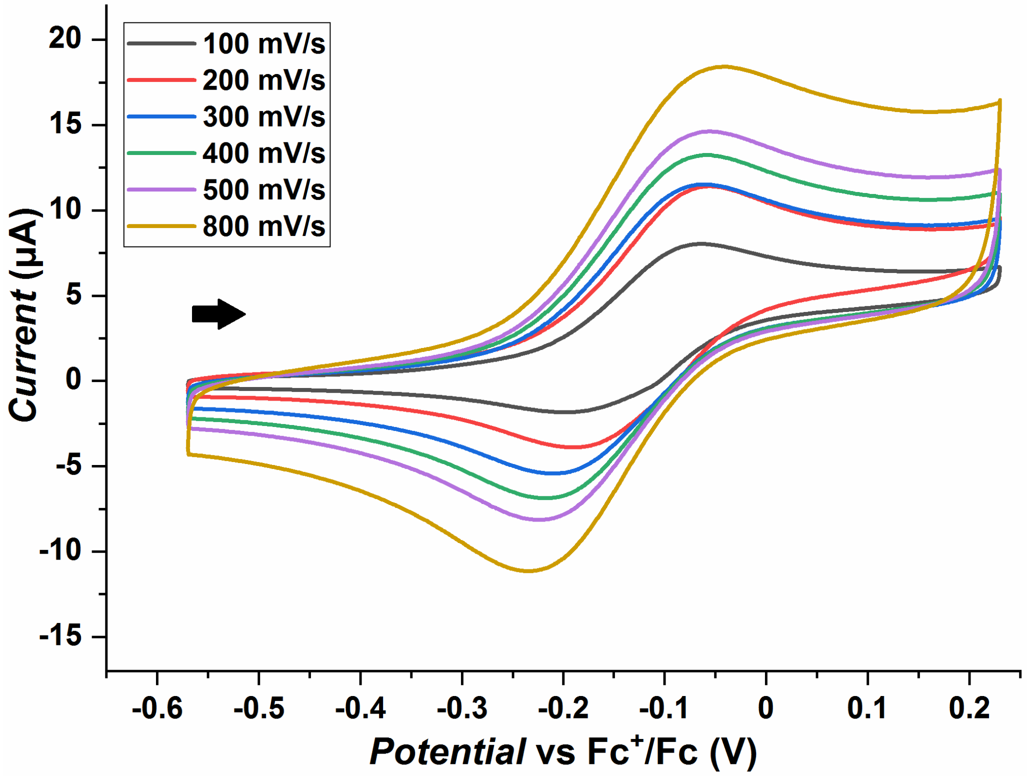

Supplementary Fig. 32. Cyclic voltammogram of the redox event at $E_{1/2}$ = –0.122 V vs Fc^+/Fc of (^{Ad}TPBN$_3$)U (**1**) (1 mM) recorded scanning anodically at different scan rates in a THF solution of [TBA][PF_6] (0.1 M).

Supplementary Table 3. Electrochemical parameters of the oxidation of **1** (Supplementary Fig. 32).

Scan rate (V/s)	E_{pa} (V)*	E_{pc} (V)*	$E_{1/2}$ (V)*	ΔE_p (V)*	i_{pa} (μA)	i_{pc} (μA)	$\lvert i_{pa}/i_{pc}\rvert$
0.100	–0.063	–0.198	–0.1305	–0.135	7.03	–4.46	1.58
0.200	–0.055	–0.189	–0.122	–0.134	8.72	–6.54	1.33
0.300	–0.062	–0.211	–0.1365	–0.149	9.52	–7.20	1.32
0.400	–0.059	–0.220	–0.1395	–0.161	10.83	–8.33	1.30
0.500	–0.055	–0.223	–0.139	–0.168	11.70	–9.24	1.27
0.800	–0.0405	–0.2335	–0.137	–0.193	13.88	–11.15	1.24

* All referenced to the Fc^+/Fc standard.

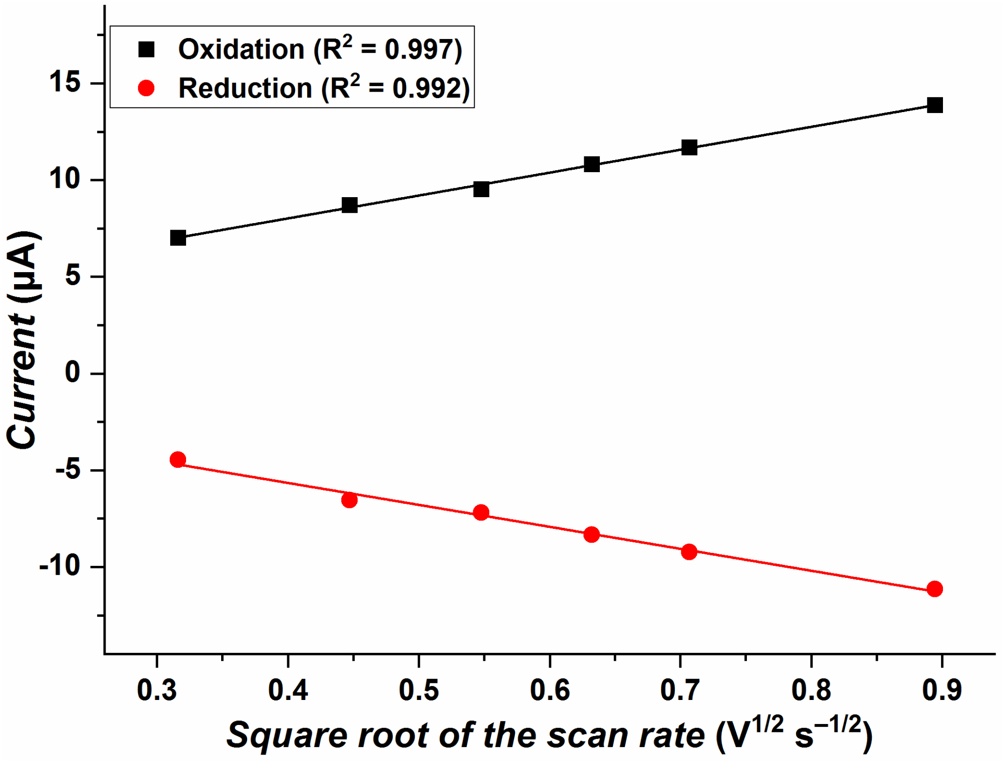

Supplementary Fig. 33. The Randles-Ševčík plots of the positive peak currents (i_{pa}) and negative peak currents (i_{pc}) of the redox event at $E_{1/2}$ = –0.122 V vs. Fc^+/Fc of **1** (Supplementary Fig. 32).

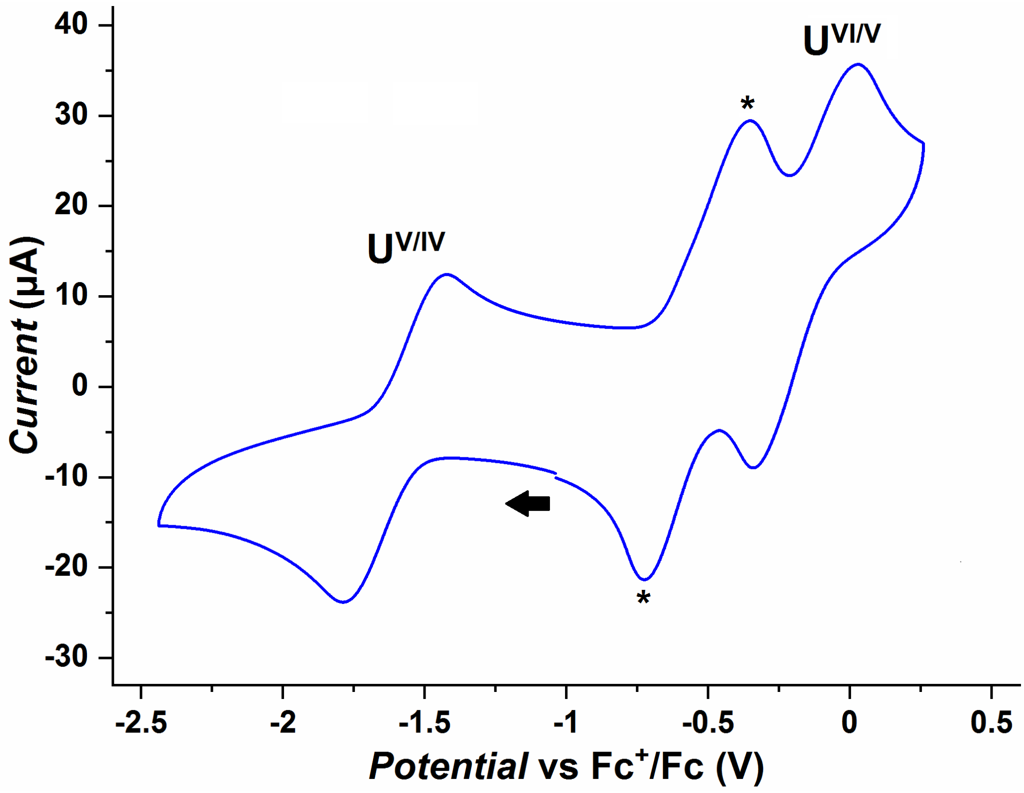

Supplementary Fig. 34. Cyclic voltammogram of (^{Ad}TPBN$_3$)UO (**3**) (2 mM) recorded scanning cathodically at v = 200 mV/s in a THF solution of [TBA][PF$_6$] (0.1 M). The event marked with asterisk is due to the internal standard Fc* (1 mM). Two other events are centered at −1.604 V (E_{pa}: –1.422 V, E_{pc}: –1.788 V) and −0.155 V (E_{pa}: 0.030 V, E_{pc}: –0.340 V), and assigned for the redox pairs of [U^{V}O]/[U^{IV}O] and [U^{VI}O]/[U^{V}O], respectively.

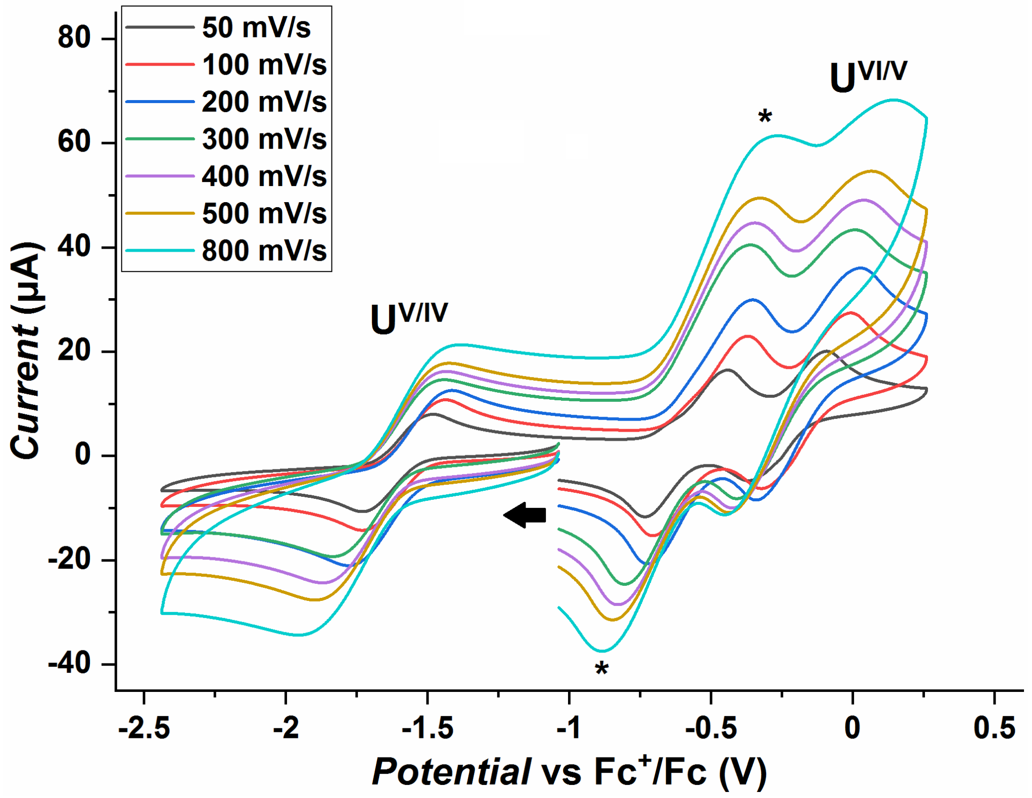

Supplementary Fig. 35. Cyclic voltammogram of (^{Ad}TPBN$_3$)UO (**3**) (2 mM) recorded scanning cathodically at v = 50, 100, 200, 300, 400, 500, 800 mV/s in a THF solution of [TBA][PF$_6$] (0.1 M). The event marked with asterisk is due to the internal standard Fc* (1 mM). The two uranium-centered redox events are identified and labelled accordingly.

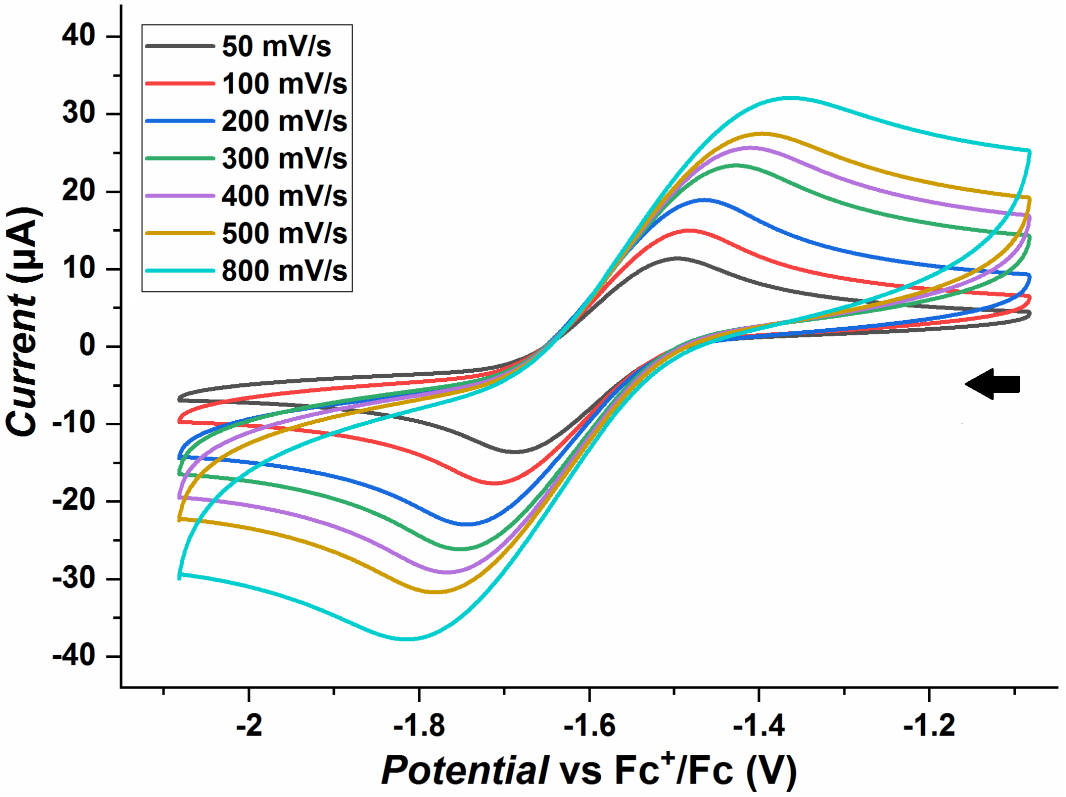

Supplementary Fig. 36. Cyclic voltammogram of the redox event at $E_{1/2}$ = –1.604 V vs Fc^+/Fc of ($^{Ad}TPBN_3$)UO (**3**) (2 mM) recorded scanning cathodically at different scan rates in a THF solution of [TBA][PF_6] (0.1 M).

Supplementary Table 4. Electrochemical parameters of the reduction of **3** (Supplementary Fig. 36).

Scan rate (V/s)	E_{pa} (V)*	E_{pc} (V)*	$E_{1/2}$ (V)*	ΔE_p (V)*	i_{pa} (μA)	i_{pc} (μA)	$\lvert i_{pa}/i_{pc}\rvert$
0.050	–1.496	–1.687	–1.5915	–0.191	13.27	–13.50	0.98
0.100	–1.482	–1.712	–1.597	–0.230	16.87	–17.03	0.99
0.200	–1.464	–1.744	–1.604	–0.280	20.51	–20.99	0.98
0.300	–1.427	–1.752	–1.5895	–0.325	23.15	–23.61	0.98
0.400	–1.411	–1.767	–1.589	–0.356	24.71	–25.29	0.98
0.500	–1.397	–1.780	–1.5885	–0.383	25.71	–26.61	0.97
0.800	–1.363	–1.815	–1.589	–0.452	27.67	–29.10	0.95

* All referenced to the Fc^+/Fc standard.

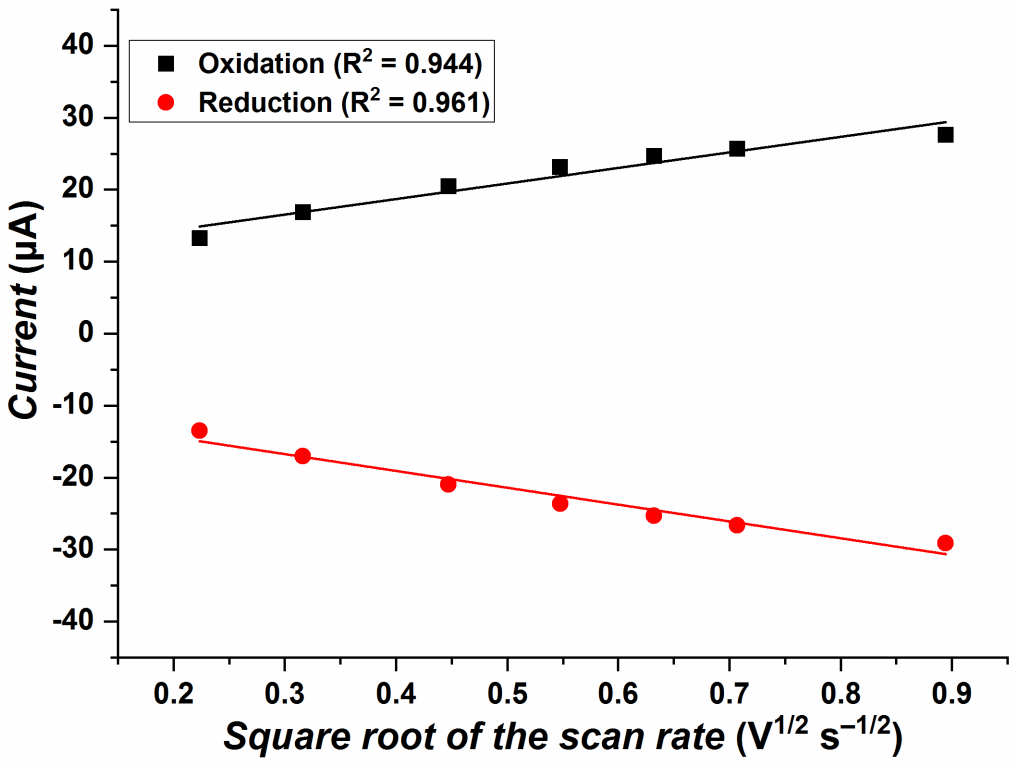

Supplementary Fig. 37. The Randles-Ševčík plots of the positive peak currents (i_{pa}) and negative peak currents (i_{pc}) of the redox event at $E_{1/2}$ = –1.604 V vs Fc^+/Fc of **3** (Supplementary Fig. 36).

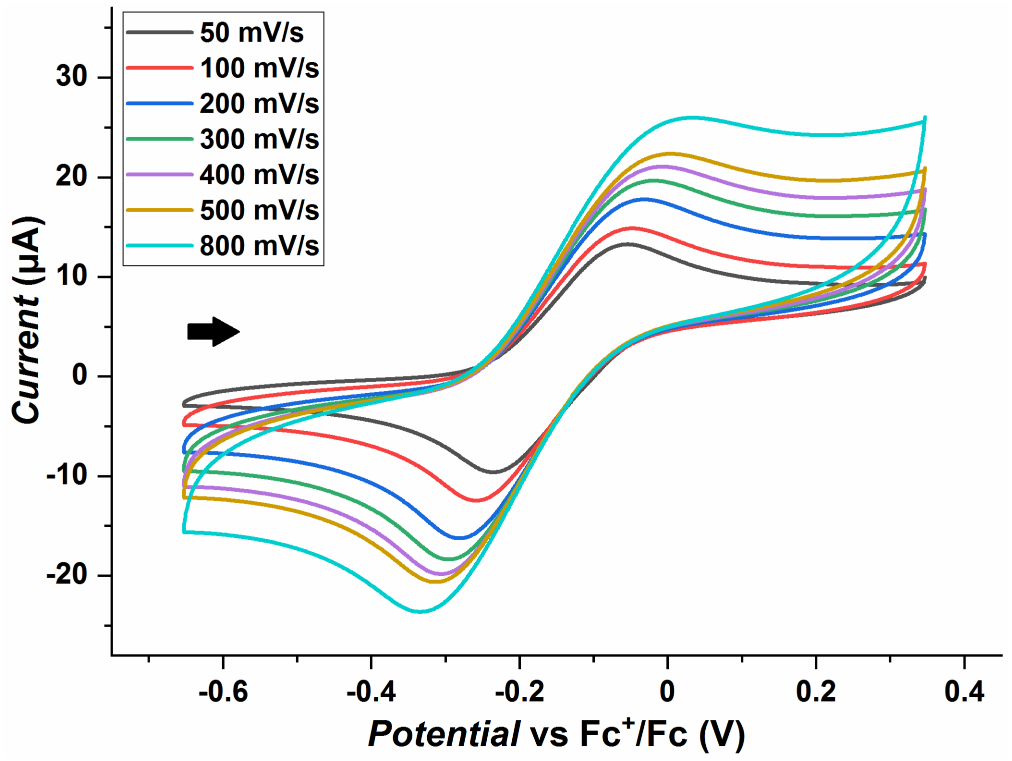

Supplementary Fig. 38. Cyclic voltammogram of the redox event at $E_{1/2}$ = –0.155 V vs Fc^+/Fc of (^{Ad}TPBN$_3$)UO (**3**) (2 mM) recorded scanning anodically at different scan rates in a THF solution of [TBA][PF_6] (0.1 M).

Supplementary Table 5. Electrochemical parameters of the oxidation of **3** (Supplementary Fig. 38).

Scan rate (V/s)	E_{pa} (V)*	E_{pc} (V)*	$E_{1/2}$ (V)*	ΔE_p (V)*	i_{pa} (μA)	i_{pc} (μA)	$\lvert i_{pa}/i_{pc} \rvert$
0.050	–0.054	–0.236	–0.145	–0.182	12.37	–12.97	0.95
0.100	–0.048	–0.259	–0.1535	–0.211	13.97	–15.23	0.92
0.200	–0.031	–0.279	–0.155	–0.248	16.66	–18.39	0.91
0.300	–0.018	–0.295	–0.1565	–0.313	18.13	–19.83	0.91
0.400	–0.007	–0.306	–0.1565	–0.313	18.93	–20.47	0.92
0.500	0.005	–0.314	–0.1545	–0.319	19.32	–21.48	0.90
0.800	0.034	–0.334	–0.150	–0.368	21.69	–23.11	0.94

* All referenced to the Fc^+/Fc standard.

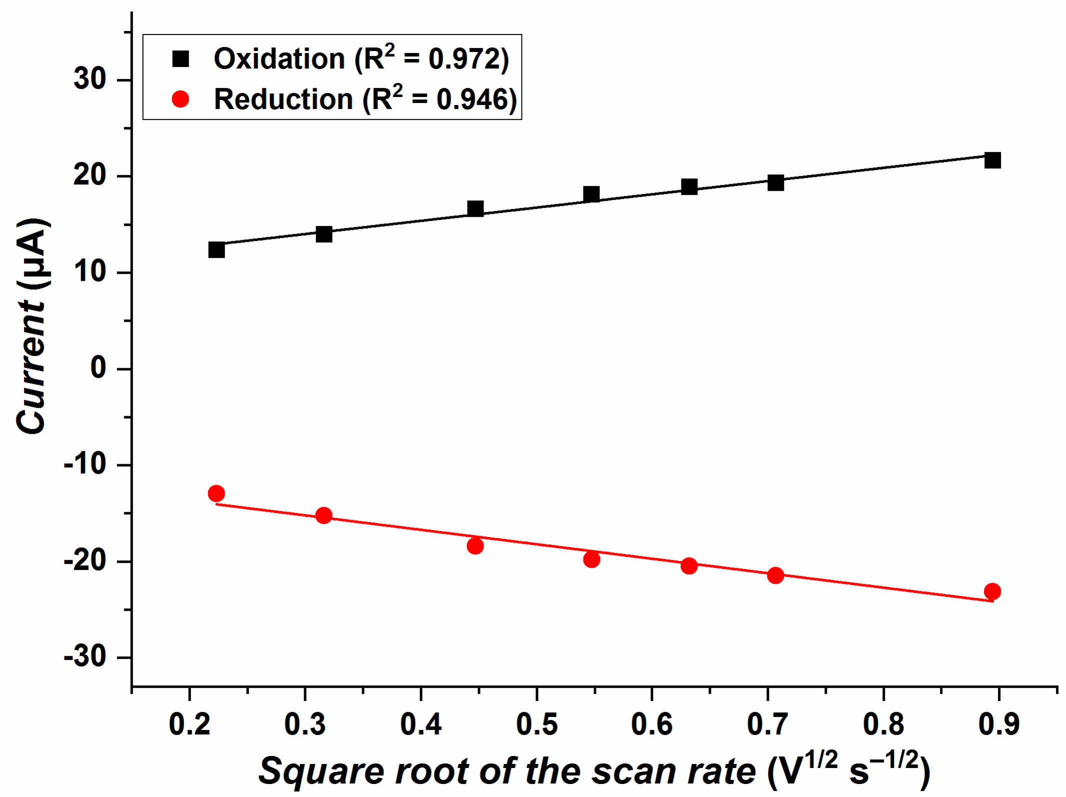

Supplementary Fig. 39. Randles-Ševčík plots of the positive peak currents (i_{pa}) and negative peak currents (i_{pc}) of the redox event at $E_{1/2}$ = –0.155 V vs Fc^+/Fc of **3** (Supplementary Fig. 38).

5. NMR Spectra

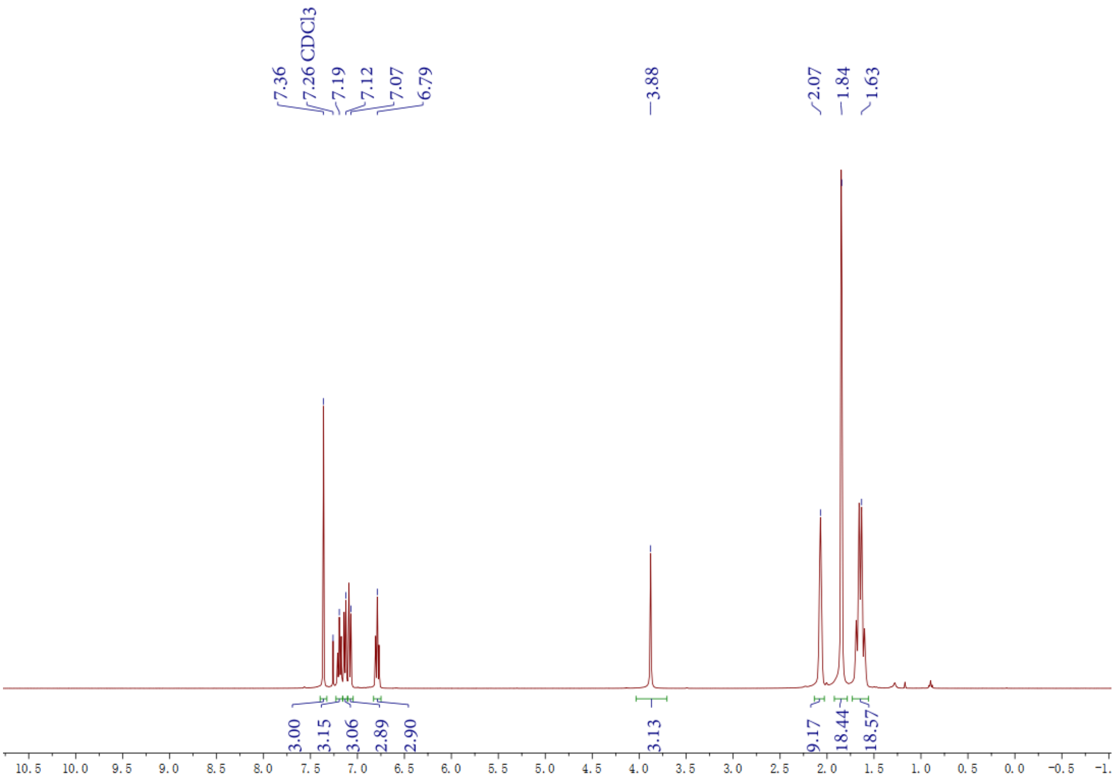

Supplementary Fig. 40. ^{1}H NMR ($CDCl_3$, 400 MHz, 298 K) spectrum of H_3(^{Ad}TPBN$_3$), δ, ppm: 7.36 (s, 3H, C*H* of the anchor ring), 7.19 (td, *J* = 7.6, 1.4 Hz, 3H, C*H* of side rings), 7.12 (dd, *J* = 7.5, 1.2 Hz, 3H, C*H* of side rings), 7.07 (d, *J* = 8.1 Hz, 3H, C*H* of side rings), 6.79 (t, *J* = 7.4 Hz, 3H, C*H* of side rings), 3.88 (s, 3H, N*H*), 2.07 (br s, FWHM = 10.4 Hz, 9H, Ad), 1.84 (m, 18 H, Ad), 1.63 (m, 18 H, Ad).

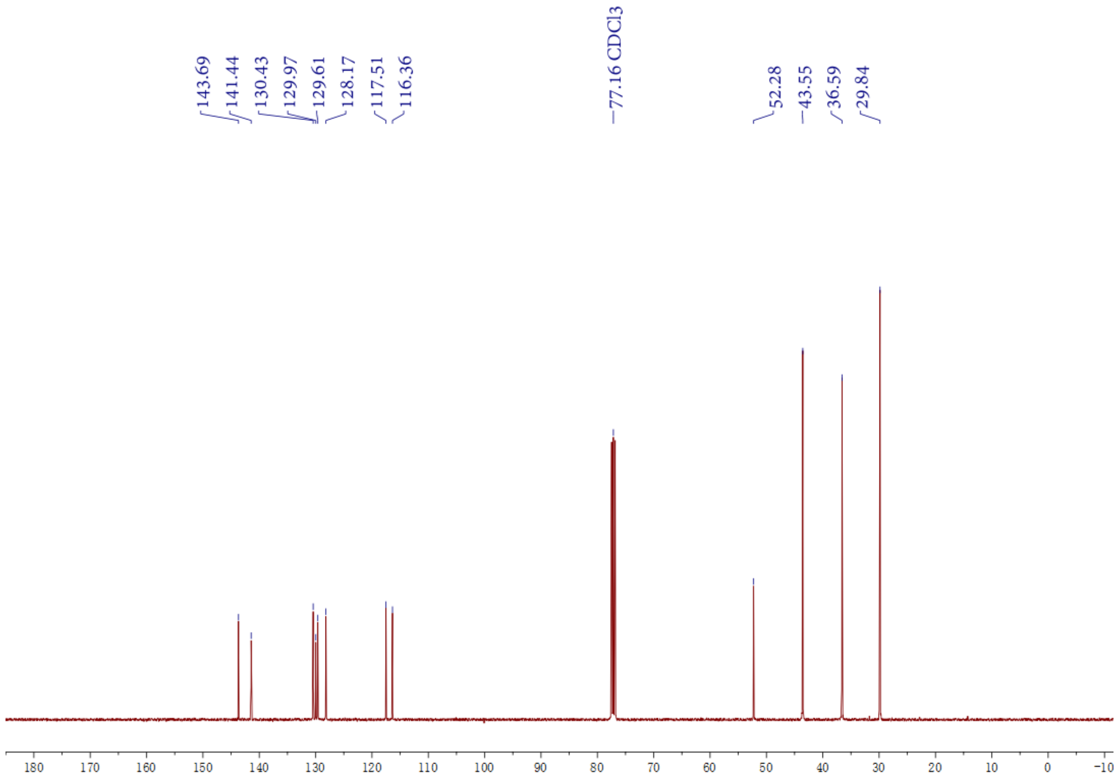

Supplementary Fig. 41. $^{13}C\{H\}$ NMR ($CDCl_3$, 100 MHz, 298 K) spectrum of $H_3(^{Ad}TPBN_3)$, δ, ppm: 143.69 (*C*N of side rings), 141.44 (*C*$_{ipso}$ of the anchor ring), 130.43, 129.97, 129.61, 128.17, 117.51, 116.36 (*C*H of the anchor ring, *C*$_{ipso}$ and four *C*H of side rings), 52.28 (*C*N of Ad), 43.55 (*C*H_2 of Ad), 36.59 (*C*H_2 of Ad), 29.84 (*C*H of Ad).

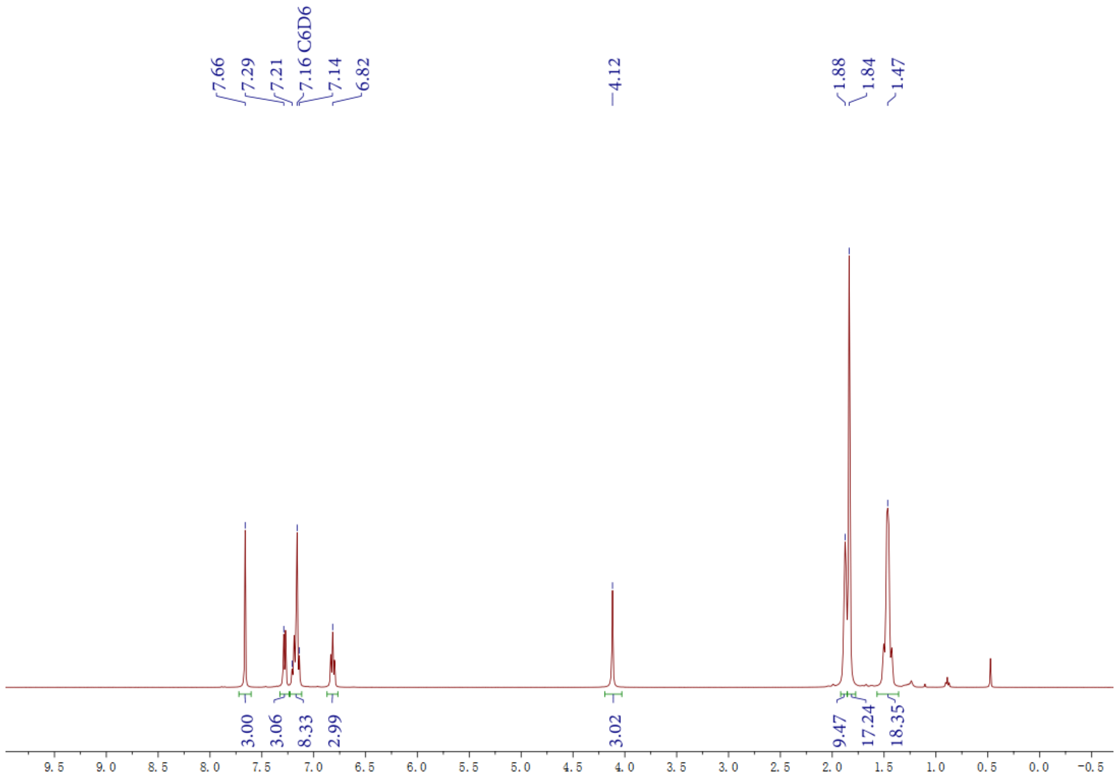

Supplementary Fig. 42. ^{1}H NMR (C_6D_6, 400 MHz, 298 K) spectrum of $H_3(^{Ad}TPBN_3)$, δ, ppm: 7.66 (s, 3H, C*H* of the anchor ring), 7.29 (dd, *J* = 7.5, 1.4 Hz, 3H, C*H* of side rings), 7.14–7.21 (m, 6H, C*H* of side rings, overlapped with C_6D_5H), 6.82 (td, *J* = 7.2, 1.4 Hz, 3H, C*H* of side rings), 4.12 (s, 3H, N*H*), 1.88 (br s, FWHM = 12.6 Hz, 9H, Ad), 1.84 (m, 18 H, Ad), 1.47 (m, 18 H, Ad).

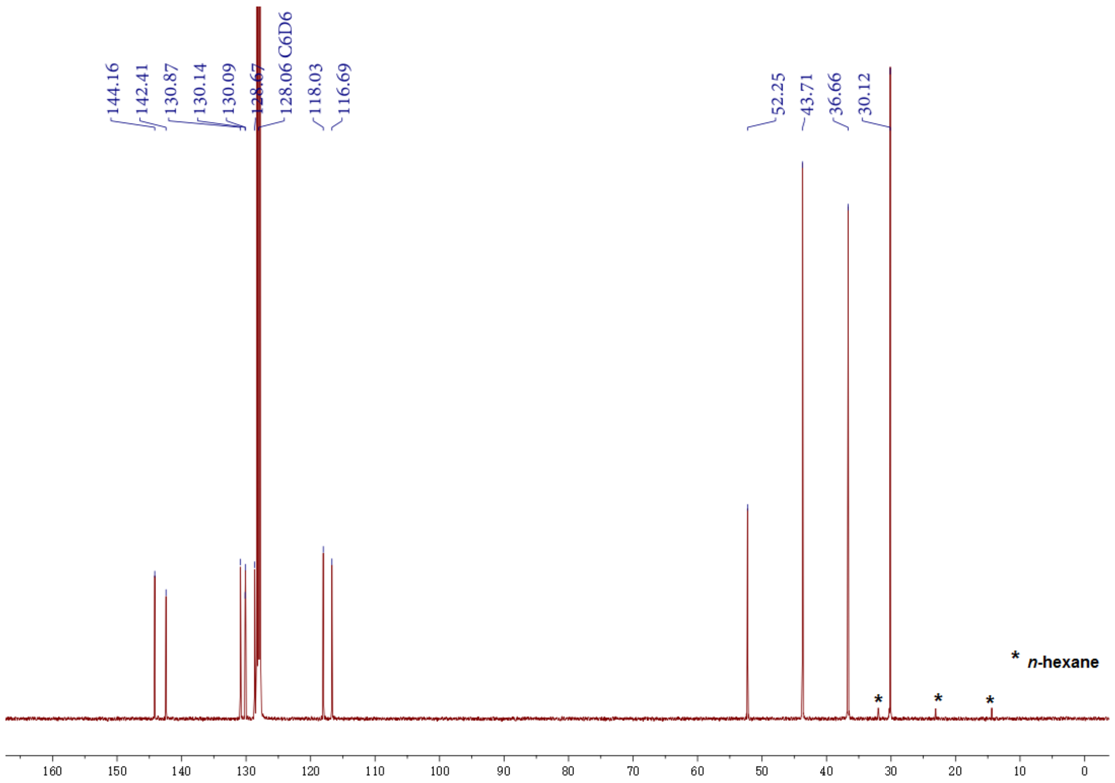

Supplementary Fig. 43. $^{13}C\{^{1}H\}$ NMR (C_6D_6, 100 MHz, 298 K) spectrum of $H_3(^{Ad}TPBN_3)$, δ, ppm: 144.16 (*C*N of side rings), 142.41 (*C*$_{ipso}$ of the anchor ring), 130.87, 130.14, 130.09, 128.67, 118.03, 116.69 (*C*H of the anchor ring, *C*$_{ipso}$ and four *C*H of side rings), 52.25 (*C*N of Ad), 43.71 (*C*H_2 of Ad), 36.66 (*C*H_2 of Ad), 30.12 (*C*H of Ad).

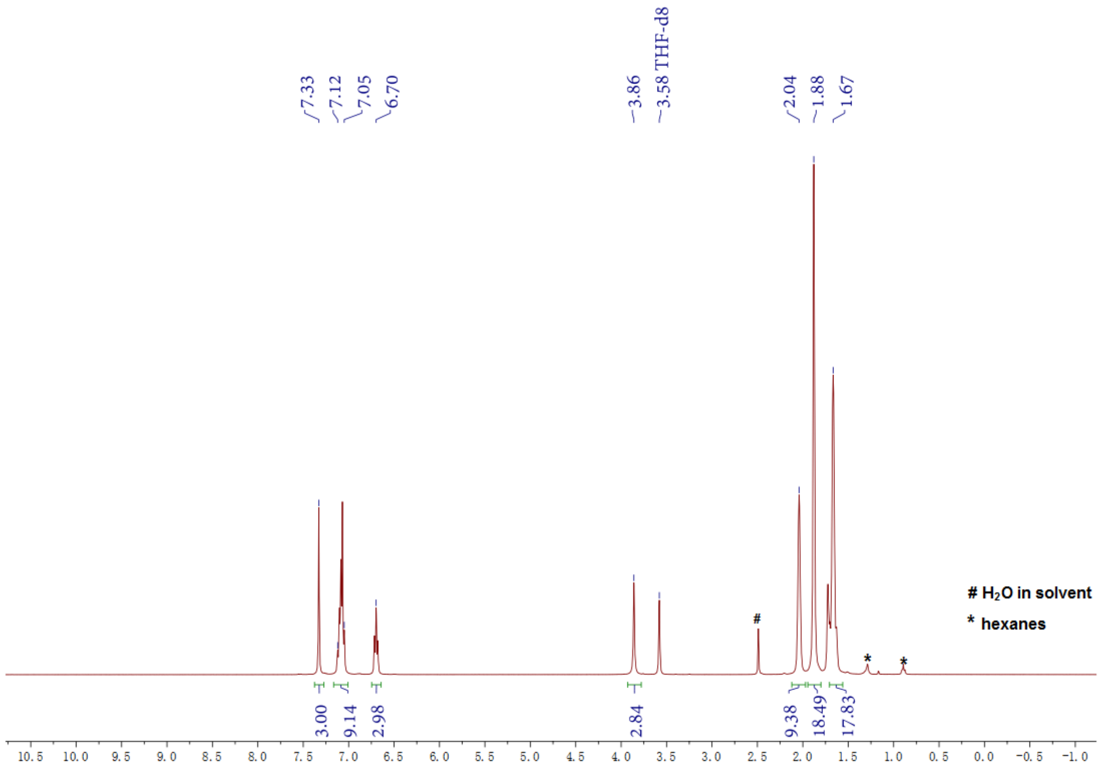

Supplementary Fig. 44. ^{1}H NMR spectrum (C_4D_8O, 400 MHz, 298 K) of H_3(^{Ad}TPBN$_3$), δ, ppm: 7.33 (s, 3H, C*H* of the anchor ring), 7.05–7.12 (m, 9H, C*H* of side rings), 6.70 (t, *J* = 7.1 Hz, C*H* of side rings), 6.82 (td, *J* = 7.2, 1.4 Hz, 3H, C*H* of side rings), 3.86 (s, 3H, N*H*), 2.04 (br s, FWHM = 11.2 Hz, 9H, Ad), 1.88 (s, 18 H, Ad), 1.67 (m, 18 H, Ad).

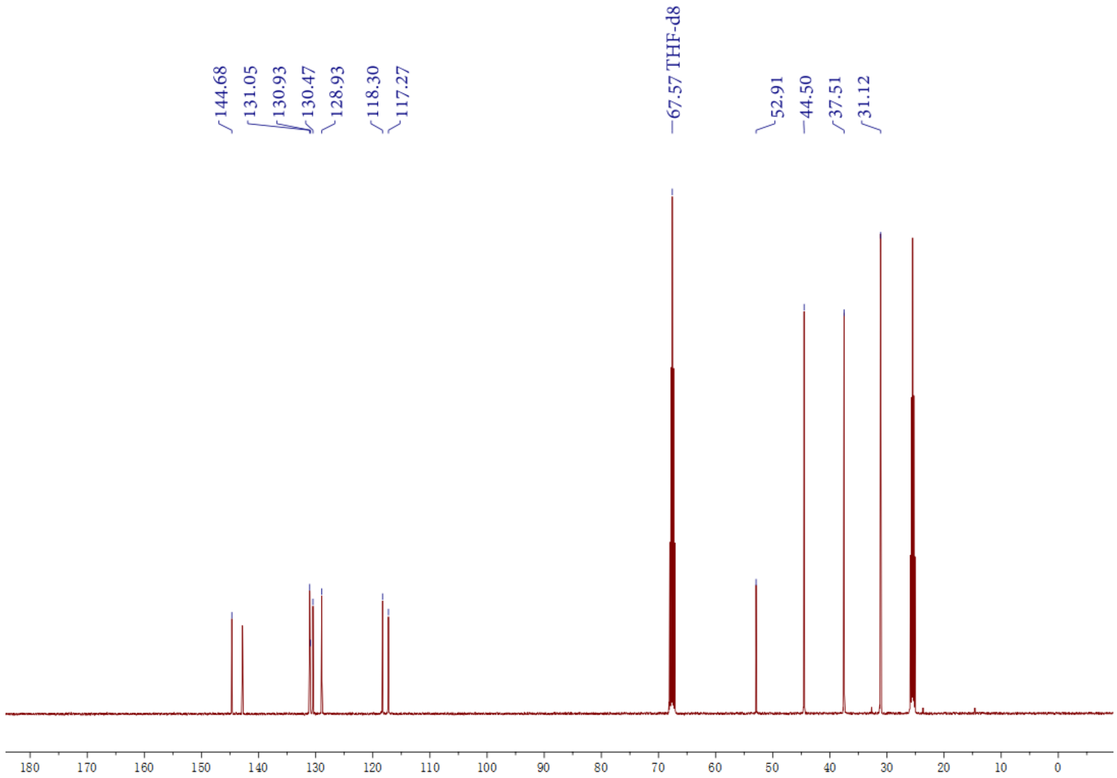

Supplementary Fig. 45. $^{13}C\{^1H\}$ NMR (C_4D_8O, 100 MHz, 298 K) spectrum of $H_3(^{Ad}TPBN_3)$, δ, ppm: 144.68 (*C*N of side rings), 142.82 (*C*$_{ipso}$ of the anchor ring), 131.05, 130.93, 130.47, 128.93, 118.30, 117.27 (*C*H of the anchor ring, *C*$_{ipso}$ and four *C*H of side rings), 52.91 (*C*N of Ad), 44.50 (*C*H_2 of Ad), 37.51 (*C*H_2 of Ad), 31.12 (*C*H of Ad).

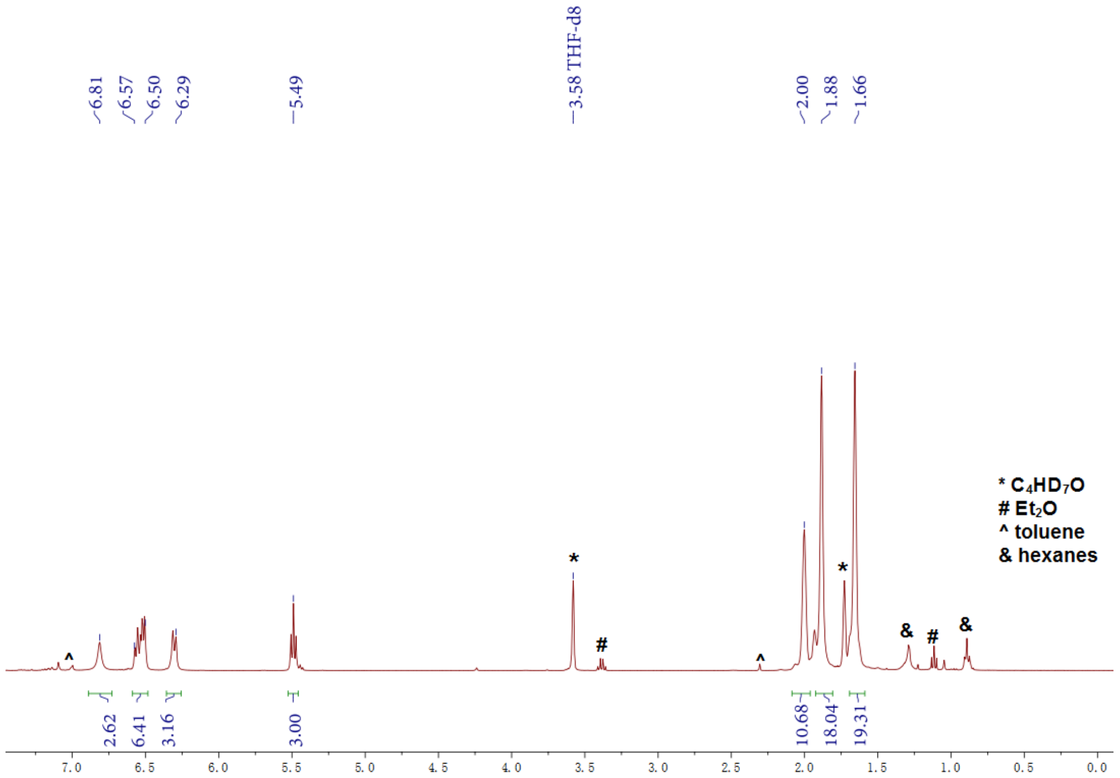

Supplementary Fig. 46. ^{1}H NMR spectrum (C_4D_8O, 400 MHz, 298 K) of K_3(^{Ad}TPBN$_3$) (saturated solution), δ, ppm: 6.81 (s, 3H, C*H* of the anchor ring), 6.50–6.57 (m, 6H, C*H* of side rings), 6.29 (d, *J* = 8.3 Hz, 3H, C*H* of side rings), 5.49 (d, *J* = 6.7 Hz, 3H, C*H* of side rings), 2.00 (br s, FWHM = 12.2 Hz, 9H, Ad), 1.88 (s, 18 H, Ad), 1.66 (s, 18 H, Ad). Less than 2 mol% free pro-ligand H_3(^{Ad}TPBN$_3$) was observed in this spectrum.

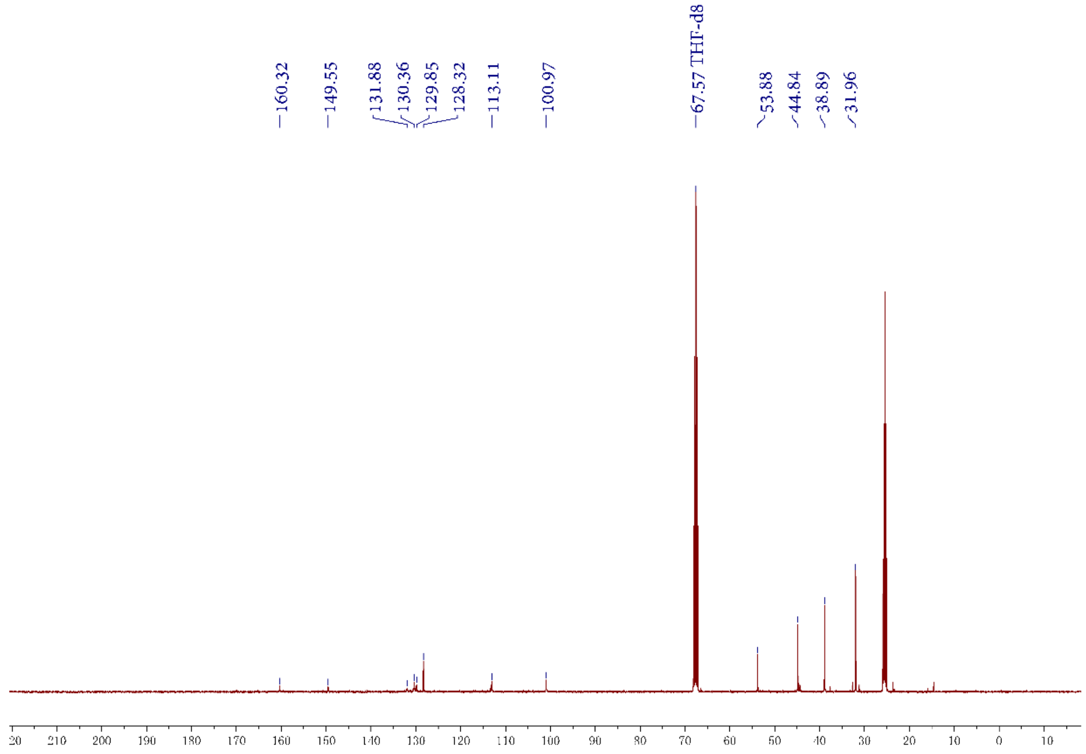

Supplementary Fig. 47. $^{13}C\{^{1}H\}$ NMR (C_4D_8O, 100 MHz, 298 K) spectrum of K_3(^{Ad}TPBN_3) (saturated solution), δ, ppm: 160.32 (*C*N of side rings), 149.55 (C_{ipso} of the anchor ring), 131.88, 130.36, 129.85, 128.32, 113.11, 100.97 (*C*H of the anchor ring, C_{ipso} and four *C*H of side rings), 53.88 (*C*N of Ad), 44.84 (CH_2 of Ad), 38.89 (CH_2 of Ad), 31.96 (*C*H of Ad). Under this NMR experiment condition, despite a low signal-to-noise ratio for K_3(^{Ad}TPBN_3) due to limited solubility, a full reasonable assignment could still be made. A minor set of peaks of free pro-ligand H_3(^{Ad}TPBN_3) (Ad region, +20 to +55 ppm) was also observed in this spectrum, in accord with ^{1}H NMR spectrum.

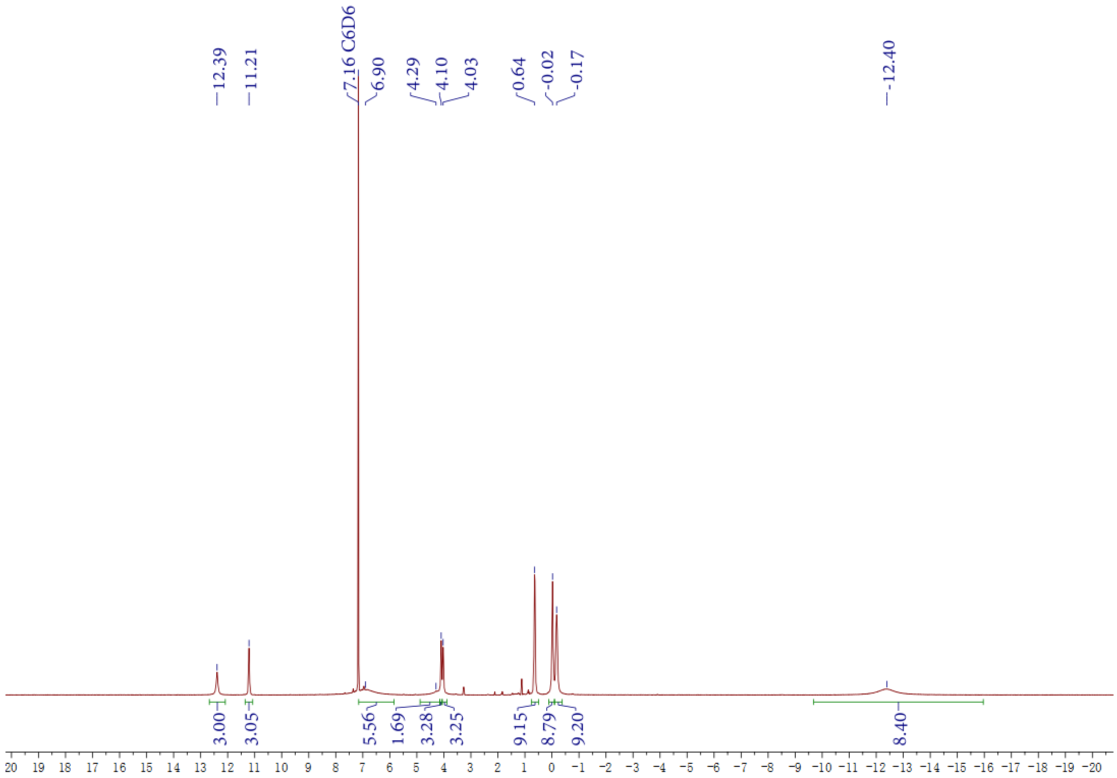

Supplementary Fig. 48. ^{1}H NMR (C_6D_6, 400 MHz, 298 K) spectrum of (^{Ad}TPBN_3)U (**1**) (saturated solution), δ, ppm: 12.39 (s, 3H, C*H* of side rings), 11.21 (s, 3H, C*H* of side rings), 6.90 (br s, FWHM ≈ 300 Hz, 9H, Ad, overlapped with C_6D_5H), 4.29 (br s, FWHM ≈ 160 Hz, 3H, C*H* of the anchor ring, overlapped with others), 4.10 (s, 3H, C*H* of side rings), 4.03 (s, 3H, C*H* of side rings), 0.64 (s, 9H, Ad), –0.02 (s, 9H, Ad), –0.17 (s, 9H, Ad), –12.40 (br s, FWHM = 320 Hz, 9H, Ad).

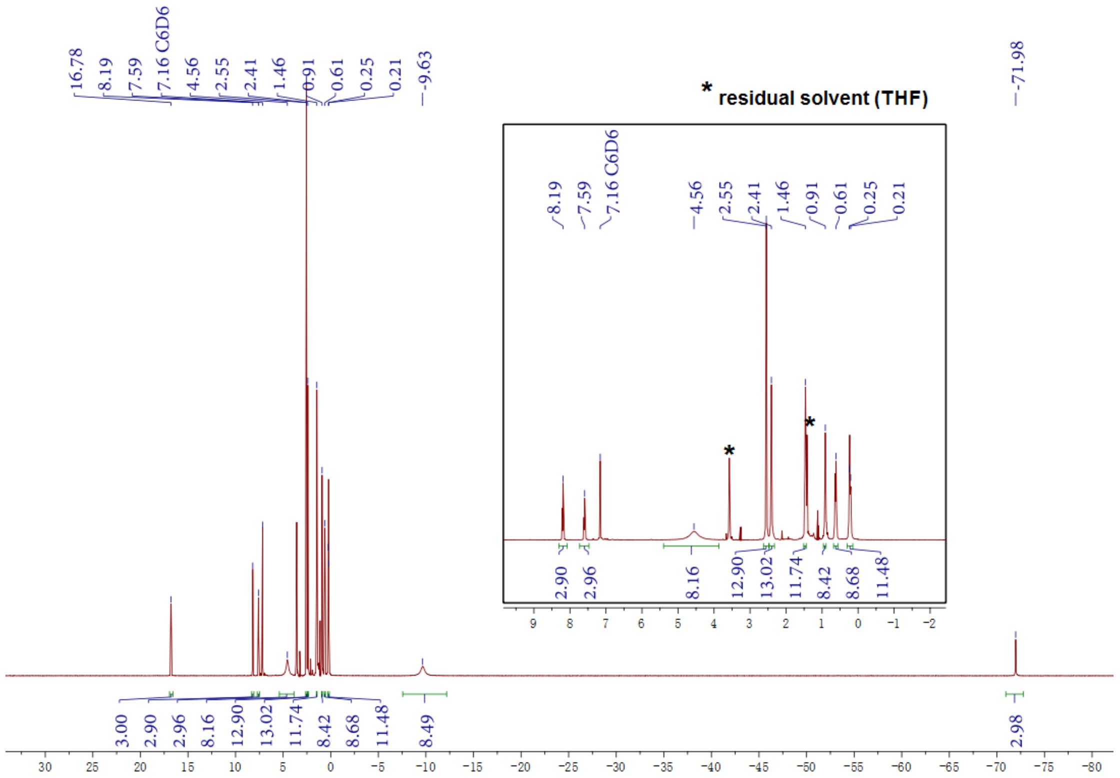

Supplementary Fig. 49. ^{1}H NMR (C_6D_6, 400 MHz, 298 K) spectrum of [K(crypt)][(^{Ad}TPBN$_3$)U] (**2**) in the presence of a small amount of THF (to improve solubility), δ, ppm: 16.78 (d, J = 8.4 Hz, 3H, CH of side rings), 8.19 (t, J = 7.0 Hz, 3H, CH of side rings), 7.59 (t, J = 7.6 Hz, 3H, CH of side rings), 4.56 (br s, FWHM = 110 Hz, 9H, Ad), 2.55 (s, 12H, OCH_2CH_2O of [K(crypt)]$^+$), 2.41 (t, J = 4.0 Hz, 12H, NCH$_2$CH_2O of [K(crypt)]$^+$), 1.46 (t, J = 4.0 Hz, 12H, NCH_2CH$_2$O of [K(crypt)]$^+$), 0.91 (br s, FWHM = 11.7 Hz, 9H, Ad), 0.61 (m, 9H, Ad), 0.21–0.25 (m, 12H, CH of side rings (3H) & Ad (9H)), –9.63 (br s, FWHM = 180 Hz, 9H, Ad), –71.98 (s, 3H, CH of the anchor ring). The region between –2.5 and 9.5 ppm was enlarged for clarification.

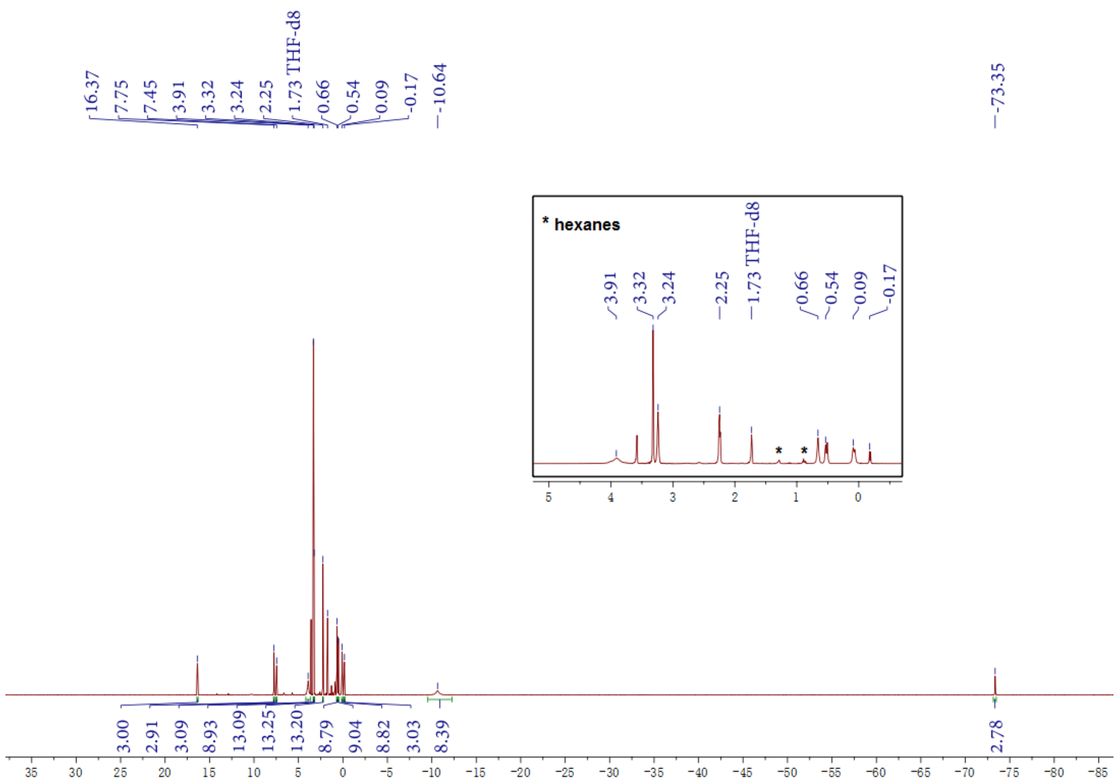

Supplementary Fig. 50. ^{1}H NMR (C_4D_8O, 400 MHz, 298 K) spectrum of [K(crypt)][($^{Ad}TPBN_3$)U] (**2**), δ, ppm: 16.37 (d, *J* = 8.4 Hz, 3H, C*H* of side rings), 7.75 (t, *J* = 7.2 Hz, 3H, C*H* of side rings), 7.45 (t, *J* = 7.8 Hz, 3H, C*H* of side rings), 3.91 (br s, FWHM = 65 Hz, 9H, Ad), 3.32 (s, 12H, OCH_2CH_2O of $[K(crypt)]^+$), 3.24 (t, *J* = 4.4 Hz, 12H, NCH_2CH_2O of $[K(crypt)]^+$), 2.25 (t, *J* = 4.5 Hz, 12H, NCH_2CH_2O of $[K(crypt)]^+$), 0.66 (br s, FWHM = 11.6 Hz, 9H, Ad), 0.54 (m, 9H, Ad), 0.09 (m, 9H, Ad), –0.17 (d, *J* = 6.6 Hz, 3H, C*H* of side rings), –10.64 (br s, FWHM = 160 Hz, 9H, Ad), –73.35 (s, 3H, C*H* of the anchor ring). The region between –0.5 and 5.0 ppm was enlarged for clarification.

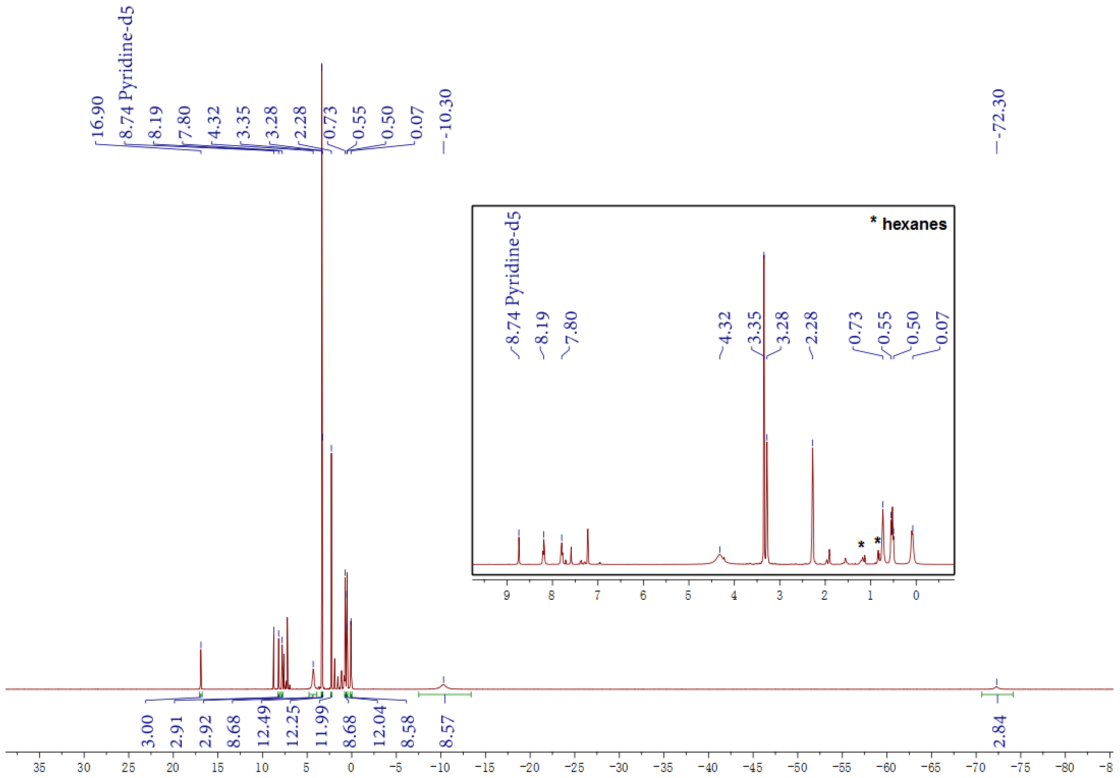

Supplementary Fig. 51. ^{1}H NMR (C_5D_5N, 400 MHz, 298 K) spectrum of [K(crypt)][(^{Ad}TPBN$_3$)U] (**2**), δ, ppm: 16.90 (d, J = 8.6 Hz, 3H, C*H* of side rings), 8.19 (t, J = 7.0 Hz, 3H, C*H* of side rings), 7.80 (t, J = 7.8 Hz, 3H, C*H* of side rings), 4.32 (br s, FWHM = 45 Hz, 9H, Ad), 3.35 (s, 12H, OC*H*$_2$C*H*$_2$O of [K(crypt)]$^+$), 3.28 (t, J = 4.4 Hz, 12H, NCH$_2$C*H*$_2$O of [K(crypt)]$^+$), 2.28 (t, J = 4.5 Hz, 12H, NC*H*$_2$CH$_2$O of [K(crypt)]$^+$), 0.73 (br s, FWHM = 13.4 Hz, 9H, Ad), 0.55–0.50 (m, 12 H, Ad (9H) & C*H* of side rings (3H)), 0.07 (m, 9H, Ad), –10.30 (br s, FWHM = 300 Hz, 9H, Ad), –72.30 (s, 3H, C*H* of the anchor ring). The region between –0.5 and 9.5 ppm was enlarged for clarification.

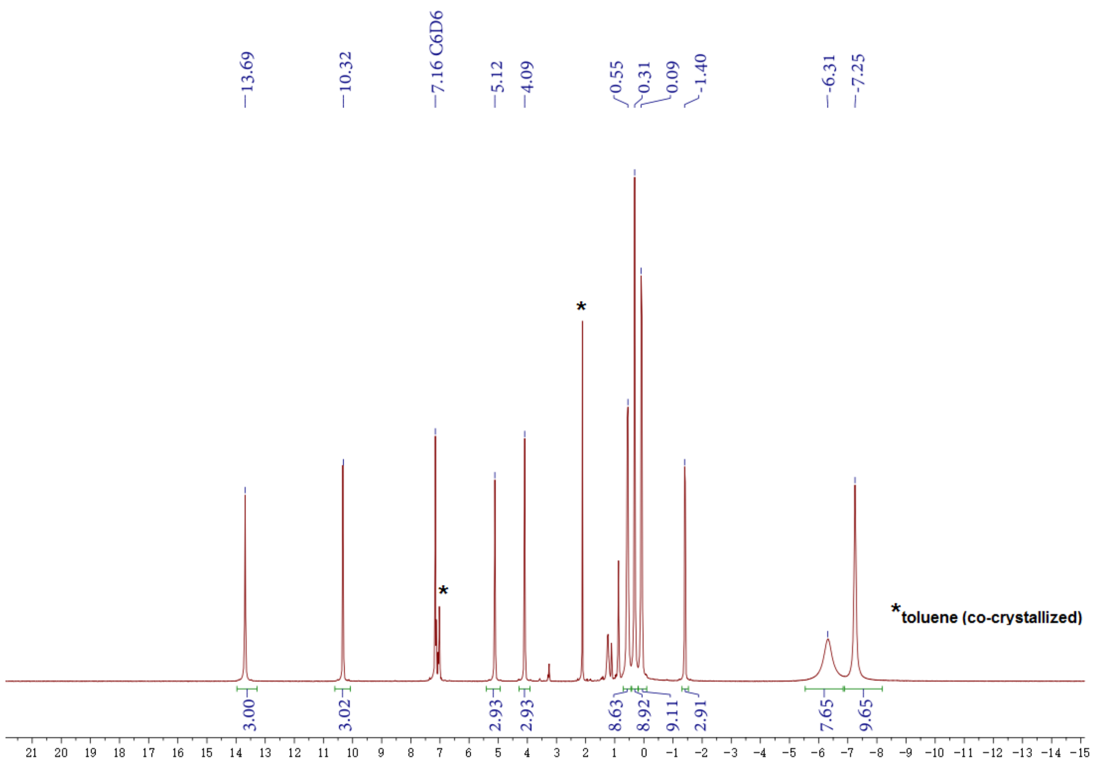

Supplementary Fig. 52. ^{1}H NMR (C_6D_6, 400 MHz, 298 K) spectrum of ($^{Ad}TPBN_3$)UO (**3**), δ, ppm: 13.69 (s, 3H, C*H* of the anchor ring), 10.32 (d, *J* = 7.4 Hz, 3H, C*H* of side rings), 5.12 (t, *J* =7.3 Hz, 3H, C*H* of side rings), 4.09 (t, *J* = 7.2 Hz, 3H, C*H* of side rings), 0.55 (m, 9H, Ad), 0.31 (br s, FWHM = 12.7 Hz, 9H, Ad), 0.09 (m, 9H, Ad), –1.40 (d, *J* = 8.1 Hz, C*H* of side rings), –6.31 (br s, FWHM = 140 Hz, 9H, Ad), –7.25 (br s, FWHM = 31.1 Hz, 9H, Ad).

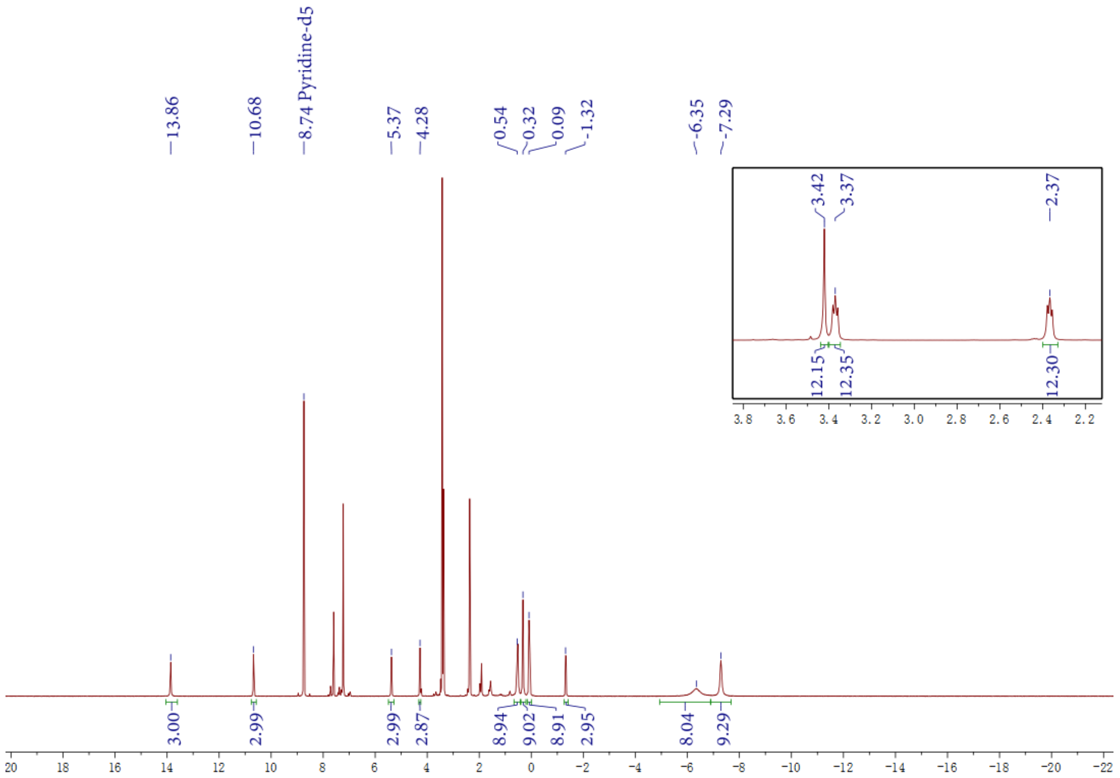

Supplementary Fig. 53. ^{1}H NMR (C_5D_5N, 400 MHz, 298 K) spectrum of (^{Ad}TPBN$_3$)UO (**3**) and 2.2.2-cryptand as the products of the reaction between **4** and AgOTf. For **3**, δ, ppm: 13.77 (s, 3H, C*H* of the anchor ring), 10.58 (d, *J* = 7.3 Hz, 3H, C*H* of side rings), 5.29 (t, *J* =7.6 Hz, 3H, C*H* of side rings), 4.19 (t, *J* = 7.2 Hz, 3H, C*H* of side rings), 0.44 (m, 9H, Ad), 0.23 (br s, FWHM = 12.6 Hz, 9H, Ad), –0.01 (m, 9H, Ad), –1.41 (d, *J* = 8.3 Hz, C*H* of side rings), –6.42 (br s, FWHM = 180 Hz, 9H, Ad), –7.38 (br s, FWHM = 31.0 Hz, 9H, Ad). The region between 2.2 and 3.8 ppm was enlarged to show the concurrent formation of one equivalent of free 2.2.2-cryptand, δ, ppm: 3.34 (s, 12H, OCH_2CH_2O of [K(crypt)]$^+$), 3.28 (t, *J* = 4.7 Hz, 12H, NCH$_2$CH_2O of [K(crypt)]$^+$), 2.28 (t, *J* = 4.5 Hz, 12H, NCH_2CH$_2$O of [K(crypt)]$^+$).

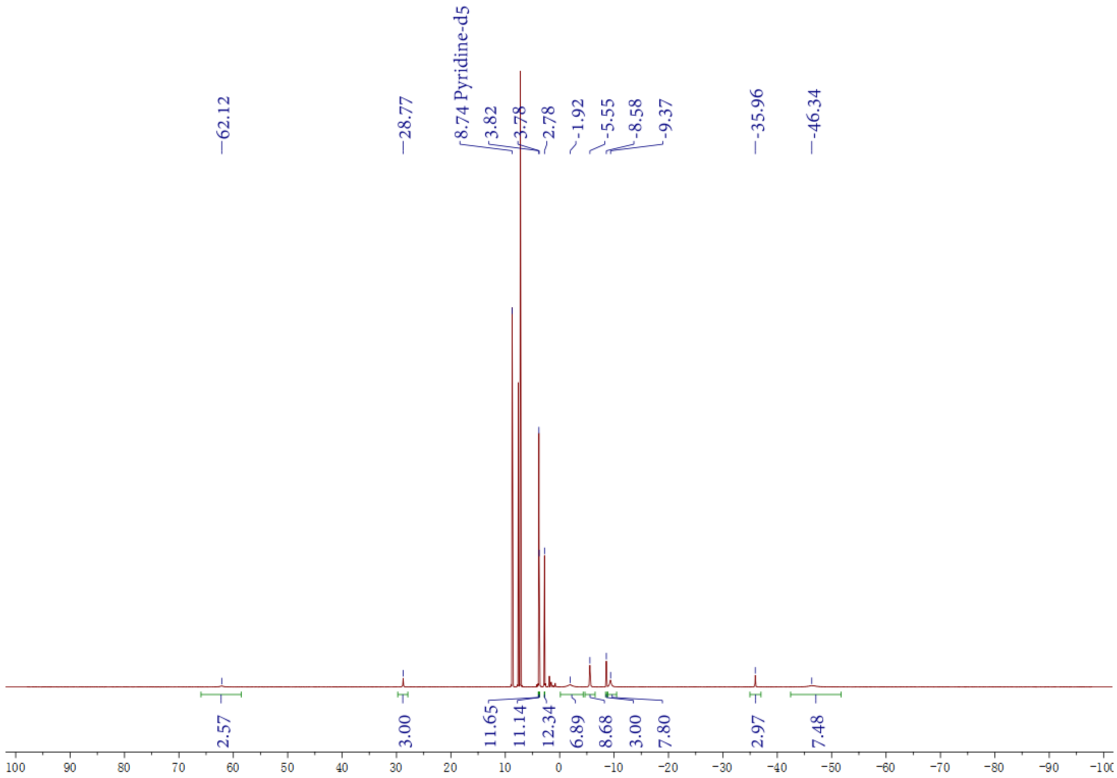

Supplementary Fig. 54. ^{1}H NMR (C_5D_5N, 400 MHz, 298 K) spectrum of [K(crypt)][(^{Ad}TPBN$_3$)UO] (**4**) (saturated solution), δ, ppm: 62.12 (br s, FWHM = 320 Hz, 3H, C*H* of the anchor ring), 28.77 (s, 3H, C*H* of side rings), 3.82 (s, 12H, OCH_2CH_2O of [K(crypt)]$^+$), 3.78 (t, *J* = 4.7 Hz, 12H, NCH$_2$CH_2O of [K(crypt)]$^+$), 2.78 (m, 15H, NCH_2CH$_2$O of [K(crypt)]$^+$ (12H) & C*H* of side rings (3H)), –1.92 (br s, FWHM = 340 Hz, 9H, Ad), –5.55 (br s, FWHM = 39.7 Hz, 9H, Ad), –8.58 (s, 3H, C*H* of side rings), –9.37 (br s, FWHM = 140 Hz, 9H, Ad), –35.96 (s, 3H, C*H* of side rings), –46.34 (br s, FWHM = 680 Hz, 9H, Ad). One peak (9H) of Ad groups was not found in the range of –500 to +500 ppm probably due to signal broadening. Notably, splitting of C*H* peaks of side rings could not be observed under this NMR experiment condition.

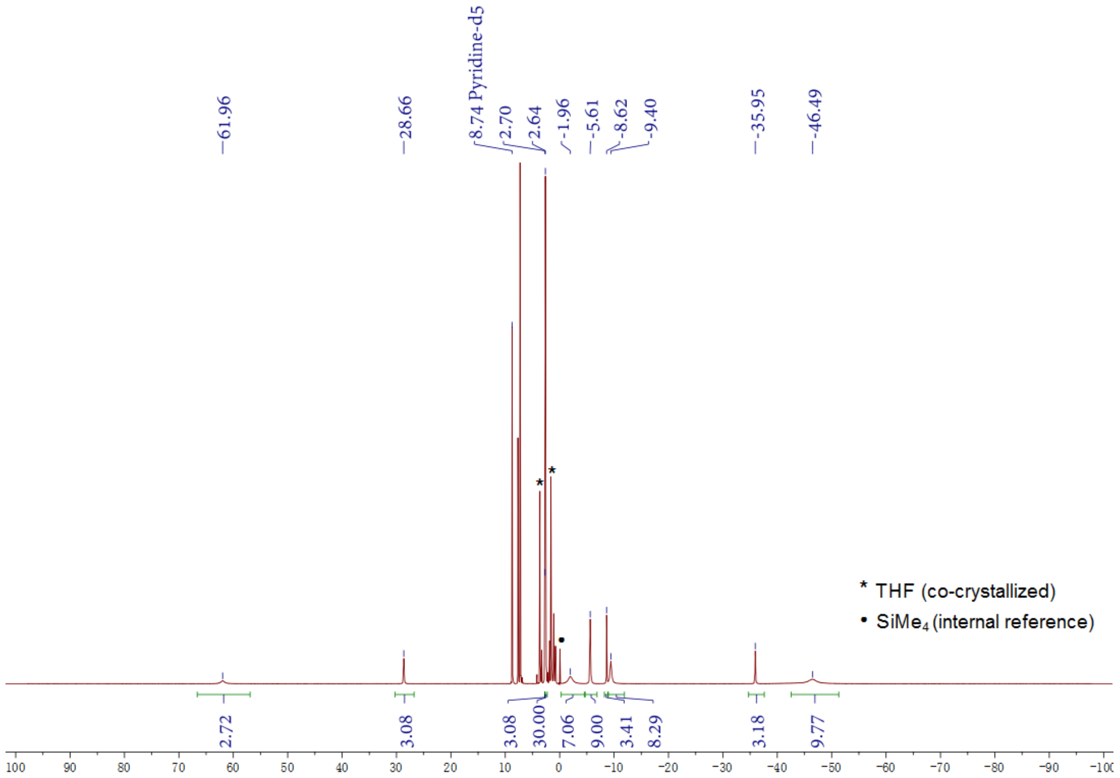

Supplementary Fig. 55. ^{1}H NMR (C_5D_5N, 400 MHz, 298 K) spectrum of $[Cp^*_2Co][(^{Ad}TPBN_3)UO]$ (**4′**), δ, ppm: 61.96 (br s, FWHM = 280 Hz, 3H, C*H* of the anchor ring), 28.86 (s, 3H, C*H* of side rings), 2.70 (s, 3H, C*H* of side rings), 2.64 (s, 30H, CH_3 of $[Cp^*_2Co]^+$), –1.96 (br s, FWHM = 320 Hz, 9H, Ad), –5.61 (br s, FWHM = 43.9 Hz, 9H, Ad), –8.62 (s, 3H, C*H* of side rings), –9.40 (br s, FWHM = 126 Hz, 9H, Ad), –35.95 (s, 3H, C*H* of side rings), –46.49 (br s, FWHM = 720 Hz, 9H, Ad). One peak (9H) of Ad groups was not found in the range of –500 to +500 ppm probably due to signal broadening. Besides, splitting of C*H* peaks of side rings could not be observed under this NMR experiment condition.

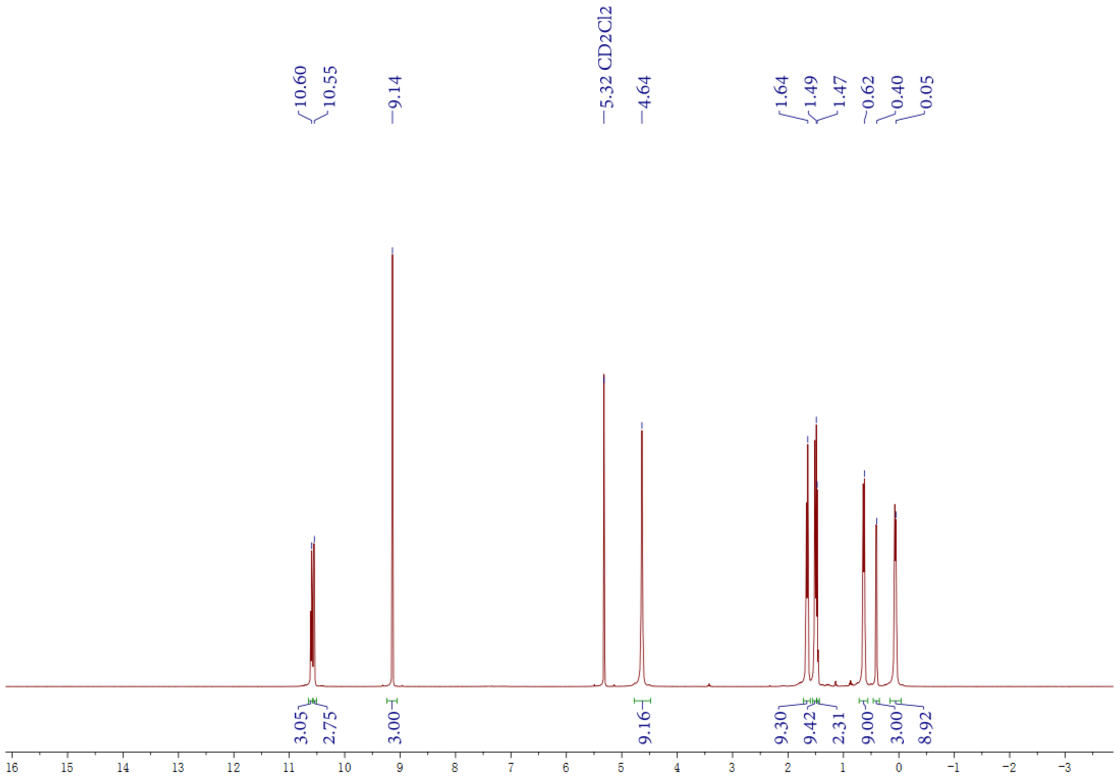

Supplementary Fig. 56. ^{1}H NMR (CD_2Cl_2, 500 MHz, 298 K) spectrum of $[(^{Ad}TPBN_3)UO][SbF_6]$ (**5**), δ, ppm: 10.60 (td, *J* = 7.8, 1.4 Hz, 3H, C*H* of side rings), 10.55 (dd, *J* = 7.7, 1.5 Hz, 3H, C*H* of side rings), 9.14 (s, 3H, C*H* of the anchor ring), 4.64 (br s, FWHM = 12.4 Hz, 9H, Ad), 1.64 (m, 9H, Ad), 1.49 (m, 9H, Ad), 1.47 (t, *J* = 7.5 Hz, 3H, C*H* of side rings), 0.62 (m, 9H, Ad), 0.40 (d, *J* = 8.4 Hz, 3H, C*H* of side rings), 0.05 (m, 9 H, Ad). Based on 2D NMR results (Supplementary Figs. 60–61), a detailed assignment could be made (Supplementary Fig. 57).

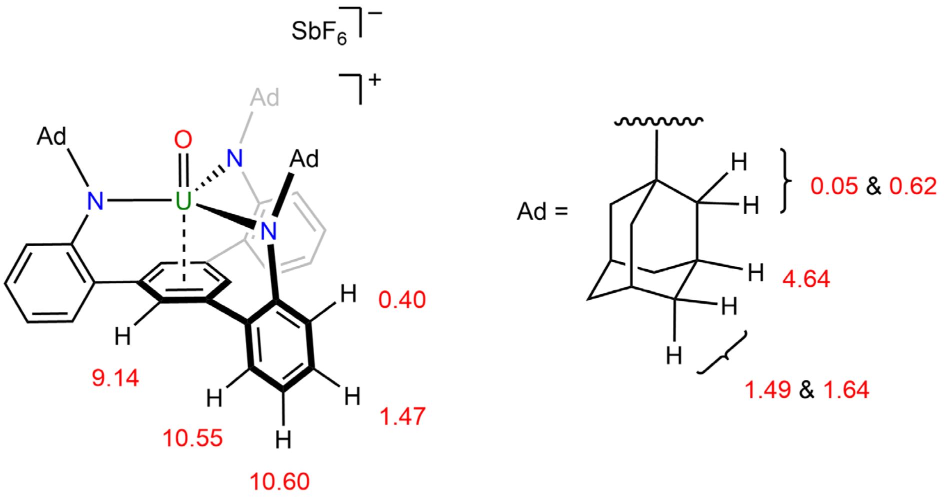

Supplementary Fig. 57. Assignment of the ^{1}H NMR peaks (δ, ppm) of **5** (Supplementary Fig. 56).

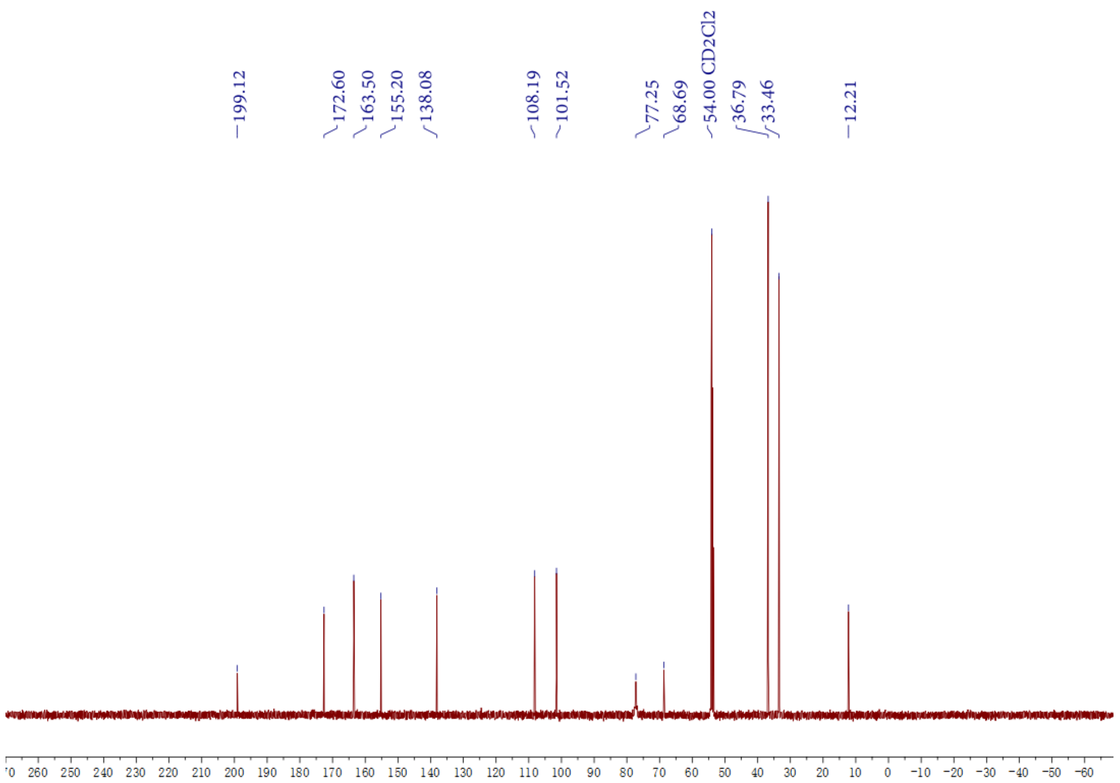

Supplementary Fig. 58. $^{13}C\{^1H\}$ NMR (CD_2Cl_2, 125 MHz, 298 K) spectrum of [($^{Ad}TPBN_3$)UO][SbF_6] (**5**), δ, ppm: 199.12, 172.60, 163.50, 155.20, 138.08, 108.19, 101.52 (C_{aryl}), 77.25 (C_{Ad}), 68.69 (C_{aryl}), 36.79, 33.46, 12.21 (C_{Ad}). Based on 2D NMR results (Supplementary Figs. 60–61), a detailed assignment could be made (Supplementary Fig. 59).

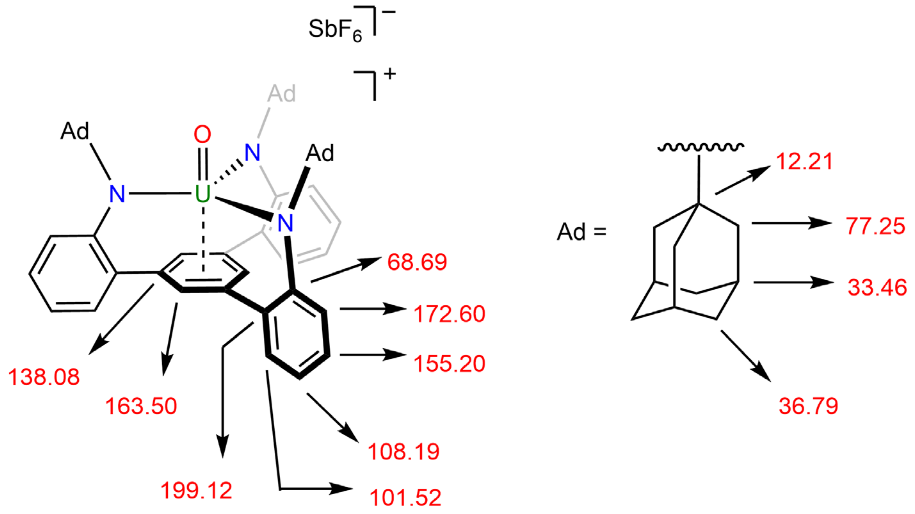

Supplementary Fig. 59. Assignment of the $^{13}C\{^1H\}$ NMR peaks (δ, ppm) of **5** (Supplementary Fig. 58).

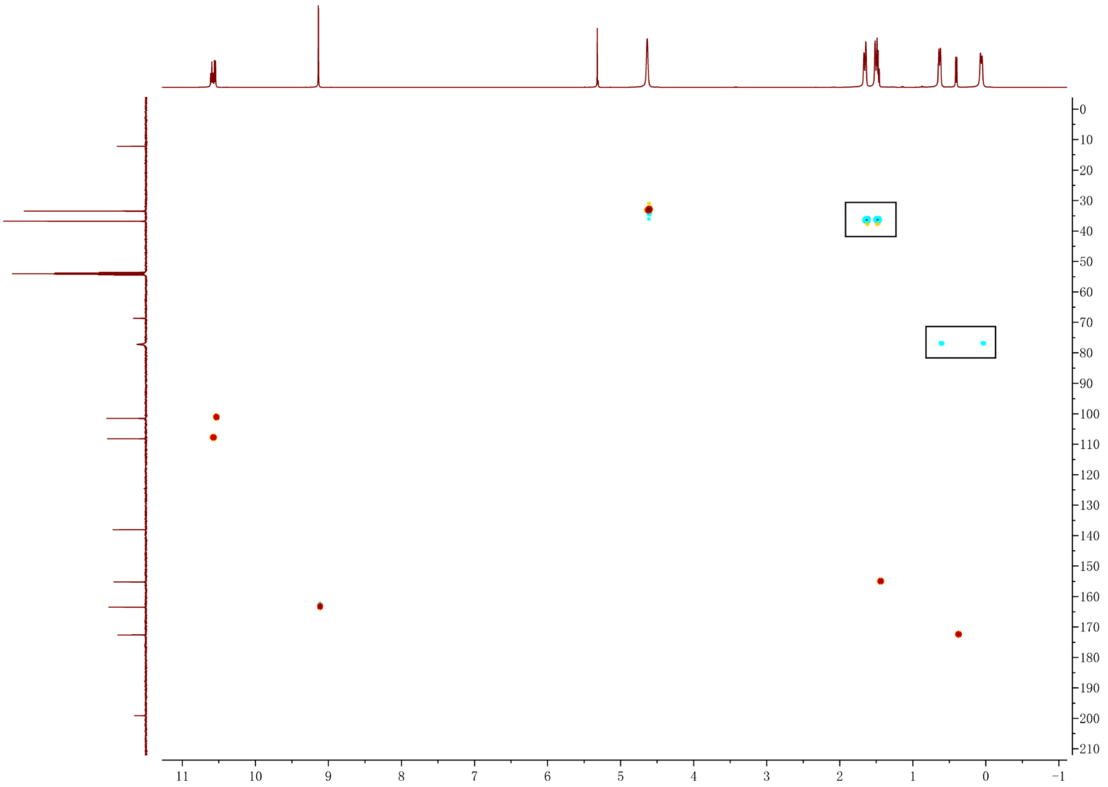

Supplementary Fig. 60. ^{1}H–^{13}C HSQC (heteronuclear singular quantum correlation) (CD_2Cl_2, 500/125 MHz, 298 K) spectrum of [(^{Ad}TPBN$_3$)UO][SbF_6] (**5**). The CH_1 correlation peaks are shown in orange, while the CH_2 correlation peaks are shown in cyan and highlighted by inner boxes.

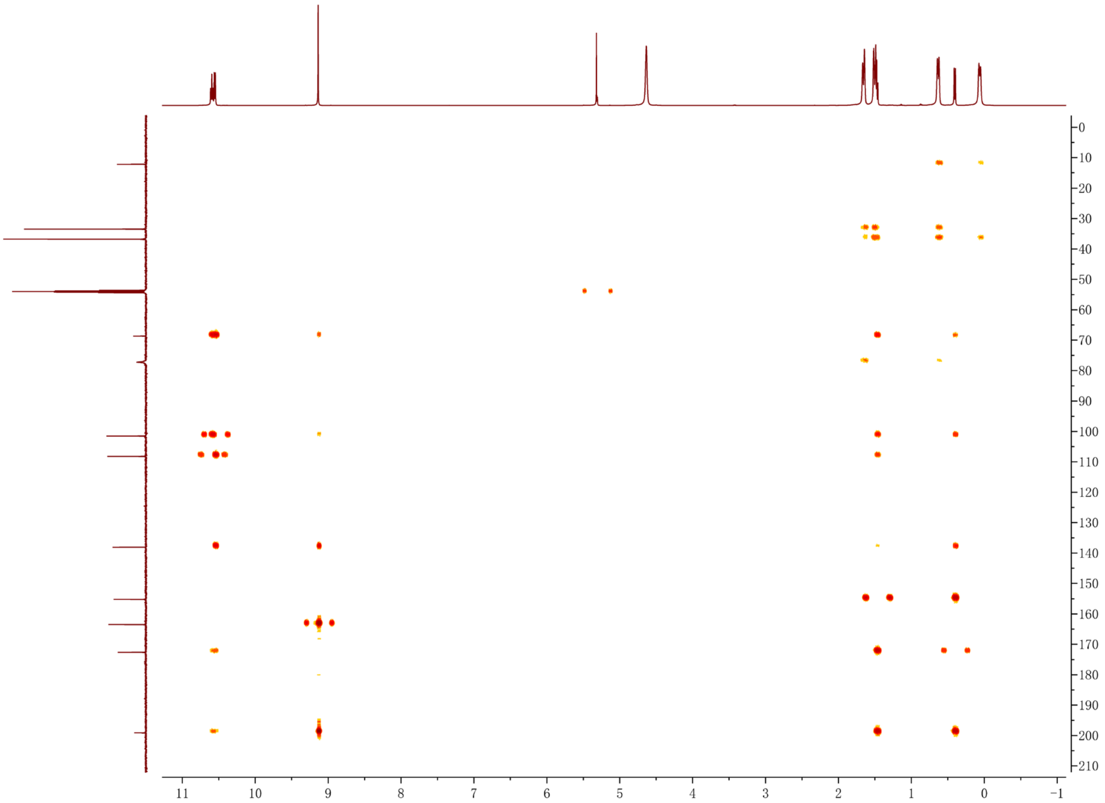

Supplementary Fig. 61. ^1H–^{13}C HMBC (heteronuclear multiple bond correlation) (CD_2Cl_2, 500/125 MHz, 298 K) spectrum of [(^{Ad}TPBN$_3$)UO][SbF_6] (**5**). The correlation peaks are shown in orange. No correlation between aryl and adamantyl groups (i.e., $^1H_{Ad}$–$^{13}C_{aryl}$ or $^1H_{aryl}$–$^{13}C_{Ad}$) was observed.

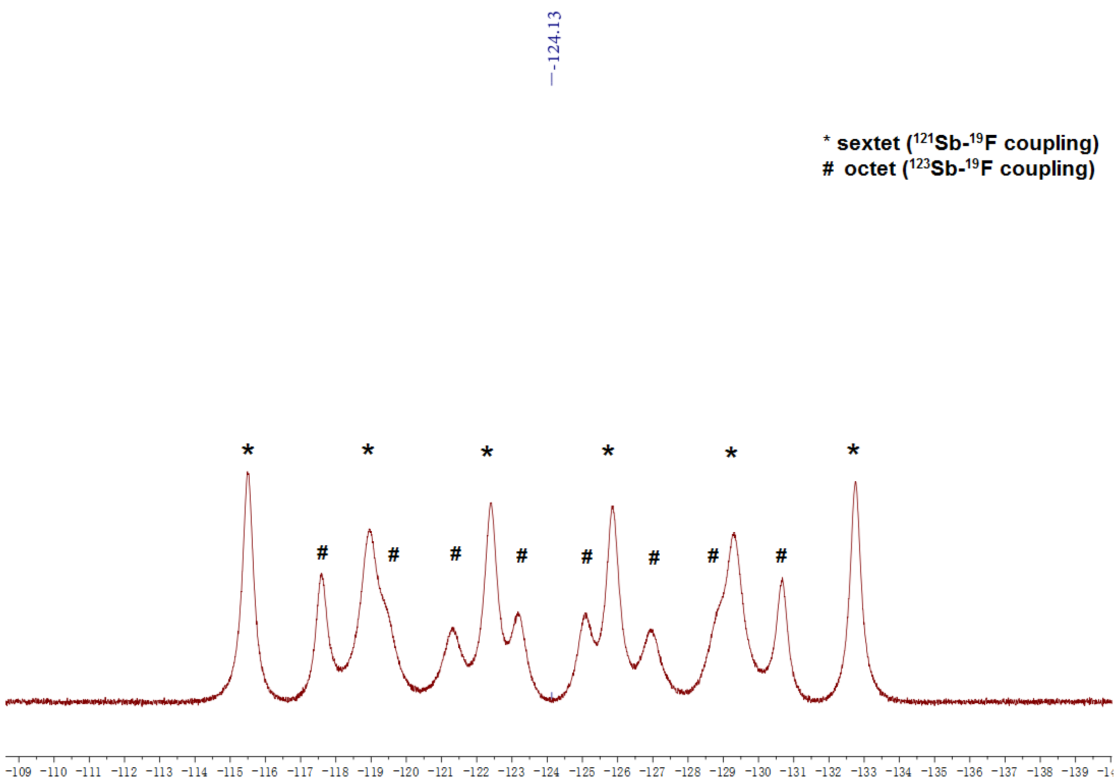

Supplementary Fig. 62. ^{19}F NMR (CD_2Cl_2, 565 MHz, 298 K) spectrum of [(^{Ad}TPBN$_3$)UO][SbF$_6$] (**5**), δ, ppm: –124.13 (m: sextet ($J_{121Sb–19F}$ = 1947 Hz) & octet ($J_{123Sb–19F}$ = 1054 Hz), [SbF_6]$^-$). Note: nucleus spin quantum number I_{121Sb} = 5/2, I_{123Sb} = 7/2; natural abundance ^{121}Sb 57%, ^{123}Sb 43%.

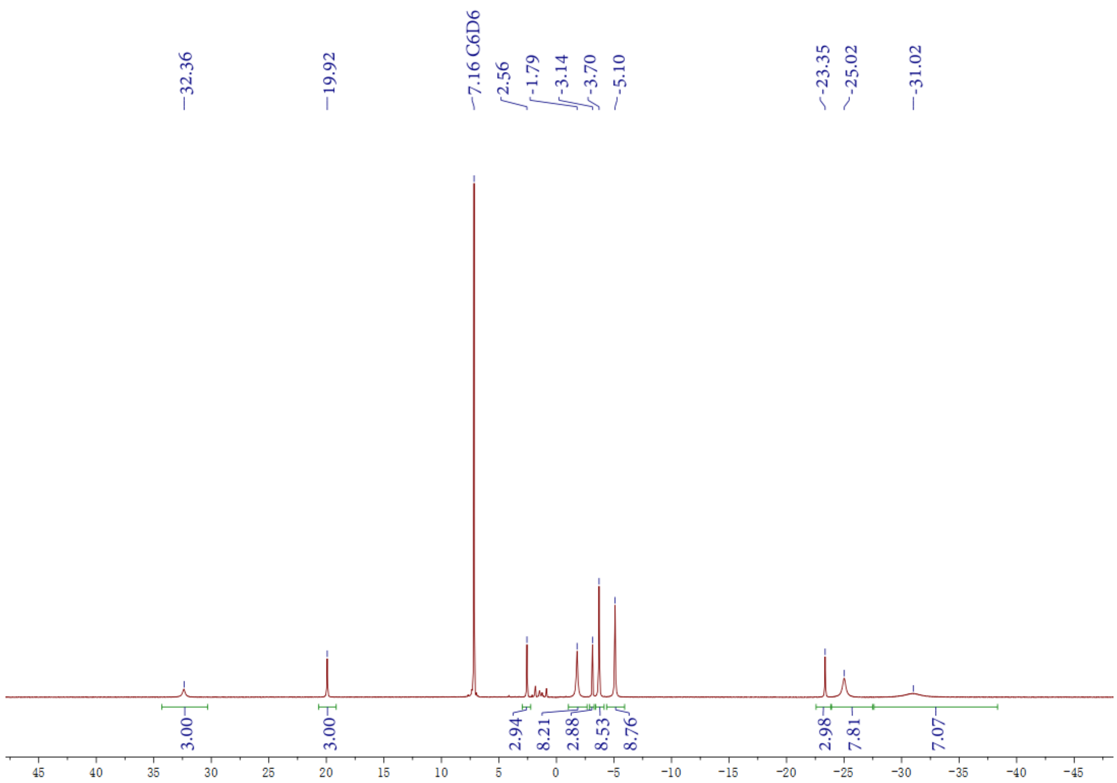

Supplementary Fig. 63. ^{1}H NMR (C_6D_6, 400 MHz, 298 K) spectrum of ($^{Ad}TPBN_3$)UF (**6**) (saturated solution), δ, ppm: 32.36 (s, 3H, C*H* of the anchor ring), 19.92 (d, *J* = 7.8 Hz, 3H, C*H* of side rings), 2.56 (t, *J* = 5.8 Hz, 3H, C*H* of side rings), –1.79 (br s, FWHM = 56.8 Hz, 9H, Ad), –3.14 (t, *J* = 6.2 Hz, 3H, C*H* of side rings), –3.70 (br s, FWHM = 25.7 Hz, 9H, Ad), –5.10 (br s, FWHM = 27.9 Hz, 9H, Ad), –23.35 (d, *J* = 8.0 Hz, 3H, C*H* of side rings), –25.02 (br s, FWHM = 160 Hz, 9H, Ad), –31.02 (br s, FWHM = 700 Hz, 9H, Ad).

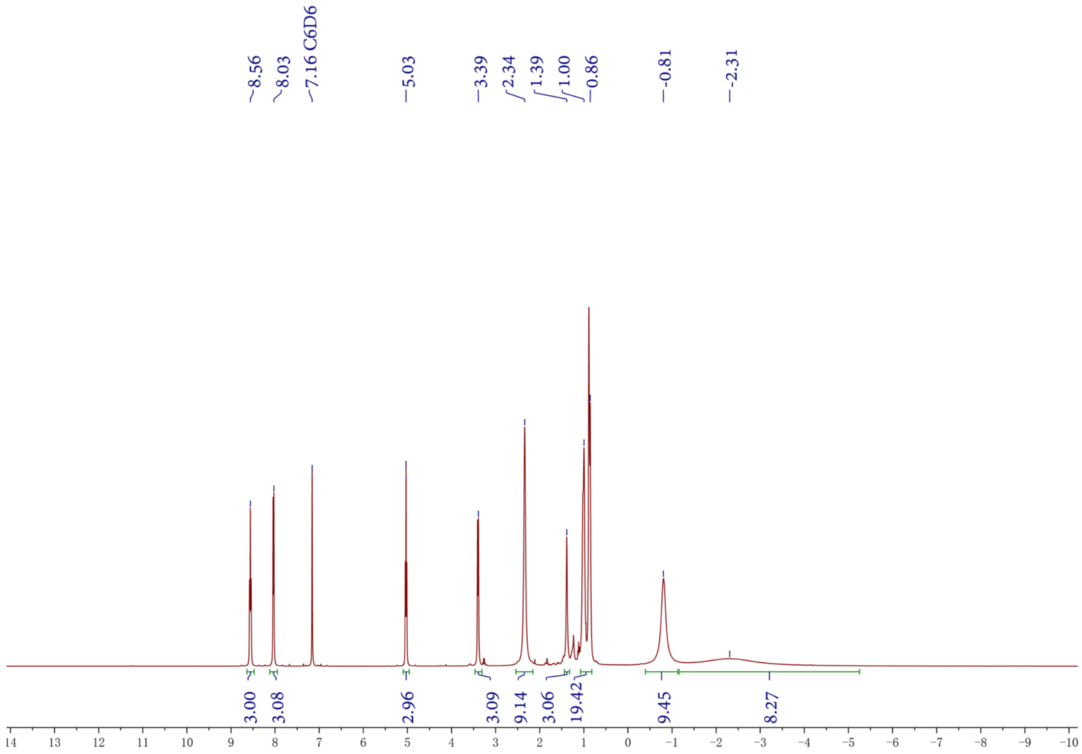

Supplementary Fig. 64. ^{1}H NMR (C_6D_6, 400 MHz, 298 K) spectrum of (^{Ad}TPBN$_3$)UI (**7**), δ, ppm: 8.56 (t, *J* = 7.5 Hz, 3H, C*H* of side rings), 8.03 (d, *J* = 7.3 Hz, 3H, C*H* of side rings), 5.03 (t, *J* = 7.3 Hz, 3H, C*H* of side rings), 3.39 (d, *J* = 8.1 Hz, 3H, C*H* of side rings), 2.34 (br s, FWHM = 17.0 Hz, 9H, Ad), 1.39 (s, 3H, C*H* of the anchor ring), 0.86–1.00 (m, 18H, Ad), –0.81 (br s, FWHM = 48.0 Hz, 9H, Ad), –2.31 (br s, FWHM = 520 Hz, 9H, Ad).

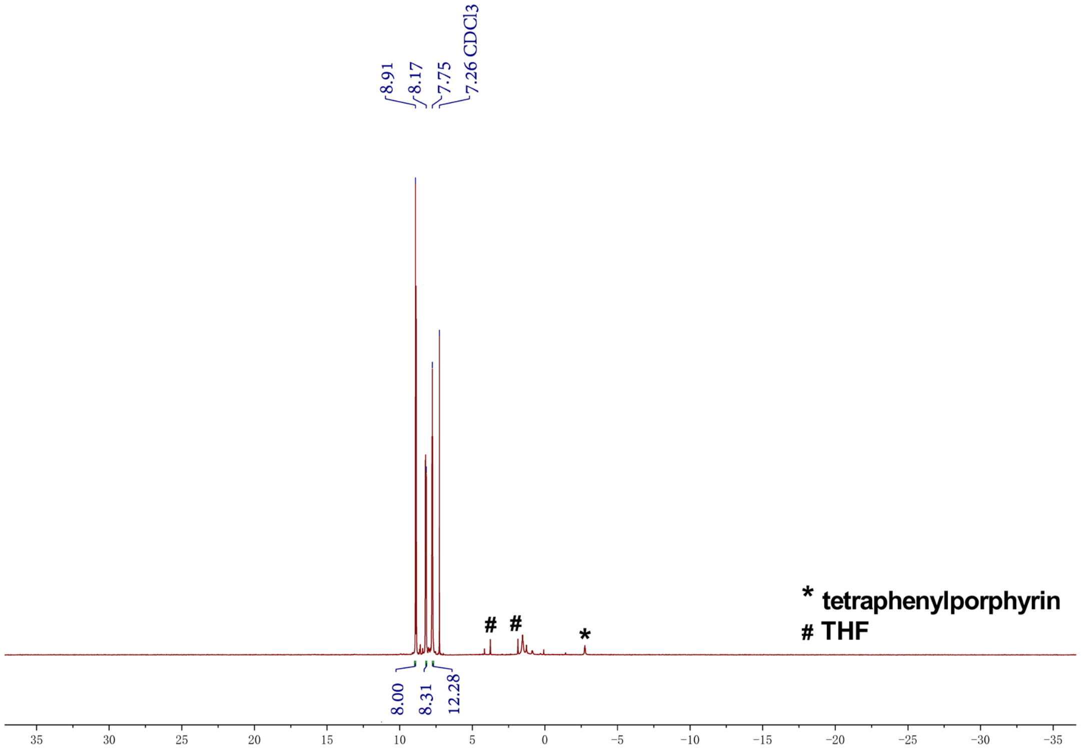

Supplementary Fig. 65. ^{1}H NMR ($CDCl_3$, 400 MHz, 298 K) spectrum of the in situ formed Co(TPP)(NO) (see Supplementary section 1.3 for experimental details), δ, ppm: 8.91 (s, 8H, C*H* of porphyrin), 8.17 (d, *J* = 5.9 Hz, 8H, ortho-C*H* of phenyl), 7.75 (m, 12H, meta- and para-C*H* of phenyl). A minor species was found to be neutral tetraphenylporphyrin[22].

6. UV-Vis-NIR Spectra

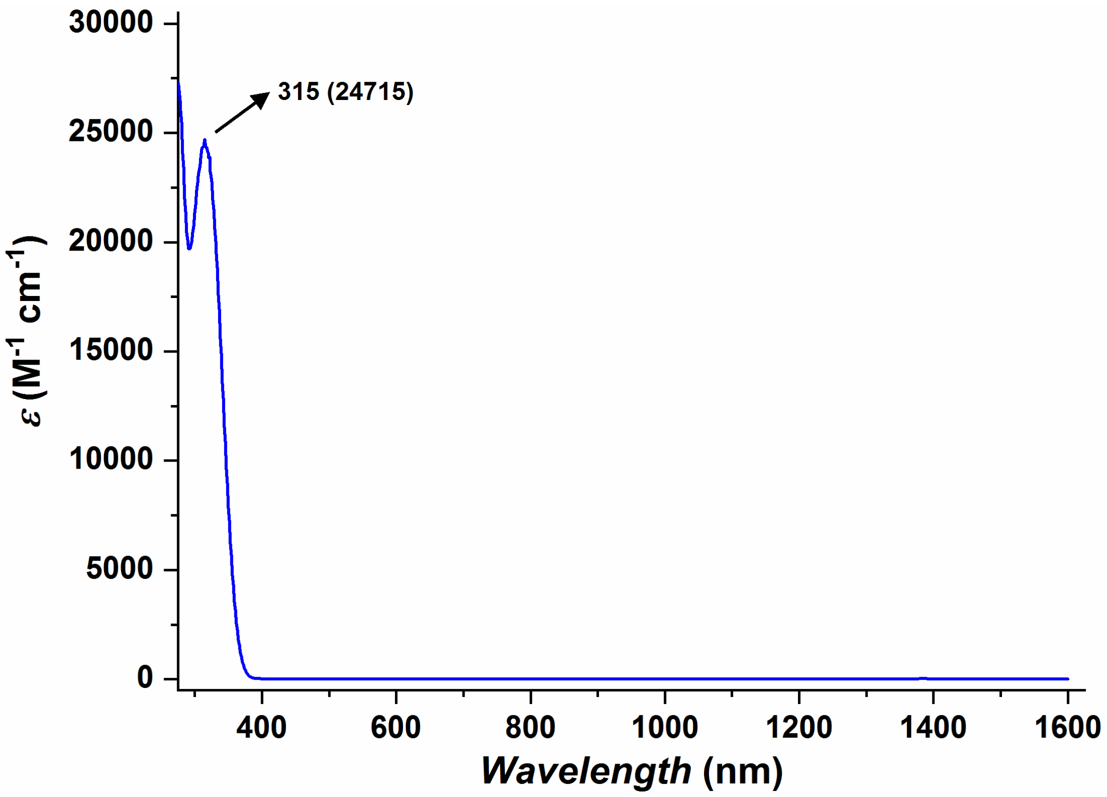

Supplementary Fig. 66. The UV-Vis-NIR spectrum (275–1600 nm) of $H_3(^{Ad}TPBN_3)$ in THF solution (1.5 mM) at room temperature. The solvent background was subtracted. λ_{max} / nm (ε / $M^{-1}\cdot cm^{-1}$): 315 (24715).

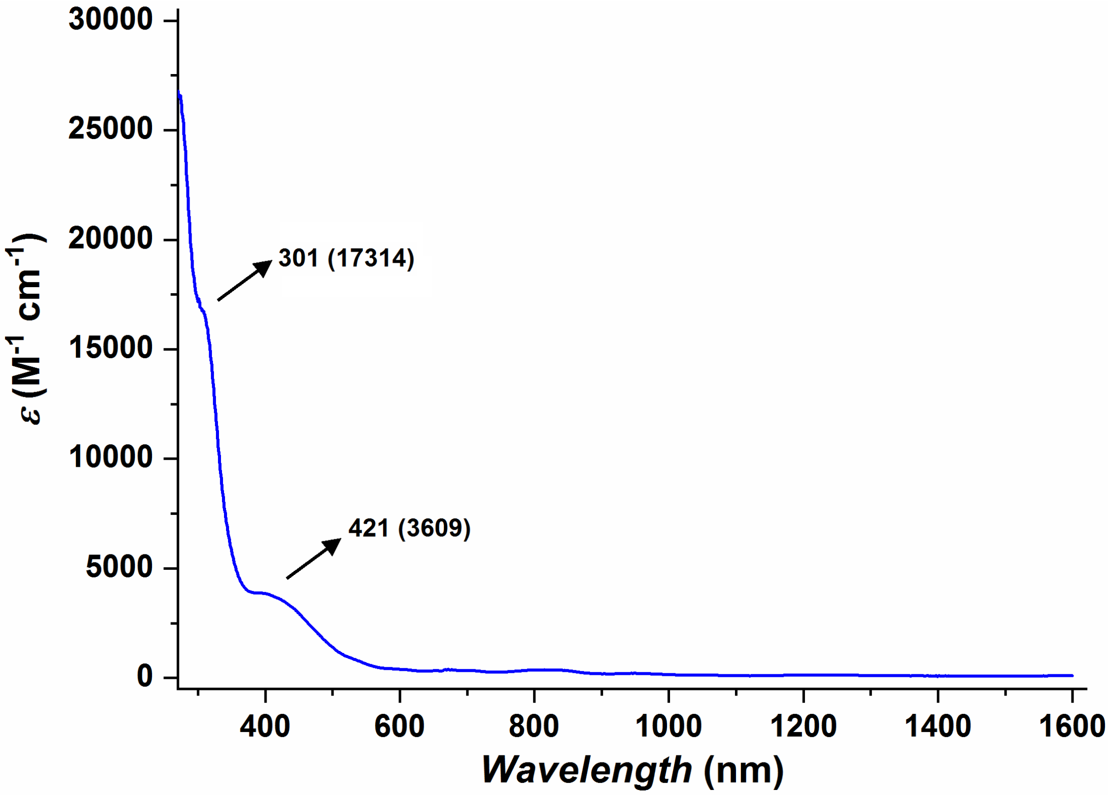

Supplementary Fig. 67. The UV-Vis-NIR spectrum (270–1600 nm) of (^{Ad}TPBN$_3$)U (**1**) in THF solution (1.5 mM) at room temperature. The solvent background was subtracted. λ_{max} / nm (ε / $M^{-1}\cdot cm^{-1}$): 301 (17314), 421 (3609).

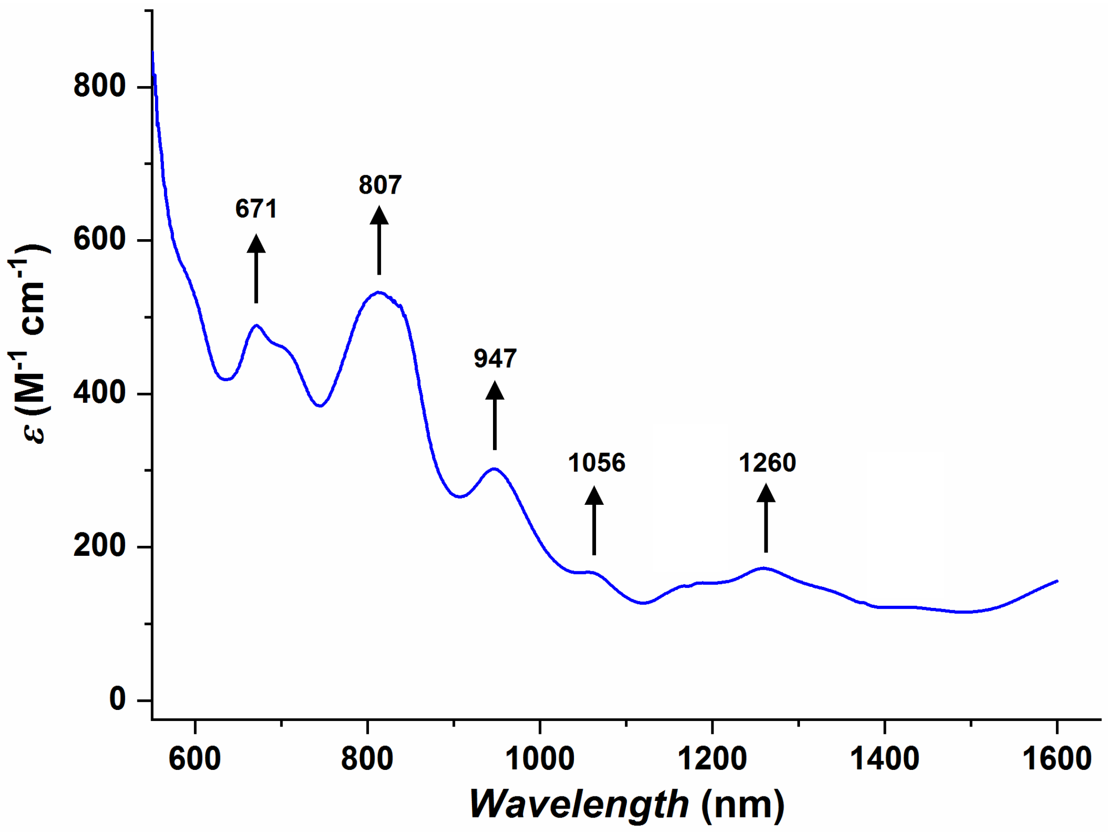

Supplementary Fig. 68. The 550–1600 nm region of the UV-Vis-NIR spectrum of (^{Ad}TPBN$_3$)U (**1**) in THF solution (5 mM) at room temperature. The solvent background was subtracted. λ_{max} / nm (ε / $M^{-1}\cdot cm^{-1}$): 671 (489), 807 (530), 947 (302), 1056 (167), 1260 (172).

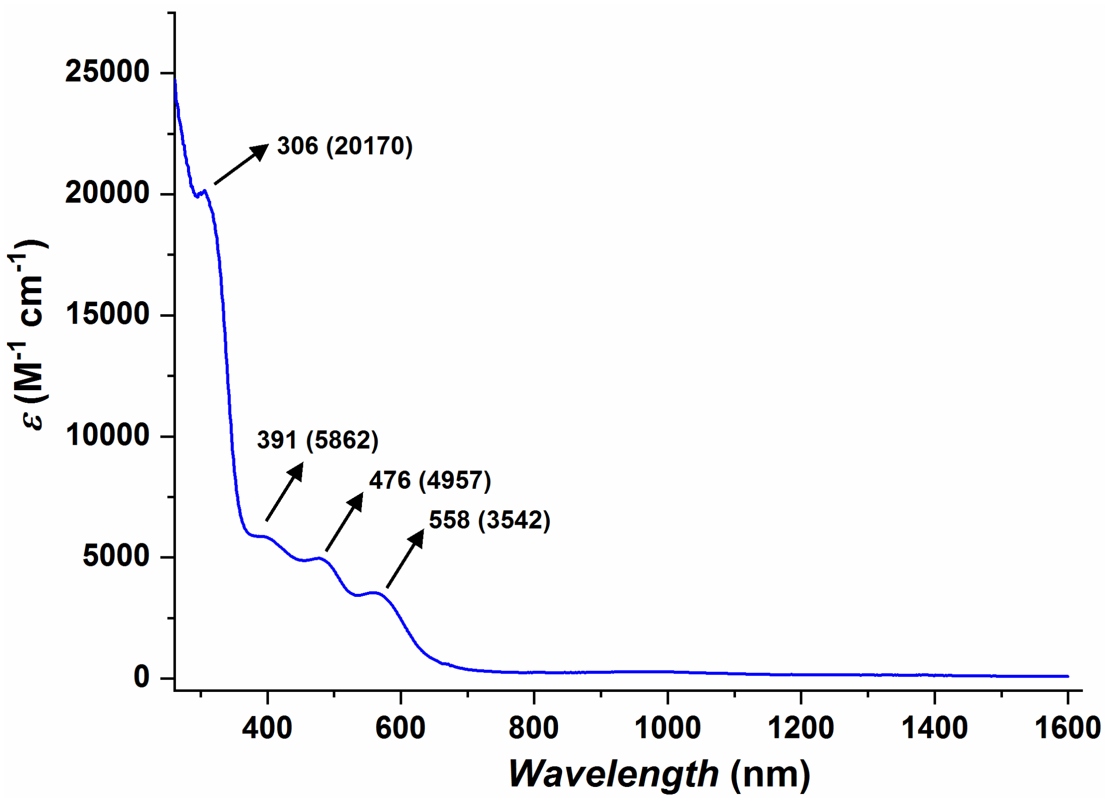

Supplementary Fig. 69. The UV-Vis-NIR spectrum (260–1600 nm) of [K(crypt)][(^{Ad}TPBN$_3$)U] (**2**) in THF solution (1.5 mM) at room temperature. The solvent background has been subtracted. λ_{max} / nm (ε / M^{-1}·cm^{-1}): 306 (20170), 391 (5862), 476 (4957), 558 (3542).

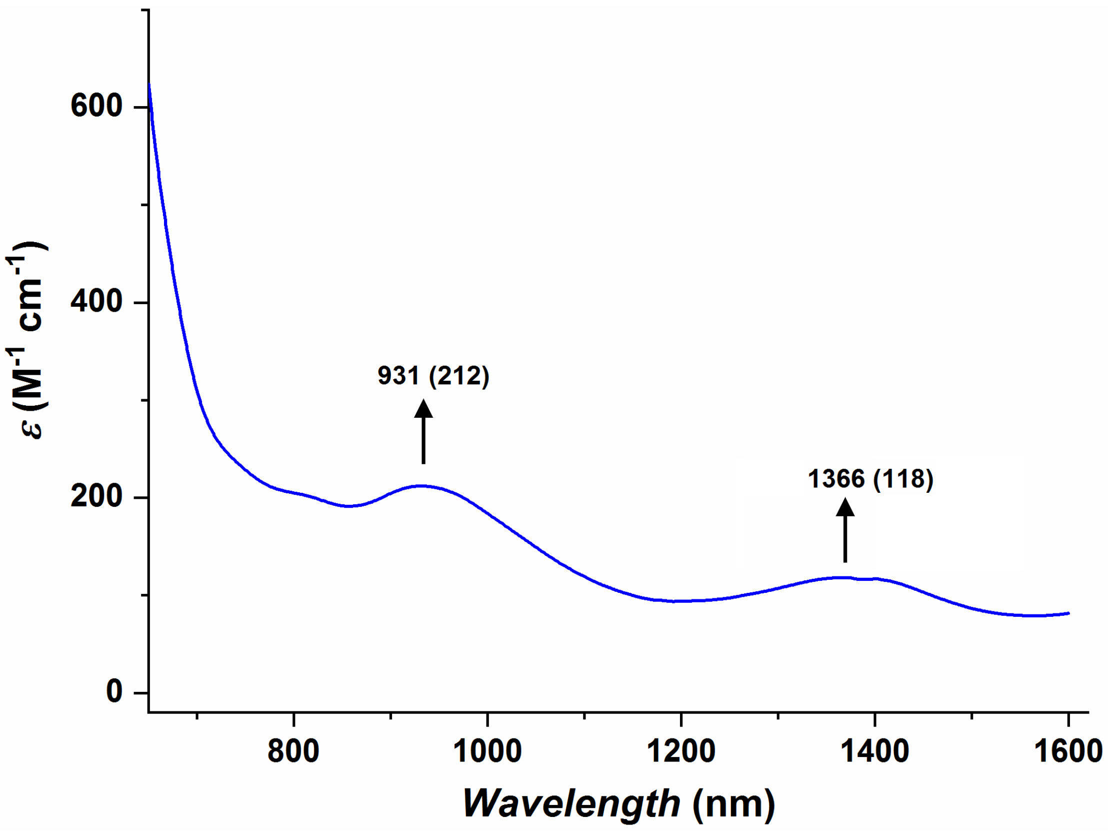

Supplementary Fig. 70. The 650–1600 nm region of the UV-Vis-NIR spectrum of [K(crypt)][(^{Ad}TPBN$_3$)U] (**2**) in THF solution (5 mM) at room temperature. The solvent background has been subtracted. λ_{max} / nm (ε / $M^{-1}\cdot cm^{-1}$): 931 (212), 1366 (118).

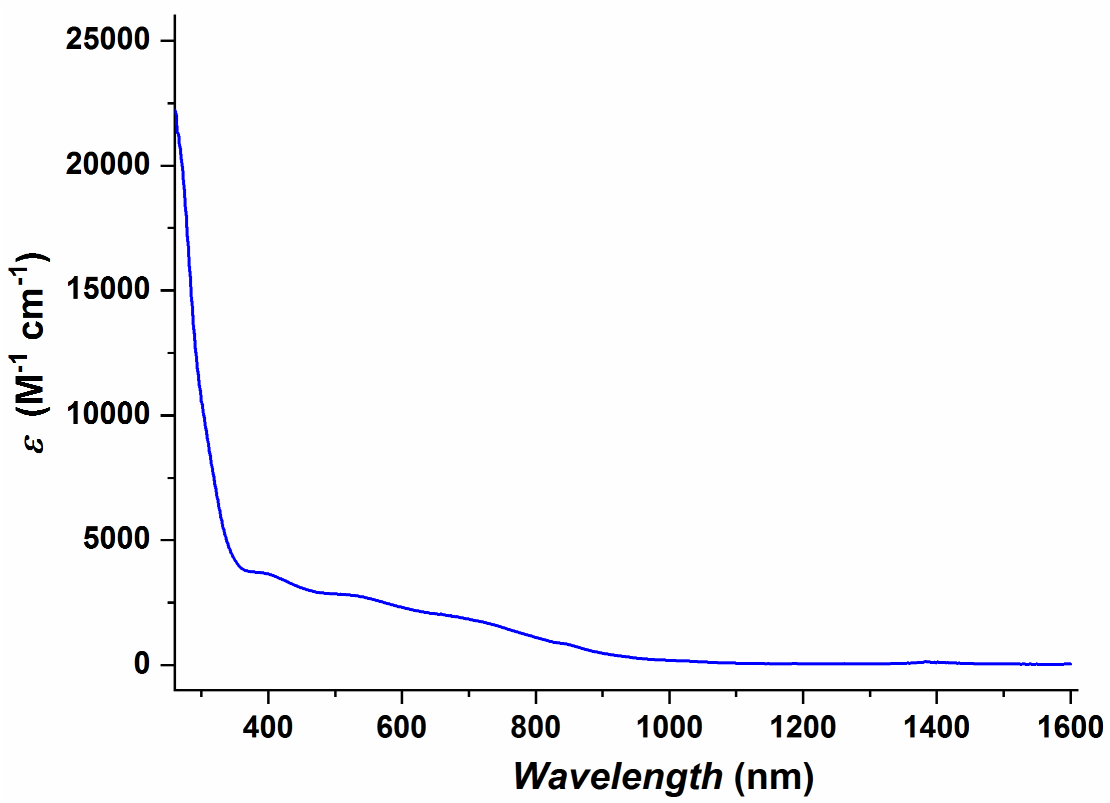

Supplementary Fig. 71. The UV-Vis-NIR spectrum (260–1600 nm) of (^{Ad}TPBN$_3$)UO (**3**) in THF solution (1.5 mM) at room temperature. The solvent background has been subtracted. Broad and intense bands over the range from 390 to 830 nm with ε of 1000–4000 $M^{-1}\cdot cm^{-1}$.

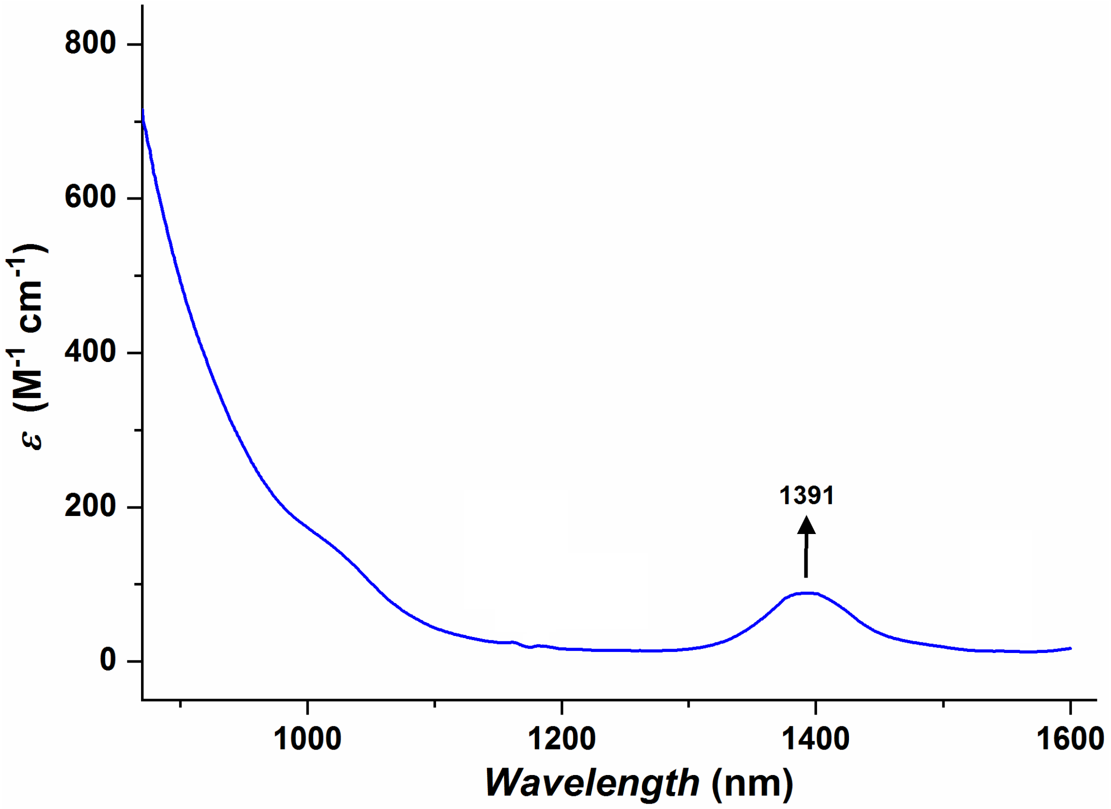

Supplementary Fig. 72. The 870–1600 nm region of the UV-Vis-NIR spectrum of ($^{Ad}TPBN_3$)UO (**3**) in THF solution (5 mM) at room temperature. The solvent background has been subtracted. λ_{max} / nm (ε / $M^{-1}\cdot cm^{-1}$): 1391 (88).

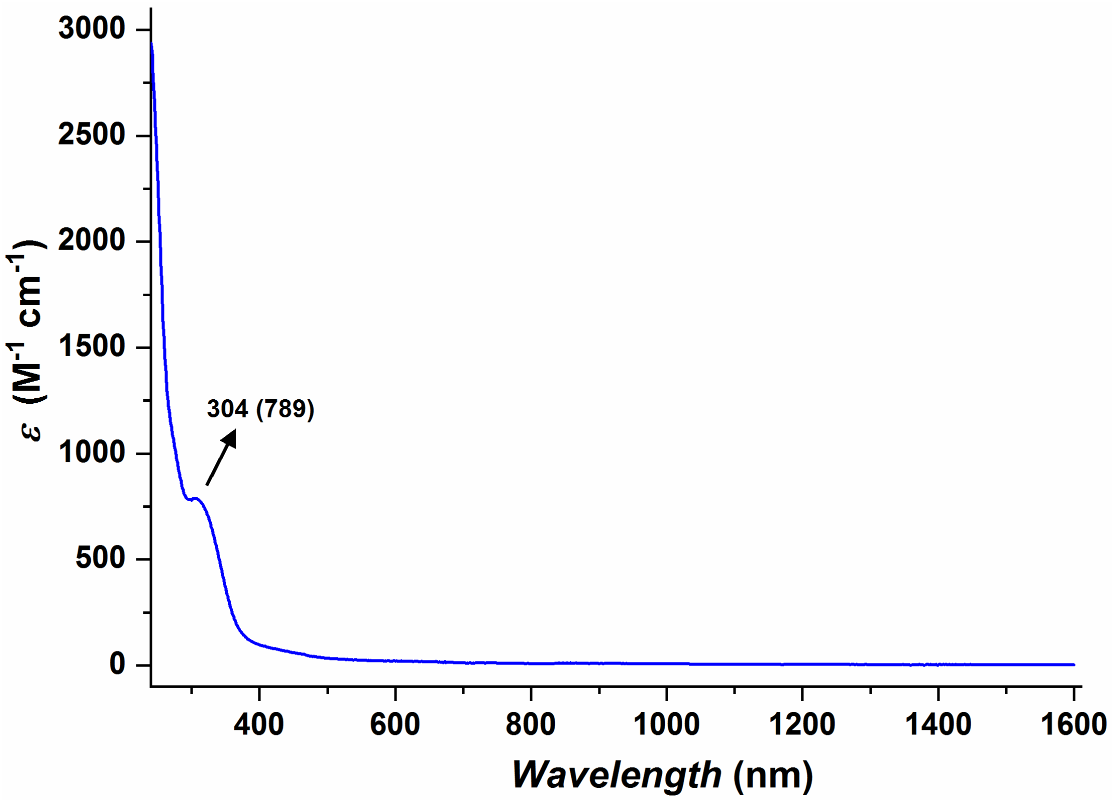

Supplementary Fig. 73. The UV-Vis-NIR spectrum (240–1600 nm) of [K(crypt)][(^{Ad}TPBN$_3$)UO] (**4**) in THF solution (ca. 1.5 mM, saturated) at room temperature. The solvent background has been subtracted. λ_{max} / nm (ε / $M^{-1}\cdot cm^{-1}$): 304 (789).

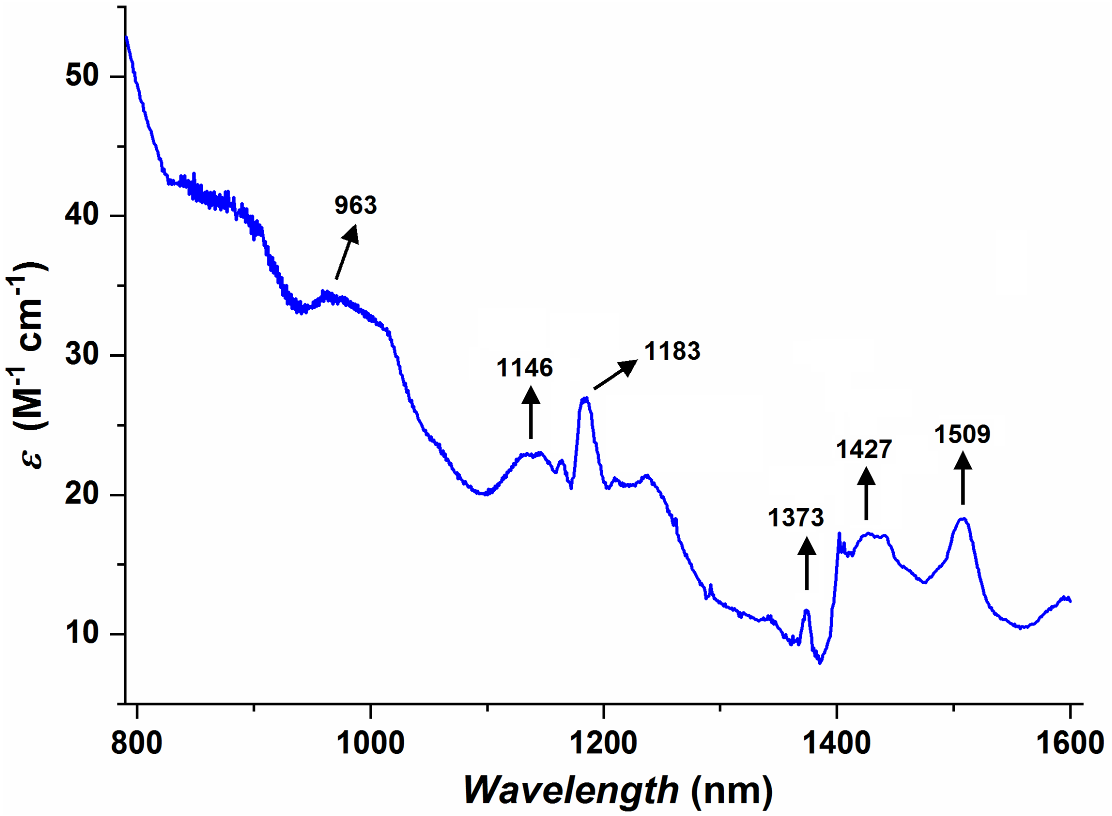

Supplementary Fig. 74. The 790–1600 nm region of the UV-Vis-NIR spectrum of [K(crypt)][(^{Ad}TPBN$_3$)UO] (**4**) in THF solution (ca. 1.5 mM, saturated) at room temperature. The solvent background has been subtracted. λ_{max} / nm (ε / $M^{-1}\cdot cm^{-1}$): 963 (35), 1146 (23), 1183 (27), 1373 (12), 1427 (17), 1509 (18).

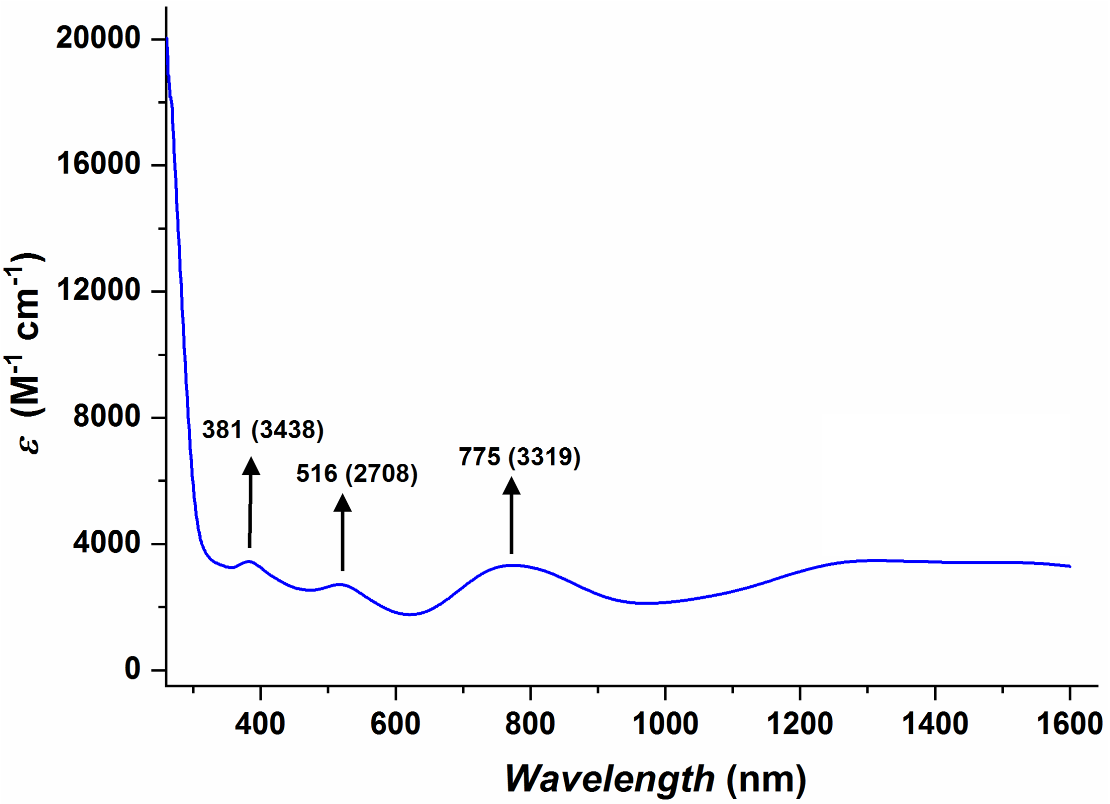

Supplementary Fig. 75. The UV-Vis-NIR spectrum (260–1600 nm) of [(^{Ad}TPBN$_3$)UO][SbF$_6$] (**5**) in CH_2Cl_2 solution (2.5 mM) at room temperature. The solvent background has been subtracted. λ_{max} / nm (ε / $M^{-1}\cdot cm^{-1}$): 381 (3438), 516 (2708), 775 (3319). Exceedingly strong absorption ($\varepsilon > 1500\ M^{-1}\cdot cm^{-1}$) over the entire range from 260 to 1600 nm, with no *f*–*f* transitions observed.

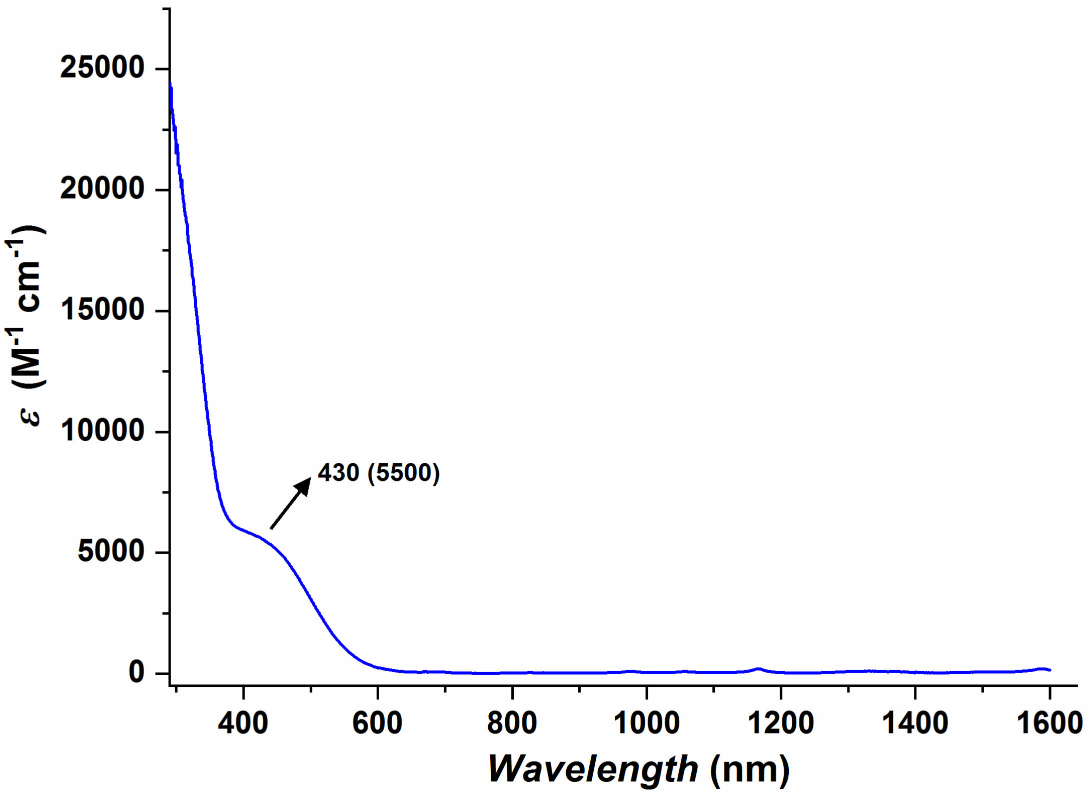

Supplementary Fig. 76. The UV-Vis-NIR spectrum (290–1600 nm) of (^{Ad}TPBN$_3$)UF (**6**) in THF solution (1.5 mM) at room temperature. The solvent background has been subtracted. λ_{max} / nm (ε / $M^{-1} \cdot cm^{-1}$): 430 (5500).

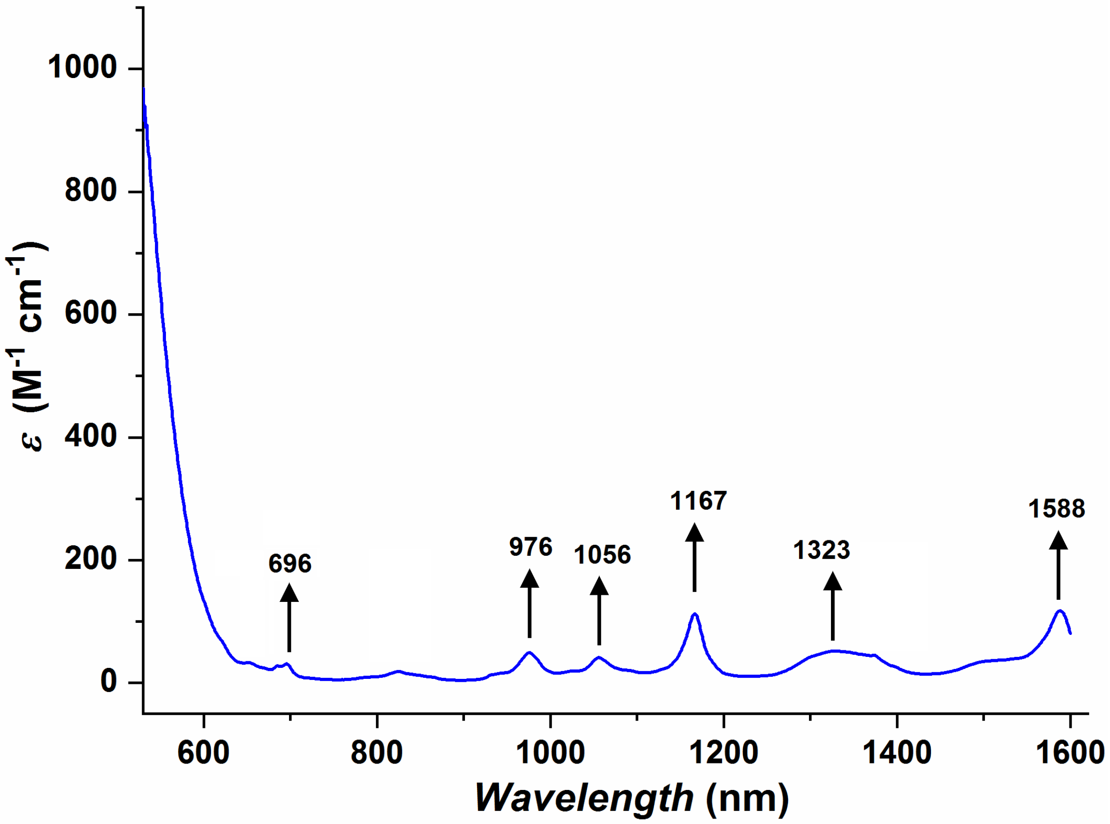

Supplementary Fig. 77. The 530–1600 nm region of the UV-Vis-NIR spectrum of (^{Ad}TPBN$_3$)UF (**6**) in THF solution (5 mM) at room temperature. The solvent background has been subtracted. λ_{max} / nm (ε / $M^{-1}\cdot cm^{-1}$): 696 (31), 976 (49), 1056 (41), 1167 (113), 1323 (52), 1588 (158).

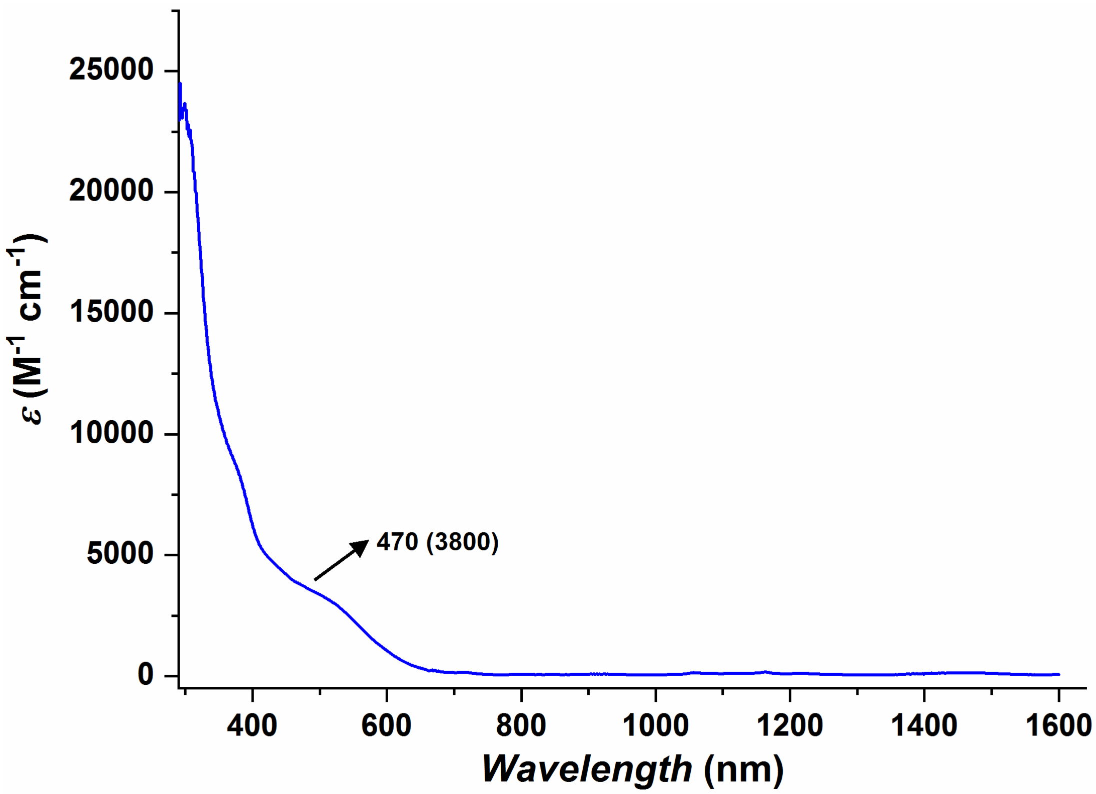

Supplementary Fig. 78. The UV-Vis-NIR spectrum (290–1600 nm) of (^{Ad}TPBN$_3$)UI (**7**) in THF solution (1.5 mM) at room temperature. The solvent background was subtracted. λ_{max} / nm (ε / $M^{-1}\cdot cm^{-1}$): 470 (3800).

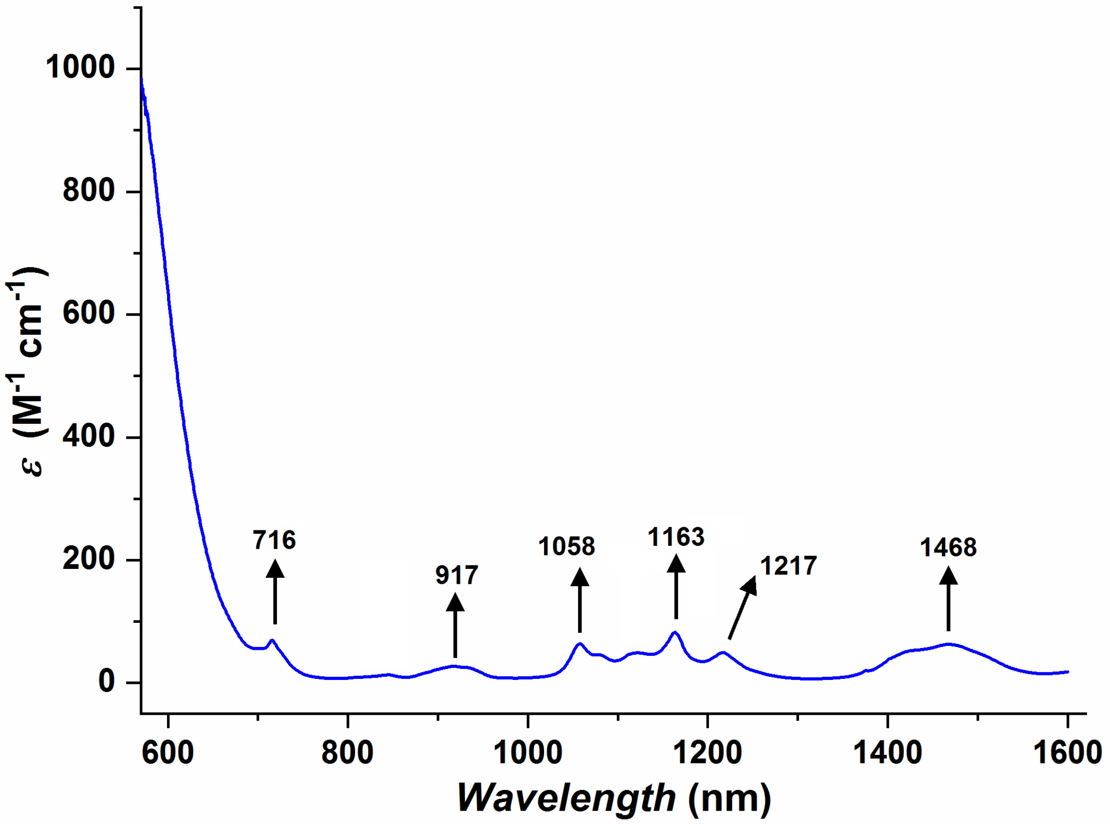

Supplementary Fig. 79. The 570–1600 nm region of the UV-Vis-NIR spectrum of (^{Ad}TPBN$_3$)UI (**7**) in THF solution (5 mM) at room temperature. The solvent background has been subtracted. λ_{max} / nm (ε / $M^{-1}\cdot cm^{-1}$): 716 (69), 917 (27), 1058 (64), 1163 (82), 1217 (49), 1468 (62).

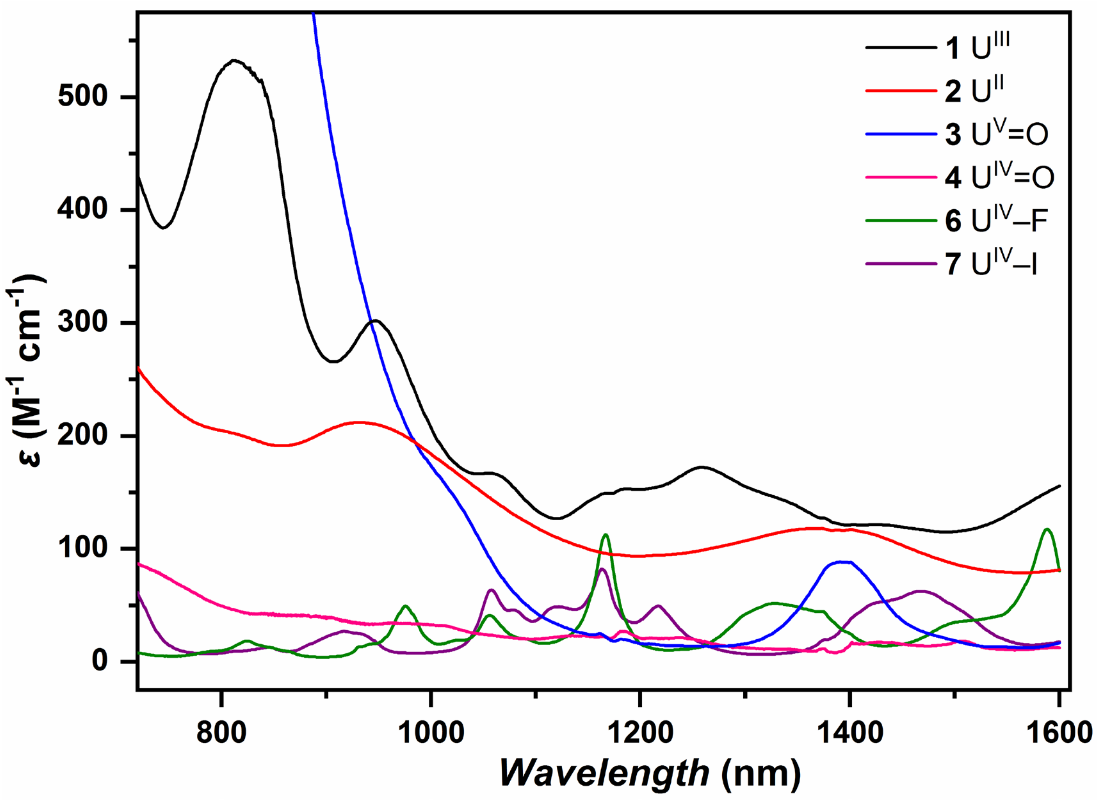

Supplementary Fig. 80. The NIR region (720–1600 nm) of the UV-Vis-NIR spectra of (^{Ad}TPBN$_3$)U (**1**), [K(crypt)][(^{Ad}TPBN$_3$)U] (**2**), (^{Ad}TPBN$_3$)UO (**3**), [K(crypt)][(^{Ad}TPBN$_3$)UO] (**4**), (^{Ad}TPBN$_3$)UF (**6**), and (^{Ad}TPBN$_3$)UI (**7**) in THF solution (ca. 1.5 mM for **4** and 5 mM for others) at room temperature. The solvent background has been subtracted. The uranium(VI) complex [(^{Ad}TPBN$_3$)UO][SbF$_6$] (**5**) absorbs too strongly in the NIR region to be depicted together with other complexes.

7. X-ray Photoelectron Spectroscopy (XPS)

Supplementary Table 6. Summary of the binding energies of 4*f* electrons of uranium for compounds **1**–**7**.

**Compounds**	**U oxidation states**	**U 4$f_{5/2}$** (eV)	**U 4$f_{7/2}$** (eV)
[K(crypt)][(^{Ad}TPBN$_3$)U] (**2**)	II	391.5 eV	380.3 eV
(^{Ad}TPBN$_3$)U (**1**)	III	391.6 eV	380.4 eV
(^{Ad}TPBN$_3$)UI (**7**)	IV	391.6 eV	380.5 eV
(^{Ad}TPBN$_3$)UF (**6**)	IV	391.6 eV	380.7 eV
[K(crypt)][(^{Ad}TPBN$_3$)UO] (**4**)	IV	392.3 eV	381.4 eV
(^{Ad}TPBN$_3$)UO (**3**)	V	392.8 eV	381.9 eV
[(^{Ad}TPBN$_3$)UO][SbF$_6$] (**5**)	VI	392.9 eV	382.0 eV

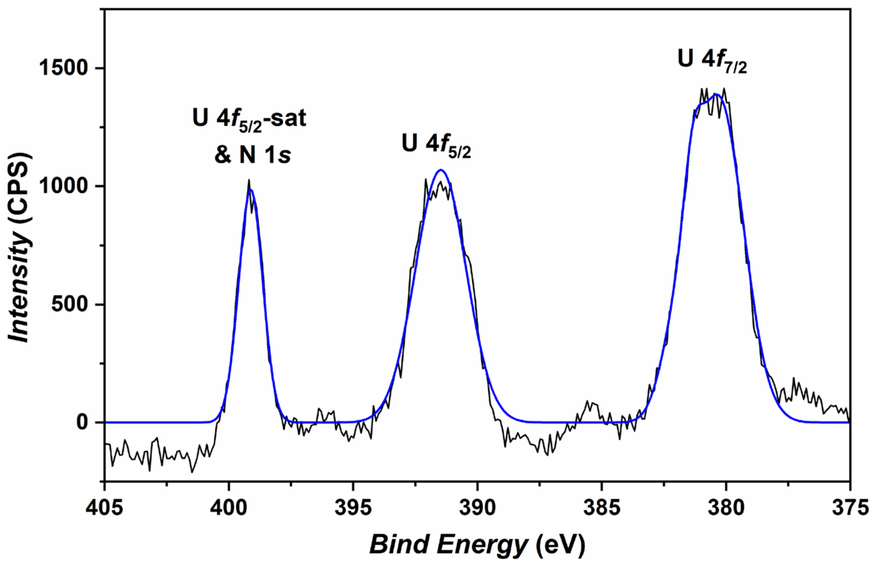

Supplementary Fig. 81. The Shirley background-subtracted XPS spectrum (black) and the fitting curve (blue) of [K(crypt)][(^{Ad}TPBN$_3$)U] (**2**). Binding energies: U 4$f_{5/2}$, 391.5 eV; U 4$f_{7/2}$, 380.3 eV. CPS: counts per second; sat: satellite.

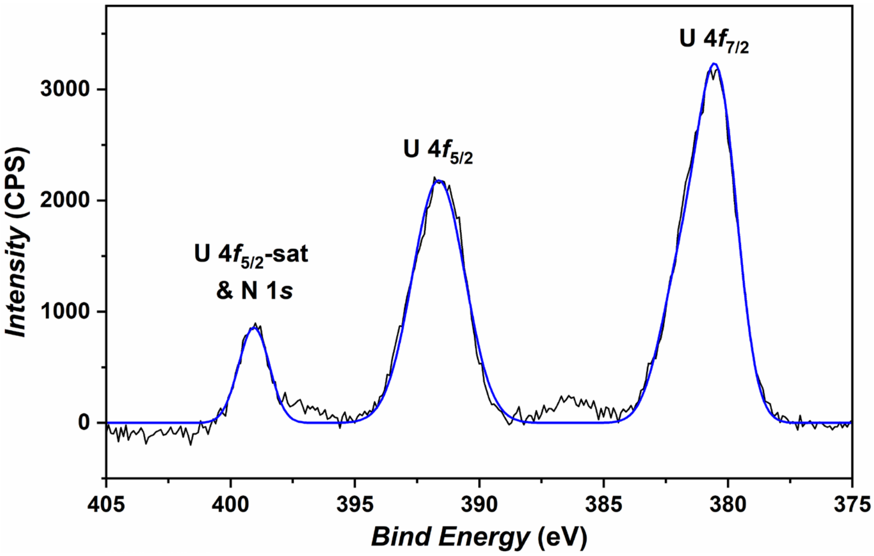

Supplementary Fig. 82. The Shirley background-subtracted XPS spectrum (black) and the fitting curve (blue) of (^{Ad}TPBN$_3$)U (**1**). Binding energies: U 4*f*$_{5/2}$, 391.6 eV; U 4*f*$_{7/2}$, 380.4 eV.

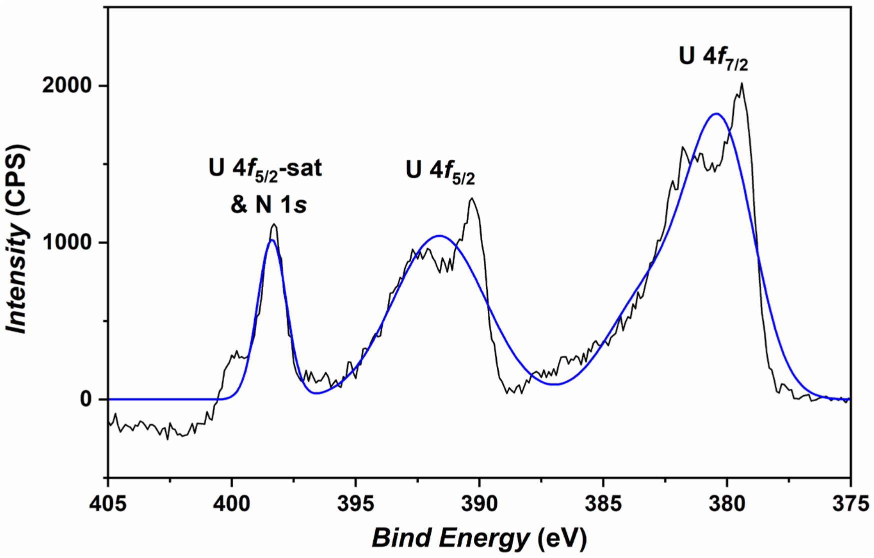

Supplementary Fig. 83. The Shirley background-subtracted XPS spectrum (black) and the fitting curve (blue) of (^{Ad}TPBN$_3$)UI (**7**). Binding energies: U 4$f_{5/2}$, 391.6 eV; U 4$f_{7/2}$, 380.5 eV.

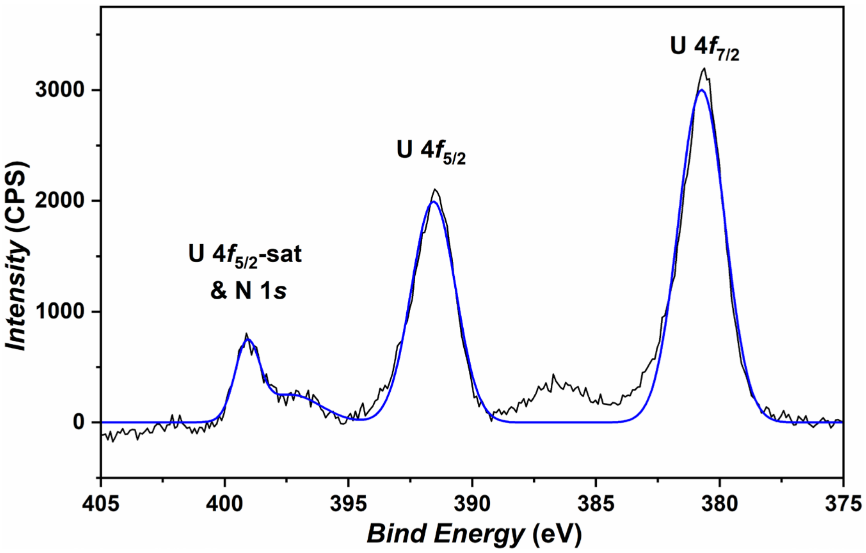

Supplementary Fig. 84. The Shirley background-subtracted XPS spectrum (black) and the fitting curve (blue) of $(^{Ad}TPBN_3)UF$ (**6**). Binding energies: U 4$f_{5/2}$, 391.6 eV; U 4$f_{7/2}$, 380.7 eV.

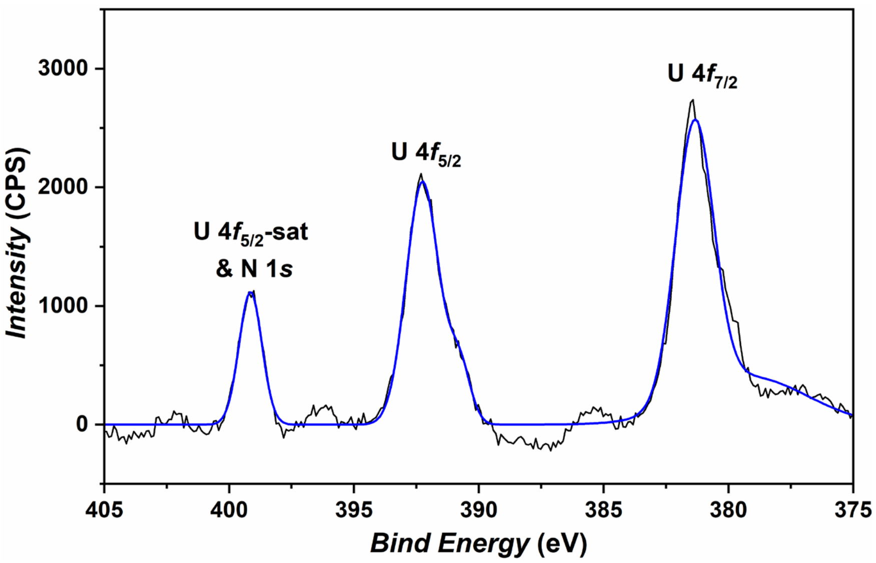

Supplementary Fig. 85. The Shirley background-subtracted XPS spectrum (black) and the fitting curve (blue) of [K(crypt)][(^{Ad}TPBN$_3$)UO] (**4**). Binding energies: U 4$f_{5/2}$, 392.3 eV; U 4$f_{7/2}$, 381.4 eV.

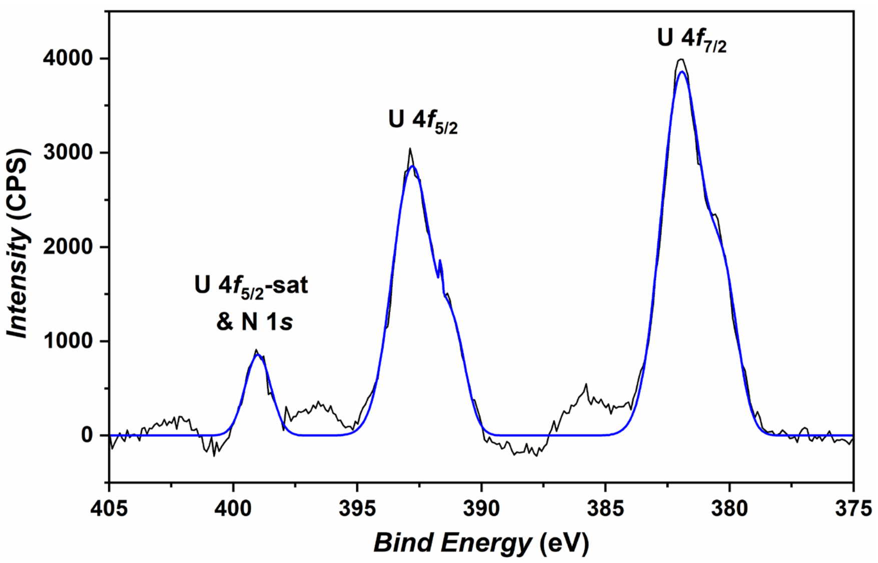

Supplementary Fig. 86. The Shirley background-subtracted XPS spectrum (black) and the fitting curve (blue) of (^{Ad}TPBN$_3$)UO (**3**). Binding energies: U 4$f_{5/2}$, 392.8 eV; U 4$f_{7/2}$, 381.9 eV.

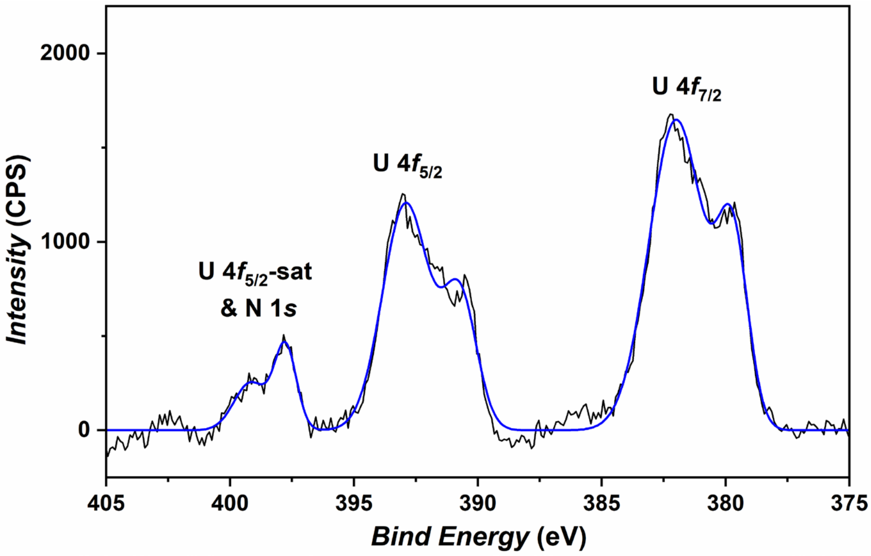

Supplementary Fig. 87. The Shirley background-subtracted XPS spectrum (black) and the fitting curve (blue) of [(^{Ad}TPBN$_3$)UO][SbF$_6$] (**5**). The peak shape is complex probably due to the final state effect of the closed-shell uranium(VI). Binding energies: U 4$f_{5/2}$, 392.9 eV; U 4$f_{7/2}$, 382.0 eV.

8. SQUID Measurement

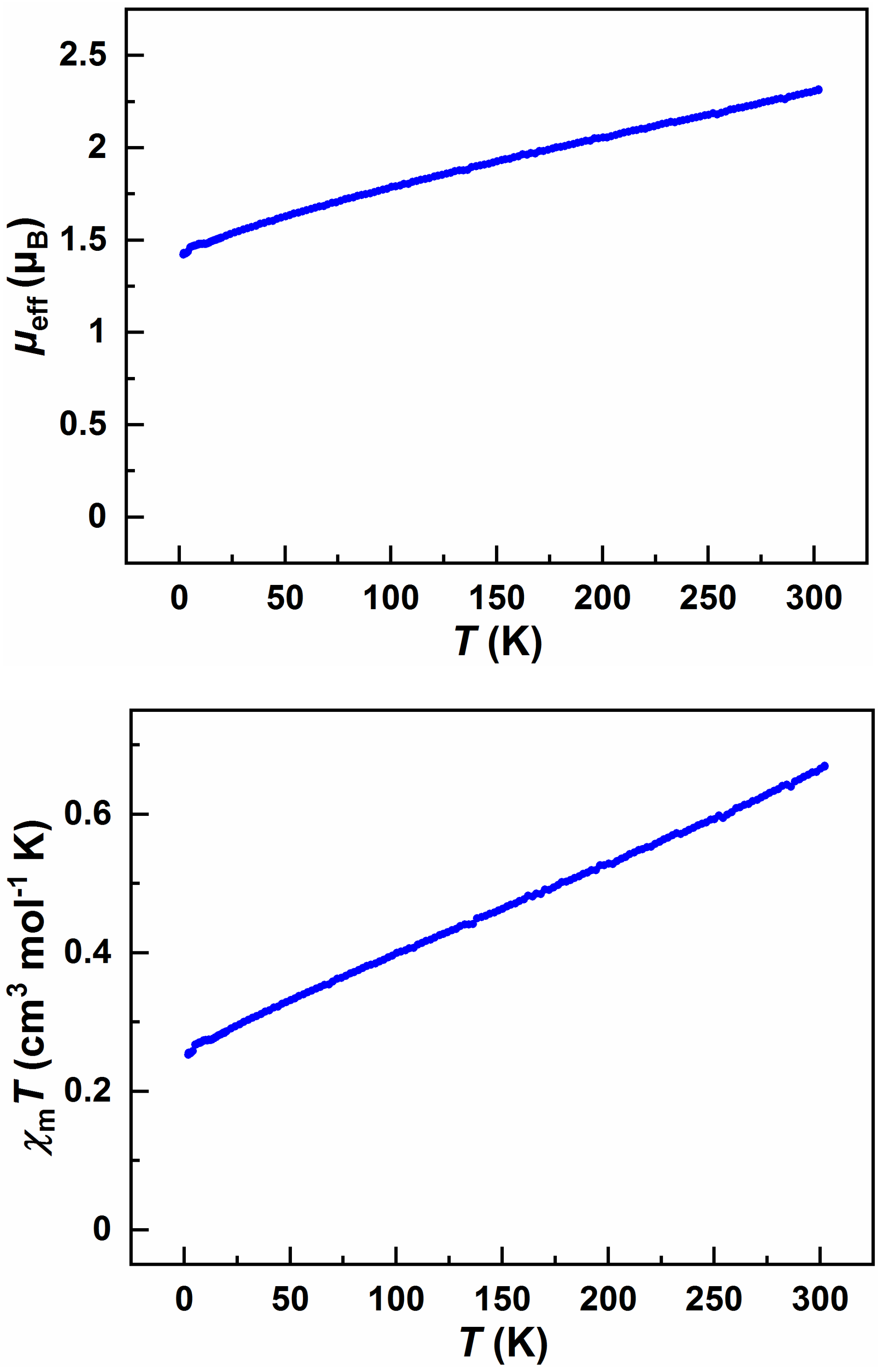

Supplementary Fig. 88. The temperature dependent magnetic susceptibility data (top: μ_{eff} vs T; bottom: $\chi_m T$ vs T) of a powdered sample of (^{Ad}TPBN$_3$)U (**1**) measured under 1 kOe DC field (2–298 K). μ_{eff} (298 K) = 2.30 μ_B, μ_{eff} (2 K) = 1.42 μ_B; $\chi_m T$ (298 K) = 0.661 cm^3·mol^{-1}·K, $\chi_m T$ (2 K) = 0.253 cm^3·mol^{-1}·K.

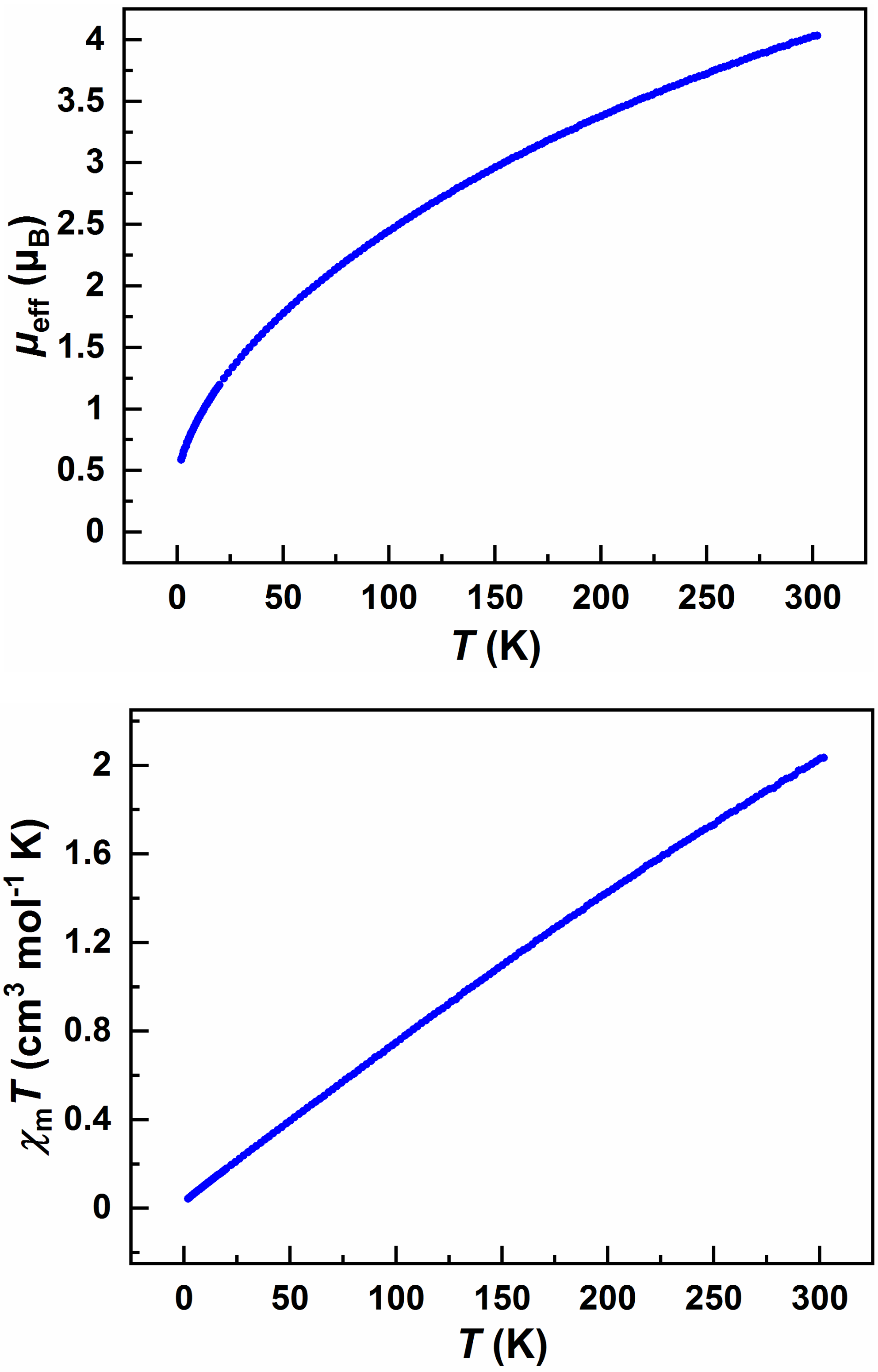

Supplementary Fig. 89. The temperature dependent magnetic susceptibility data (top: μ_{eff} vs T; bottom: $\chi_m T$ vs T) of a powdered sample of [K(crypt)][(^{Ad}TPBN$_3$)U] (**2**) measured under 1 kOe DC field (2–298 K). μ_{eff} (298 K) = 4.02 μ_B, μ_{eff} (2 K) = 0.59 μ_B; $\chi_m T$ (298 K) = 2.017 cm^3·mol^{-1}·K, $\chi_m T$ (2 K) = 0.043 cm^3·mol^{-1}·K.

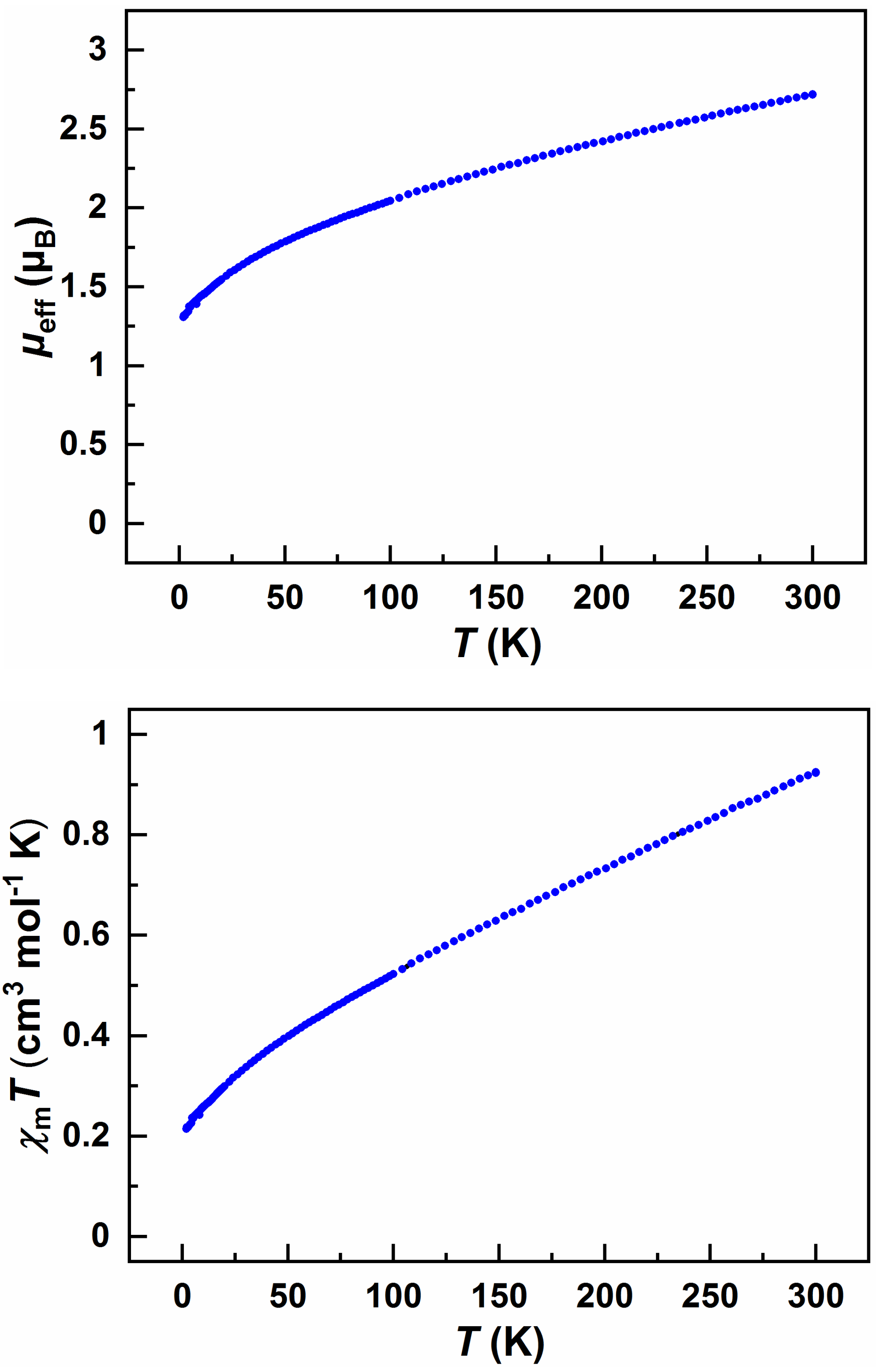

Supplementary Fig. 90. The temperature dependent magnetic susceptibility data (top: μ_{eff} vs T; bottom: $\chi_m T$ vs T) of a powdered sample of (^{Ad}TPBN$_3$)UO (**3**) measured under 1 kOe DC field (2–298 K). μ_{eff} (298 K) = 2.71 μ_B, μ_{eff} (2 K) = 1.31 μ_B; $\chi_m T$ (298 K) = 0.921 cm^3·mol^{-1}·K, $\chi_m T$ (2 K) = 0.214 cm^3·mol^{-1}·K.

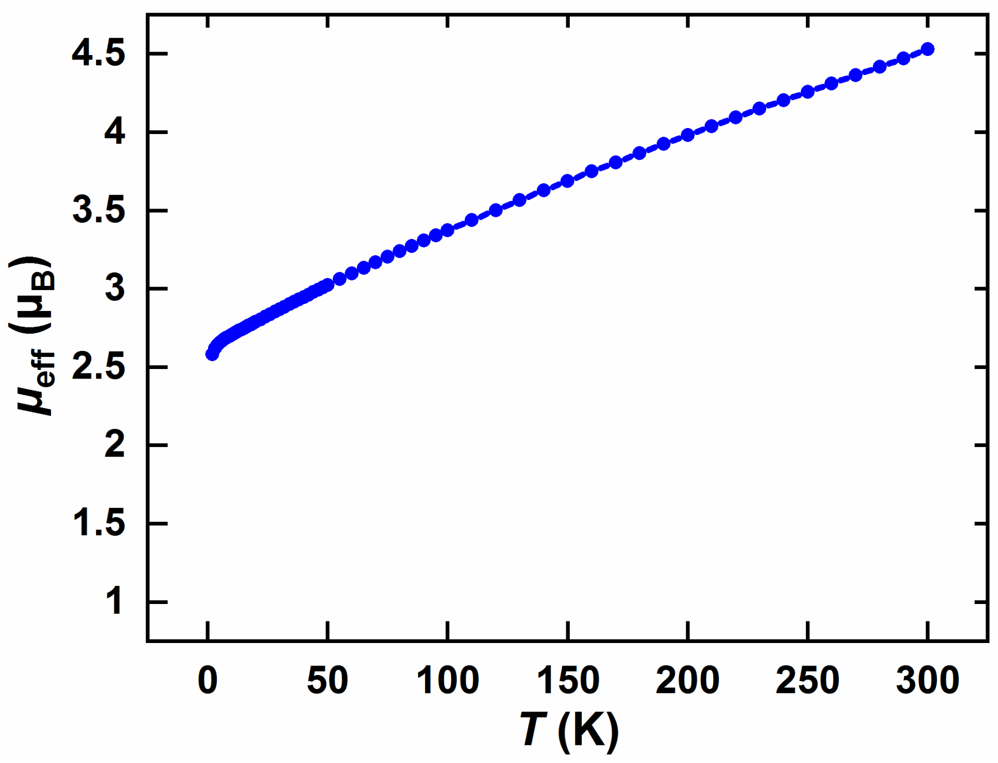

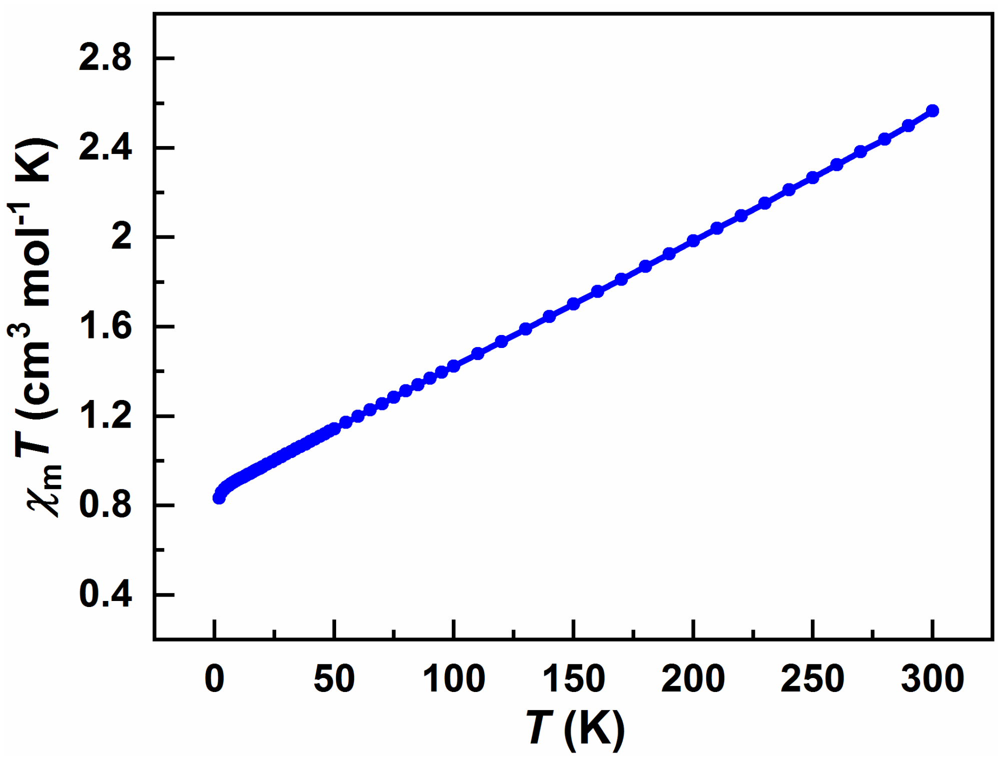

Supplementary Fig. 91. The temperature dependent magnetic susceptibility data (top: μ_{eff} vs T; bottom: $\chi_m T$ vs T) of a powdered sample of [K(crypt)][(^{Ad}TPBN$_3$)UO] (**4**) measured under 1 kOe DC field (2–300 K). μ_{eff} (300 K) = 4.53 μ_B, μ_{eff} (2 K) = 2.58 μ_B; $\chi_m T$ (300 K) = 2.565 cm^3·mol^{-1}·K, $\chi_m T$ (2 K) = 0.835 cm^3·mol^{-1}·K.

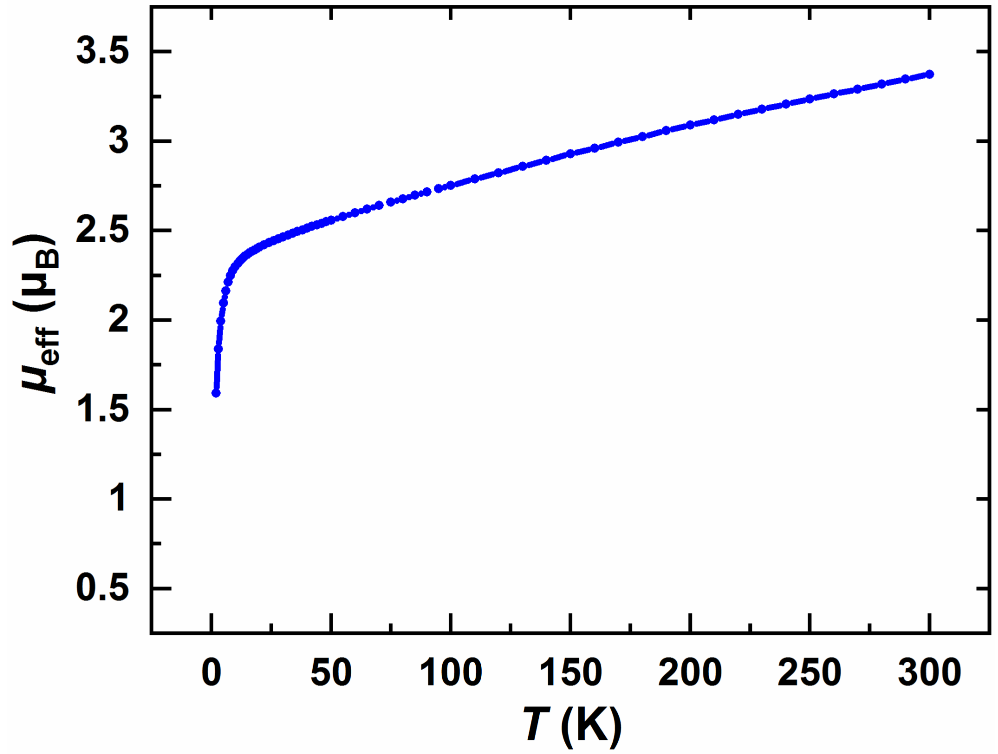

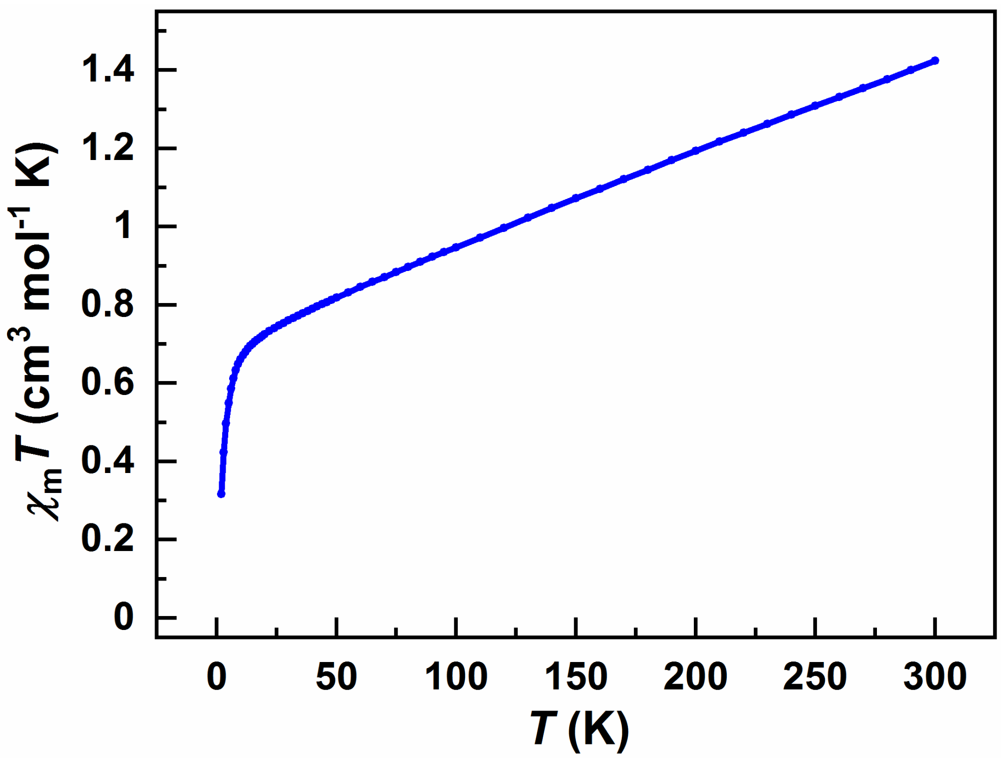

Supplementary Fig. 92. The temperature dependent magnetic susceptibility data (top: μ_{eff} vs T; bottom: $\chi_{\mathrm{m}}T$ vs T) of a powdered sample of (^{Ad}TPBN$_3$)UF (**6**) measured under 1 kOe DC field (2–300 K). μ_{eff} (300 K) = 3.37 μ_B, μ_{eff} (2 K) = 1.59 μ_B; $\chi_{\mathrm{m}}T$ (300 K) = 1.424 cm^3·mol^{-1}·K, $\chi_{\mathrm{m}}T$ (2 K) = 0.317 cm^3·mol^{-1}·K.

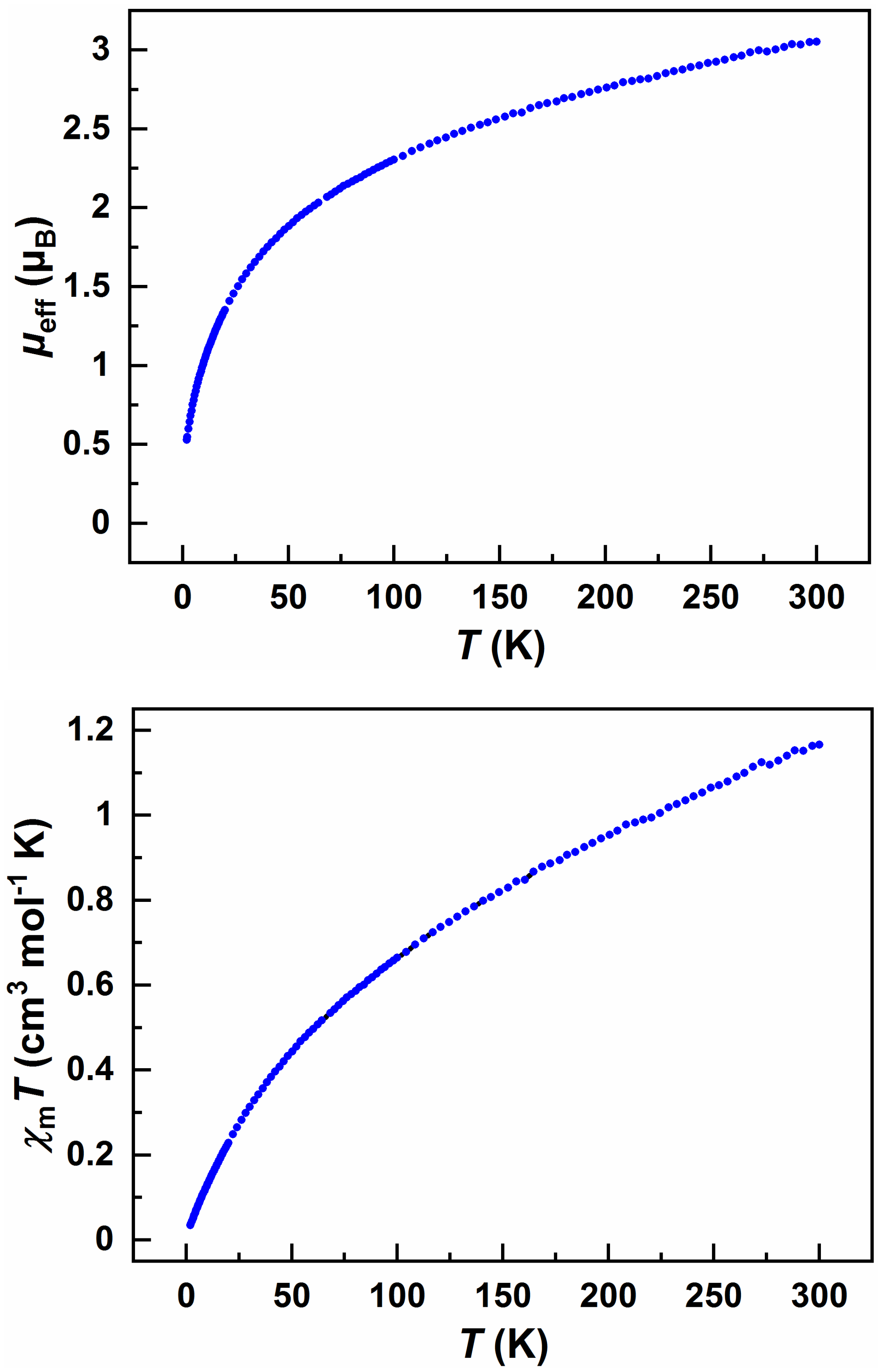

Supplementary Fig. 93. The temperature dependent magnetic susceptibility data (top: μ_{eff} vs T; bottom: $\chi_m T$ vs T) of a powdered sample of (^{Ad}TPBN$_3$)UI (**7**) measured under 1 kOe DC field (2–298 K). μ_{eff} (298 K) = 3.05 μ_B, μ_{eff} (2 K) = 0.53 μ_B; $\chi_m T$ (298 K) = 1.164 cm^3·mol^{-1}·K, $\chi_m T$ (2 K) = 0.035 cm^3·mol^{-1}·K.

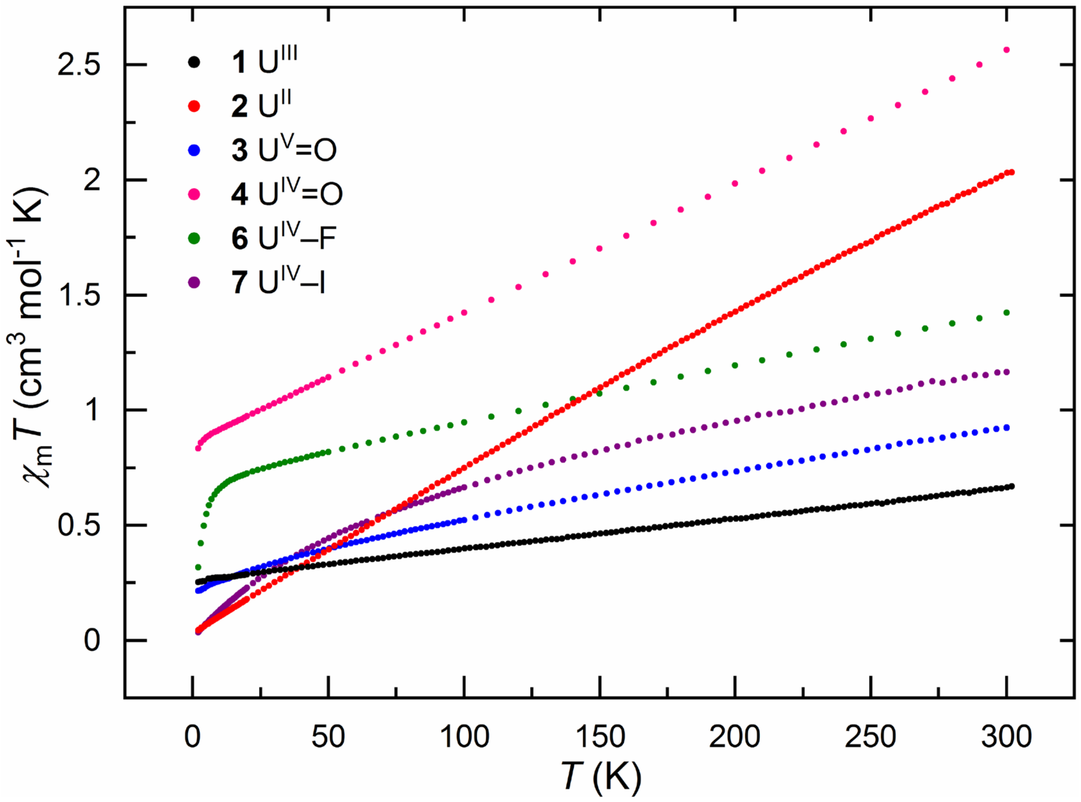

Supplementary Fig. 94. The temperature dependent magnetic susceptibility data ($\chi_m T$ vs T) of powdered samples of (^{Ad}TPBN$_3$)U (**1**), [K(crypt)][(^{Ad}TPBN$_3$)U] (**2**), (^{Ad}TPBN$_3$)UO (**3**), [K(crypt)][(^{Ad}TPBN$_3$)UO] (**4**), (^{Ad}TPBN$_3$)UF (**6**), and (^{Ad}TPBN$_3$)UI (**7**) measured under 1 kOe DC field (2–298 K or 2–300 K).

9. EPR Spectra

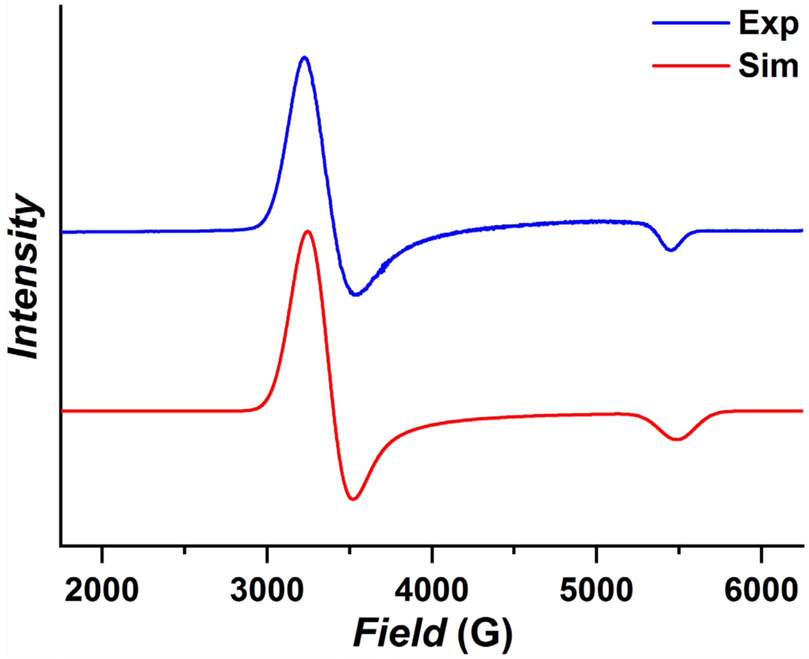

Supplementary Fig. 95. Experimental (top, blue) and simulated (bottom, red) CW-EPR spectra of (^{Ad}TPBN$_3$)U (**1**) as a frozen toluene solution (10 mM) measured at 10 K. Spectrometer parameters: v = 9.385 GHz, P = 2.377 mW, and MA (modulation amplitude) = 4.720 G. Simulation parameters: $g_{\perp}$ = 1.98 (g_x), 2.07 (g_y), $g_{\parallel}$ = 1.22 (g_z).

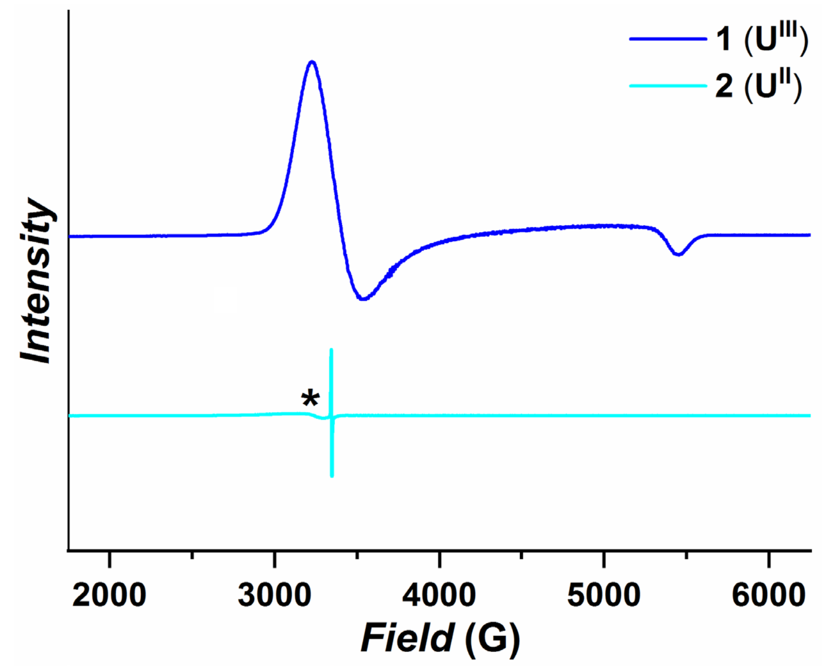

Supplementary Fig. 96. Experimental CW-EPR spectra of (^{Ad}TPBN$_3$)U (**1**) (top, blue) and [K(crypt)][(^{Ad}TPBN$_3$)U] (**2**) (bottom, cyan) as frozen solutions (10 mM) of toluene (for **1**) and THF (for **2**) measured at 10 K. Spectrometer parameters: v = 9.382 GHz, P = 2.377 mW, and MA = 2.000 G. The cavity signal (background) is labelled with an asterisk (*). For **2**, only a small signal of the free radical impurity or the residual solvated electrons was observed at g = 2.00[23,24], which is in line with an EPR-silent $5f^4$ electronic configuration for uranium(II).

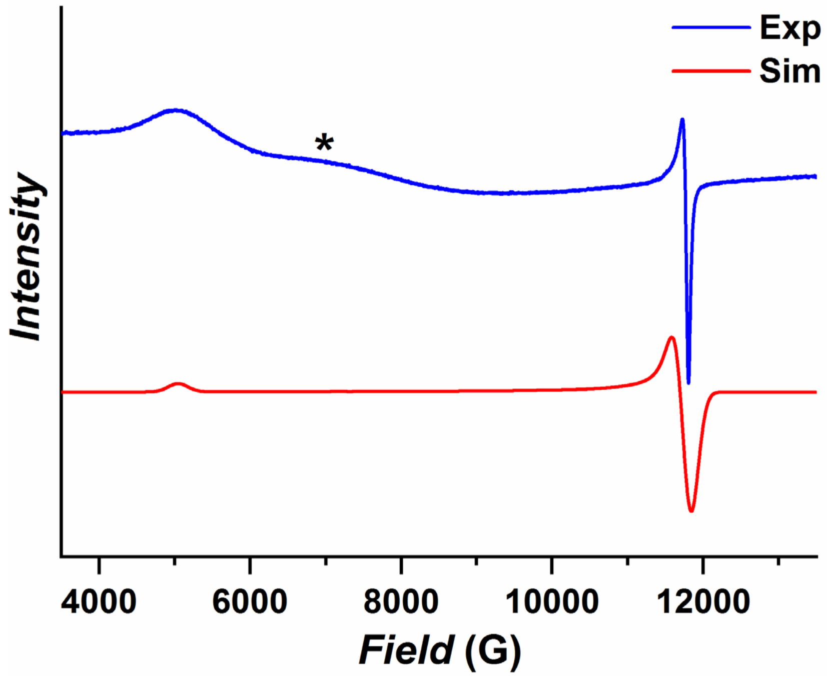

Supplementary Fig. 97. Experimental (top, blue) and simulated (bottom, red) CW-EPR spectra of (^{Ad}TPBN$_3$)UO (**3**) as a powder sample measured at 10 K. Spectrometer parameters: v = 9.389 GHz, P = 9.464 mW, and MA = 4.720 G. The cavity signal (background) is labelled with an asterisk (*). Simulation parameters: $g_{\perp}$ = 0.57 (g_x = g_y), $g_{\parallel}$ = 1.33 (g_z).

10. Density Functional Theory (DFT) Calculations

10.1. Calculational Details

All DFT calculations were performed with ORCA v. 5.0.1[25,26], starting from single crystal structures, in which counter ions or solvent molecules in lattice were omitted. All structures were optimized twice. Firstly, valence double-zeta basis sets with polarization functions def2-SVP were applied for all light atoms[27]. The zeroth order regular approximation (ZORA) was adopted to take scalar relativistic effects into account[28], and accordingly, adapted versions of the def2 basis sets and segmented all-electron relativistically contracted (SARC) basis sets were utilized for the light atoms and the heavy atoms (iodine and uranium) respectively[29,30]. The TRAH solver was turned off, otherwise leading to convergence problem. Hybrid functional B3PW with dispersion correction ("D3BJ") was chosen[31-33], while other settings were default. After convergence, larger basis sets at triple-zeta level, def2-TZVP[27], were used for non-hydrogen atoms. Both the calculated structures optimized at different levels of basis sets matched the single crystal structures well, and the data listed below were from the one with larger basis sets. Natural localized molecular orbital (NLMO) analysis was carried out with NBO 6.0[34]. VMD v. 1.9.3 was used to visualize the molecular orbitals[35], combined with the wave function analysis program Multiwfn[36]. The Extended Transition State–Natural Orbitals for Chemical Valence (ETS–NOCV) calculations were carried on Multiwfn to probe the uranium–arene interactions in compounds **1**–**5**[37]. All compounds were divided into two fragments, the anchoring arene and the rest of the molecule, by breaking three C_{ipso}–C_{ipso} bonds between the side rings and the anchoring arene. The spins of the anchoring arene are paired with the rest of the molecule upon combining the two fragments.

10.2. Comparison between Experimental (X-ray) and Calculated Structures

Supplementary Table 7. Selected structural parameters of (^{Ad}TPBN$_3$)U (**1**).

Selected distances [Å] and angles [°]	Exp.	Calc.	Diff.
U–N1	2.413(3)	2.382	–0.031
U–N2	2.429(2)	2.393	–0.036
U–N3	2.426(2)	2.394	–0.032
U–C1	2.733(3)	2.701	–0.032
U–C2	2.742(3)	2.707	–0.035
U–C3	2.734(3)	2.716	–0.018
U–C4	2.728(3)	2.728	0.000
U–C5	2.722(2)	2.718	–0.004
U–C6	2.720(3)	2.707	–0.013
U–C$_{centroid}$	2.339(1)	2.323	–0.016
U–3N$_{plane}$	–0.170(1)	–0.153	–0.017
C1–C2	1.417(4)	1.411	–0.006
C2–C3	1.392(5)	1.393	0.001
C3–C4	1.420(5)	1.409	–0.011
C4–C5	1.398(4)	1.392	–0.006
C5–C6	1.413(5)	1.410	–0.003
C6–C1	1.407(5)	1.394	–0.013
N1–U–N2	119.85(8)	120.48	0.63
N2–U–N3	119.59(8)	118.13	–1.46
N3–U–N1	119.09(8)	120.19	1.10

Supplementary Table 8. Selected structural parameters of [(^{Ad}TPBN$_3$)U]$^-$ (The anionic part of **2**).

Selected distances [Å] and angles [°]	Exp.*	Calc.	Diff.
U–N1	2.470(3)	2.470	0.000
U–N2	2.483(3)	2.473	–0.010
U–N3	2.478(3)	2.474	–0.004
U–C1	2.593(4)	2.598	0.005
U–C2	2.616(4)	2.614	–0.002
U–C3	2.592(4)	2.596	0.004
U–C4	2.599(4)	2.615	0.016
U–C5	2.583(3)	2.599	0.016
U–C6	2.592(4)	2.613	0.021
U–$C_{centroid}$	2.175(1)	2.189	0.014
U–3N_{plane}	–0.339(1)	–0.329	0.010
C1–C2	1.415(5)	1.414	–0.001
C2–C3	1.424(6)	1.414	–0.010
C3–C4	1.420(5)	1.415	–0.005
C4–C5	1.403(6)	1.409	0.006
C5–C6	1.420(5)	1.418	–0.002
C6–C1	1.423(6)	1.410	–0.013
N1–U–N2	118.62(9)	117.83	–0.79
N2–U–N3	116.82(9)	117.49	0.67
N3–U–N1	119.03(9)	119.49	0.46

*: The average of two independent molecules in a crystallographic asymmetric unit.

Supplementary Table 9. Selected structural parameters of (^{Ad}TPBN$_3$)UO (**3**).

Selected distances [Å] and angles [°]	Exp.*	Exp.**	Calc.	Diff.*	Diff.**
U–O	1.829(2)	1.836(3)	1.814	–0.015	–0.022
U–N1	2.321(2)	2.342(2)	2.327	0.006	–0.015
U–N2	2.327(3)	2.312(3)	2.299	–0.028	–0.013
U–N3	2.302(2)	2.311(3)	2.310	0.008	–0.001
U–C1	2.918(2)	2.931(3)	2.911	–0.007	–0.020
U–C2	2.939(2)	2.908(4)	2.922	–0.017	0.014
U–C3	2.926(3)	2.885(4)	2.914	–0.012	0.029
U–C4	2.926(2)	2.915(4)	2.933	0.007	0.018
U–C5	2.903(3)	2.915(3)	2.911	0.008	–0.004
U–C6	2.919(3)	2.937(3)	2.924	0.005	–0.013
U–$C_{centroid}$	2.565(1)	2.556(1)	2.563	–0.002	0.007
U–3N_{plane}	+0.122(1)	+0.093(1)	+0.127	0.005	0.034
C1–C2	1.383(4)	1.390(4)	1.387	0.004	–0.003
C2–C3	1.409(4)	1.415(4)	1.409	0.000	–0.006
C3–C4	1.383(4)	1.391(4)	1.386	0.003	–0.005
C4–C5	1.422(4)	1.408(4)	1.409	–0.013	0.001
C5–C6	1.383(3)	1.386(4)	1.386	0.003	0.000
C6–C1	1.411(3)	1.420(4)	1.408	–0.003	–0.012
N1–U–N2	118.34(8)	120.03(10)	120.11	1.77	0.08
N2–U–N3	119.37(8)	118.35(9)	118.63	–0.74	0.28
N3–U–N1	121.46(8)	121.14(10)	120.36	–1.10	–0.78

*: Based on the crystallographic data of (^{Ad}TPBN$_3$)UO (**3**)
: Based on the crystallographic data of (^{Ad}TPBN$_3$)UO·C_7H_8 (3**·C_7H_8)

Supplementary Table 10. Selected structural parameters of [(^{Ad}TPBN$_3$)UO]$^-$ (The anionic part of **4** and **4′**).

Selected distances [Å] and angles [°]	Exp. **4** *	Exp. **4′** **	Calc.	Diff.*	Diff.**
U–O	1.874(4)	1.851(7)	1.868	–0.006	0.017
U–N1	2.421(4)	2.432(8)	2.422	0.001	–0.010
U–N2	2.421(4)	2.452(9)	2.455	0.034	0.003
U–N3	2.421(4)	2.418(8)	2.431	0.010	0.013
U–C1	3.023(4)	2.952(9)	2.946	–0.077	–0.006
U–C2	3.034(4)	2.991(11)	2.990	–0.044	–0.001
U–C3	3.023(4)	2.960(10)	2.991	–0.032	0.031
U–C4	3.034(4)	3.000(9)	3.002	–0.032	0.002
U–C5	3.023(4)	3.003(9)	2.970	–0.053	–0.033
U–C6	3.034(4)	2.967(10)	2.955	–0.079	–0.012
U–$C_{centroid}$	2.686(1)	2.635(1)	2.627	–0.059	–0.008
U–3N_{plane}	+0.253(1)	+0.189(1)	+0.228	–0.025	0.039
C1–C2	1.417(6)	1.409(15)	1.408	–0.009	–0.001
C2–C3	1.381(6)	1.370(14)	1.386	0.005	0.016
C3–C4	1.417(6)	1.397(13)	1.407	–0.010	0.010
C4–C5	1.381(6)	1.390(12)	1.387	0.006	–0.003
C5–C6	1.417(6)	1.391(13)	1.409	–0.008	0.018
C6–C1	1.381(6)	1.387(15)	1.389	0.008	0.002
N1–U–N2	118.9(1)	118.5(3)	117.0	–1.9	–1.5
N2–U–N3	118.9(1)	118.9(3)	123.1	4.2	4.2
N3–U–N1	118.9(1)	120.8(3)	117.3	–1.6	–3.5

*: Based on the crystallographic data of [K(crypt)][(^{Ad}TPBN$_3$)UO] (**4**)

: Based on the crystallographic data of [Cp$^*_2$Co][(^{Ad}TPBN$_3$)UO]·THF (4′**·THF)

Supplementary Table 11. Selected structural parameters for $[(^{Ad}TPBN_3)UO]^+$ (The cationic part of **5**).

Selected distances [Å] and angles [°]	Exp.	Calc.	Diff.
U–O	1.818(2)	1.800	–0.018
U–N1	2.275(3)	2.248	–0.027
U–N2	2.270(3)	2.244	–0.026
U–N3	2.276(3)	2.251	–0.025
U–C1	2.856(3)	2.860	0.004
U–C2	2.868(4)	2.878	0.010
U–C3	2.844(4)	2.862	0.018
U–C4	2.861(4)	2.876	0.015
U–C5	2.852(3)	2.858	0.006
U–C6	2.881(3)	2.875	–0.006
U–$C_{centroid}$	2.493(1)	2.504	0.011
U–3N_{plane}	+0.036(1)	+0.067	0.031
C1–C2	1.390(6)	1.386	–0.004
C2–C3	1.418(6)	1.411	–0.007
C3–C4	1.395(5)	1.386	–0.009
C4–C5	1.416(5)	1.411	–0.005
C5–C6	1.389(6)	1.385	–0.004
C6–C1	1.412(5)	1.411	–0.001
N1–U–N2	120.80(10)	120.07	–0.73
N2–U–N3	119.42(10)	119.91	0.49
N3–U–N1	119.71(10)	119.75	0.04

Supplementary Table 12. Selected structural parameters of (^{Ad}TPBN_3)UF (**6**).

Selected distances [Å] and angles [°]	Exp.	Calc.	Diff.
U–F	2.090(2)	2.114	0.024
U–N1	2.309(2)	2.335	0.026
U–N2	2.323(3)	2.310	–0.013
U–N3	2.338(3)	2.308	–0.030
U–C1	2.872(3)	2.892	0.020
U–C2	2.893(3)	2.899	0.006
U–C3	2.893(3)	2.882	–0.011
U–C4	2.909(3)	2.897	–0.012
U–C5	2.900(3)	2.890	–0.010
U–C6	2.894(3)	2.907	0.013
U–$C_{centroid}$	2.532(1)	2.535	0.003
U–3N_{plane}	+0.070(1)	+0.093	0.023
C1–C2	1.386(4)	1.386	0.000
C2–C3	1.411(5)	1.410	–0.001
C3–C4	1.388(5)	1.387	–0.001
C4–C5	1.408(4)	1.410	0.002
C5–C6	1.385(5)	1.385	0.000
C6–C1	1.420(5)	1.408	–0.012
N1–U–N2	121.47(9)	119.84	–0.63
N2–U–N3	118.90(9)	120.56	1.64
N3–U–N1	119.36(8)	119.12	–0.24

Supplementary Table 13. Selected structural parameters of (^{Ad}TPBN$_3$)UI (**7**).

Selected distances [Å] and angles [°]	Exp.	Calc.	Diff.
U–I	3.085(1)	3.065	–0.020
U–N1	2.279(3)	2.309	0.030
U–N2	2.289(3)	2.297	0.008
U–N3	2.310(3)	2.307	–0.003
U–C1	2.978(4)	2.890	–0.088
U–C2	2.980(4)	2.894	–0.086
U–C3	2.970(4)	2.884	–0.086
U–C4	2.984(4)	2.907	–0.077
U–C5	2.980(4)	2.896	–0.084
U–C6	2.995(4)	2.908	–0.087
U–$C_{centroid}$	2.632(1)	2.538	–0.094
U–3N_{plane}	+0.107(1)	+0.090	–0.017
C1–C2	1.384(6)	1.386	0.002
C2–C3	1.413(5)	1.409	–0.004
C3–C4	1.388(6)	1.385	–0.003
C4–C5	1.416(6)	1.409	–0.007
C5–C6	1.386(5)	1.385	–0.001
C6–C1	1.414(6)	1.408	–0.006
N1–U–N2	119.50(11)	119.96	0.46
N2–U–N3	120.20(11)	119.99	–0.21
N3–U–N1	119.66(11)	119.60	–0.06

10.3. Composition Analysis of Molecular Orbitals (MOs)

Supplementary Table 14. Composition analysis of the singly occupied orbitals (SOMOs) of (^{Ad}TPBN$_3$)U (**1**).

MO	Energy (eV)	U (%)	Anchor ring (%)
**250α**	–4.18	78.0 (*d* 2.2, *f* 75.7)	C *p* 11.2
**249α**	–4.20	81.4 (*d* 2.7, *f* 78.7)	C *p* 10.9
**248α**	–4.55	5*f* 96.1	/

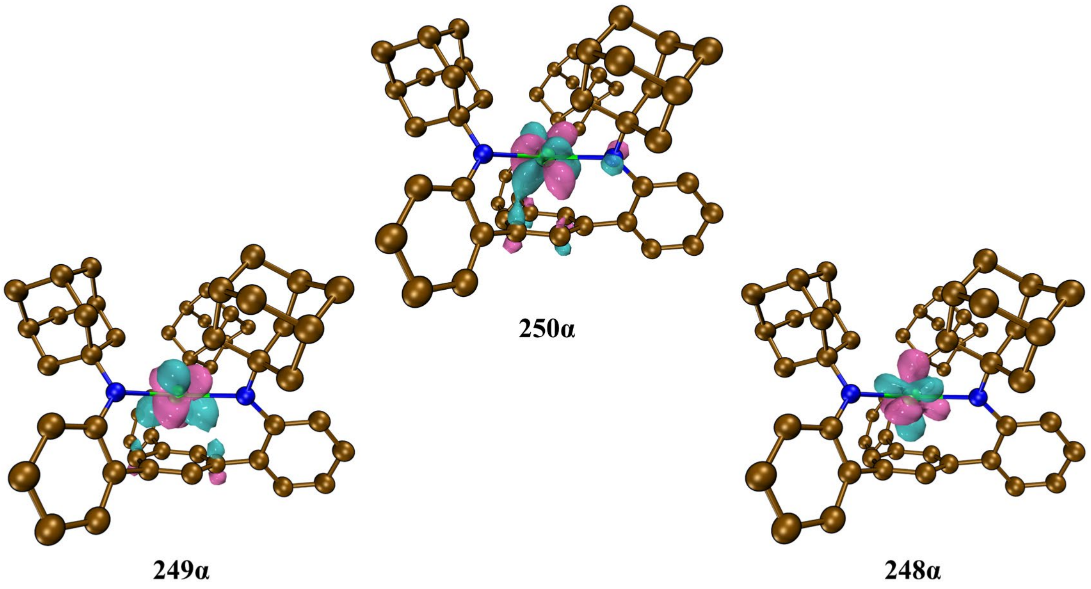

Supplementary Fig. 98. Kohn-Sham orbitals (isosurface = 0.05) of the SOMOs of (^{Ad}TPBN$_3$)U (**1**). All hydrogens were omitted for clarity.

Supplementary Table 15. Composition analysis of the SOMOs of $[(^{Ad}TPBN_3)U]^-$ (**2**).

MO	Energy (eV)	U (%)	Anchor ring (%)
**251α**	–0.42	47.1 (*d* 4.2, *f* 42.2)	C *p* 42.4
**250α**	–0.48	53.2 (*d* 3.7, *f* 48.8)	C *p* 37.4
**249α**	–0.69	5*f* 87.0	/
**248α**	–1.18	5*f* 95.0	/

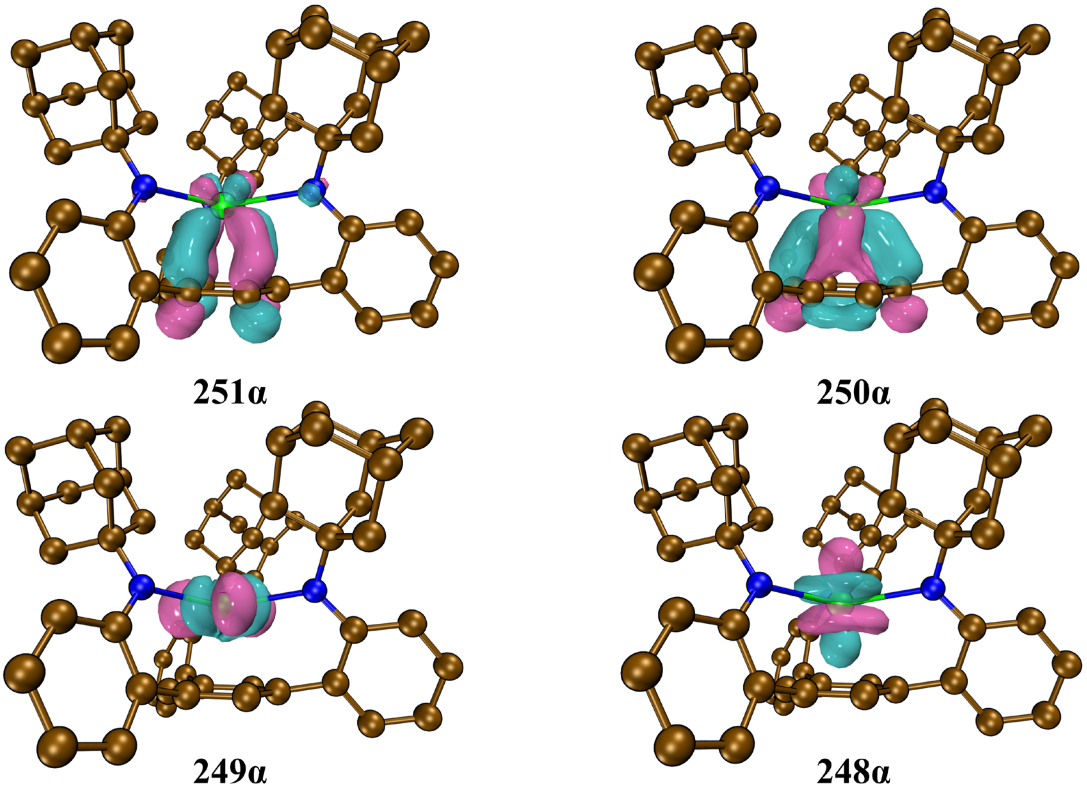

Supplementary Fig. 99. Kohn-Sham orbitals (isosurface = 0.05) of the SOMOs of $[(^{Ad}TPBN_3)U]^-$ (**2**). All hydrogens were omitted for clarity.

Supplementary Table 16. Composition analysis of the MOs of (^{Ad}TPBN$_3$)UO (**3**), featuring π-interactions from π orbitals of the arene anchor to uranium-based orbitals.

MO	Energy (eV)	U (%)	O (%)	Anchor ring (%)
**234α**	–8.20	7.3 (*p* 1.1, *d* 1.3, *f* 4.9)	*p* 15.1	C *p* 27.3
**233α**	–8.21	7.2 (*p* 1.1, *d* 1.3, *f* 4.9)	*p* 13.6	C *p* 29.5
**231α**	–8.38	4.8 (*d* 1.7, *f* 2.8)	*p* 3.0	C *p* 20.6
**230α**	–8.40	3.8 (*d* 2.2, *f* 1.5)	*p* 1.0	C *p* 16.6

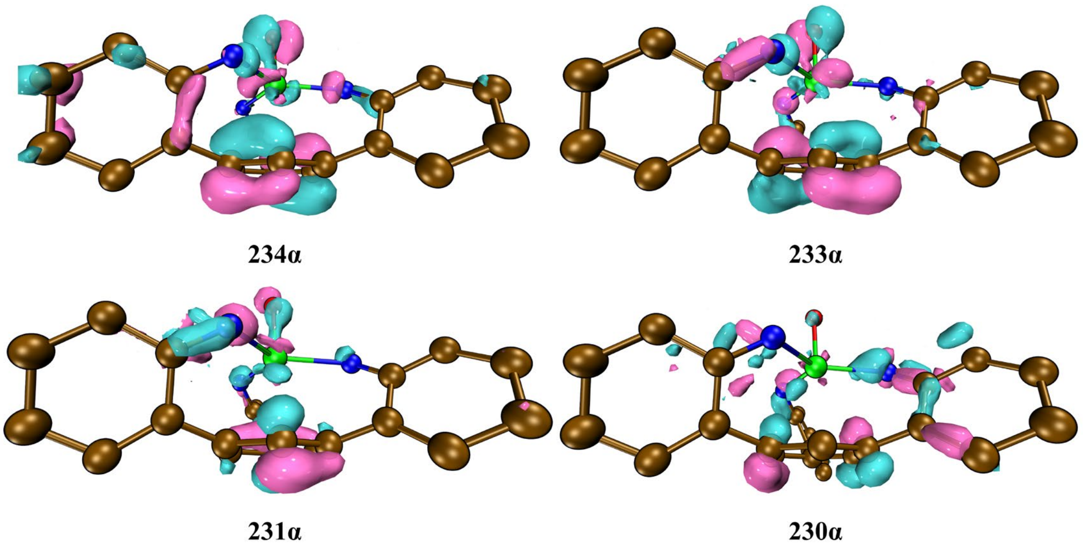

Supplementary Fig. 100. Kohn-Sham orbitals (isosurface = 0.04) of the MOs of (^{Ad}TPBN$_3$)UO (**3**), featuring π-interactions from π orbitals of the anchoring arene to uranium-based orbitals. All hydrogens and the adamantyl groups were omitted for clarity.

Supplementary Table 17. Composition analysis of the MOs of [(^{Ad}TPBN$_3$)UO]$^−$ (**4**), featuring π-interactions from π orbitals of the arene anchor to uranium-based orbitals.

MO	Energy (eV)	U (%)	O (%)	Anchor ring (%)
**234α**	–5.48	4.2 (*d* 3.6, *f* 0.3)	*p* 4.3	C *p* 40.4
**233α**	–5.50	4.2 (*d* 3.4, *f* 0.3)	*p* 3.9	C *p* 36.7

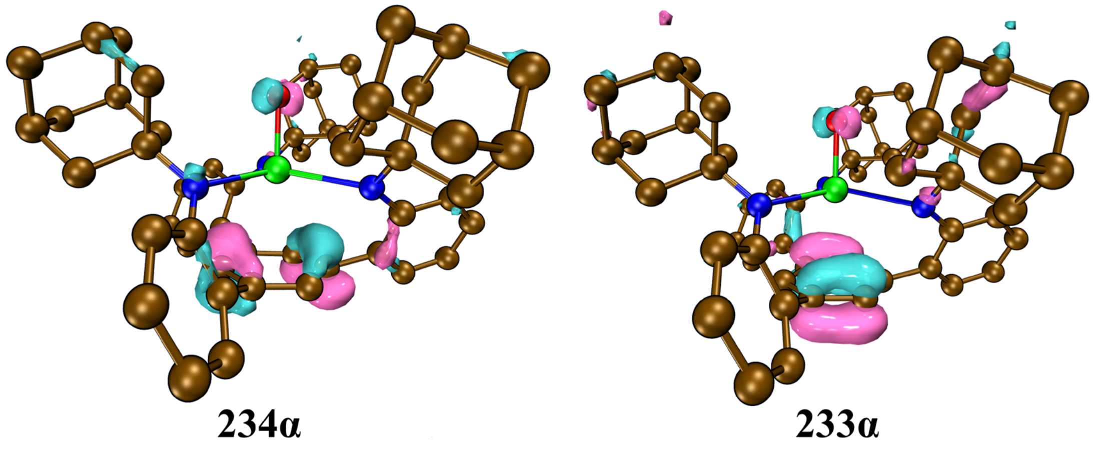

Supplementary Fig. 101. Kohn-Sham orbitals (isosurface = 0.05) of the MOs of [(^{Ad}TPBN$_3$)UO]$^−$ (**4**), featuring π-interactions from π orbitals of the arene anchor to uranium-based orbitals. All hydrogens were omitted for clarity.

Supplementary Table 18. Composition analysis of the MOs of $[(^{Ad}TPBN_3)UO]^+$ (**5**), featuring π-interactions from π orbitals of the arene anchor to uranium-based orbitals.

MO	Energy (eV)	U (%)	O (%)	Anchor ring (%)
**231**	–11.19	4.4 (*d* 0.6, *f* 3.4)	*p* 3.7	C *p* 22.2
**230**	–11.20	4.5 (*d* 0.6, *f* 3.5)	*p* 3.5	C *p* 24.8
**222**	–11.42	4.1 (*d* 1.0, *f* 2.2)	*p* 2.3	C *p* 23.4
**221**	–11.42	4.0 (*d* 1.0, *f* 2.2)	*p* 2.2	C *p* 23.6

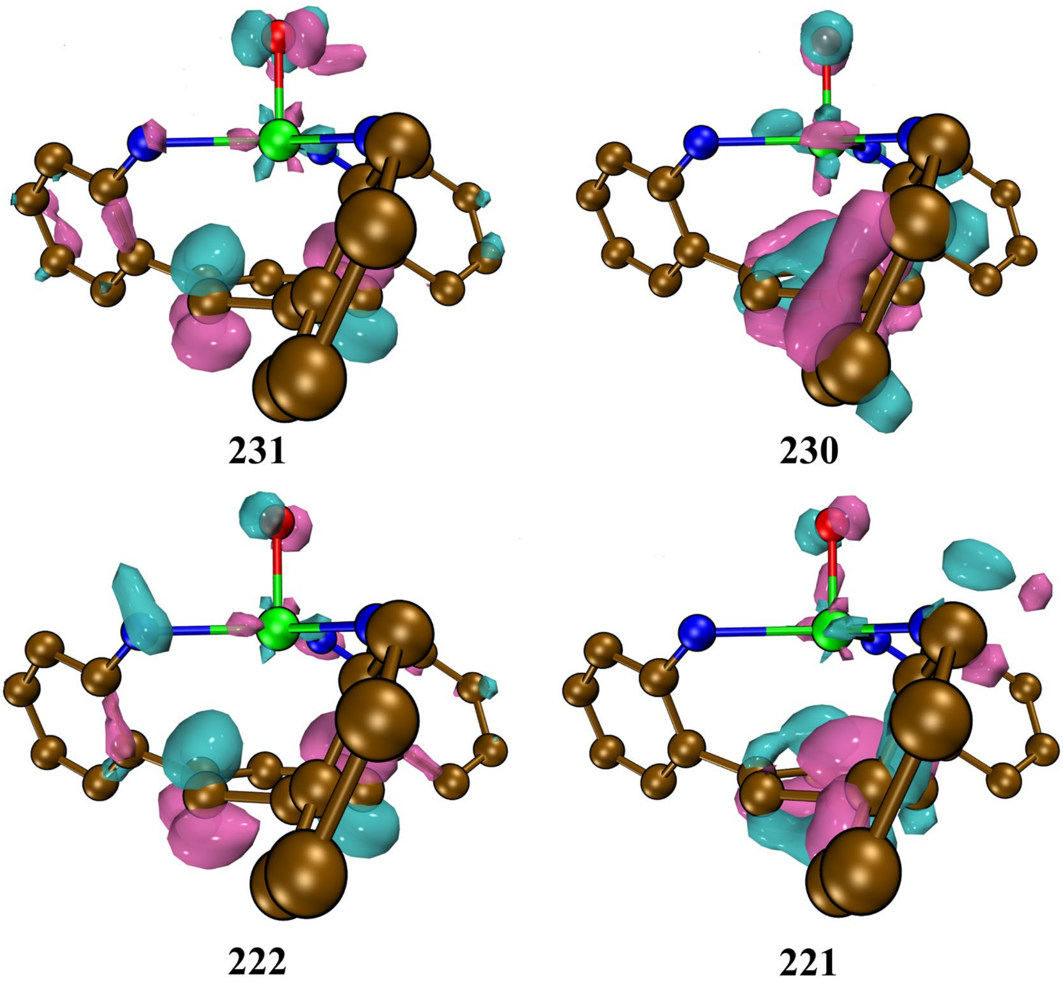

Supplementary Fig. 102. Kohn-Sham orbitals (isosurface = 0.04) of the MOs of $[(^{Ad}TPBN_3)UO]^+$ (**5**), featuring π-interactions from π orbitals of the anchoring arene to uranium-based orbitals. All hydrogens and the adamantyl groups were omitted for clarity.

10.4. Natural Localized Molecular Orbital (NLMO) Analysis

Supplementary Table 19. NLMO contributions for U–O interactions in [(^{Ad}TPBN$_3$)UO]$^-$ (**4**).

U–O interactions	U (%)	O (%)
**σ** (105α)	13.9 (*d* 3.2, *f* 9.9)	84.7 (*s* 8.2, *p* 76.1)
**π_1** (106α)	14.5 (*d* 5.4, *f* 9.1)	*p* 84.1
**π_2** (107α)	15.0 (*d* 5.2, *f* 9.7)	*p* 83.6

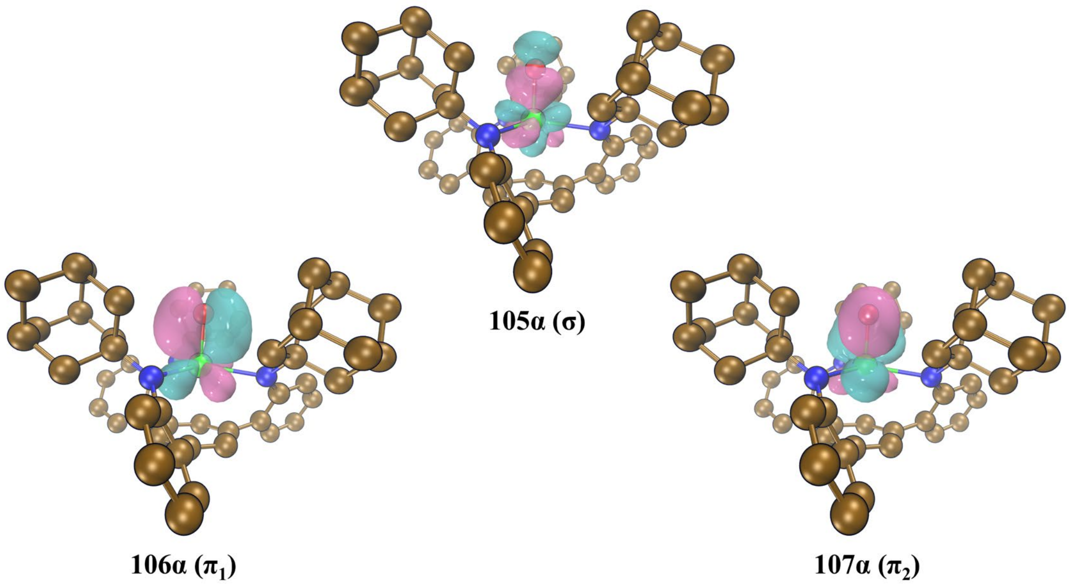

Supplementary Fig. 103. NLMOs (isosurface = 0.05) for U–O interactions in [(^{Ad}TPBN$_3$)UO]$^-$ (**4**). All hydrogens were omitted for clarity.

Supplementary Table 20. NLMO contributions for U–O interactions in ([Ad]TPBN3)UO (**3**).

U–O interactions	U (%)	O (%)
**σ** (104α)	17.7 (*d* 2.7, *f* 14.3)	81.0 (*s* 9.2, *p* 71.4)
**π_1** (105α)	17.8 (*d* 4.8, *f* 13.0)	*p* 80.6
**π_2** (106α)	17.9 (*d* 4.8, *f* 13.0)	*p* 80.8

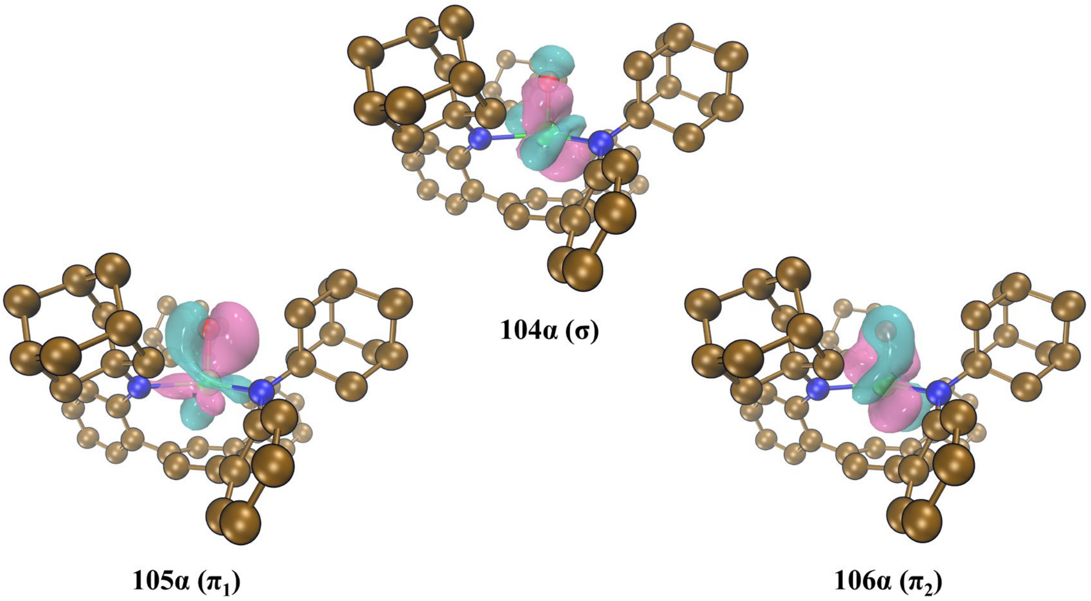

Supplementary Fig. 104. NLMOs (isosurface = 0.05) for U–O interactions in ([Ad]TPBN3)UO (**3**). All hydrogens were omitted for clarity.

Supplementary Table 21. NLMO contributions for U–O interactions in [(^{Ad}TPBN$_3$)UO]$^+$ (**5**).

**U–O interactions**	**U (%)**	**O (%)**
**σ** (103)	16.6 (*d* 3.1, *f* 12.9)	82.0 (*s* 13.7, *p* 67.8)
$\boldsymbol{\pi_1}$ (104)	19.4 (*d* 5.0, *f* 14.3)	*p* 79.5
$\boldsymbol{\pi_2}$ (105)	19.4 (*d* 4.9, *f* 14.4)	*p* 79.5

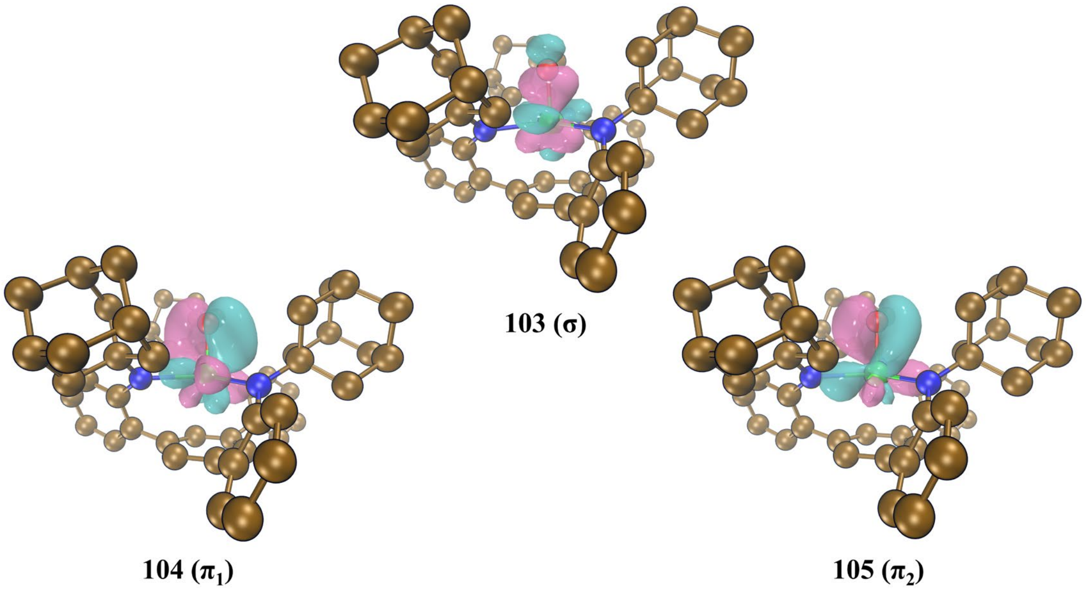

Supplementary Fig. 105. NLMOs (isosurface = 0.05) for U–O interactions in [(^{Ad}TPBN$_3$)UO]$^+$ (**5**). All hydrogens were omitted for clarity.

Supplementary Table 22. NLMO contributions for U–F interactions in ([Ad]TPBN_3)UF (**6**).

**U–F interactions**	**U (%)**	**F (%)**
**σ** (104α)	4.7 (*d* 2.3, *f* 2.0)	94.9 (*s* 43.7, *p* 51.2)
$\boldsymbol{\pi_1}$ (105α)	5.4 (*d* 2.1, *f* 3.3)	93.9 (*s* 3.1, *p* 90.8)
$\boldsymbol{\pi_2}$ (106α)	5.4 (*d* 2.1, *f* 3.3)	93.9 (*s* 3.1, *p* 90.8)

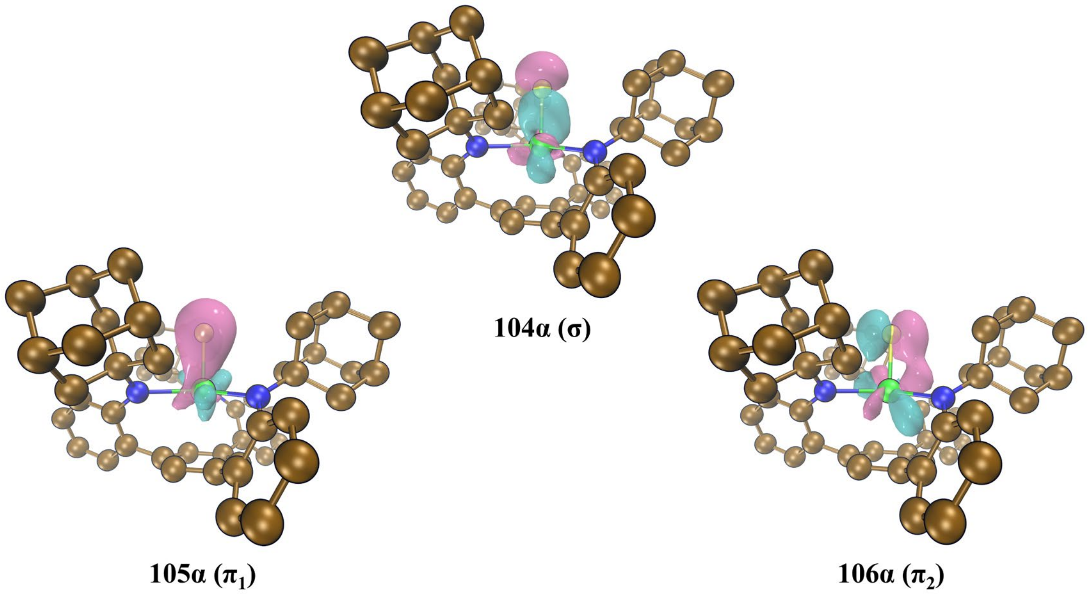

Supplementary Fig. 106. NLMOs (isosurface = 0.05) for U–F interactions in ([Ad]TPBN_3)UF (**6**). All hydrogens were omitted for clarity.

Supplementary Table 23. NLMO contributions for U–I interactions in (^{Ad}TPBN$_3$)UI (**7**).

U–I interactions	U (%)	I (%)
**σ** (126α)	10.3 (*s* 1.2, *d* 5.8, *f* 2.8)	88.9 (*s* 44.3, *p* 44.5)
**π$_1$** (127α)	9.2 (*d* 4.1, *f* 5.2)	*p* 88.9
**π$_2$** (128α)	9.2 (*d* 4.1, *f* 5.2)	*p* 88.9

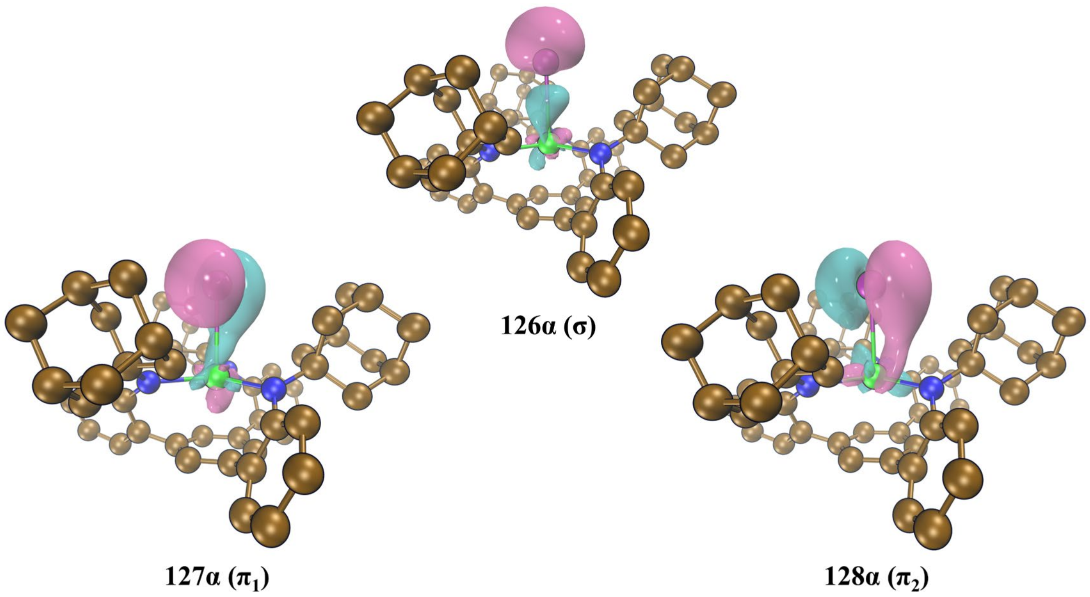

Supplementary Fig. 107. NLMOs (isosurface = 0.05) for U–I interactions in (^{Ad}TPBN$_3$)UI (**7**). All hydrogens were omitted for clarity.

10.5. Calculated Bond Orders

Supplementary Table 24. Wiberg bond index of the selected bonds for compounds **1**–**7**.

**Compounds**	**U Oxidation states**	**U–O/F/I**	**Avg. U–N**	**Avg. U–C**	**Avg. C–C for the anchor ring**
**2**	II	/	0.49	0.35	1.24
**1**	III	/	0.57	0.25	1.30
**6**	IV	0.73	0.63	0.15	1.33
**7**	IV	1.21	0.67	0.15	1.33
**4**	IV	1.52	0.44	0.13	1.34
**3**	V	1.77	0.70	0.16	1.32
**5**	VI	1.93	0.99	0.20	1.31

10.6. Population Analysis

Supplementary Table 25. Calculated Mulliken atomic charges for compounds **1**–**7**.

Compounds	U Oxidation states	U	O/F/I	Avg. N_{amide}	The Anchor ring (C+H/C)*
**2**	II	0.84	/	–0.65	–0.09/–0.60
**1**	III	0.90	/	–0.65	0.29/–0.30
**6**	IV	1.30	–0.56	–0.65	0.28/–0.36
**7**	IV	0.93	–0.31	–0.64	0.23/–0.39
**4**	IV	1.31	–0.81	–0.69	0.16/–0.43
**3**	V	1.41	–0.71	–0.64	0.31/–0.31
**5**	VI	1.34	–0.65	–0.54	0.40/–0.28

*: C+H, containing six carbon atoms and three hydrogen of the anchor ring; C, only containing six carbon atoms of the anchor ring.

Supplementary Table 26. Calculated spin populations on uranium for compounds **1**–**7**.

Compounds	U Oxidation states	Spin populations on uranium
**2**	II	3.27
**1**	III	2.94
**6**	IV	2.16
**7**	IV	2.21
**4**	IV	2.15
**3**	V	1.17
**5**	VI	0

Supplementary Table 27. Calculated natural charges for compounds **1**–**7**.

Compounds	U Oxidation states	U	O/F/I	N Avg.	The Anchor ring (C+H/C)*
**2**	II	0.88	/	–0.68	–0.30/–1.15
**1**	III	0.92	/	–0.69	0.18/–0.71
**6**	IV	1.36	–0.57	–0.72	0.26/–0.62
**7**	IV	0.88	–0.17	–0.73	0.26/–0.62
**4**	IV	1.47	–1.03	–0.71	0.17/–0.68
**3**	V	1.45	–0.87	–0.67	0.30/–0.59
**5**	VI	1.24	–0.74	–0.56	0.42/–0.48

*: C+H, containing six carbon atoms and three hydrogen of the anchor ring; C, only containing six carbon atoms of the anchor ring.

Supplementary Table 28. Calculated natural spin density on uranium for compounds **1**–**7**.

**Compounds**	**U Oxidation states**	**Spin density on uranium**
**2**	II	3.05
**1**	III	2.78
**6**	IV	2.05
**7**	IV	2.08
**4**	IV	2.03
**3**	V	1.09
**5**	VI	0

10.7. ETS–NOCV Analysis

Supplementary Table 29. Summary of NOCV energies in kcal/mol for compounds **1**–**5**.

Cpd	U OS*	σ	π		δ		Total energies#
		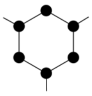 π_1	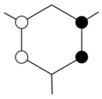 π_2	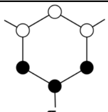 π_3	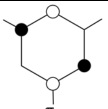 π_4	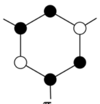 π_5	
**2**	II	–8.1	–26.1	–25.8	–79.1	–74.6	–213.7 (–60.0)
**1**	III	–13.5	–25.0	–23.7	–39.9	–49.7	–151.8 (–62.2)
**4**	IV	–11.0	–32.3	–29.9	/	/	–73.2
**3**	V	–12.9	–37.1	–34.7	/	/	–84.7
**5**	VI	–15.7	–44.0	–43.9	/	/	–103.6

*: Uranium oxidation states; #: Stabilisation energies attributed to the σ and π donations from the anchoring arene to uranium in brackets. For U(IV–VI), since no δ backdonation from uranium to the anchoring arene was found, the total energies equal to the stabilisation energies of the σ and π donations from the anchoring arene to uranium.

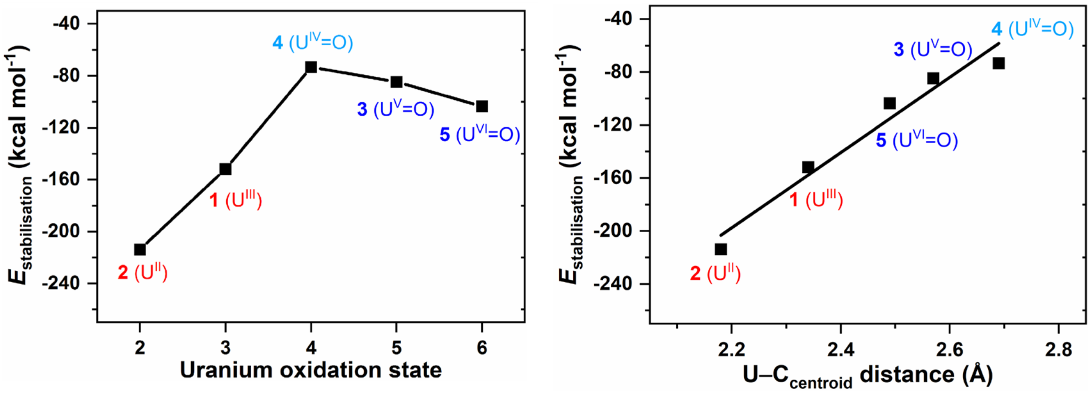

Supplementary Fig. 108. The plots of the total stabilisation energies of uranium–arene interactions ($E_{stabilisation}$) versus oxidation states of uranium (left) and U–C$_{centroid}$ distances (right) for **1**–**5**. For $E_{stabilisation}$ vs. U–C$_{centroid}$ distances, a linear fitting with R^2 = 0.96 is shown.

Supplementary Table 30. Selected NOCV pairs of [(^{Ad}TPBN$_3$)U]$^-$ (the anionic part of **2**), showing interactions between the anchoring arene ((C_6H_3)$^{3\cdot}$, ^{4}A) and the fragment containing U(II) ({[AdN(C_6H_4)$^\cdot$]$_3$U}$^-$, ^{8}A). The eigenvalue *v* of the NOCV pairs corresponds to the degree of charge migration. The direction of the negative charge flow is from red to blue. Main fragment orbital contributors are shown for each NOCV pair, where the orbital composition (only those larger than 3%) of U is given. Hydrogens are omitted for clarity, except for the hydrogens on the anchoring arene (labelled in yellow). Only α orbitals were shown for fragment orbital contributors, except for σ interaction (the corresponding α orbital in uranium fragment is the singly-occupied 6d_{z2} orbital).

TOI	NOCV Pair Densities	Fragment Orbital Contributors
σ	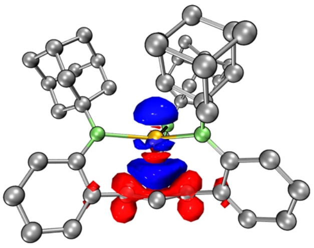<br>$\|v_\beta\|$ = 0.15<br>-8.1 kcal/mol<br>σ donation<br>iso 0.0005	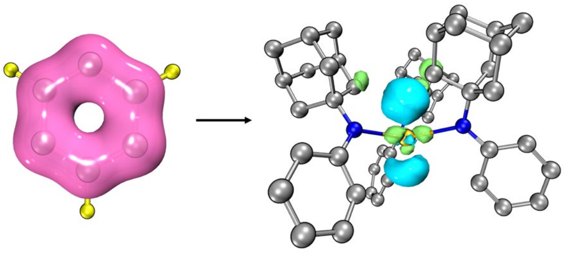<br>β HOMO-2<br>π_1<br>iso 0.05<br>→<br>β LUMO+15<br>49% U 6d, 41% U 7s<br>iso 0.05
π	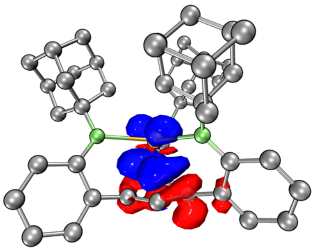<br>$\|v_\alpha\|$ = 0.19, $\|v_\beta\|$ = 0.23<br>-26.1 kcal/mol<br>π donation<br>iso 0.001	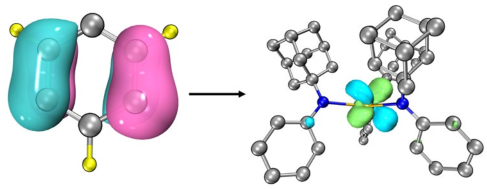<br>α HOMO-2<br>π_2<br>iso 0.05<br>→<br>α LUMO+12<br>39% U 5f, 56% U 6d<br>iso 0.05
	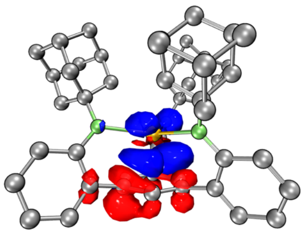<br>$\|v_\alpha\|$ = 0.19, $\|v_\beta\|$ = 0.23<br>-25.8 kcal/mol<br>π donation<br>iso 0.001	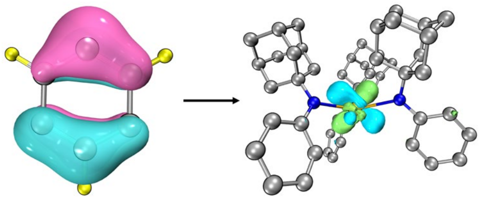<br>α HOMO-3<br>π_3<br>iso 0.05<br>→<br>α LUMO+11<br>40% U 5f, 54% U 6d<br>iso 0.05

	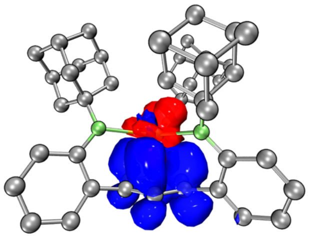<br>$\lvert v_{\alpha} \rvert = 0.94$<br>-79.1 kcal/mol<br>δ backdonation<br>iso 0.001	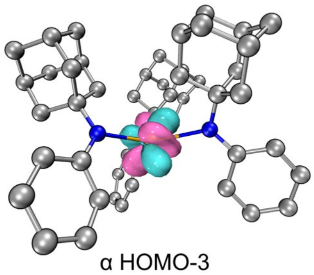<br>α HOMO-3<br>39% U $5f_{\pi}$, 53% U $5f_{\delta}$<br>iso 0.05	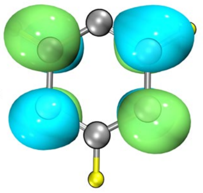<br>α LUMO<br>π_4<br>iso 0.05
	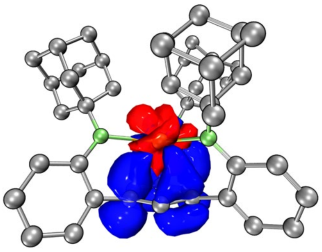<br>$\lvert v_{\alpha} \rvert = 0.90$<br>-74.6 kcal/mol<br>δ backdonation<br>iso 0.001	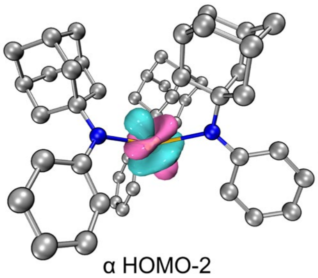<br>α HOMO-2<br>60% U $5f_{\pi}$, 29% U $5f_{\delta}$<br>iso 0.05	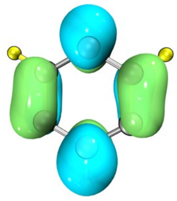<br>α LUMO+1<br>π_5<br>iso 0.05

TOI: Types of Interactions.

Supplementary Table 31. Selected NOCV pairs of (^{Ad}TPBN$_3$)U (**1**), featuring interactions between the anchoring arene ((C_6H_3)$^{3\cdot}$, ^{4}A) and the fragment containing U(III) ([AdN(C_6H_4)$^{\cdot}$]$_3$U, ^{7}A). Hydrogens are omitted for clarity, except for the hydrogens on the anchoring arene (labelled in yellow). Only α orbitals were shown for fragment orbital contributors.

TOI	NOCV Pair Densities	Fragment Orbital Contributors
σ	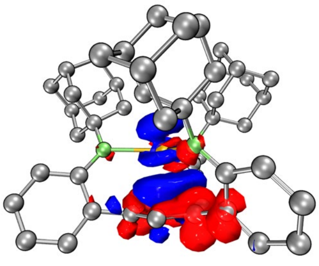<br>$\lvert v_\alpha \rvert$ = 0.14, $\lvert v_\beta \rvert$ = 0.14<br>-13.5 kcal/mol<br>σ donation<br>iso 0.0005	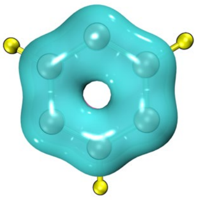<br>α HOMO-5<br>π_1<br>iso 0.05<br>→<br>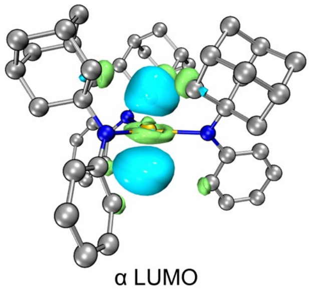<br>α LUMO<br>70% U 6d, 37% U 7s<br>iso 0.05
π	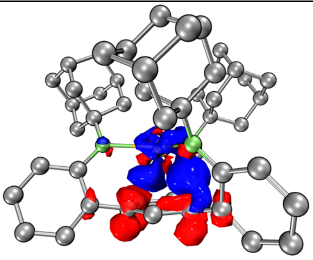<br>$\lvert v_\alpha \rvert$ = 0.23, $\lvert v_\beta \rvert$ = 0.23<br>-25.0 kcal/mol<br>π donation<br>iso 0.001	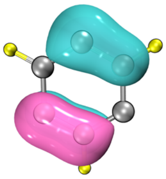<br>α HOMO-2<br>π_2<br>iso 0.05<br>→<br>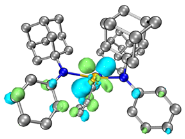<br>α LUMO+1<br>10% U 5f, 77% U 6d<br>iso 0.05<br>+<br>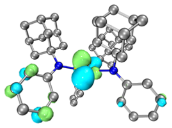<br>α LUMO+2<br>50% U 5f, 37% U 6d<br>iso 0.05
	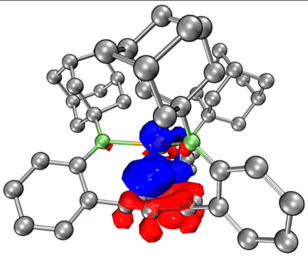<br>$\lvert v_\alpha \rvert$ = 0.21, $\lvert v_\beta \rvert$ = 0.23<br>-23.7 kcal/mol<br>π donation<br>iso 0.001	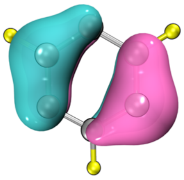<br>α HOMO-3<br>π_3<br>iso 0.05<br>→<br>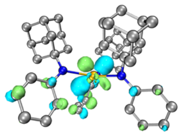<br>α LUMO+1<br>10% U 5f, 77% U 6d<br>iso 0.05<br>+<br>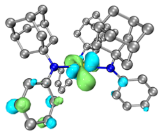<br>α LUMO+3<br>55% U 5f, 24% U 6d<br>iso 0.05
	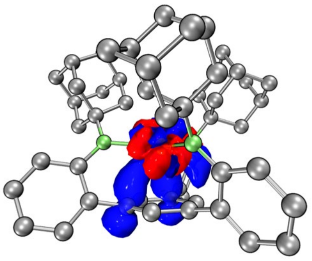<br>$\lvert v_\alpha \rvert$ = 0.64<br>-39.9 kcal/mol<br>δ backdonation<br>iso 0.001	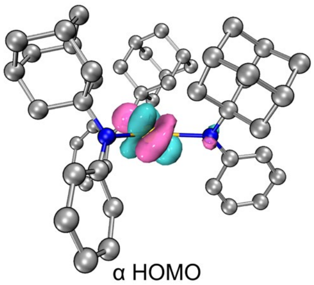<br>α HOMO<br>9% U 5f$_\pi$, 62% U 5f$_\delta$, 19% U 5f$_\varphi$<br>iso 0.05<br>→<br>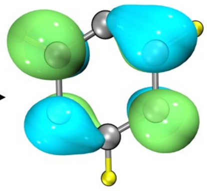<br>α LUMO<br>π_4<br>iso 0.05

	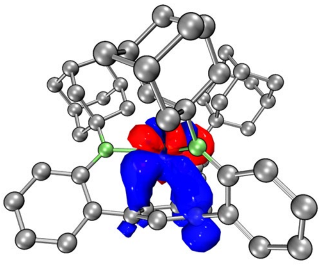<br>$\lvert v_{\alpha} \rvert$ = 0.74<br>-49.7 kcal/mol<br>δ backdonation<br>iso 0.001	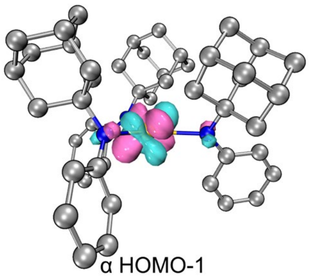<br>α HOMO-1<br>5% U $5f_{\pi}$, 67% U $5f_{\delta}$, 16% U $5f_{\varphi}$<br>iso 0.05<br>→<br>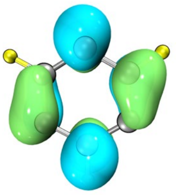<br>α LUMO+1<br>π_5<br>iso 0.05

Supplementary Table 32. Selected NOCV pairs of [(^{Ad}TPBN$_3$)UO]$^-$ (the anionic part of **4**), featuring interactions between the anchoring arene ((C_6H_3)$^{3\cdot}$, ^{4}A) and the fragment containing U(IV) ({[AdN(C_6H_4)$^\cdot$]$_3$UO}$^-$, ^{6}A). Hydrogens are omitted for clarity, except for the hydrogens on the anchoring arene (labelled in yellow). Only α orbitals were shown for fragment orbital contributors.

TOI	NOCV Pair Densities	Fragment Orbital Contributors	
σ	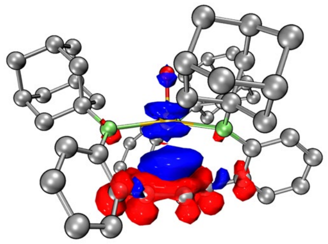<br>$\lvert v_\alpha \rvert = 0.13$, $\lvert v_\beta \rvert = 0.13$<br>-11.0 kcal/mol<br>σ donation<br>iso 0.0005	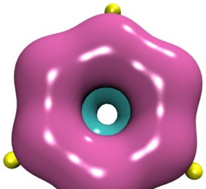<br>α HOMO-5<br>π_1<br>iso 0.05	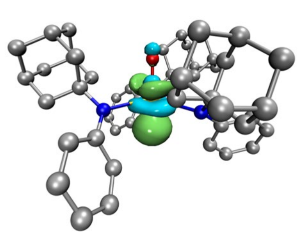<br>α LUMO<br>71% U 5f, 11% U 6d, 11% U 7s, 4% U 7p, 3% O 2p<br>iso 0.05
π	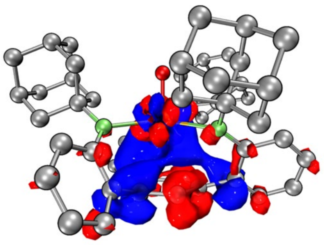<br>$\lvert v_\alpha \rvert = 0.51$, $\lvert v_\beta \rvert = 0.35$<br>-32.3 kcal/mol<br>π donation<br>iso 0.001	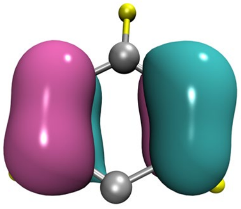<br>α HOMO-2<br>π_2<br>iso 0.05	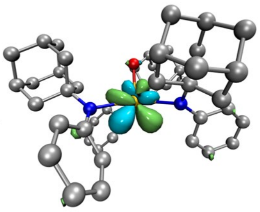<br>α LUMO+1<br>78% U 5f, 17% U 6d<br>iso 0.05
	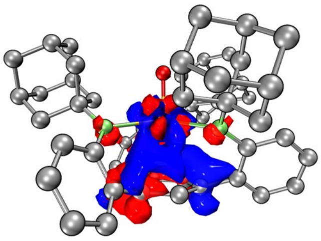<br>$\lvert v_\alpha \rvert = 0.47$, $\lvert v_\beta \rvert = 0.35$<br>-29.9 kcal/mol<br>π donation<br>iso 0.001	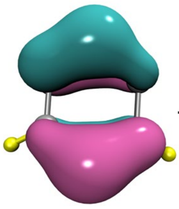<br>α HOMO-3<br>π_3<br>iso 0.05	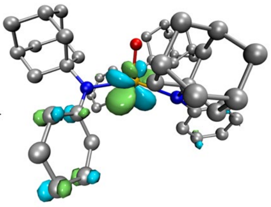<br>α LUMO+2<br>73% U 5f, 23% U 6d<br>iso 0.05

Supplementary Table 33. Selected NOCV pairs of (^{Ad}TPBN$_3$)UO (**3**), featuring interactions between the anchoring arene ((C_6H_3)$^{3\cdot}$, ^{4}A) and the fragment containing U(V) ([AdN(C_6H_4)$^{\cdot}$]$_3$UO, ^{5}A). Hydrogens are omitted for clarity, except for the hydrogens on the anchoring arene (labelled in yellow). Only α orbitals were shown for fragment orbital contributors.

TOI	NOCV Pair Densities	Fragment Orbital Contributors
σ	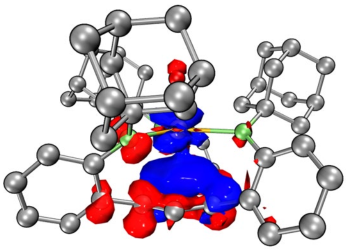<br>$\lvert v_\alpha \rvert = 0.15$, $\lvert v_\beta \rvert = 0.15$<br>-12.9 kcal/mol<br>σ donation<br>iso 0.0005	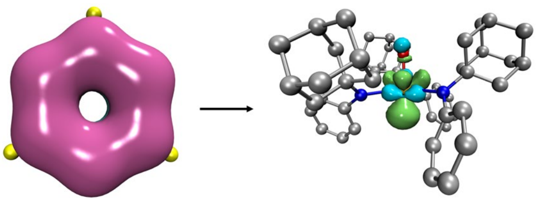<br>α HOMO-5<br>π_1<br>iso 0.05<br>α LUMO+2<br>73% U 5f, 7% U 6d, 10% 7s, 5% O 2p<br>iso 0.05
π	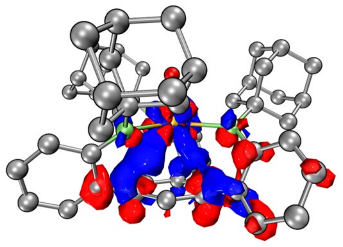<br>$\lvert v_\alpha \rvert = 0.44$, $\lvert v_\beta \rvert = 0.40$<br>-37.1 kcal/mol<br>π donation<br>iso 0.001	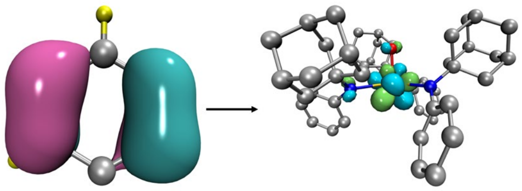<br>α HOMO-2<br>π_2<br>iso 0.05<br>α LUMO+4<br>83% U 5f, 8% U 6d<br>iso 0.05
	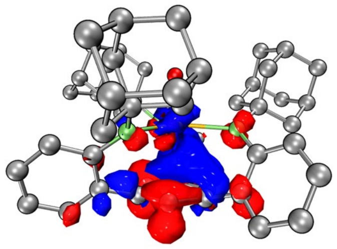<br>$\lvert v_\alpha \rvert = 0.39$, $\lvert v_\beta \rvert = 0.41$<br>-34.7 kcal/mol<br>π donation<br>iso 0.001	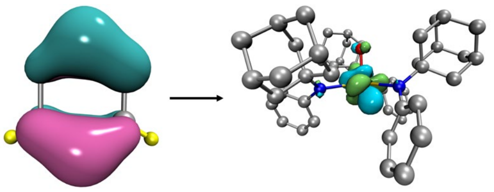<br>α HOMO-3<br>π_3<br>iso 0.05<br>α LUMO+5<br>84% U 5f, 9% U 6d<br>iso 0.05

Supplementary Table 34. Selected NOCV pairs of $[(^{Ad}TPBN_3)UO]^+$ (the cationic part of **5**), featuring interactions between the anchoring arene ($(C_6H_3)^{3\cdot}$, ^4A) and the fragment containing U(VI) ($\{[AdN(C_6H_4)^{\cdot}]_3UO\}^+$, ^4A). Hydrogens are omitted for clarity, except for the hydrogens on the anchoring arene (labelled in yellow). Only α orbitals were shown for fragment orbital contributors.

TOI	NOCV Pair Densities	Fragment Orbital Contributors
σ	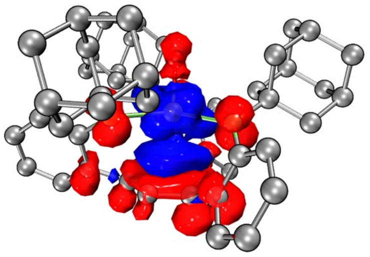<br>$\lvert\nu_\alpha\rvert$ = 0.22, $\lvert\nu_\beta\rvert$ = 0.17<br>-15.7 kcal/mol<br>σ donation<br>iso 0.0005	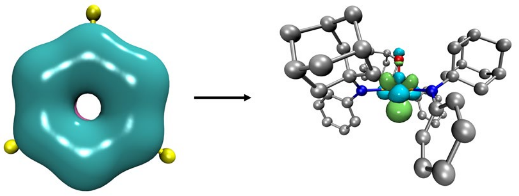<br>α HOMO-5<br>π_1<br>iso 0.05<br>α LUMO+2<br>80% U 5f, 12% U 6d, 3% O 2p<br>iso 0.05
π	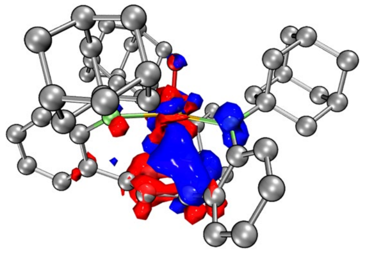<br>$\lvert\nu_\alpha\rvert$ = 0.47, $\lvert\nu_\beta\rvert$ = 0.55<br>-44.0 kcal/mol<br>π donation<br>iso 0.0015	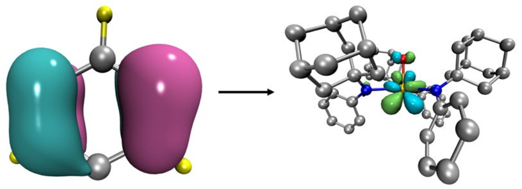<br>α HOMO-3<br>π_2<br>iso 0.05<br>α LUMO+4<br>81% U 5f, 11% U 6d, 4% O 2p<br>iso 0.05
	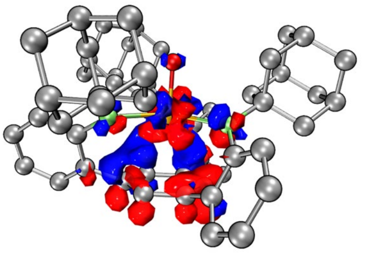<br>$\lvert\nu_\alpha\rvert$ = 0.48, $\lvert\nu_\beta\rvert$ = 0.55<br>-43.9 kcal/mol<br>π donation<br>iso 0.0015	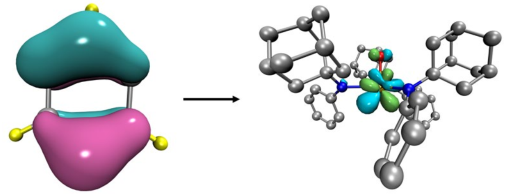<br>α HOMO-2<br>π_3<br>iso 0.05<br>α LUMO+5<br>81% U 5f, 11% U 6d, 4% O 2p<br>iso 0.05

10.8. XYZ Coordinates

Supplementary Table 35. Atomic coordinates for the optimized structure of (^{Ad}TPBN$_3$)U (**1**).

U	0.46986813731512	3.62980097361131	10.99529196584768
N	-1.71237008137376	3.21105932821000	11.85276033872447
N	1.30864261896916	2.36924045493429	9.14182734527081
N	1.66815985079087	5.59290676173528	11.65989494964406
C	0.36021651460964	2.32707441736594	13.35859336417895
C	0.52672823169429	1.31156182409622	12.39253949094965
H	-0.28140909456835	0.61388804248695	12.21564266345989
C	1.66744762358754	1.26681883987346	11.59401107217932
C	2.67568351107983	2.23234961985744	11.78503042881661
H	3.52488532069966	2.24182353797024	11.11398684282542
C	2.53110684899139	3.24833978715402	12.72488110546783
C	1.36959976159778	3.27474790687536	13.52400875518682
H	1.22341511765848	4.09825420753888	14.21100036199042
C	-0.94956047654847	2.49897669575556	14.01089157409140
C	-2.01303006311934	2.93361784808788	13.17687410804610
C	-3.26513630307878	3.06191240513103	13.80792187219408
H	-4.12881251834646	3.38272024014406	13.24843337602567
C	-3.43010655889903	2.80753469689727	15.16093021826293
H	-4.41608150126487	2.93027548361359	15.59659647362770
C	-2.36728621760298	2.41547059454867	15.95884342129621
H	-2.50284989633816	2.22503764630688	17.01608366546112
C	-1.12229456185621	2.26467598336823	15.36335319893900
H	-0.26812178699861	1.94180158012801	15.94922002882171
C	3.44922304696874	4.40072060017224	12.73864915236011
C	2.94466687644815	5.61301488794875	12.19822994534437
C	3.82751876838710	6.70935904759196	12.24659877404179
H	3.52815639323838	7.66957479205609	11.85893377902400
C	5.10854007301024	6.59466979908903	12.76498773754005
H	5.74799131148180	7.47114946839704	12.76955130315805
C	5.58487717937070	5.39093777431994	13.25873686230326
H	6.58914907904404	5.30704921381131	13.65464472387499
C	4.73492172416735	4.29350797102739	13.23783659239804
H	5.06335237461855	3.33691242311698	13.63062492778117
C	1.74651095882030	0.38374418936394	10.41770574400108
C	1.59173236637434	1.01257549700563	9.15326437556861
C	1.72484667612713	0.16219878760673	8.03829869336474
H	1.63194808358554	0.55249406366367	7.03835383771834
C	1.95918807034179	-1.19767787042978	8.17801928200572
H	2.04504165393376	-1.80433127533720	7.28262069523642

C	2.07333634626934	-1.78892824645556	9.42523190073228
H	2.24967788373615	-2.85239603637178	9.52666041649897
C	1.96616942633255	-0.97543000952428	10.54561483057545
H	2.07187246104726	-1.39446046844586	11.54083241260414
C	-2.76919604890405	3.19300776966688	10.84347313694895
C	-2.07699053074379	3.21298162914846	9.47229531902526
C	-3.07330060655368	3.16760993082015	8.31591246871707
H	-2.52627368843558	3.17896271197273	7.36912423794127
C	-3.99089863802041	4.38448236639469	8.39817011584703
H	-4.71039199621594	4.36445277369315	7.57354104121549
H	-3.40667648773918	5.30592947807512	8.29514437632663
C	-4.71567043328471	4.37225076912240	9.74143299914457
H	-5.37410888652433	5.24383797169140	9.81073433882679
C	-5.54317107187865	3.09218002374739	9.86672009060045
H	-6.30476041523529	3.06057007547207	9.08068231161728
H	-6.07064693505106	3.08034043023714	10.82620852550628
C	-4.62822900291220	1.87129366772000	9.75889464239646
H	-5.22470121394198	0.95760210709029	9.84058222872451
C	-3.60015830607806	1.90131832471474	10.88750428215950
H	-2.91321649127888	1.05271372938241	10.80364961565456
H	-4.10058727913178	1.80311175059069	11.85047017196106
C	-3.90282783560948	1.89060088969117	8.41592749806122
H	-3.25584335617686	1.01234228835722	8.32395918084410
H	-4.62078030846694	1.85198674461459	7.59023815777559
C	-3.68760801659269	4.42846639687684	10.87029875272956
H	-4.19704924241986	4.52327719684879	11.82711931779500
H	-3.06186642990229	5.31843158672288	10.76599170191984
C	1.55299735503915	3.15551984900184	7.93335996483134
C	1.41670586608964	4.63642458244598	8.31883901520302
H	2.11189441752756	4.88034302765477	9.13366001788684
H	0.38644698526327	4.83470466615215	8.66741945572999
C	1.67268069636305	5.57259963695412	7.13894921357926
H	1.56153377554469	6.60825859676298	7.47208977245161
C	0.66975199222407	5.27681867518663	6.02770600124956
H	-0.35133631534148	5.46924482440144	6.37611598308275
H	0.85008616549466	5.94194343625928	5.17709958815650
C	0.81209420733154	3.81717296318219	5.60585189200561
H	0.09313612311584	3.59258319576167	4.81163535346713
C	2.23248468371916	3.56437659871448	5.09865175117429
H	2.33829512925947	2.51942338306415	4.78852641206049
H	2.43061236306466	4.18154790184856	4.21605271484869
C	3.24134095815028	3.88765386222517	6.20174817125662
H	4.25662670939263	3.71554782936872	5.83162095531199
C	2.98796113481561	2.98627683792250	7.40820835162926

H	3.67616176783149	3.23473481349751	8.22290105120110
H	3.17953397845332	1.94661684159095	7.14411167998595
C	3.09068042946987	5.34769867881463	6.62145205882333
H	3.28404027582727	6.01303061367321	5.77366618246798
H	3.82010945527448	5.59341595751903	7.39991178243947
C	0.52919449569481	2.91690464040822	6.80720058350936
H	0.51868012245563	1.87560486119839	6.49289667215075
H	-0.46650046474544	3.12572271572457	7.20611000959575
C	0.90292741336956	6.83454740938520	11.54989697705488
C	-0.55441619273113	6.44515352862340	11.25846408484729
H	-0.93333791288034	5.77910828863454	12.04546683377316
H	-0.61002703464682	5.91112311320761	10.29176040075493
C	-1.47130782616995	7.66147783385347	11.14240293666334
H	-2.49104037860847	7.32418443273060	10.93685006205438
C	-0.98219105708358	8.55560761493924	10.00674531100427
H	-1.02861258537848	8.01678783245475	9.05358073976226
H	-1.63382851579927	9.43031820891103	9.91411546630847
C	0.45405781917642	8.98461035401021	10.29452745800902
H	0.81514768584457	9.62464788944597	9.48334840062151
C	0.50624229068133	9.75187870407678	11.61624785675567
H	1.53356199701948	10.06925801946070	11.82403949724026
H	-0.10164627039562	10.66005883518921	11.54665240681142
C	-0.00622265359855	8.86377771926022	12.75063363241447
H	0.02673047296601	9.41798221652307	13.69356719059211
C	0.87705346580268	7.62383312277971	12.86847494956819
H	0.50403664555953	6.95927750707307	13.65502653611956
H	1.88705257405547	7.91217361046547	13.15843566240843
C	-1.44195238752637	8.43692739327071	12.45661804083966
H	-1.82535372666297	7.81346646443156	13.27075505033775
H	-2.09576572029882	9.31211093836462	12.38330070018883
C	1.34101873208922	7.74393614968709	10.38737605466293
H	2.38238031816643	8.04206170338705	10.49114671680570
H	1.27775607177857	7.16727631016566	9.46125403984174
H	-1.50608450179670	4.15306239862581	9.36016865409760
H	-1.37635303945166	2.36985931476090	9.38887258718274

Supplementary Table 36. Atomic coordinates for the optimized structure of $[(^{Ad}TPBN_3)U]^-$ (the anionic part of **2**).

U	-2.51182384945447	16.12827105644388	3.24897968605237
N	-4.12519986611998	16.65486692555586	1.45436380094746
N	-0.11075352008476	16.45379427697623	2.75497802779351
N	-3.30254632503224	16.26260856258975	5.58887033570532
C	-1.28297150871845	13.98683761867060	2.43942573755490
C	-1.39904817074134	13.83795073549113	3.84119228799887
H	-0.50656038772838	13.83337704042185	4.45332240231037
C	-3.83195803350851	13.90796497125354	3.65393156411243
H	-4.80344329082109	13.96118345928665	4.12860018121616
C	-2.67049390879841	13.83694102628222	4.45886677709569
C	-2.79374262482548	13.94375364430382	5.92553083137275
C	-3.97834675144620	17.93255707755331	0.77564455888779
C	-4.01644374014972	17.46407237567187	5.99042737540742
C	-3.73070533019002	14.05879407894743	2.25651401288291
C	-2.55536663901616	12.85158840799192	6.73855456813758
H	-2.26889413589152	11.91772441000227	6.26481050982978
C	-5.43448369419680	17.17801137743038	6.51766548825806
H	-5.39283985197948	16.56069574513812	7.41498103275446
H	-5.96009648219911	16.59059306845808	5.75788000772379
C	-2.44887919501842	14.06311135189083	1.64960750550404
H	-2.36425490414400	14.23021738307142	0.58382307308227
C	-3.16443266829642	15.21196415793286	6.46571080170212
C	-3.32936177844670	15.24404393564193	7.86961635625003
H	-3.62415711947694	16.15575462790274	8.36398334768219
C	-4.91982746117163	14.39355860397618	1.44978901736472
C	-5.06208633849286	15.74640996462975	1.01919039426788
C	-2.69319669147062	12.92494228599645	8.12187847690174
H	-2.50721355866949	12.05971258350232	8.74751859336363
C	0.04012107780788	14.24403567018888	1.83768422179851
C	-3.09568441487103	14.13242632663619	8.66685948825520
H	-3.22555051274097	14.22643935792682	9.74126590045806
C	-6.18377610447791	16.00278007493546	0.19766512311250
H	-6.36506200084338	16.99357659830427	-0.18714593487090
C	-5.11472257130178	18.93857874405093	1.05447813180109
H	-5.19275283140208	19.06559392233035	2.13775188073439
H	-6.07573990100427	18.54878147528013	0.72419205557144
C	0.54521762534340	17.59779407248146	3.36964520103882
C	-3.25112217425596	18.36253432077235	6.98492166888052
H	-3.05253332045233	17.83656159021357	7.91734745880294
H	-2.27452702408544	18.59218821455752	6.55013116096447
C	0.67978724445791	13.27075880294235	1.09339880601793

H	0.17762436167481	12.31703196148757	0.96400351939287
C	-0.49137951686848	18.24946559861044	4.29456989535550
H	-1.33422363627445	18.59190900461969	3.68047739065247
H	-0.86131375903658	17.50314708186672	5.01126828251281
C	0.62537868428375	15.52723058584834	2.05379367204131
C	0.98351764079708	18.70104082575816	2.38471878199057
H	1.71456876860518	18.32480122522221	1.67095337155874
H	0.11074267929819	19.00177037954049	1.79866148548616
C	-2.70218105947284	18.58275236575533	1.32624138973479
H	-2.83762108557416	18.74621110605346	2.40299189891031
H	-1.85174299066355	17.90224222223002	1.18005484496578
C	-4.20260828515802	18.30566879312036	4.72101534322057
H	-4.72877685948395	17.70975134569867	3.96291782964821
H	-3.21003447463328	18.56698787006430	4.33195086369191
C	-7.10598101487170	15.01648284959581	-0.12231963769267
H	-7.95452249053135	15.28107505818239	-0.74683977608127
C	-5.85660969641036	13.42854861607401	1.13073876135385
H	-5.69351904368082	12.41984070615729	1.49738357331239
C	-2.39998200699045	19.93405726766006	0.68787065617019
H	-1.48135301330083	20.34241618519259	1.12125259664195
C	-4.02339037747532	19.64958381121547	7.27347766751814
H	-3.47117043038022	20.24748187296421	8.00719744338701
C	-6.34273906061436	19.27856033792504	5.52106456493961
H	-6.90253067945907	20.20252609036702	5.70729040137551
H	-6.90837387790264	18.69799546629298	4.78502577459232
C	-3.56208765411524	20.88780135713002	0.95205029896062
H	-3.35958217826265	21.86303700985361	0.49446774582236
H	-3.67563678275606	21.05265587399262	2.02938630588668
C	1.91124810181202	15.70514439831365	1.49371482338562
H	2.43332648376276	16.64178667292537	1.61030050123239
C	-4.84153314763797	20.28274621625023	0.37998901070271
H	-5.68268038682837	20.95950258707304	0.56782202673272
C	0.05349806645813	19.45836504544084	5.04627065262308
H	-0.73303670675846	19.87096905794570	5.68597325210894
C	-6.18618726704962	18.47584105429056	6.81094445736160
H	-7.17588267303191	18.23669743047544	7.21544796671404
C	-6.96904965636306	13.72082193818648	0.34657449851081
H	-7.69414743175924	12.95415282887290	0.09937890659511
C	-3.75679192829858	17.78346272567393	-0.73993188318049
H	-2.90276497613931	17.11405529331633	-0.88461984350224
H	-4.61420772294197	17.30110110338431	-1.20884270496062
C	-4.95786049190387	19.60560157469383	4.97092375107798
H	-5.05473049587061	20.15630386419561	4.02978287202764
C	2.54124758256334	14.71099932723634	0.75854010559634

H	3.52484952918039	14.91425982552269	0.34466380824139
C	1.72720709152000	17.19634423418670	4.26906562120522
H	1.36248768167256	16.45524866713700	4.98787791547036
H	2.50230087089772	16.70502439503712	3.68088717625041
C	-4.18612436655482	20.44821443320866	5.98274611486654
H	-4.71780694481666	21.38683316729819	6.17646996870959
H	-3.20224811785968	20.71017648483945	5.57799300968462
C	-2.22062691369421	19.74751500928419	-0.81601192718553
H	-1.36950985256664	19.08671997199941	-1.00932459150861
H	-2.00210948922805	20.71100201414675	-1.29105190527703
C	-4.67651000387064	20.07406002434107	-1.12624152240100
H	-5.59323946204453	19.64597165324990	-1.54672991803370
H	-4.51390342133080	21.03778103086375	-1.62326855339678
C	1.93706190245587	13.48512897959179	0.53601174224339
H	2.42995147624441	12.71210297553664	-0.04197471644766
C	-5.40357715888603	19.30130813890067	7.83378115883624
H	-5.95219899969402	20.21871442255034	8.07766517410929
H	-5.29471446332543	18.73367126615536	8.76449456506995
C	2.30732607171019	18.40991540569171	4.99341744450974
H	3.16413922632848	18.09451820208008	5.59892090693694
C	1.24093773951051	19.02218283123911	5.90014960077578
H	0.91596625504329	18.28777873038657	6.64417596155583
H	1.64798624100371	19.88176367121648	6.44506758736020
C	1.56798449990340	19.90335625947120	3.12670133517550
H	1.89566366488676	20.65300922259891	2.39770415517154
C	-3.49584946512897	19.13879665140327	-1.39504575842304
H	-3.37797639602946	19.00211357551693	-2.47562381208742
C	0.50497278212925	20.51122486114057	4.03826835491282
H	-0.35008772939148	20.85147966194864	3.44327971770738
H	0.90428360968468	21.38756476092163	4.56165891401769
C	2.76220444320573	19.45019662558697	3.96836049169853
H	3.53423626074626	19.02149117260999	3.31990856700261
H	3.21057666145803	20.31085776394701	4.47851816014871

Supplementary Table 37. Atomic coordinates for the optimized structure of (^{Ad}TPBN$_3$)UO (**3**).

U	7.77464719271445	4.14229125578845	7.04542381211171
O	6.13062719377518	3.59935750773214	7.58613693359660
N	8.70292327616050	3.48494076787335	9.07535560958558
N	7.80206766638656	2.71015992170744	5.24697780802041
N	7.16483118517214	6.34286690062352	6.69864529093323
C	10.66768188331222	3.86564644152177	7.21268948364088
C	10.39350995046984	3.56067569760753	5.88781036102536
H	10.48007816702968	2.53788916263309	5.54860220364554
C	9.83551112203433	4.52036092673688	5.02049890533419
C	9.62525757753714	5.80631631904320	5.49286811256773
H	9.11886174021500	6.52757021421040	4.86686966315785
C	9.88350872974311	6.13739518769126	6.83735310370156
C	10.43744957392554	5.17740878999215	7.67027591697881
H	10.56340437831809	5.39232053097869	8.72216585827209
C	10.84086547389785	2.78094106779738	8.19562270218608
C	9.78586547524976	2.62566025914842	9.13748445317455
C	9.88988765045712	1.52901730668592	10.01002606293936
H	9.09379250295116	1.33551951450501	10.71377440240357
C	10.96297196211131	0.65598910108397	9.95542931336658
H	10.99577511607263	-0.17963921968984	10.64612474056885
C	11.97490665253265	0.81863819463414	9.01907404684623
H	12.80617976644541	0.12620718167729	8.97449641856423
C	11.89417805041807	1.88248478255829	8.13296587500250
H	12.66650783006298	2.03240757218860	7.38588044515484
C	9.15747302049590	4.07946484063033	3.78697815246555
C	8.09736222882415	3.15151761069871	3.96419945634396
C	7.35636959135183	2.81959277739329	2.81877504959042
H	6.50783434115516	2.15846310132800	2.91488022797673
C	7.65509326933815	3.36111979517226	1.58059026631753
H	7.05274493063820	3.08114006418281	0.72331206081761
C	8.69252668310768	4.27144262123533	1.42895101877705
H	8.91619559214374	4.69906204685721	0.45951160788075
C	9.42968518124506	4.63459760244190	2.54572986540670
H	10.23837436946407	5.35227725903919	2.45842222624304
C	9.24946849296013	7.33759395143014	7.41175698072657
C	7.83255992397005	7.39096803218611	7.31635614222234
C	7.20671163360613	8.48053848288085	7.94335321580993
H	6.12928156223958	8.54817987704309	7.93640543360376
C	7.93526990810098	9.44979740720846	8.61159483721465
H	7.40759058198570	10.27133397591981	9.08372353589052
C	9.31799062948306	9.37186443907760	8.70425054330876

H	9.88180314698974	10.12771552527620	9.23658782257050
C	9.96425168710204	8.29973466412777	8.10778121673670
H	11.04366883527365	8.20925478010335	8.16680449321052
C	8.00889781110301	3.92446467030572	10.29278967353785
C	7.39607183937888	5.29718901904859	9.96585721513482
H	8.19283441861168	5.98480345850401	9.66634805110623
H	6.68349244892358	5.21273806798171	9.13607158309302
C	6.63074671015211	5.87584489931514	11.15235993896656
H	6.20637918477038	6.84098739938739	10.86065574387761
C	5.51562582627900	4.90965380691604	11.54729454574735
H	4.95188662559589	5.31639096771559	12.39349522552106
H	4.81379513629723	4.78812148847206	10.71600449766343
C	6.12372353351638	3.55783119136050	11.91963895588238
H	5.32714242397372	2.86598753483718	12.21106039097144
C	7.10064520333674	3.73625981240901	13.08203766239277
H	7.53500004768107	2.76879045726158	13.35640182393885
H	6.57519324456982	4.11612419922959	13.96461658451003
C	8.20918979111407	4.70902881779642	12.67746172140671
H	8.91015946534082	4.83162516733170	13.50917903325295
C	8.96512632661108	4.14720828055487	11.47213131788961
H	9.75425075172077	4.83973475036830	11.15894573943650
H	9.45748462714795	3.21586288574513	11.75407723139121
C	6.87083454053308	2.98406383623007	10.71616269418902
H	6.19682062070312	2.85257613739047	9.86693068687541
H	7.27127240599458	1.99945586790635	10.96775218201980
C	7.59425548984492	6.06092155760409	12.32063787057928
H	8.37888722389371	6.77436308674583	12.04810172528703
H	7.06415888236706	6.47513273609754	13.18476896935475
C	7.41352506668339	1.31109665763998	5.48858866443426
C	7.86446153856764	0.96516007606201	6.91823592042330
H	8.94670003426334	1.10336915727165	6.99896361279338
H	7.37886086753825	1.62375742829374	7.64778537917605
C	7.50107488029320	-0.46876429425796	7.29280823598249
H	7.83061332855999	-0.65510027475129	8.31882337994260
C	5.98800349459626	-0.64749948261675	7.18596309848276
H	5.48083556418644	0.02939666183858	7.88084552557736
H	5.71083424484029	-1.66949025581552	7.46521876923582
C	5.54247493752594	-0.35135269434953	5.75448518409415
H	4.45919224814872	-0.48572689756766	5.67287844051157
C	6.25274537643978	-1.29825869338191	4.78719514063931
H	5.93043717745099	-1.09376008949467	3.76033601122048
H	5.98327860946661	-2.33658277409188	5.00759356949420
C	7.76590825753377	-1.11373372262422	4.90719189043390
H	8.27382949170008	-1.78736717693781	4.20978296989822

C	8.13284301238351	0.32846899677482	4.55530479822194
H	9.21409876771999	0.47953689627046	4.64749622598334
H	7.87790703451374	0.52366241013943	3.51311923582974
C	5.89558393870526	1.09211553107137	5.40045830720086
H	5.40411547867256	1.78980595276647	6.08273086859817
H	5.54103360386282	1.32068031524888	4.39269476312434
C	8.20496630078279	-1.42586979600968	6.33622085132989
H	9.29047605003783	-1.31719635440428	6.43092924595393
H	7.95987732808730	-2.46261776972015	6.59027608713967
C	5.95875150837967	6.58603070974814	5.89458009876630
C	5.87132453511949	5.43581122506380	4.87989543652969
H	6.78044388913519	5.42279639427119	4.27186533641603
H	5.78602285942392	4.47112051988037	5.39565082526375
C	4.65204904249419	5.56803420911506	3.97213851073451
H	4.63954591856583	4.72065030490563	3.28063665616802
C	4.74930736284808	6.87589452512943	3.19304002210338
H	3.87990831593348	6.98877237413781	2.53663706983144
H	5.63821776848380	6.86623284136545	2.55356011996669
C	4.81950334945176	8.03840778801122	4.18081855550438
H	4.89964000818831	8.98295450013010	3.63351139065540
C	3.55621695082363	8.05119198124980	5.04247060287754
H	3.58947645872220	8.89256206938138	5.74310782154276
H	2.67388836481890	8.19384854006869	4.40974413952724
C	3.44565648758443	6.73482861597864	5.81210242853665
H	2.53913515123845	6.74222798561780	6.42544474089431
C	4.66350554313833	6.57539845712971	6.72296870761511
H	4.61187571376094	5.63329745393288	7.27492642700328
H	4.67691538368920	7.38072361798825	7.45971064176431
C	3.38743007457780	5.56767769822198	4.82776491730773
H	2.50193374674239	5.65434967121724	4.18939370211953
H	3.30481020872005	4.62260852080664	5.37388665616581
C	6.04712942828156	7.88218682744339	5.07898282279211
H	6.13242319714693	8.74978768949477	5.73454850014196
H	6.95969179424076	7.85513133687915	4.47339095413101

Supplementary Table 38. Atomic coordinates for the optimized structure of $[(^{Ad}TPBN_3)UO]^-$ (the anionic part of **4** and **4′**).

U	4.11391618475319	10.55095021021333	4.12293000421973
O	2.78897362568265	10.58620528777457	5.43979442222035
N	5.62693902683541	9.16668910115009	5.41110605742298
N	2.76584139713033	9.42922338150450	2.40495906383040
N	4.33224536377350	12.96697883081682	3.96644880056071
C	6.94155091350791	10.36294611081421	3.31784415452344
C	6.38914093289879	9.21879220992758	2.71167475516081
H	6.61600587538990	8.24771700930283	3.12855113111044
C	5.41953492391975	9.32674366998338	1.72659963056905
C	5.05692810812998	10.60841865085128	1.27308277634194
H	4.23711316066538	10.69766717433921	0.57402751407537
C	5.58982544641135	11.75462659757337	1.84414402219090
C	6.56103655250438	11.61633920480311	2.85519272079068
H	6.90729633107517	12.50102079029481	3.37137148256855
C	7.63887075836973	10.21865368160002	4.61181709344701
C	6.90657746432074	9.57452053717968	5.66211018051484
C	7.57776188193419	9.48180698628457	6.90178308472347
H	7.06364263890518	9.04198552183328	7.74435979356189
C	8.85163603669451	9.99037695999618	7.08795765132035
H	9.31413785986389	9.90056306906383	8.06641643008713
C	9.53164465788834	10.63077552327075	6.06019643437592
H	10.52440870618000	11.03641955522454	6.21435364426270
C	8.90146190792369	10.74366697304277	4.82703527486280
H	9.40655507083020	11.23155294007537	3.99895867461900
C	5.04384964118857	7.99304942391663	6.05162146029816
C	3.93172419674321	7.50590433796930	5.10631009683352
H	4.36585837326747	7.29581910856638	4.12449119103420
H	3.17496492858585	8.29056462609240	4.98841744807209
C	4.39570999869212	8.28723594950914	7.41463471392041
H	5.15228662981203	8.63043823672849	8.12517867099172
H	3.68032495886048	9.10294088398446	7.27779767822691
C	6.03642356443610	6.82919222908119	6.19053108572353
H	6.45863616764963	6.61517485180336	5.20250288430956
H	6.87236020561822	7.10583932200795	6.83525187976051
C	5.34246202233007	5.58685263662215	6.75041959429015
H	6.08016332361875	4.78403089484516	6.86296948243034
C	3.22336354638412	6.26678005505292	5.64157523684506
H	2.44280049460239	5.97227861712077	4.93346258442420
C	3.70527948150426	7.03778408502805	7.96278028685141
H	3.26484307793327	7.26625578597526	8.93993785556851
C	4.73222217698034	5.91454449090544	8.11438254203298

H	5.51997635213866	6.22089782879818	8.81240068660300
H	4.25691665521690	5.02058323862591	8.53562986793086
C	4.23831721612357	5.13759288423612	5.79484918134994
H	3.74919377479410	4.23429821110702	6.17894937596717
H	4.66559312015523	4.88788465691571	4.81792328818900
C	2.60473598707585	6.59420886704291	6.99910653269601
H	2.08488067577282	5.71535293376089	7.39927925735694
H	1.86465299695288	7.39262572051854	6.88674099477951
C	4.55685087110379	8.17273724958565	1.40387542108511
C	3.18049073966082	8.28456696298957	1.79009540607165
C	2.39165345337216	7.13748952119389	1.54221577070977
H	1.35773546351684	7.13373664638849	1.85531822721834
C	2.91478609066755	5.99145822692566	0.96798706601343
H	2.26019809486988	5.13873794679975	0.81324912591095
C	4.25405634265714	5.90616473572699	0.61293109981708
H	4.66074529530293	5.00384092023614	0.17225970290656
C	5.06440467538944	7.01066218786761	0.85133903695157
H	6.11780667043758	6.98054799723027	0.58948771140194
C	1.39645221394755	9.91910954447283	2.28218235775932
C	0.82228100483673	9.75316185090185	0.86624691052758
H	1.50706930411970	10.23468102474797	0.15938249865108
H	0.77340436819062	8.70051503984384	0.58524835065797
C	0.42899328378341	9.30987383218229	3.31248605519810
H	0.35087453414435	8.22984631946080	3.16408997984969
H	0.85016652808132	9.47370980884835	4.30817854154498
C	1.45277783012657	11.43613188650128	2.54467786745287
H	2.14589743490456	11.89429102663617	1.83219342380593
H	1.82436170551474	11.62785952676880	3.55728259505399
C	0.07765490376599	12.08765743318060	2.44410173422910
H	0.17962131247124	13.15810660187271	2.64858523703011
C	-0.56986728817719	10.37713745010954	0.76326076656350
H	-0.96248969872165	10.21260218594907	-0.24679954842370
C	-0.95545254175239	9.94730309947476	3.19673460328740
H	-1.62929226717681	9.48706708749460	3.92824462160594
C	-0.85127851454993	11.44664946154194	3.47394555350737
H	-1.84378889436020	11.91124084324534	3.42848483572854
H	-0.45596483088734	11.61017941503955	4.48125418679681
C	-0.48383181292905	11.87641398897320	1.04168294195221
H	-1.47551627908727	12.33698038014958	0.95567192546231
H	0.16534360540119	12.35932550248477	0.30355239782410
C	-1.50171739278704	9.72426057653647	1.78574805187444
H	-1.58471345698097	8.65001388340882	1.58379227114685
H	-2.50919639274694	10.14832508763839	1.69689222132090
C	4.91732174674028	13.05219571938827	1.63433885883584

C	4.27175427132603	13.62916664943526	2.77413566441950
C	3.55880839752634	14.82440239605627	2.52677778223623
H	3.00603401490837	15.28141110093776	3.33517427401963
C	3.49158801053697	15.39526272052353	1.26777458038312
H	2.91905046346841	16.30868636273149	1.13629800973728
C	4.11759339554693	14.80928858204093	0.17528180654810
H	4.05432626123780	15.25440373313250	-0.81051302209992
C	4.81795618155682	13.62647606751638	0.37842485206509
H	5.31593125691433	13.13818761711930	-0.45382048061579
C	4.35842089159231	13.67980081311384	5.23975984833031
C	5.07653851239492	12.74812928391963	6.23504555625334
H	4.51641257909884	11.81060825415739	6.33323896492847
H	6.07671806274019	12.52009659408006	5.85278594501199
C	2.95852845469922	13.97158362977483	5.80648127966535
H	2.41329975773447	13.02498364621215	5.85424572020725
H	2.41166651035688	14.63286673190536	5.12923542926608
C	5.17968742073449	14.97678133638362	5.19370960577985
H	6.18101340362299	14.73901444781256	4.81765432672074
H	4.74440409361161	15.68867149707273	4.49129421389743
C	5.26715358021233	15.61622499286375	6.57992539187392
H	5.83440882943738	16.55134135789358	6.50833445189745
C	5.17379821834441	13.36358818655021	7.62739355252321
H	5.68037967189769	12.65744072248981	8.29259756443624
C	3.05950367206389	14.61209598030459	7.19049642911426
H	2.05247540633668	14.82593301035883	7.56622856625049
C	3.85687380800075	15.91322474903322	7.09375305548109
H	3.91043765146076	16.39929431790637	8.07536413297003
H	3.35207012530432	16.61057308936904	6.41531389794059
C	5.96753636218629	14.66410827031871	7.54744811687128
H	6.04727853195662	15.12265304972596	8.54044944806754
H	6.98574875725192	14.45719124484052	7.20102181399977
C	3.76506800637508	13.64894307238064	8.14543534606691
H	3.81410260516608	14.08073181833016	9.15237753768037
H	3.19891033952226	12.71488384877627	8.21309985689605

Supplementary Table 39. Atomic coordinates for the optimized structure of $[(^{Ad}TPBN_3)UO]^+$ (the cationic part of **5**).

U	5.00474504459003	5.19985082387483	3.84744697174605
O	5.21558550611535	4.00867809075660	5.18030454457539
N	3.41735701152561	3.94683898180719	2.86656118857094
N	4.42879534381872	6.80173986144618	5.30979121473597
N	7.15410625856725	4.98499268594351	3.21531707011172
C	3.37271103823921	6.45780229157737	1.86342139109704
C	3.11376968878277	-0.31626866604976	2.23669638072902
H	2.02810573121307	-0.29939979777356	2.37769019168605
H	3.39271740182073	-1.35511269056824	2.03797022130809
C	3.68729377383153	7.49795628459994	2.72324057764257
H	2.91981237229656	7.92609861563291	3.35294384126557
C	5.02642103845754	7.90332812357115	2.90875709518766
C	6.02329697611692	7.29621516908833	2.16211854874927
H	7.05898002235455	7.54915014159383	2.34117819737849
C	5.72388383048099	6.23449602319374	1.28197231639022
C	4.40307011097556	5.85227512435702	1.11277555020618
H	4.16573296345399	4.99758253083110	0.49468919062604
C	2.08272395923731	5.76177228641359	1.99243368143743
C	2.15627396402696	4.45738755452174	2.53207698965632
C	0.94944068977057	3.79763806819733	2.78872920790980
H	0.95934461912599	2.81845490626978	3.24261613228137
C	-0.26396015026294	4.40167107894918	2.50836877347308
H	-1.18147610156013	3.86593951224085	2.72163845731658
C	-0.31923138525437	5.68410081219498	1.97863320490174
H	-1.27312136034990	6.14965853009196	1.76549677826737
C	0.86187268713086	6.36616279426289	1.73446491084584
H	0.84247273048064	7.37159661553370	1.32959266160609
C	5.38005178541823	8.67451110204291	4.11171479619265
C	6.09350769294102	9.86288724254555	4.07290035411449
H	6.32722413754249	10.31034501919390	3.11353851180883
C	6.49005213735134	10.47798744493736	5.24951506623618
H	7.03516403444519	11.41289743634450	5.21999850183179
C	6.19054112373067	9.87504026558189	6.46412311318338
H	6.51449683198264	10.33554336381936	7.39002308637019
C	5.49811121974112	8.67761700207480	6.51287708610374
H	5.32138643588338	8.20798150024729	7.46870275425943
C	5.06426842052182	8.05031001980660	5.34004268902932
C	6.80676351856644	5.33818974296964	0.84781867277523
C	7.51524957657477	4.69974440442782	1.89159034929761
C	8.46902061643283	3.74219196581264	1.52912510758059
H	9.00001907037347	3.19812466457708	2.29527702667859

C	8.71334903515650	3.44924314944153	0.19877702059442
H	9.45672091086054	2.70049245286772	-0.04844539810134
C	8.00812630533907	4.08488884216897	-0.81479419329711
H	8.20303057635583	3.84664220215919	-1.85275208707677
C	7.04369775511286	5.02191994064111	-0.48132342067343
H	6.47519436348627	5.52446291122961	-1.25560378767465
C	3.73280703875866	2.52667648203663	2.59847573257853
C	5.24857291211961	2.45191233258056	2.37112112143280
H	5.51723158365732	3.07831328355992	1.51611846784607
H	5.79866460378223	2.80922470221447	3.25188881383003
C	3.37714203395604	1.62062484625387	3.78729093249970
H	3.87181950694299	2.00291590113992	4.68298916003616
H	2.30262682037537	1.64482146598740	3.97553830248443
C	3.06500394092318	2.00490556799527	1.32115365728208
H	1.97885877894434	2.04361025557946	1.40463230994378
H	3.34014871495578	2.65226887771698	0.48123398055616
C	5.32920108758438	0.14466059835923	3.30146496486567
H	5.83424070649816	0.50155395064730	4.20480895026089
H	5.66081840293861	-0.88339319968438	3.12972641748987
C	5.01543988233073	0.50875786812997	0.84692174744902
H	5.32962364887639	-0.51794911505397	0.63778668983344
H	5.30878046254833	1.11655354306985	-0.01535203539975
C	3.50301613994466	0.56431474494376	1.04924836956866
H	2.99614816408303	0.21110232266336	0.14712623157902
C	3.81497065636875	0.18478785994158	3.49906362458718
H	3.53943312610348	-0.44461244367811	4.34973023877579
C	5.70595054049727	1.01871897892350	2.10827295418289
H	6.79012168497697	1.01969781310617	1.96801611779070
C	3.34453624976647	6.50358975936207	6.27113640258269
C	3.87017940355692	5.85490195765646	7.56082096916177
H	4.53264059054342	6.54399787658609	8.08716019114592
H	4.45995118458524	4.97273953841311	7.30168000177671
C	2.50645238542163	7.74037804113786	6.61526055924458
H	2.13004993176254	8.19126944924809	5.69050269075201
H	3.11577309218822	8.49800852120363	7.10794771908239
C	2.40801479561841	5.50581925090528	5.57676555982187
H	2.01055694132771	5.95800820009720	4.66402900870369
H	2.94311652231117	4.58660086321438	5.30428488404961
C	1.24855036729024	5.09900465500787	6.48317585039610
H	0.62354300629206	4.38052624737098	5.94596575210353
C	2.70106542652424	5.47571116181813	8.47019428199131
H	3.09965154331248	5.02967259664411	9.38558114726400
C	1.34433682513467	7.35129924875177	7.53116877534744
H	0.77606380887196	8.25318681982271	7.77419342807606

C	0.43804778936910	6.34501940123356	6.82591862143112
H	-0.40439088783427	6.07948291796041	7.47125543674677
H	0.01912741336709	6.78258032660811	5.91373990061746
C	1.80437916697103	4.46620801683896	7.75585655365262
H	0.98286218298687	4.16078293039284	8.41036876675680
H	2.37435871861922	3.56402364358971	7.51100762401582
C	1.89638115936360	6.72896917602265	8.81379730166927
H	1.07423373168679	6.47492390126258	9.48909323628657
H	2.53061155078814	7.45016362379531	9.33939151505508
C	8.20313935489621	5.13395196398138	4.24746970937036
C	7.64229401790999	6.09783542625406	5.30131422337203
H	7.41427638376939	7.05584979884088	4.82598952048285
H	6.72103010281942	5.69990832638463	5.74590243195769
C	9.48528083096839	5.77006094048778	3.69763007910998
H	9.23757309401155	6.72512416413079	3.22164670873489
H	9.93320611671029	5.14141324874367	2.92832713863699
C	8.53236397398340	3.80251057635761	4.94021003627008
H	7.60868007102881	3.36856139353269	5.33034756023641
H	8.94451785961300	3.09148010811289	4.22218650595844
C	9.54160861698869	4.03437205133292	6.06494340126166
H	9.77186820580264	3.07340738071106	6.53324632294889
C	8.63150423498966	6.32167908169096	6.44265616124608
H	8.17881686193679	7.00337007267178	7.16767324540649
C	10.49192429712080	5.98691387011879	4.82892739783492
H	11.40238772460776	6.42008035617667	4.40593241566347
C	10.81722340934973	4.64480788766588	5.48471931282133
H	11.26024582954807	3.96374455071047	4.75066039533781
H	11.55951457018673	4.78629994155492	6.27566714872882
C	8.94367093366071	4.98150323071390	7.10326326923734
H	9.64613304370415	5.12686367802740	7.92898556959963
H	8.03178944424618	4.54904419331649	7.52778623863142
C	9.90550526173949	6.93578225664727	5.87156125743443
H	9.68359931956754	7.90790419515316	5.41930971450274
H	10.63083379104644	7.11117011985669	6.67146076907498

Supplementary Table 40. Atomic coordinates for the optimized structure of (^{Ad}TPBN$_3$)UF (**6**).

U	5.33146891421124	11.68205924466701	14.76049257687228
F	4.62083967470241	9.83061571929113	14.02777915396451
N	3.45749795868335	12.83591537921427	13.98068978769429
N	5.32782621710577	10.79068483576315	16.89145022348195
N	7.26123979169634	11.68635852254524	13.49345595784024
C	4.77416715800166	14.28950044393212	15.88014749886573
C	5.38370507834621	13.61093072809055	16.92364334527145
H	4.79652250796372	13.30910925158052	17.77925963212102
C	6.71303870937602	13.15376705251851	16.81817162150892
C	7.43351470780048	13.45414698226794	15.67227432898930
H	8.42420924804518	13.04275487327832	15.54047581289332
C	6.83694193724479	14.14326967475095	14.59673526195024
C	5.52961331534512	14.58236573430239	14.72796111819869
H	5.03400148465607	15.03754081857281	13.88222003592320
C	3.30541685663280	14.40069216365811	15.81570980710589
C	2.67116885784573	13.62922475979766	14.80326008979671
C	1.26779140682667	13.67679387062188	14.77746721437871
H	0.73097764991287	13.08556268053129	14.05133826755725
C	0.54761782274090	14.42851527108303	15.69105756087189
H	-0.53517482504809	14.42738055839505	15.63443387790140
C	1.18685558421028	15.15678694149477	16.68436816430691
H	0.61854120531610	15.73426664303951	17.40220268397048
C	2.57252466252584	15.12753660651079	16.74031372129132
H	3.10150509090458	15.68971526070842	17.50213883465759
C	7.18744015238796	12.09273472627664	17.72837492839184
C	6.43568210017042	10.88673547630079	17.72262346565817
C	6.93421501798755	9.83446603290179	18.50638006929574
H	6.41898330469054	8.88506521349563	18.50021398701127
C	8.09354875064616	9.97029685175335	19.25040321206110
H	8.44429968858964	9.12985062035686	19.83868965171284
C	8.82166252879744	11.15285367362219	19.23163310631112
H	9.73329155662290	11.25055195840107	19.80719882260406
C	8.36405144284315	12.20550288891410	18.45412433422172
H	8.91485441485559	13.13892727289129	18.41717623243196
C	7.46009879507427	14.08152832203885	13.26103440906969
C	7.65149226338114	12.77913747005533	12.72911407209705
C	8.14979509843539	12.70447330217581	11.41945484605978
H	8.27235692002080	11.73649775232130	10.95670333840499
C	8.44521106742749	13.84420221115424	10.69038218733751
H	8.82446936638552	13.73994317087755	9.68011367186545
C	8.24033594327494	15.10898404129583	11.22477440396511

H	8.46499591985260	15.99687133768916	10.64759255690204
C	7.73294535953963	15.21512036100606	12.51098514518695
H	7.55778565583709	16.19024812488303	12.95201022883091
C	3.12917861147603	12.68949584044523	12.55679048303574
C	2.12638757729944	11.56071713514076	12.26489382162431
H	1.16858723288359	11.76925422206807	12.74389542303065
H	2.50837455128054	10.63367761615023	12.70087661637806
C	2.63131760416241	13.99974902755347	11.93348233674063
H	1.70162168449251	14.31664336612815	12.40757975070479
H	3.36913911315657	14.78483154937727	12.13024508166222
C	4.44081179185841	12.33042021044397	11.84496352467642
H	5.18061717103264	13.11352593063940	12.03742407868955
H	4.83771571917073	11.37920909979785	12.22285957196515
C	4.25343886785287	12.16347371250467	10.34063533048542
H	5.21769544038309	11.90703966819345	9.89321415718212
C	2.41623951580338	13.83651777461911	10.42937792242700
H	2.03947210045622	14.77860065062116	10.01928725144682
C	1.91652334237698	11.40782096901332	10.75735178193539
H	1.18698757550964	10.61275011121006	10.57546537309234
C	3.24156261797907	11.04830801818507	10.08663214091650
H	3.09537102261508	10.91335301802299	9.01001332750579
H	3.62000754316861	10.10173058830513	10.48477690249150
C	3.73920919547973	13.47490797415003	9.75628480711275
H	3.59930894062096	13.37585632766233	8.67493428784455
H	4.47536673755123	14.26917094557986	9.91648036334495
C	1.39531257440406	12.72510297430445	10.18129776286460
H	1.21197578624312	12.61904564347319	9.10716050256072
H	0.43906301636354	12.98527160217493	10.64722809742348
C	4.08845327418839	10.15860893963379	17.37333490061098
C	3.84861974193059	10.40721964299847	18.86765479687230
H	4.64660984905684	9.95965558214401	19.46173802203326
H	3.88142569819075	11.48509668117532	19.05929007055562
C	2.93036116919185	10.83087790662777	16.61943141176084
H	3.02560817387244	10.66933835786220	15.53935966542832
H	2.95359907480315	11.90773541048456	16.80765982613834
C	4.01857686609176	8.65050652806983	17.08603865997504
H	4.17630657180591	8.49166145596056	16.01703882380434
H	4.82051510161301	8.12893248149012	17.61292377560799
C	2.66624433781027	8.08450486769192	17.52472581088690
H	2.64557028971452	7.00929457197787	17.32212622826336
C	1.57427366131736	10.27162177887476	17.03801165697457
H	0.79425393025884	10.78266005081022	16.46698721246368
C	2.50070602749155	9.83300981727728	19.30284102770438
H	2.37045158347206	10.00770069866307	20.37530748223213

C	2.47478155075738	8.33021022947038	19.02155409754537
H	1.52300608385043	7.90373427376962	19.35454598020230
H	3.26752895759617	7.83009847791507	19.58791243491301
C	1.54408438686952	8.77254604422411	16.74836559199266
H	0.57425264663330	8.35504704891754	17.03783059839627
H	1.66805277619643	8.59663141804453	15.67534329824962
C	1.37440761413662	10.51693798748225	18.53039268549880
H	1.37422048406313	11.59226954972948	18.73505499466751
H	0.40338407365505	10.12562228537043	18.85068349974257
C	8.09316456734655	10.47357331303807	13.53382709774876
C	9.59386672923575	10.78127217207182	13.45843988924710
H	9.84908742887128	11.49253170499992	14.25124925536930
H	9.84026041590475	11.26581309330709	12.51299798509773
C	7.71929371693177	9.45206953824333	12.44860816018068
H	6.65153636222405	9.23165623244659	12.53081694681278
H	7.88313760138973	9.87840294182857	11.45688216701512
C	7.84581332927401	9.81405779519292	14.89912418651919
H	8.11097130357537	10.51889447542505	15.69203839329084
H	6.79033435287510	9.54150976442989	15.02175643721966
C	8.65493899970431	8.53117153552872	15.06913559083512
H	8.43280121960606	8.10715301481645	16.05210848612868
C	8.54777725292754	8.17603789100919	12.60474687350702
H	8.27451622443799	7.47230548556851	11.81259666659084
C	10.41589878789306	9.50104278632176	13.60728488666600
H	11.47814117453996	9.75124202013373	13.52595711525123
C	10.13973632092055	8.86616177680719	14.96847328281447
H	10.74197109168658	7.96046354930727	15.09317706689360
H	10.42067761324654	9.55623816865134	15.77033120533332
C	8.26621430370901	7.54643994330596	13.96854701389372
H	8.83434367000555	6.61726514262900	14.07899969580942
H	7.20529779251238	7.29110983962150	14.05120470840483
C	10.03410514755389	8.51921043779681	12.49859186633131
H	10.63618970177138	7.60887185813286	12.58348498276936
H	10.24971927180082	8.95832463192667	11.51878754377055

Supplementary Table 41. Atomic coordinates for the optimized structure of (^{Ad}TPBN$_3$)UI (**7**).

U	14.99053936252168	6.95293012755690	13.40414415131692
I	13.17467611044849	5.24677849786744	15.18914666976061
N	14.57537672415311	8.80963641249901	14.69110831148285
N	13.69821227597577	6.53112230253831	11.53742854356840
N	16.85808904144497	5.66736004729811	13.82792805499476
C	12.15060948680243	4.53285251722290	11.57800554716981
H	12.04263543780548	4.68000358106437	12.65309503454047
H	11.32277950852131	5.05421432403196	11.09537738961781
C	15.64292471775312	9.49004118065183	12.17471732160154
H	14.94869151147769	10.27611512480771	12.43193551410865
C	16.72464364158914	9.22839747021449	13.03887238323742
C	16.75185386511294	9.83589883667015	14.38238698379489
C	11.01810790872106	10.88544082046162	16.07390945935273
H	9.98022817907304	11.23103232056543	16.11965850698496
H	11.55190981211431	11.35679093261103	16.90573621262085
C	10.86670130216485	10.66310674101574	13.59523835398713
H	9.82728221576181	11.00622163219834	13.61089464797393
H	11.29265644386346	10.96104375212744	12.63171914162070
C	13.10651345794396	10.83787198897148	14.70140616621934
H	13.56530267075425	11.14139571262530	13.75489247032197
H	13.67365012618885	11.32481873653684	15.49777192170594
C	11.07520620000594	9.36438088817702	16.21025167400685
H	10.63418932236348	9.06171504750653	17.16482775961896
C	12.07652618802724	3.04803341286681	11.22609913704611
H	11.11460550592932	2.65084937253411	11.56410258525227
C	14.02944492818636	8.68453161930115	10.47696524698487
C	10.92210951030283	9.14417553379488	13.73510914758556
H	10.39453657872520	8.67289126158607	12.90158749049024
C	17.55688985331892	8.15708530353861	12.75885032840309
H	18.34141714701526	7.89696452916736	13.45429292396486
C	13.62528148650643	4.93685248269784	9.60587053969832
H	12.84280654016859	5.49132879348478	9.08352320459103
H	14.58369330395049	5.34814382252548	9.27222129272545
C	13.49119305296577	5.12980235625086	11.12259529161925
C	11.64796756108634	11.30089901977907	14.74405404483758
H	11.62047345754546	12.39072172263359	14.64829163021490
C	14.55012127703769	2.84731930187805	11.43810197888247
H	15.37421325712366	2.33180462409274	11.93834564994331
C	14.62507981262777	4.33126851515190	11.77247923502354
H	15.58219841125990	4.72693212958183	11.42102775056951
H	14.58488210734525	4.42032415663079	12.86754468412910

C	11.89760470233680	7.61338378288732	10.22984014084145
H	11.21895873457459	6.80052456448611	10.43659776005698
C	13.20401209002108	7.55895046133062	10.73529861455331
C	17.29198973644668	7.29259399833333	11.67867179905242
C	18.80395468051939	5.55645324932068	15.40401184310794
H	19.30516773726105	4.76460075531038	14.84362265526951
H	19.15305477785099	6.50850738088331	14.99055873428854
C	13.54049228685611	3.45162705810190	9.24481175454200
H	13.62600787835040	3.34708063462143	8.15882847181182
C	13.19355246749614	9.31278620265658	14.84637228333639
C	15.36451382513045	8.65857896375753	11.10193141174702
C	16.22299178155546	7.57291930017352	10.84417293865618
H	15.96071030494702	6.87466103232824	10.06272881949870
C	17.87034836434929	10.49104136237426	14.87830282229049
H	18.71925072025168	10.65023615866900	14.22277573906445
C	15.69096194552637	10.03826984740451	16.52500990566360
H	14.87593019484675	9.82175361689118	17.19834104906980
C	10.30488050887603	8.71363115770305	15.06324922100883
H	10.34457285437882	7.62449061487774	15.15826380288311
H	9.25103271473827	9.00821762365067	15.10030114386482
C	13.21050266909314	2.29595528661058	11.92038882872052
H	13.12724027933355	2.41309749679204	13.00483218253856
H	13.14445924286162	1.22575609611233	11.69932033566362
C	12.19924507381870	2.88484737348748	9.71154742809551
H	12.12578752617839	1.82753216234208	9.43661004200882
H	11.37649959232505	3.40572506381199	9.21055144686073
C	12.38167941835569	8.70636513360404	13.69798667518602
H	12.81761846798882	9.02350148143153	12.74702230463521
H	12.40198877854225	7.60997914746417	13.74879488691535
C	14.67474319259822	2.68591973357994	9.92569438498175
H	14.62846944696648	1.62636527608801	9.65423150475992
H	15.64599563778314	3.06472243303294	9.59088095311632
C	17.16705175046274	4.04492893347626	17.30999700080591
H	16.81081757677464	3.09082829627872	17.71013179857681
C	17.28273189326944	5.45727470012581	15.22725499304526
C	13.55117839289282	9.78945407265745	9.78889322898742
H	14.21113788412094	10.63533311773695	9.63161549052001
C	15.62576080607967	9.57519816683649	15.20383927945755
C	19.05902209373631	4.15327726768190	10.55158181858531
H	19.59539142295447	3.76953064038158	9.69315950707376
C	17.90624632570273	5.95359175262617	11.66296045277943
C	12.53417206936711	8.90924753719196	16.17393605937423
H	12.60516184702158	7.82718654069048	16.29215877122147
H	13.06663047421613	9.36371828412644	17.01048024184659

C	16.50968529011116	5.19455931799680	18.07102356975928
H	16.74207379646492	5.12249625460617	19.13848098442631
H	15.42222112475059	5.13755588891591	17.96703876569444
C	17.64309507209305	5.14537937665918	12.79862884216155
C	17.01796244259473	6.52175996098202	17.51357586232841
H	16.54274885235791	7.35753132767720	18.03404871453384
C	12.25572303970157	9.81020190717247	9.29563056423810
H	11.88832857263715	10.67080246960157	8.75134293037046
C	18.58319351233927	5.45522463939762	10.56021173159888
H	18.74509631509700	6.10425883900050	9.70683236962952
C	18.68631885723320	4.12996687254035	17.45412236176465
H	18.97254316446142	4.04617527490611	18.50762746968220
H	19.15986134745155	3.29684602868817	16.92409515068030
C	18.53413102366672	6.60933912048123	17.66083045757355
H	18.88920678090773	7.57311656798650	17.28134636311715
H	18.81516259136441	6.55325597108266	18.71748017496924
C	11.43883893832349	8.71108879086656	9.52107711470270
H	10.41744115464982	8.71357312216507	9.15788244794552
C	17.90015656378214	10.94754134569552	16.18610963864791
H	18.77004566560234	11.46768656070575	16.56654618200630
C	18.12857534038823	3.83118972199063	12.75579691094124
H	17.91895055071271	3.16411552329507	13.57717451204243
C	16.79845673052692	4.12915450801880	15.82978976712506
H	15.71869347599467	4.05116846262952	15.69619513763666
H	17.25079471047530	3.28731907280720	15.30405240922113
C	19.17907328192700	5.46094443293392	16.88520039199262
H	20.26792983482251	5.51968704822477	16.97775401669666
C	18.82578355324761	3.35104444181633	11.65972268550599
H	19.17483566279864	2.32479432878134	11.66687010348356
C	16.80020967659544	10.71554952471068	17.00125861990435
H	16.81280229000528	11.04746078155853	18.03309717331456
C	16.66926907161925	6.60632004542404	16.03298760202055
H	17.04056721243811	7.55333516403734	15.63351496509190
H	15.57418503241374	6.59451659399839	15.95367910125719

11. References

1. Trawny, D. *et al.* Syntheses, Structures and Conformational Dynamics of 1,3,5-Tris(3''-ethynylbi-phen-yl-2'-yl)benzene Derivatives. *Eur. J. Org. Chem.* **2015**, 4667-4674 (2015).
2. Bailey, P. J. *et al.* The First Structural Characterisation of a Group 2 Metal Alkylperoxide Complex: Comments on the Cleavage of Dioxygen by Magnesium Alkyl Complexes. *Chem. Eur. J.* **9**, 4820-4828 (2003).
3. Clark, D. L., Sattelberger, A. P., Bott, S. G. & Vrtis, R. N. Lewis base adducts of uranium triiodide: a new class of synthetically useful precursors for trivalent uranium chemistry. *Inorg. Chem.* **28**, 1771-1773 (1989).
4. Avens, L. R. *et al.* A Convenient Entry into Trivalent Actinide Chemistry: Synthesis and Characterization of $AnI_3(THF)_4$ and $An[N(SiMe_3)_2]_3$ (An = U, Np, Pu). *Inorg. Chem.* **33**, 2248-2256 (1994).
5. Monreal, M. J. *et al.* UI_4(1,4-dioxane)$_2$, [UCl_4(1,4-dioxane)]$_2$, and UI_3(1,4-dioxane)$_{1.5}$: Stable and Versatile Starting Materials for Low- and High-Valent Uranium Chemistry. *Organometallics* **30**, 2031-2038 (2011).
6. Bergbreiter, D. E. & Killough, J. M. Reactions of potassium-graphite. *J. Am. Chem. Soc.* **100**, 2126-2134 (1978).
7. Stoll, S. & Schweiger, A. EasySpin, a comprehensive software package for spectral simulation and analysis in EPR. *J. Magn. Reson.* **178**, 42-55 (2006).
8. Seaman, L. A. *et al.* A Rare Uranyl(VI)–Alkyl Ate Complex $[Li(DME)_{1.5}]_2[UO_2(CH_2SiMe_3)_4]$ and Its Comparison with a Homoleptic Uranium(VI)–Hexaalkyl. *Angew. Chem. Int. Ed.* **52**, 3259-3263 (2013).
9. Greif, A. H., Hrobárik, P., Autschbach, J. & Kaupp, M. Giant spin–orbit effects on ^{1}H and ^{13}C NMR shifts for uranium(vi) complexes revisited: role of the exchange–correlation response kernel, bonding analyses, and new predictions. *Phys. Chem. Chem. Phys.* **18**, 30462-30474 (2016).
10. Vícha, J. *et al.* Relativistic Heavy-Neighbor-Atom Effects on NMR Shifts: Concepts and Trends Across the Periodic Table. *Chem. Rev.* **120**, 7065-7103 (2020).
11. Ford, C. L., Park, Y. J., Matson, E. M., Gordon, Z. & Fout, A. R. A bioinspired iron catalyst for nitrate and perchlorate reduction. *Science* **354**, 741-743 (2016).
12. Tao, W., Yerbulekova, A., Moore, C. E., Shafaat, H. S. & Zhang, S. Controlling the Direction of S-Nitrosation versus Denitrosation: Reversible Cleavage and Formation of an S–N Bond within a Dicopper Center. *J. Am. Chem. Soc.* **144**, 2867-2872 (2022).
13. Kwon, Y. M., Delgado, M., Zakharov, L. N., Seda, T. & Gilbertson, J. D. Nitrite reduction by a pyridinediimine complex with a proton-responsive secondary coordination sphere. *Chem. Commun.* **52**, 11016-11019 (2016).
14. Chiang, C.-K. *et al.* Photoinduced NO and HNO Production from Mononuclear $\{FeNO\}^6$ Complex Bearing a Pendant Thiol. *J. Am. Chem. Soc.* **142**, 8649-8661 (2020).
15. Lewis, A. J., Carroll, P. J. & Schelter, E. J. Reductive Cleavage of Nitrite to Form Terminal Uranium Mono-Oxo Complexes. *J. Am. Chem. Soc.* **135**, 511-518 (2013).

16. Sheldrick, G. SHELXT – Integrated space-group and crystal-structure determination. *Acta Cryst. A* **71**, 3-8 (2015).
17. Sheldrick, G. A short history of SHELX. *Acta Cryst. A* **64**, 112-122 (2008).
18. Sheldrick, G. Crystal structure refinement with SHELXL. *Acta Cryst. C* **71**, 3-8 (2015).
19. Dolomanov, O. V., Bourhis, L. J., Gildea, R. J., Howard, J. A. K. & Puschmann, H. OLEX2: a complete structure solution, refinement and analysis program. *J. Appl. Cryst.* **42**, 339-341 (2009).
20. Spek, A. Single-crystal structure validation with the program PLATON. *J. Appl. Cryst.* **36**, 7-13 (2003).
21. Spek, A. PLATON SQUEEZE: a tool for the calculation of the disordered solvent contribution to the calculated structure factors. *Acta Cryst. C* **71**, 9-18 (2015).
22. Kosugi, K., Akatsuka, C., Iwami, H., Kondo, M. & Masaoka, S. Iron-Complex-Based Supramolecular Framework Catalyst for Visible-Light-Driven CO_2 Reduction. *J. Am. Chem. Soc.* **145**, 10451-10457 (2023).
23. Ichimura, A. S., Wagner, M. J. & Dye, J. L. Anisotropic Charge Transport and Spin−Spin Interactions in K^+(Cryptand [2.2.2]) Electride. *J. Phys. Chem. B* **106**, 11196-11202 (2002).
24. La Pierre, H. S., Scheurer, A., Heinemann, F. W., Hieringer, W. & Meyer, K. Synthesis and Characterization of a Uranium(II) Monoarene Complex Supported by δ Backbonding. *Angew. Chem. Int. Ed.* **53**, 7158-7162 (2014).
25. Neese, F. Software update: the ORCA program system, version 4.0. *WIREs Comput. Mol. Sci.* **8**, e1327 (2018).
26. Neese, F. The ORCA program system. *WIREs Comput. Mol. Sci.* **2**, 73-78 (2012).
27. Weigend, F. & Ahlrichs, R. Balanced basis sets of split valence, triple zeta valence and quadruple zeta valence quality for H to Rn: Design and assessment of accuracy. *Phys. Chem. Chem. Phys.* **7**, 3297-3305 (2005).
28. Lenthe, E. v., Baerends, E. J. & Snijders, J. G. Relativistic regular two-component Hamiltonians. *J. Chem. Phys.* **99**, 4597-4610 (1993).
29. Pantazis, D. A. & Neese, F. All-Electron Scalar Relativistic Basis Sets for the Actinides. *J. Chem. Theory Comput.* **7**, 677-684 (2011).
30. Rolfes, J. D., Neese, F. & Pantazis, D. A. All-electron scalar relativistic basis sets for the elements Rb–Xe. *J. Comput. Chem.* **41**, 1842-1849 (2020).
31. Becke, A. D. Density-functional thermochemistry. III. The role of exact exchange. *J. Chem. Phys.* **98**, 5648-5652 (1993).
32. Grimme, S., Antony, J., Ehrlich, S. & Krieg, H. A consistent and accurate ab initio parametrization of density functional dispersion correction (DFT-D) for the 94 elements H-Pu. *J. Chem. Phys.* **132**, 154104 (2010).
33. Grimme, S., Ehrlich, S. & Goerigk, L. Effect of the damping function in dispersion corrected density functional theory. *J. Comput. Chem.* **32**, 1456-1465 (2011).
34. Glendening, E. D., Landis, C. R. & Weinhold, F. NBO 6.0: Natural bond orbital analysis program. *J. Comput. Chem.* **34**, 1429-1437 (2013).
35. Humphrey, W., Dalke, A. & Schulten, K. VMD: Visual molecular dynamics. *J. Mol. Graph.* **14**, 33-38 (1996).

36. Lu, T. & Chen, F. Multiwfn: A multifunctional wavefunction analyzer. *J. Comput. Chem.* **33**, 580-592 (2012).
37. Mitoraj, M. P., Michalak, A. & Ziegler, T. A Combined Charge and Energy Decomposition Scheme for Bond Analysis. *J. Chem. Theory Comput.* **5**, 962-975 (2009).
