# Supplementary material for: Accessing five oxidation states of uranium in a retained ligand framework
Source: Nat Commun. 2023 Aug 3;14:4657. doi: 10.1038/s41467-023-40403-w (PMC10400547; doi:10.1038/s41467-023-40403-w)

checkCIF/PLATON report

Structure factors have been supplied for datablock(s) H3Ad

THIS REPORT IS FOR GUIDANCE ONLY. IF USED AS PART OF A REVIEW PROCEDURE FOR PUBLICATION, IT SHOULD NOT REPLACE THE EXPERTISE OF AN EXPERIENCED CRYSTALLOGRAPHIC REFEREE.

No syntax errors found. CIF dictionary Interpreting this report

Datablock: H3Ad

Bond precision: C-C = 0.0020 A Wavelength=0.71073

Cell:	a=13.0156(4)	b=13.4914(3)	c=14.0146(3)
	alpha=91.216(2)	beta=106.490(2)	gamma=116.398(3)
Temperature:	180 K		

	Calculated	Reported
Volume	2082.30(11)	2082.30(10)
Space group	P -1	P -1
Hall group	-P 1	-P 1
Moiety formula	C54 H63 N3	C54 H63 N3
Sum formula	C54 H63 N3	C54 H63 N3
Mr	754.07	754.07
Dx,g cm-3	1.203	1.203
Z	2	2
Mu (mm-1)	0.069	0.069
F000	816.0	816.0
F000’	816.27	
h,k,lmax	17,18,19	17,18,19
Nref	11469	10198
Tmin,Tmax	0.984,0.988	0.580,1.000
Tmin’	0.983	

Correction method= # Reported T Limits: Tmin=0.580 Tmax=1.000
AbsCorr = MULTI-SCAN

Data completeness= 0.889 Theta(max)= 29.372

R(reflections)= 0.0454(7937) wR2(reflections)= 0.1202(10198)

S = 1.038 Npar= 526

The following ALERTS were generated. Each ALERT has the format
test-name_ALERT_alert-type_alert-level.
Click on the hyperlinks for more details of the test.

Alert level C

```
PLAT420_ALERT_2_C D-H Bond Without Acceptor  N1       --H1       .     Please Check
PLAT420_ALERT_2_C D-H Bond Without Acceptor  N2       --H2       .     Please Check
PLAT420_ALERT_2_C D-H Bond Without Acceptor  N3       --H3       .     Please Check
PLAT906_ALERT_3_C Large K Value in the Analysis of Variance ......      3.246 Check
PLAT911_ALERT_3_C Missing FCF Refl Between Thmin & STh/L=    0.600         38 Report
```

Alert level G

```
PLAT883_ALERT_1_G No Info/Value for _atom_sites_solution_primary .     Please Do !
PLAT910_ALERT_3_G Missing # of FCF Reflection(s) Below Theta(Min).          4 Note
PLAT912_ALERT_4_G Missing # of FCF Reflections Above STh/L=  0.600       1230 Note
PLAT933_ALERT_2_G Number of HKL-OMIT Records in Embedded .res File          2 Note
PLAT941_ALERT_3_G Average HKL Measurement Multiplicity ..........         2.3 Low
PLAT978_ALERT_2_G Number C-C Bonds with Positive Residual Density.         24 Info
PLAT992_ALERT_5_G Repd & Actual _reflns_number_gt Values Differ by          3 Check
```

0 **ALERT level A** = Most likely a serious problem - resolve or explain
0 **ALERT level B** = A potentially serious problem, consider carefully
5 **ALERT level C** = Check. Ensure it is not caused by an omission or oversight
7 **ALERT level G** = General information/check it is not something unexpected

1 ALERT type 1 CIF construction/syntax error, inconsistent or missing data
5 ALERT type 2 Indicator that the structure model may be wrong or deficient
4 ALERT type 3 Indicator that the structure quality may be low
1 ALERT type 4 Improvement, methodology, query or suggestion
1 ALERT type 5 Informative message, check

Publication of your CIF

You should attempt to resolve as many as possible of the alerts in all categories. Often the minor alerts point to easily fixed oversights, errors and omissions in your CIF or refinement strategy, so attention to these fine details can be worthwhile. In order to resolve some of the more serious problems it may be necessary to carry out additional measurements or structure refinements. However, the nature of your study may justify the reported deviations from journal submission requirements and the more serious of these should be commented upon in the discussion or experimental section of a paper or in the "special_details" fields of the CIF. *checkCIF* was carefully designed to identify outliers and unusual parameters, but every test has its limitations and alerts that are not important in a particular case may appear. Conversely, the absence of alerts does not guarantee there are no aspects of the results needing attention. It is up to the individual to critically assess their own results and, if necessary, seek expert advice.

If you wish to submit your CIF for publication in Acta Crystallographica Section C or E, you should upload your CIF via the web. If you wish to submit your CIF for publication in IUCrData you should upload your CIF via the web. If your CIF is to form part of a submission to another IUCr journal, you will be asked, either during electronic submission or by the Co-editor handling your paper, to upload your CIF via our web site.

PLATON version of 12/09/2022; check.def file version of 09/08/2022

Datablock H3Ad - ellipsoid plot

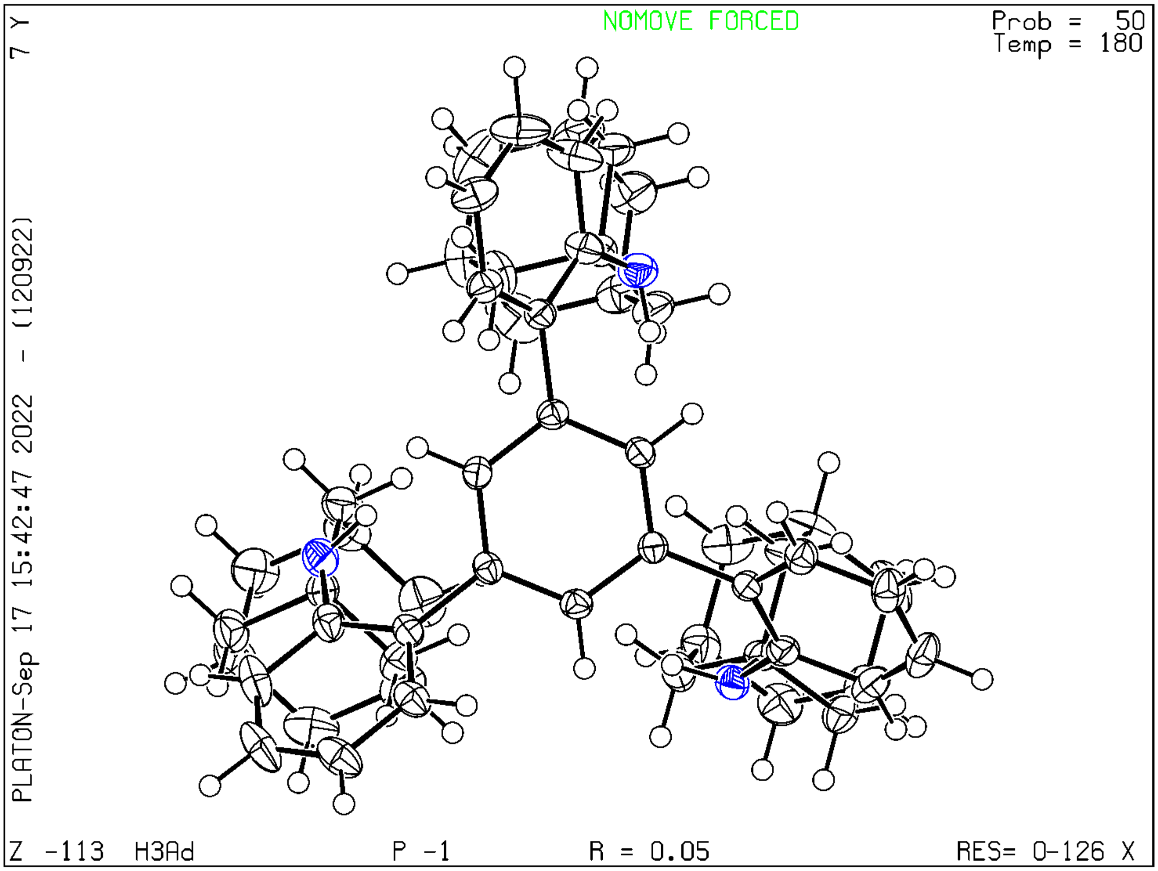

checkCIF/PLATON report

Structure factors have been supplied for datablock(s) AdU_sq

THIS REPORT IS FOR GUIDANCE ONLY. IF USED AS PART OF A REVIEW PROCEDURE FOR PUBLICATION, IT SHOULD NOT REPLACE THE EXPERTISE OF AN EXPERIENCED CRYSTALLOGRAPHIC REFEREE.

No syntax errors found. CIF dictionary Interpreting this report

Datablock: AdU_sq

```
Bond precision:   C-C = 0.0047 A          Wavelength=0.71073

Cell:           a=11.6503(2)       b=14.5422(2)       c=15.3316(3)
                alpha=94.225(1)    beta=105.144(2)    gamma=90.410(1)
Temperature:    180 K

                 Calculated                    Reported
Volume           2499.54(8)                    2499.53(8)
Space group      P -1                          P -1
Hall group       -P 1                          -P 1
Moiety formula   C54 H60 N3 U [+ solvent]      C54 H60 N3 U, 1.8[C4H10O]
Sum formula      C54 H60 N3 U [+ solvent]      C54 H60 N3 U
Mr               989.08                        989.08
Dx,g cm-3        1.314                         1.314
Z                2                             2
Mu (mm-1)        3.282                         3.282
F000             994.0                         994.0
F000'            975.05
h,k,lmax         16,20,21                      16,20,21
Nref             14854                         13156
Tmin,Tmax        0.226,0.374                   0.441,1.000
Tmin'            0.128

Correction method= # Reported T Limits: Tmin=0.441 Tmax=1.000
AbsCorr = MULTI-SCAN

Data completeness= 0.886           Theta(max)= 30.198

                                                        wR2(reflections)=
R(reflections)= 0.0316( 11339)                          0.0754( 13156)

S = 1.035                    Npar= 531
```

The following ALERTS were generated. Each ALERT has the format
test-name_ALERT_alert-type_alert-level.
Click on the hyperlinks for more details of the test.

Alert level C

```
PLAT094_ALERT_2_C Ratio of Maximum / Minimum Residual Density ....       2.05 Report
PLAT410_ALERT_2_C Short Intra H...H Contact  H9       ..H34A     .       1.94 Ang.
                                                      x,y,z  =      1_555 Check
PLAT410_ALERT_2_C Short Intra H...H Contact  H15      ..H54A     .       1.95 Ang.
                                                      x,y,z  =      1_555 Check
PLAT410_ALERT_2_C Short Intra H...H Contact  H21      ..H44A     .       1.95 Ang.
                                                      x,y,z  =      1_555 Check
PLAT910_ALERT_3_C Missing # of FCF Reflection(s) Below Theta(Min).          6 Note
PLAT911_ALERT_3_C Missing FCF Refl Between Thmin & STh/L=    0.600         11 Report
PLAT971_ALERT_2_C Check Calcd Resid. Dens.  0.87Ang From U1              2.43 eA-3
PLAT971_ALERT_2_C Check Calcd Resid. Dens.  0.77Ang From U1              2.21 eA-3
PLAT973_ALERT_2_C Check Calcd Positive Resid. Density on        U1       1.13 eA-3
```

Alert level G

```
FORMU01_ALERT_1_G  There is a discrepancy between the atom counts in the
            _chemical_formula_sum and _chemical_formula_moiety. This is
            usually due to the moiety formula being in the wrong format.
            Atom count from _chemical_formula_sum:   C54 H60 N3 U1
            Atom count from _chemical_formula_moiety:C61.2 H78 N3 O1.8 U1
PLAT164_ALERT_4_G Nr. of Refined C-H H-Atoms in Heavy-Atom Struct.          2 Note
PLAT232_ALERT_2_G Hirshfeld Test Diff (M-X)  U1       --C5       .        5.1 s.u.
PLAT606_ALERT_4_G Solvent Accessible VOID(S) in Structure ........          ! Info
PLAT883_ALERT_1_G No Info/Value for _atom_sites_solution_primary .     Please Do !
PLAT912_ALERT_4_G Missing # of FCF Reflections Above STh/L=  0.600       1679 Note
PLAT913_ALERT_3_G Missing # of Very Strong Reflections in FCF ....          1 Note
PLAT933_ALERT_2_G Number of HKL-OMIT Records in Embedded .res File         10 Note
PLAT941_ALERT_3_G Average HKL Measurement Multiplicity ..........         3.9 Low
PLAT978_ALERT_2_G Number C-C Bonds with Positive Residual Density.          0 Info
```

0 **ALERT level A** = Most likely a serious problem - resolve or explain
0 **ALERT level B** = A potentially serious problem, consider carefully
9 **ALERT level C** = Check. Ensure it is not caused by an omission or oversight
10 **ALERT level G** = General information/check it is not something unexpected

2 ALERT type 1 CIF construction/syntax error, inconsistent or missing data
10 ALERT type 2 Indicator that the structure model may be wrong or deficient
4 ALERT type 3 Indicator that the structure quality may be low
3 ALERT type 4 Improvement, methodology, query or suggestion
0 ALERT type 5 Informative message, check

Publication of your CIF

You should attempt to resolve as many as possible of the alerts in all categories. Often the minor alerts point to easily fixed oversights, errors and omissions in your CIF or refinement strategy, so attention to these fine details can be worthwhile. In order to resolve some of the more serious problems it may be necessary to carry out additional measurements or structure refinements. However, the nature of your study may justify the reported deviations from journal submission requirements and the more serious of these should be commented upon in the discussion or experimental section of a paper or in the "special_details" fields of the CIF. *checkCIF* was carefully designed to identify outliers and unusual parameters, but every test has its limitations and alerts that are not important in a particular case may appear. Conversely, the absence of alerts does not guarantee there are no aspects of the results needing attention. It is up to the individual to critically assess their own results and, if necessary, seek expert advice.

If you wish to submit your CIF for publication in Acta Crystallographica Section C or E, you should upload your CIF via the web. If you wish to submit your CIF for publication in IUCrData you should upload your CIF via the web. If your CIF is to form part of a submission to another IUCr journal, you will be asked, either during electronic submission or by the Co-editor handling your paper, to upload your CIF via our web site.

PLATON version of 10/05/2023; check.def file version of 10/05/2023

Datablock AdU_sq - ellipsoid plot

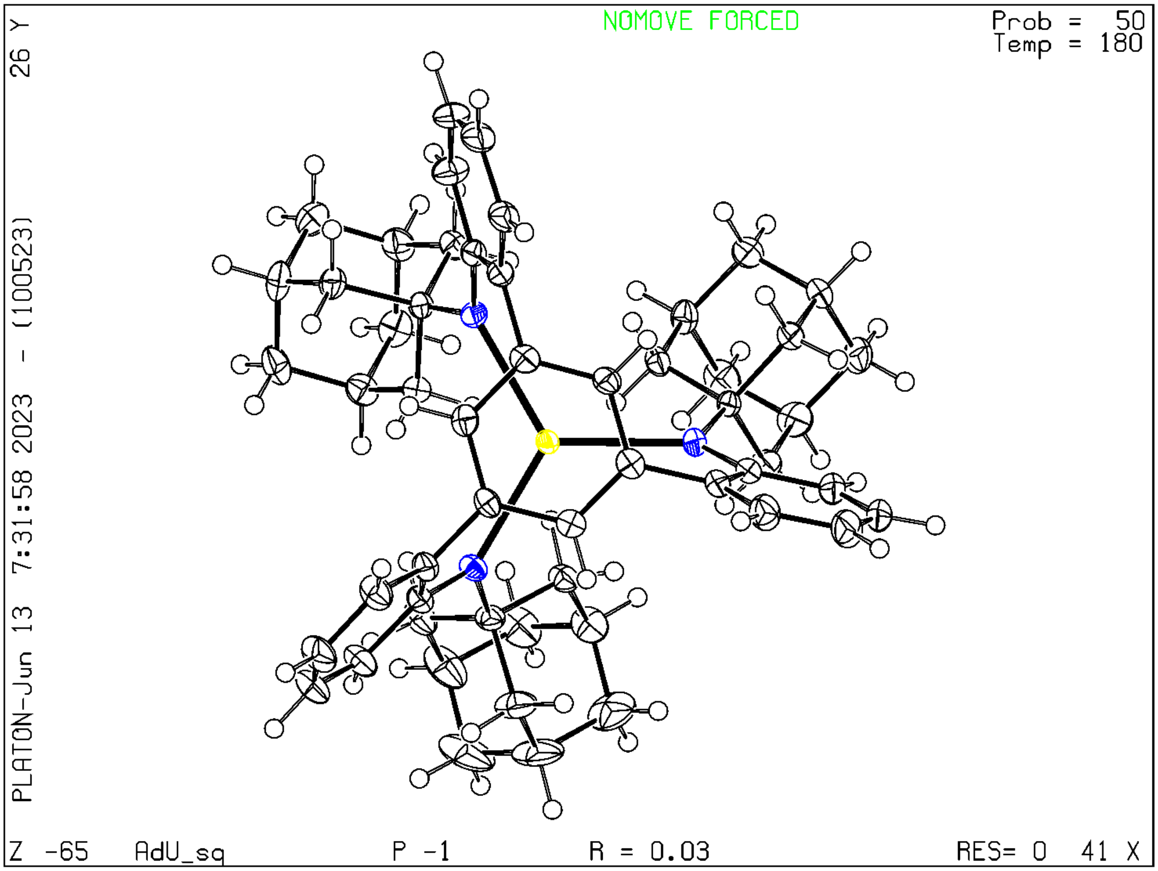

checkCIF/PLATON report

Structure factors have been supplied for datablock(s) Kcrypt_AdU_THF_sq

THIS REPORT IS FOR GUIDANCE ONLY. IF USED AS PART OF A REVIEW PROCEDURE FOR PUBLICATION, IT SHOULD NOT REPLACE THE EXPERTISE OF AN EXPERIENCED CRYSTALLOGRAPHIC REFEREE.

No syntax errors found. CIF dictionary Interpreting this report

Datablock: Kcrypt_AdU_THF_sq

```
Bond precision:   C-C = 0.0060 A          Wavelength=0.71073

Cell:           a=12.9095(2)       b=23.2609(3)      c=25.3412(4)
                alpha=105.030(1)   beta=90.328(1)    gamma=100.678(1)
Temperature:    180 K
```

	Calculated	Reported
Volume	7210.44(19)	7210.43(19)
Space group	P -1	P -1
Hall group	-P 1	-P 1
Moiety formula	2(C54 H60 N3 U), 2(C18 H36 K N2 O6), C4 H8 O [+ solvent]	2(C54 H60 N3 U), 2(C18 H36 K N2 O6), C4 H8 O, 2.43[C4H8O]
Sum formula	C148 H200 K2 N10 O13 U2 [+ solvent]	C148 H200 K2 N10 O13 U2
Mr	2881.44	2881.43
Dx,g cm-3	1.327	1.327
Z	2	2
Mu (mm-1)	2.362	2.362
F000	2968.0	2968.0
F000’	2931.24	
h,k,lmax	17,32,35	17,31,34
Nref	40471	36454
Tmin,Tmax	0.359,0.389	0.258,1.000
Tmin’	0.332	

```
Correction method= # Reported T Limits: Tmin=0.258 Tmax=1.000
AbsCorr = MULTI-SCAN

Data completeness= 0.901           Theta(max)= 29.579
```

R(reflections)= 0.0398(27338)   wR2(reflections)= 0.1045(36454)

S = 1.026   Npar= 1576

The following ALERTS were generated. Each ALERT has the format
test-name_ALERT_alert-type_alert-level.
Click on the hyperlinks for more details of the test.

Alert level B

```
PLAT910_ALERT_3_B Missing # of FCF Reflection(s) Below Theta(Min).         16 Note
```

Author Response: Several low-order reflections might have been 'Omitted' from the (final) least-squares refinement

---

Alert level C

```
ABSTY02_ALERT_1_C  An _exptl_absorpt_correction_type has been given without
            a literature citation. This should be contained in the
            _exptl_absorpt_process_details field.
            Absorption correction given as multi-scan
PLAT220_ALERT_2_C NonSolvent   Resd 1  C   Ueq(max)/Ueq(min) Range        3.6 Ratio
PLAT230_ALERT_2_C Hirshfeld Test Diff for    C13      --C14      .        5.7 s.u.
PLAT260_ALERT_2_C Large Average Ueq of Residue Including       O13      0.106 Check
PLAT410_ALERT_2_C Short Intra H...H Contact  H15      ..H38A     .       1.95 Ang.
                                                      x,y,z  =      1_555 Check
PLAT410_ALERT_2_C Short Intra H...H Contact  H21      ..H46B     .       1.95 Ang.
                                                      x,y,z  =      1_555 Check
PLAT410_ALERT_2_C Short Intra H...H Contact  H11D     ..H93      .       1.93 Ang.
                                                      x,y,z  =      1_555 Check
PLAT410_ALERT_2_C Short Intra H...H Contact  H81      ..H99B     .       1.91 Ang.
                                                      x,y,z  =      1_555 Check
PLAT911_ALERT_3_C Missing FCF Refl Between Thmin & STh/L=    0.600         43 Report
PLAT971_ALERT_2_C Check Calcd Resid. Dens.  1.49Ang From N7              1.68 eA-3
PLAT971_ALERT_2_C Check Calcd Resid. Dens.  1.00Ang From U2              1.64 eA-3
PLAT971_ALERT_2_C Check Calcd Resid. Dens.  1.00Ang From U1              1.58 eA-3
PLAT971_ALERT_2_C Check Calcd Resid. Dens.  1.12Ang From C78             1.57 eA-3
PLAT971_ALERT_2_C Check Calcd Resid. Dens.  1.02Ang From U2              1.54 eA-3
PLAT971_ALERT_2_C Check Calcd Resid. Dens.  0.89Ang From U2              1.53 eA-3
PLAT972_ALERT_2_C Check Calcd Resid. Dens.  1.17Ang From N7             -1.64 eA-3
PLAT972_ALERT_2_C Check Calcd Resid. Dens.  1.62Ang From U2             -1.57 eA-3
PLAT972_ALERT_2_C Check Calcd Resid. Dens.  1.50Ang From C27            -1.51 eA-3
```

Alert level G

```
FORMU01_ALERT_1_G  There is a discrepancy between the atom counts in the
            _chemical_formula_sum and _chemical_formula_moiety. This is
            usually due to the moiety formula being in the wrong format.
            Atom count from _chemical_formula_sum:   C148 H200 K2 N10 O13 U2
            Atom count from _chemical_formula_moiety:C157.7199 H219.44 K2 N10 O15.
PLAT154_ALERT_1_G The s.u.'s on the Cell Angles are Equal ..(Note)      0.001 Degree
PLAT398_ALERT_2_G Deviating  C-O-C   Angle From 120 for O13      .      108.5 Degree
```

```
PLAT606_ALERT_4_G Solvent Accessible VOID(S) in Structure ........          ! Info
PLAT790_ALERT_4_G Centre of Gravity not Within Unit Cell: Resd.  #          5 Note
             C4 H8 O
PLAT883_ALERT_1_G No Info/Value for _atom_sites_solution_primary .     Please Do !
PLAT912_ALERT_4_G Missing # of FCF Reflections Above STh/L=  0.600       3909 Note
PLAT913_ALERT_3_G Missing # of Very Strong Reflections in FCF ....          1 Note
PLAT933_ALERT_2_G Number of HKL-OMIT Records in Embedded .res File         43 Note
PLAT941_ALERT_3_G Average HKL Measurement Multiplicity ..........         2.9 Low
PLAT978_ALERT_2_G Number C-C Bonds with Positive Residual Density.          0 Info
```

```
   0 ALERT level A = Most likely a serious problem - resolve or explain
   1 ALERT level B = A potentially serious problem, consider carefully
  18 ALERT level C = Check. Ensure it is not caused by an omission or oversight
  11 ALERT level G = General information/check it is not something unexpected

   4 ALERT type 1 CIF construction/syntax error, inconsistent or missing data
  19 ALERT type 2 Indicator that the structure model may be wrong or deficient
   4 ALERT type 3 Indicator that the structure quality may be low
   3 ALERT type 4 Improvement, methodology, query or suggestion
   0 ALERT type 5 Informative message, check
```

Publication of your CIF

You should attempt to resolve as many as possible of the alerts in all categories. Often the minor alerts point to easily fixed oversights, errors and omissions in your CIF or refinement strategy, so attention to these fine details can be worthwhile. In order to resolve some of the more serious problems it may be necessary to carry out additional measurements or structure refinements. However, the nature of your study may justify the reported deviations from journal submission requirements and the more serious of these should be commented upon in the discussion or experimental section of a paper or in the "special_details" fields of the CIF. *checkCIF* was carefully designed to identify outliers and unusual parameters, but every test has its limitations and alerts that are not important in a particular case may appear. Conversely, the absence of alerts does not guarantee there are no aspects of the results needing attention. It is up to the individual to critically assess their own results and, if necessary, seek expert advice.

If you wish to submit your CIF for publication in Acta Crystallographica Section C or E, you should upload your CIF via the web. If you wish to submit your CIF for publication in IUCrData you should upload your CIF via the web. If your CIF is to form part of a submission to another IUCr journal, you will be asked, either during electronic submission or by the Co-editor handling your paper, to upload your CIF via our web site.

PLATON version of 10/05/2023; check.def file version of 10/05/2023

Datablock Kcrypt_AdU_THF_sq - ellipsoid plot

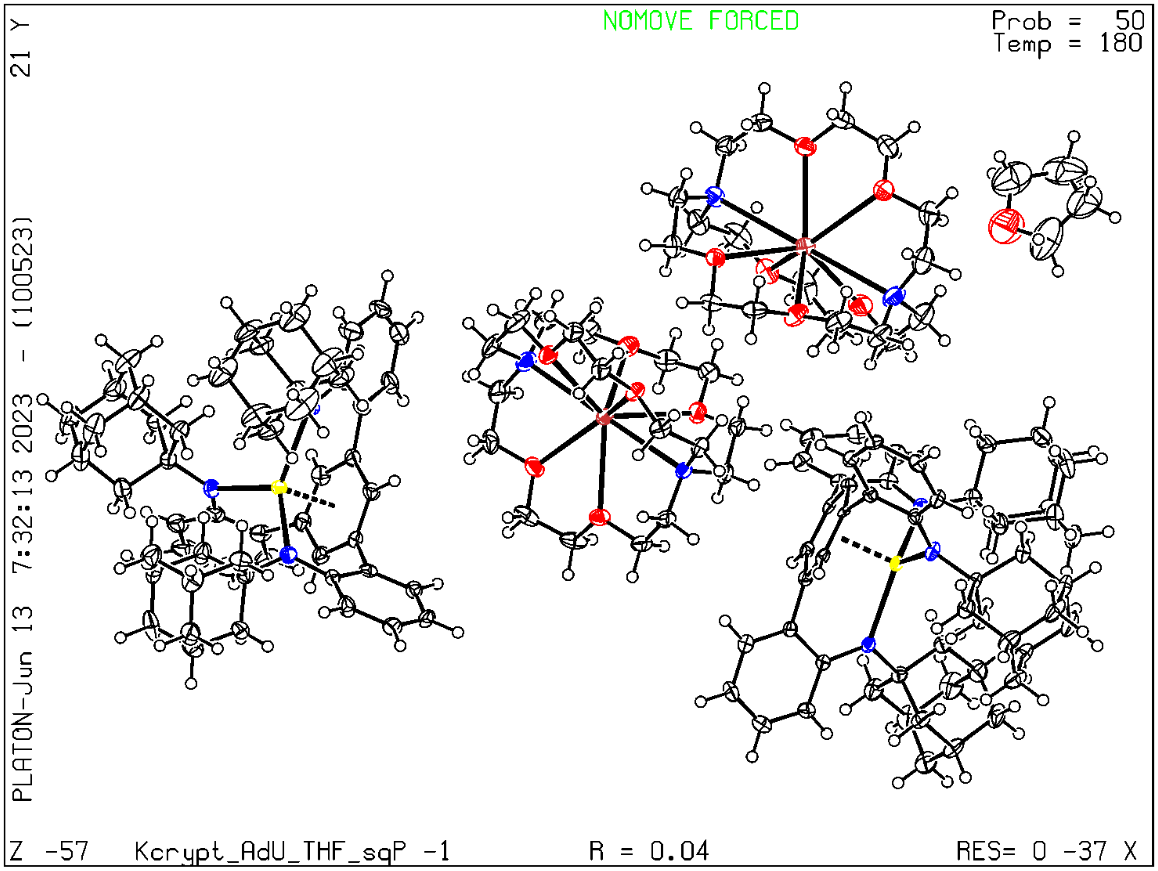

checkCIF/PLATON report

Structure factors have been supplied for datablock(s) AdUO_C7H8_sq

THIS REPORT IS FOR GUIDANCE ONLY. IF USED AS PART OF A REVIEW PROCEDURE FOR PUBLICATION, IT SHOULD NOT REPLACE THE EXPERTISE OF AN EXPERIENCED CRYSTALLOGRAPHIC REFEREE.

No syntax errors found. CIF dictionary Interpreting this report

Datablock: AdUO_C7H8_sq

```
Bond precision:   C-C = 0.0053 A          Wavelength=0.71073

Cell:            a=16.6702(3)     b=13.7504(3)        c=23.1478(6)
                 alpha=90         beta=107.260(2)     gamma=90
Temperature:     180 K
```

	Calculated	Reported
Volume	5067.1(2)	5067.0(2)
Space group	P 21/n	P 1 21/n 1
Hall group	-P 2yn	-P 2yn
Moiety formula	C54 H60 N3 O U, C7 H8 [+ solvent]	C54 H60 N3 O U, C7 H8, 0.5[C5H12]
Sum formula	C61 H68 N3 O U [+ solvent]	C61 H68 N3 O U
Mr	1097.21	1097.21
Dx,g cm-3	1.438	1.438
Z	4	4
Mu (mm-1)	3.247	3.247
F000	2220.0	2220.0
F000'	2182.17	
h,k,lmax	20,17,28	20,17,28
Nref	10351	10342
Tmin,Tmax	0.246,0.378	0.307,1.000
Tmin'	0.184	

```
Correction method= # Reported T Limits: Tmin=0.307 Tmax=1.000
AbsCorr = MULTI-SCAN

Data completeness= 0.999          Theta(max)= 26.373

R(reflections)= 0.0289( 8483)     wR2(reflections)= 0.0717( 10342)

S = 1.026                Npar= 596
```

The following ALERTS were generated. Each ALERT has the format
test-name_ALERT_alert-type_alert-level.
Click on the hyperlinks for more details of the test.

Alert level C

```
PLAT094_ALERT_2_C Ratio of Maximum / Minimum Residual Density ....       2.72 Report
PLAT244_ALERT_4_C Low    'Solvent' Ueq as Compared to Neighbors of        C55 Check
PLAT911_ALERT_3_C Missing FCF Refl Between Thmin & STh/L=    0.600          2 Report
PLAT971_ALERT_2_C Check Calcd Resid. Dens.  0.92Ang From U1              2.08 eA-3
PLAT971_ALERT_2_C Check Calcd Resid. Dens.  1.08Ang From U1              1.95 eA-3
PLAT975_ALERT_2_C Check Calcd Resid. Dens.  1.08Ang From N2      .       0.45 eA-3
PLAT976_ALERT_2_C Check Calcd Resid. Dens.  0.64Ang From O1      .      -0.50 eA-3
```

Alert level G

```
FORMU01_ALERT_1_G  There is a discrepancy between the atom counts in the
            _chemical_formula_sum and _chemical_formula_moiety. This is
            usually due to the moiety formula being in the wrong format.
            Atom count from _chemical_formula_sum:   C61 H68 N3 O1 U1
            Atom count from _chemical_formula_moiety:C63.5 H74 N3 O1 U1
PLAT605_ALERT_4_G Largest Solvent Accessible VOID in the Structure        206 A**3
PLAT794_ALERT_5_G Tentative Bond Valency for U1        (IV)      .       4.16 Info
PLAT883_ALERT_1_G No Info/Value for _atom_sites_solution_primary .     Please Do !
PLAT910_ALERT_3_G Missing # of FCF Reflection(s) Below Theta(Min).          4 Note
PLAT933_ALERT_2_G Number of HKL-OMIT Records in Embedded .res File          1 Note
PLAT978_ALERT_2_G Number C-C Bonds with Positive Residual Density.          4 Info
```

0 **ALERT level A** = Most likely a serious problem - resolve or explain
0 **ALERT level B** = A potentially serious problem, consider carefully
7 **ALERT level C** = Check. Ensure it is not caused by an omission or oversight
7 **ALERT level G** = General information/check it is not something unexpected

2 ALERT type 1 CIF construction/syntax error, inconsistent or missing data
7 ALERT type 2 Indicator that the structure model may be wrong or deficient
2 ALERT type 3 Indicator that the structure quality may be low
2 ALERT type 4 Improvement, methodology, query or suggestion
1 ALERT type 5 Informative message, check

Publication of your CIF

You should attempt to resolve as many as possible of the alerts in all categories. Often the minor alerts point to easily fixed oversights, errors and omissions in your CIF or refinement strategy, so attention to these fine details can be worthwhile. In order to resolve some of the more serious problems it may be necessary to carry out additional measurements or structure refinements. However, the nature of your study may justify the reported deviations from journal submission requirements and the more serious of these should be commented upon in the discussion or experimental section of a paper or in the "special_details" fields of the CIF. *checkCIF* was carefully designed to identify outliers and unusual parameters, but every test has its limitations and alerts that are not important in a particular case may appear. Conversely, the absence of alerts does not guarantee there are no aspects of the results needing attention. It is up to the individual to critically assess their own results and, if necessary, seek expert advice.

If you wish to submit your CIF for publication in Acta Crystallographica Section C or E, you should upload your CIF via the web. If you wish to submit your CIF for publication in IUCrData you should upload your CIF via the web. If your CIF is to form part of a submission to another IUCr journal, you will be asked, either during electronic submission or by the Co-editor handling your paper, to upload your CIF via our web site.

PLATON version of 10/05/2023; check.def file version of 10/05/2023

Datablock AdUO_C7H8_sq - ellipsoid plot

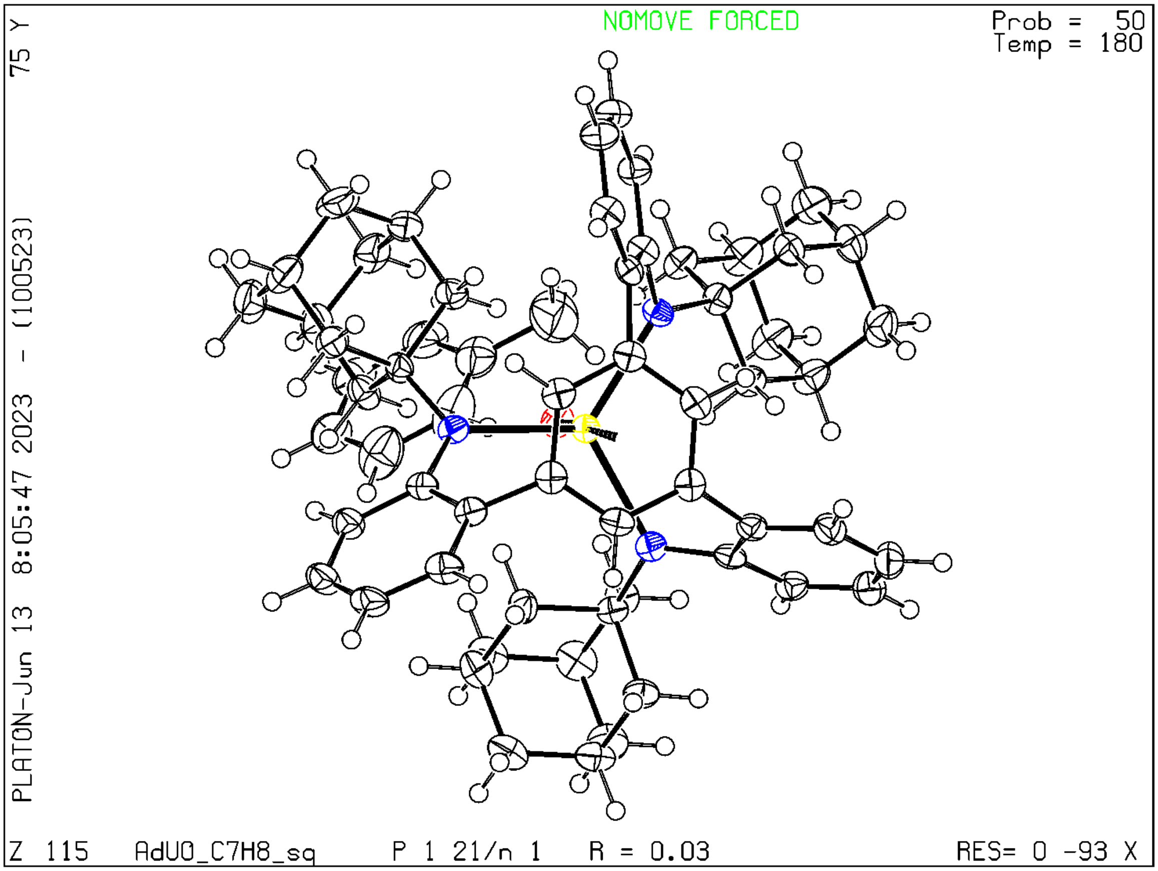

checkCIF/PLATON report

Structure factors have been supplied for datablock(s) AdUO

THIS REPORT IS FOR GUIDANCE ONLY. IF USED AS PART OF A REVIEW PROCEDURE FOR PUBLICATION, IT SHOULD NOT REPLACE THE EXPERTISE OF AN EXPERIENCED CRYSTALLOGRAPHIC REFEREE.

No syntax errors found. CIF dictionary Interpreting this report

Datablock: AdUO

```
Bond precision:   C-C = 0.0042 A          Wavelength=0.71073

Cell:             a=10.9609(2)     b=18.5423(3)       c=21.1803(5)
                  alpha=90         beta=100.713(2)    gamma=90
Temperature:      180 K

                 Calculated                  Reported
Volume           4229.66(15)                 4229.66(15)
Space group      P 21/c                      P 1 21/c 1
Hall group       -P 2ybc                     -P 2ybc
Moiety formula   C54 H60 N3 O U              C54 H60 N3 O U
Sum formula      C54 H60 N3 O U              C54 H60 N3 O U
Mr               1005.08                     1005.08
Dx,g cm-3        1.578                       1.578
Z                4                           4
Mu (mm-1)        3.882                       3.882
F000             2020.0                      2020.0
F000'            1982.13
h,k,lmax         15,25,29                    15,25,27
Nref             11685                       10717
Tmin,Tmax        0.257,0.312                 0.674,1.000
Tmin'            0.204

Correction method= # Reported T Limits: Tmin=0.674 Tmax=1.000
AbsCorr = MULTI-SCAN

Data completeness= 0.917           Theta(max)= 29.405

R(reflections)= 0.0251( 8956)                          wR2(reflections)=
                                                       0.0583( 10717)
S = 1.044                    Npar= 532
```

The following ALERTS were generated. Each ALERT has the format
test-name_ALERT_alert-type_alert-level.
Click on the hyperlinks for more details of the test.

Alert level C

```
ABSTY02_ALERT_1_C  An _exptl_absorpt_correction_type has been given without
            a literature citation. This should be contained in the
            _exptl_absorpt_process_details field.
            Absorption correction given as multi-scan
PLAT094_ALERT_2_C Ratio of Maximum / Minimum Residual Density ....       3.16 Report
PLAT910_ALERT_3_C Missing # of FCF Reflection(s) Below Theta(Min).          5 Note
PLAT911_ALERT_3_C Missing FCF Refl Between Thmin & STh/L=    0.600          2 Report
PLAT971_ALERT_2_C Check Calcd Resid. Dens.  0.86Ang From U1              1.82 eA-3
```

Alert level G

```
PLAT794_ALERT_5_G Tentative Bond Valency for U1        (IV)      .       4.22 Info
PLAT883_ALERT_1_G No Info/Value for _atom_sites_solution_primary .     Please Do !
PLAT912_ALERT_4_G Missing # of FCF Reflections Above STh/L=  0.600        899 Note
PLAT933_ALERT_2_G Number of HKL-OMIT Records in Embedded .res File          3 Note
PLAT941_ALERT_3_G Average HKL Measurement Multiplicity ..........         3.0 Low
PLAT952_ALERT_5_G Calculated (ThMax) and CIF-Reported Lmax Differ.          2 Units
PLAT958_ALERT_1_G Calculated (ThMax) and Actual (FCF) Lmax Differ.          2 Units
PLAT978_ALERT_2_G Number C-C Bonds with Positive Residual Density.         12 Info
```

0 **ALERT level A** = Most likely a serious problem - resolve or explain
0 **ALERT level B** = A potentially serious problem, consider carefully
5 **ALERT level C** = Check. Ensure it is not caused by an omission or oversight
8 **ALERT level G** = General information/check it is not something unexpected

3 ALERT type 1 CIF construction/syntax error, inconsistent or missing data
4 ALERT type 2 Indicator that the structure model may be wrong or deficient
3 ALERT type 3 Indicator that the structure quality may be low
1 ALERT type 4 Improvement, methodology, query or suggestion
2 ALERT type 5 Informative message, check

Publication of your CIF

You should attempt to resolve as many as possible of the alerts in all categories. Often the minor alerts point to easily fixed oversights, errors and omissions in your CIF or refinement strategy, so attention to these fine details can be worthwhile. In order to resolve some of the more serious problems it may be necessary to carry out additional measurements or structure refinements. However, the nature of your study may justify the reported deviations from journal submission requirements and the more serious of these should be commented upon in the discussion or experimental section of a paper or in the "special_details" fields of the CIF. *checkCIF* was carefully designed to identify outliers and unusual parameters, but every test has its limitations and alerts that are not important in a particular case may appear. Conversely, the absence of alerts does not guarantee there are no aspects of the results needing attention. It is up to the individual to critically assess their own results and, if necessary, seek expert advice.

If you wish to submit your CIF for publication in Acta Crystallographica Section C or E, you should upload your CIF via the web. If you wish to submit your CIF for publication in IUCrData you should upload your CIF via the web. If your CIF is to form part of a submission to another IUCr journal, you will be asked, either during electronic submission or by the Co-editor handling your paper, to upload your CIF via our web site.

PLATON version of 12/09/2022; check.def file version of 09/08/2022

Datablock AdUO - ellipsoid plot

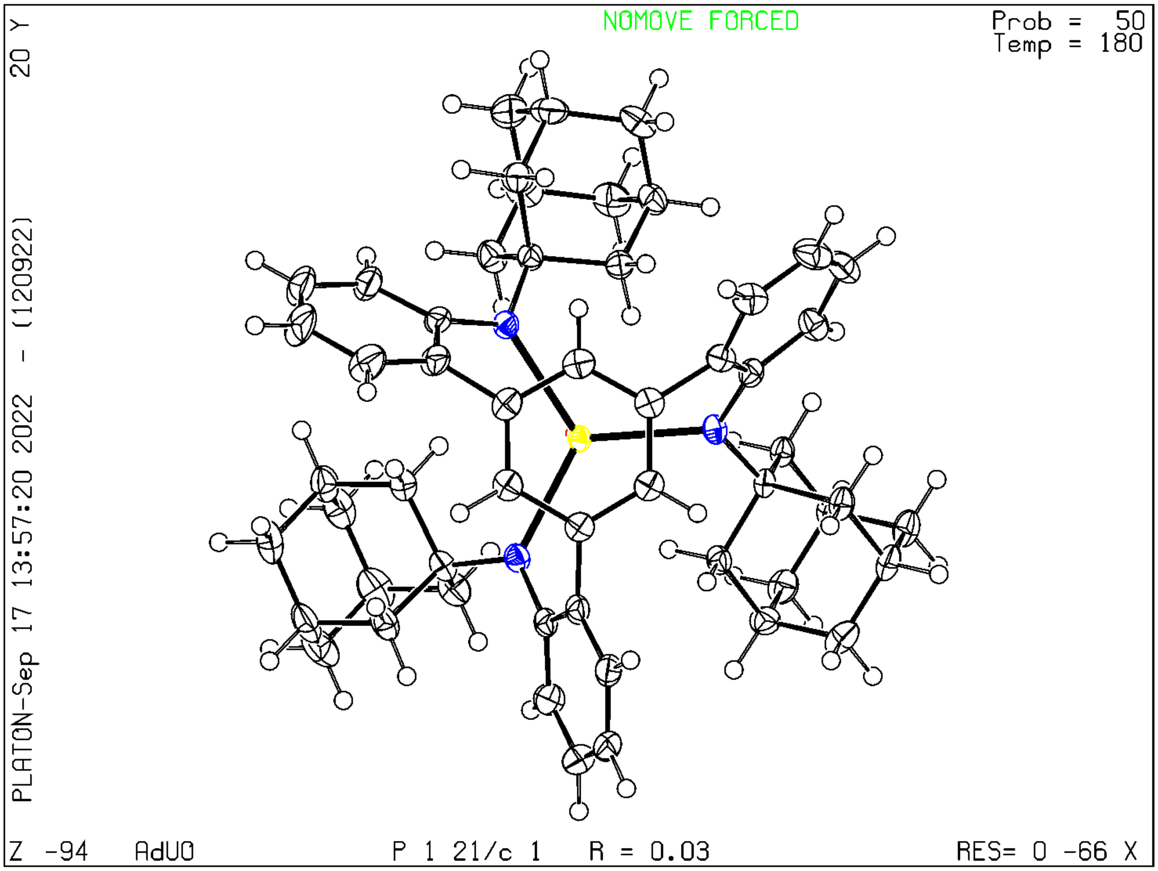

checkCIF/PLATON report

Structure factors have been supplied for datablock(s) Kcrypt_AdUO

THIS REPORT IS FOR GUIDANCE ONLY. IF USED AS PART OF A REVIEW PROCEDURE FOR PUBLICATION, IT SHOULD NOT REPLACE THE EXPERTISE OF AN EXPERIENCED CRYSTALLOGRAPHIC REFEREE.

No syntax errors found. CIF dictionary Interpreting this report

Datablock: Kcrypt_AdUO

Bond precision: C-C = 0.0071 A Wavelength=0.71073

Cell:	a=17.4587(4)	b=17.4587(4)	c=18.5858(6)
	alpha=90	beta=90	gamma=120
Temperature:	180 K		

	Calculated	Reported
Volume	4906.1(3)	4906.1(3)
Space group	R 3	R 3
Hall group	R 3	R 3
Moiety formula	C54 H60 N3 O U, C18 H36 K N2 O6	C54 H60 N3 O1 U1, C18 H36 K1 N2 O6
Sum formula	C72 H96 K N5 O7 U	C72 H96 K N5 O7 U
Mr	1420.67	1420.66
Dx,g cm-3	1.443	1.443
Z	3	3
Mu (mm-1)	2.603	2.603
F000	2190.0	2190.0
F000'	2162.44	
h,k,lmax	24,24,25	22,23,24
Nref	6102[3051]	4511
Tmin,Tmax	0.855,0.925	0.811,1.000
Tmin'	0.522	

Correction method= # Reported T Limits: Tmin=0.811 Tmax=1.000
AbsCorr = MULTI-SCAN

Data completeness= 1.48/0.74 Theta(max)= 29.528

R(reflections)= 0.0205(4511) wR2(reflections)= 0.0448(4511)

S = 1.036 Npar= 259

The following ALERTS were generated. Each ALERT has the format
test-name_ALERT_alert-type_alert-level.
Click on the hyperlinks for more details of the test.

Alert level C

```
CRYSC01_ALERT_1_C The word below has not been recognised as a standard
            identifier.
            greenish
PLAT042_ALERT_1_C Calc. and Reported MoietyFormula Strings  Differ     Please Check
PLAT915_ALERT_3_C No Flack x Check Done: Low Friedel Pair Coverage         58 %
PLAT971_ALERT_2_C Check Calcd Resid. Dens.  0.93Ang From O1              1.90 eA-3
PLAT971_ALERT_2_C Check Calcd Resid. Dens.  0.82Ang From U1              1.83 eA-3
PLAT975_ALERT_2_C Check Calcd Resid. Dens.  0.89Ang From O1      .       0.41 eA-3
```

Alert level G

```
PLAT764_ALERT_4_G Overcomplete CIF Bond List Detected (Rep/Expd) .       1.12 Ratio
PLAT794_ALERT_5_G Tentative Bond Valency for U1        (IV)      .       3.47 Info
PLAT883_ALERT_1_G No Info/Value for _atom_sites_solution_primary .     Please Do !
PLAT910_ALERT_3_G Missing # of FCF Reflection(s) Below Theta(Min).          1 Note
PLAT912_ALERT_4_G Missing # of FCF Reflections Above STh/L=  0.600        304 Note
PLAT933_ALERT_2_G Number of HKL-OMIT Records in Embedded .res File          2 Note
PLAT941_ALERT_3_G Average HKL Measurement Multiplicity ...........        2.3 Low
PLAT950_ALERT_5_G Calculated (ThMax) and CIF-Reported Hmax Differ           2 Units
PLAT961_ALERT_5_G Dataset Contains no Negative Intensities .......     Please Check
PLAT978_ALERT_2_G Number C-C Bonds with Positive Residual Density.          1 Info
```

```
   0 ALERT level A = Most likely a serious problem - resolve or explain
   0 ALERT level B = A potentially serious problem, consider carefully
   6 ALERT level C = Check. Ensure it is not caused by an omission or oversight
  10 ALERT level G = General information/check it is not something unexpected

   3 ALERT type 1 CIF construction/syntax error, inconsistent or missing data
   5 ALERT type 2 Indicator that the structure model may be wrong or deficient
   3 ALERT type 3 Indicator that the structure quality may be low
   2 ALERT type 4 Improvement, methodology, query or suggestion
   3 ALERT type 5 Informative message, check
```

Publication of your CIF

You should attempt to resolve as many as possible of the alerts in all categories. Often the minor alerts point to easily fixed oversights, errors and omissions in your CIF or refinement strategy, so attention to these fine details can be worthwhile. In order to resolve some of the more serious problems it may be necessary to carry out additional measurements or structure refinements. However, the nature of your study may justify the reported deviations from journal submission requirements and the more serious of these should be commented upon in the discussion or experimental section of a paper or in the "special_details" fields of the CIF. *checkCIF* was carefully designed to identify outliers and unusual parameters, but every test has its limitations and alerts that are not important in a particular case may appear. Conversely, the absence of alerts does not guarantee there are no aspects of the results needing attention. It is up to the individual to critically assess their own results and, if necessary, seek expert advice.

If you wish to submit your CIF for publication in Acta Crystallographica Section C or E, you should upload your CIF via the web. If you wish to submit your CIF for publication in IUCrData you should upload your CIF via the web. If your CIF is to form part of a submission to another IUCr journal, you will be asked, either during electronic submission or by the Co-editor handling your paper, to upload your CIF via our web site.

PLATON version of 12/09/2022; check.def file version of 09/08/2022

Datablock Kcrypt_AdUO - ellipsoid plot

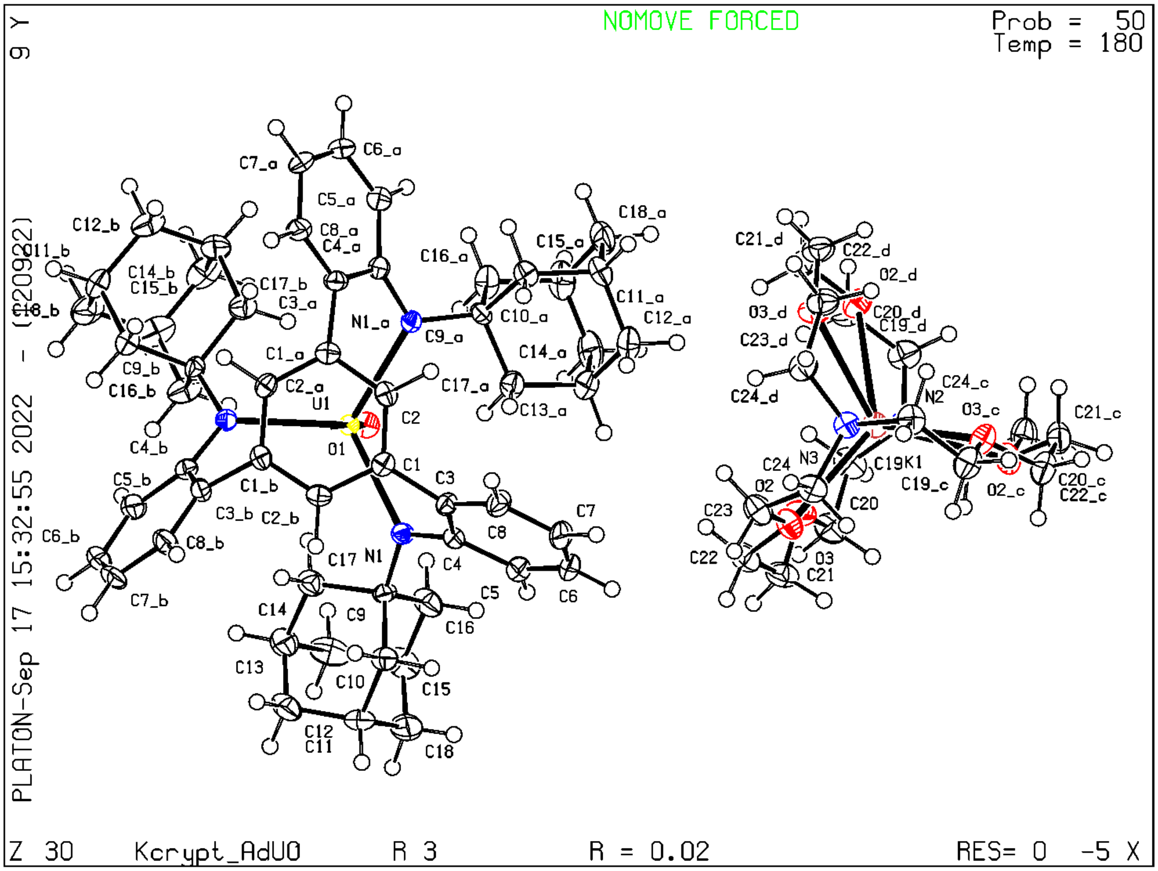

checkCIF/PLATON report

Structure factors have been supplied for datablock(s) Cpstar2Co_AdUO_THF

THIS REPORT IS FOR GUIDANCE ONLY. IF USED AS PART OF A REVIEW PROCEDURE FOR PUBLICATION, IT SHOULD NOT REPLACE THE EXPERTISE OF AN EXPERIENCED CRYSTALLOGRAPHIC REFEREE.

No syntax errors found.   CIF dictionary   Interpreting this report

Datablock: Cpstar2Co_AdUO_THF

Bond precision:	C-C = 0.0183 A		Wavelength=0.71073
Cell:	a=13.3624(5)	b=26.7946(8)	c=18.4675(7)
	alpha=90	beta=90.015(3)	gamma=90
Temperature:	180 K		

	Calculated	Reported
Volume	6612.1(4)	6612.1(4)
Space group	P 21/n	P 1 21/n 1
Hall group	-P 2yn	-P 2yn
Moiety formula	C54 H60 N3 O U, C20 H30 Co, C4 H8 O	C54 H60 N3 O U, C20 H30 Co, C4 H8 O
Sum formula	C78 H98 Co N3 O2 U	C78 H98 Co N3 O2 U
Mr	1406.55	1406.55
Dx,g cm-3	1.413	1.413
Z	4	4
Mu (mm-1)	2.746	2.746
F000	2888.0	2888.0
F000'	2851.70	
h,k,lmax	15,31,21	15,31,21
Nref	11662	11650
Tmin,Tmax	0.848,0.872	0.799,1.000
Tmin'	0.662	

Correction method= # Reported T Limits: Tmin=0.799 Tmax=1.000
AbsCorr = MULTI-SCAN

Data completeness= 0.999   Theta(max)= 25.026

R(reflections)= 0.0765(8587)   wR2(reflections)= 0.1769(11650)

S = 1.104   Npar= 775

The following ALERTS were generated. Each ALERT has the format
test-name_ALERT_alert-type_alert-level.
Click on the hyperlinks for more details of the test.

Alert level A

```
PLAT971_ALERT_2_A Check Calcd Resid. Dens.  0.92Ang From U1            4.03 eA-3
```

Author Response: This error is due to residual density near heavy metal (U) or incomplete absorption correction. The specific structural parameter might not be precise; however, we believe the key finding (atom connection) of the structure is shown unambiguously.

Alert level B

```
PLAT973_ALERT_2_B Check Calcd Positive Resid. Density on        U1       1.82 eA-3
```

Author Response: Near heavy metal or incomplete absorption correction

Alert level C

```
ABSTY02_ALERT_1_C  An _exptl_absorpt_correction_type has been given without
            a literature citation. This should be contained in the
            _exptl_absorpt_process_details field.
            Absorption correction given as multi-scan
RINTA01_ALERT_3_C  The value of Rint is greater than 0.12
            Rint given   0.133
PLAT020_ALERT_3_C The Value of Rint is Greater Than 0.12 .........      0.133 Report
PLAT094_ALERT_2_C Ratio of Maximum / Minimum Residual Density ....       2.79 Report
PLAT214_ALERT_2_C Atom C74       (Anion/Solvent) ADP max/min Ratio        4.6 prolat
PLAT221_ALERT_2_C Solv./Anion  Resd 2  C   Ueq(max)/Ueq(min) Range        5.3 Ratio
PLAT234_ALERT_4_C Large Hirshfeld Difference C5       --C6       .       0.16 Ang.
PLAT234_ALERT_4_C Large Hirshfeld Difference C29      --C30      .       0.16 Ang.
PLAT234_ALERT_4_C Large Hirshfeld Difference C41      --C42      .       0.17 Ang.
PLAT234_ALERT_4_C Large Hirshfeld Difference C46      --C50      .       0.17 Ang.
PLAT234_ALERT_4_C Large Hirshfeld Difference C49      --C53      .       0.24 Ang.
PLAT234_ALERT_4_C Large Hirshfeld Difference C50      --C53      .       0.22 Ang.
PLAT234_ALERT_4_C Large Hirshfeld Difference C58      --C59      .       0.17 Ang.
PLAT234_ALERT_4_C Large Hirshfeld Difference C66      --C67      .       0.18 Ang.
PLAT241_ALERT_2_C High   'MainMol' Ueq as Compared to Neighbors of        C52 Check
PLAT242_ALERT_2_C Low    'MainMol' Ueq as Compared to Neighbors of        C45 Check
PLAT242_ALERT_2_C Low    'MainMol' Ueq as Compared to Neighbors of        C51 Check
PLAT242_ALERT_2_C Low    'MainMol' Ueq as Compared to Neighbors of        Co1 Check
PLAT242_ALERT_2_C Low    'MainMol' Ueq as Compared to Neighbors of        C65 Check
PLAT242_ALERT_2_C Low    'MainMol' Ueq as Compared to Neighbors of        C66 Check
PLAT242_ALERT_2_C Low    'MainMol' Ueq as Compared to Neighbors of        C67 Check
PLAT242_ALERT_2_C Low    'MainMol' Ueq as Compared to Neighbors of        C68 Check
PLAT242_ALERT_2_C Low    'MainMol' Ueq as Compared to Neighbors of        C69 Check
PLAT243_ALERT_4_C High   'Solvent' Ueq as Compared to Neighbors of        C75 Check
PLAT243_ALERT_4_C High   'Solvent' Ueq as Compared to Neighbors of        C78 Check
```

```
PLAT244_ALERT_4_C Low    'Solvent' Ueq as Compared to Neighbors of        C77 Check
PLAT244_ALERT_4_C Low    'Solvent' Ueq as Compared to Neighbors of        C79 Check
PLAT250_ALERT_2_C Large U3/U1 Ratio for Average U(i,j) Tensor ....        2.6 Note
PLAT260_ALERT_2_C Large Average Ueq of Residue Including       Co1      0.107 Check
PLAT260_ALERT_2_C Large Average Ueq of Residue Including        O2      0.185 Check
PLAT342_ALERT_3_C Low Bond Precision on  C-C Bonds ..............     0.0183 Ang.
PLAT360_ALERT_2_C Short  C(sp3)-C(sp3) Bond  C75      - C79      .       1.42 Ang.
PLAT360_ALERT_2_C Short  C(sp3)-C(sp3) Bond  C77      - C78      .       1.38 Ang.
PLAT601_ALERT_2_C Unit Cell Contains Solvent Accessible VOIDS of .         34 Ang**3
PLAT906_ALERT_3_C Large K Value in the Analysis of Variance ......      6.266 Check
PLAT910_ALERT_3_C Missing # of FCF Reflection(s) Below Theta(Min).          8 Note
PLAT911_ALERT_3_C Missing FCF Refl Between Thmin & STh/L=    0.595          4 Report
PLAT971_ALERT_2_C Check Calcd Resid. Dens.  0.82Ang From U1              1.80 eA-3
```

Author Response: This error is due to residual density near heavy metal (U) or incomplete absorption correction. The specific structural parameter might not be precise; however, we believe the key finding (atom connection) of the structure is shown unambiguously.

```
PLAT971_ALERT_2_C Check Calcd Resid. Dens.  2.38Ang From O1              1.67 eA-3
```

Author Response: This error is due to residual density near heavy metal (U) or incomplete absorption correction. The specific structural parameter might not be precise; however, we believe the key finding (atom connection) of the structure is shown unambiguously.

```
PLAT972_ALERT_2_C Check Calcd Resid. Dens.  1.93Ang From C14            -2.07 eA-3
PLAT972_ALERT_2_C Check Calcd Resid. Dens.  0.76Ang From U1             -1.80 eA-3
PLAT972_ALERT_2_C Check Calcd Resid. Dens.  0.79Ang From U1             -1.67 eA-3
PLAT977_ALERT_2_C Check Negative Difference Density on H79A      .      -0.32 eA-3
```

Alert level G

```
PLAT002_ALERT_2_G Number of Distance or Angle Restraints on AtSite          6 Note
PLAT003_ALERT_2_G Number of Uiso or Uij Restrained non-H Atoms ...          2 Report
PLAT083_ALERT_2_G SHELXL Second Parameter in WGHT  Unusually Large      73.90 Why ?
PLAT176_ALERT_4_G The CIF-Embedded .res File Contains SADI Records          1 Report
PLAT177_ALERT_4_G The CIF-Embedded .res File Contains DELU Records          1 Report
PLAT178_ALERT_4_G The CIF-Embedded .res File Contains SIMU Records          1 Report
PLAT192_ALERT_3_G A Non-default DELU Restraint Value for First Par     0.0010 Report
PLAT192_ALERT_3_G A Non-default DELU Restraint Value for SecondPar     0.0010 Report
PLAT380_ALERT_4_G Incorrectly? Oriented X(sp2)-Methyl Moiety .....        C73 Check
PLAT398_ALERT_2_G Deviating  C-O-C   Angle From 120 for O2       .      103.6 Degree
PLAT794_ALERT_5_G Tentative Bond Valency for U1        (IV)      .       3.54 Info
PLAT794_ALERT_5_G Tentative Bond Valency for Co1       (III)     .       3.42 Info
PLAT860_ALERT_3_G Number of Least-Squares Restraints ............          16 Note
PLAT883_ALERT_1_G No Info/Value for _atom_sites_solution_primary .     Please Do !
PLAT909_ALERT_3_G Percentage of I>2sig(I) Data at Theta(Max) Still        48% Note
PLAT933_ALERT_2_G Number of HKL-OMIT Records in Embedded .res File          4 Note
PLAT941_ALERT_3_G Average HKL Measurement Multiplicity ..........         4.6 Low
PLAT978_ALERT_2_G Number C-C Bonds with Positive Residual Density.          0 Info
```

```
 1 ALERT level A = Most likely a serious problem - resolve or explain
 1 ALERT level B = A potentially serious problem, consider carefully
43 ALERT level C = Check. Ensure it is not caused by an omission or oversight
18 ALERT level G = General information/check it is not something unexpected

 2 ALERT type 1 CIF construction/syntax error, inconsistent or missing data
32 ALERT type 2 Indicator that the structure model may be wrong or deficient
11 ALERT type 3 Indicator that the structure quality may be low
16 ALERT type 4 Improvement, methodology, query or suggestion
 2 ALERT type 5 Informative message, check
```

Publication of your CIF

You should attempt to resolve as many as possible of the alerts in all categories. Often the minor alerts point to easily fixed oversights, errors and omissions in your CIF or refinement strategy, so attention to these fine details can be worthwhile. In order to resolve some of the more serious problems it may be necessary to carry out additional measurements or structure refinements. However, the nature of your study may justify the reported deviations from journal submission requirements and the more serious of these should be commented upon in the discussion or experimental section of a paper or in the "special_details" fields of the CIF. *checkCIF* was carefully designed to identify outliers and unusual parameters, but every test has its limitations and alerts that are not important in a particular case may appear. Conversely, the absence of alerts does not guarantee there are no aspects of the results needing attention. It is up to the individual to critically assess their own results and, if necessary, seek expert advice.

If you wish to submit your CIF for publication in Acta Crystallographica Section C or E, you should upload your CIF via the web. If you wish to submit your CIF for publication in IUCrData, you should upload your CIF via the web. If your CIF is to form part of a submission to another IUCr journal, you will be asked, either during electronic submission or by the Co-editor handling your paper, to upload your CIF via our web site.

PLATON version of 10/05/2023; check.def file version of 10/05/2023

Datablock Cpstar2Co_AdUO_THF - ellipsoid plot

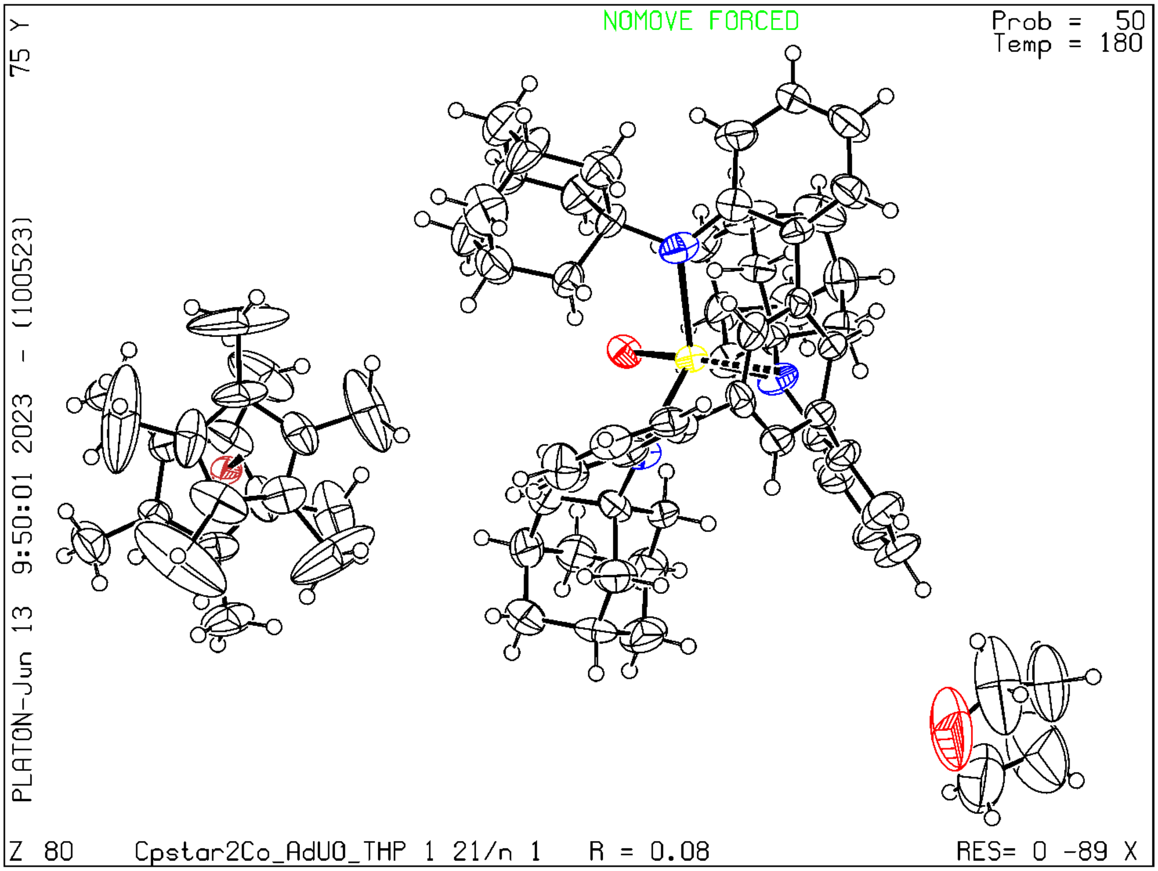

checkCIF/PLATON report

Structure factors have been supplied for datablock(s) AdUO_SbF6_2CH2Cl2

THIS REPORT IS FOR GUIDANCE ONLY. IF USED AS PART OF A REVIEW PROCEDURE FOR PUBLICATION, IT SHOULD NOT REPLACE THE EXPERTISE OF AN EXPERIENCED CRYSTALLOGRAPHIC REFEREE.

No syntax errors found. CIF dictionary Interpreting this report

Datablock: AdUO_SbF6_2CH2Cl2

Bond precision: C-C = 0.0057 A Wavelength=0.71073

Cell:	a=13.4938(4)	b=14.1867(4)	c=15.4238(4)
	alpha=66.506(3)	beta=82.626(2)	gamma=78.965(2)
Temperature:	180 K		

	Calculated	Reported
Volume	2653.34(14)	2653.34(14)
Space group	P -1	P -1
Hall group	-P 1	-P 1
Moiety formula	C54 H60 N3 O U, F6 Sb, 2(C H2 Cl2)	C54 H60 N3 O U, F6 Sb, 2(C H2 Cl2)
Sum formula	C56 H64 Cl4 F6 N3 O Sb U	C56 H64 Cl4 F6 N3 O Sb U
Mr	1410.69	1410.68
Dx,g cm-3	1.766	1.766
Z	2	2
Mu (mm-1)	3.823	3.823
F000	1388.0	1388.0
F000’	1369.31	
h,k,lmax	16,17,19	16,17,19
Nref	10828	10820
Tmin,Tmax	0.415,0.466	0.397,1.000
Tmin’	0.208	

Correction method= # Reported T Limits: Tmin=0.397 Tmax=1.000
AbsCorr = MULTI-SCAN

Data completeness= 0.999 Theta(max)= 26.372

R(reflections)= 0.0280(9872) wR2(reflections)= 0.0664(10820)

S = 1.053 Npar= 704

The following ALERTS were generated. Each ALERT has the format
test-name_ALERT_alert-type_alert-level.
Click on the hyperlinks for more details of the test.

Alert level C

```
ABSTY02_ALERT_1_C  An _exptl_absorpt_correction_type has been given without
            a literature citation. This should be contained in the
            _exptl_absorpt_process_details field.
            Absorption correction given as multi-scan
PLAT243_ALERT_4_C High   'Solvent' Ueq as Compared to Neighbors of        C55 Check
PLAT244_ALERT_4_C Low    'Solvent' Ueq as Compared to Neighbors of        Sb1 Check
PLAT250_ALERT_2_C Large U3/U1 Ratio for Average U(i,j) Tensor ....        2.4 Note
PLAT260_ALERT_2_C Large Average Ueq of Residue Including       Sb1      0.102 Check
PLAT911_ALERT_3_C Missing FCF Refl Between Thmin & STh/L=    0.600          4 Report
PLAT971_ALERT_2_C Check Calcd Resid. Dens.  0.76Ang From F4             2.04 eA-3
PLAT972_ALERT_2_C Check Calcd Resid. Dens.  0.72Ang From Sb1           -2.13 eA-3
PLAT972_ALERT_2_C Check Calcd Resid. Dens.  0.58Ang From Sb1           -1.54 eA-3
```

Alert level G

```
PLAT233_ALERT_4_G Hirshfeld (M-X Solvent)    Sb1      --F4       .       22.9 s.u.
PLAT302_ALERT_4_G Anion/Solvent/Minor-Residue Disorder (Resd  2  )        86% Note
PLAT720_ALERT_4_G Number of Unusual/Non-Standard Labels ..........          3 Note
PLAT794_ALERT_5_G Tentative Bond Valency for U1        (IV)       .       4.54 Info
PLAT883_ALERT_1_G No Info/Value for _atom_sites_solution_primary .     Please Do !
PLAT910_ALERT_3_G Missing # of FCF Reflection(s) Below Theta(Min).          4 Note
PLAT933_ALERT_2_G Number of HKL-OMIT Records in Embedded .res File          2 Note
PLAT941_ALERT_3_G Average HKL Measurement Multiplicity ..........         3.0 Low
PLAT978_ALERT_2_G Number C-C Bonds with Positive Residual Density.          1 Info
```

0 **ALERT level A** = Most likely a serious problem - resolve or explain
0 **ALERT level B** = A potentially serious problem, consider carefully
9 **ALERT level C** = Check. Ensure it is not caused by an omission or oversight
9 **ALERT level G** = General information/check it is not something unexpected

2 ALERT type 1 CIF construction/syntax error, inconsistent or missing data
7 ALERT type 2 Indicator that the structure model may be wrong or deficient
3 ALERT type 3 Indicator that the structure quality may be low
5 ALERT type 4 Improvement, methodology, query or suggestion
1 ALERT type 5 Informative message, check

Publication of your CIF

You should attempt to resolve as many as possible of the alerts in all categories. Often the minor alerts point to easily fixed oversights, errors and omissions in your CIF or refinement strategy, so attention to these fine details can be worthwhile. In order to resolve some of the more serious problems it may be necessary to carry out additional measurements or structure refinements. However, the nature of your study may justify the reported deviations from journal submission requirements and the more serious of these should be commented upon in the discussion or experimental section of a paper or in the "special_details" fields of the CIF. *checkCIF* was carefully designed to identify outliers and unusual parameters, but every test has its limitations and alerts that are not important in a particular case may appear. Conversely, the absence of alerts does not guarantee there are no aspects of the results needing attention. It is up to the individual to critically assess their own results and, if necessary, seek expert advice.

If you wish to submit your CIF for publication in Acta Crystallographica Section C or E, you should upload your CIF via the web. If you wish to submit your CIF for publication in IUCrData you should upload your CIF via the web. If your CIF is to form part of a submission to another IUCr journal, you will be asked, either during electronic submission or by the Co-editor handling your paper, to upload your CIF via our web site.

PLATON version of 12/09/2022; check.def file version of 09/08/2022

Datablock AdUO_SbF6_2CH2Cl2 - ellipsoid plot

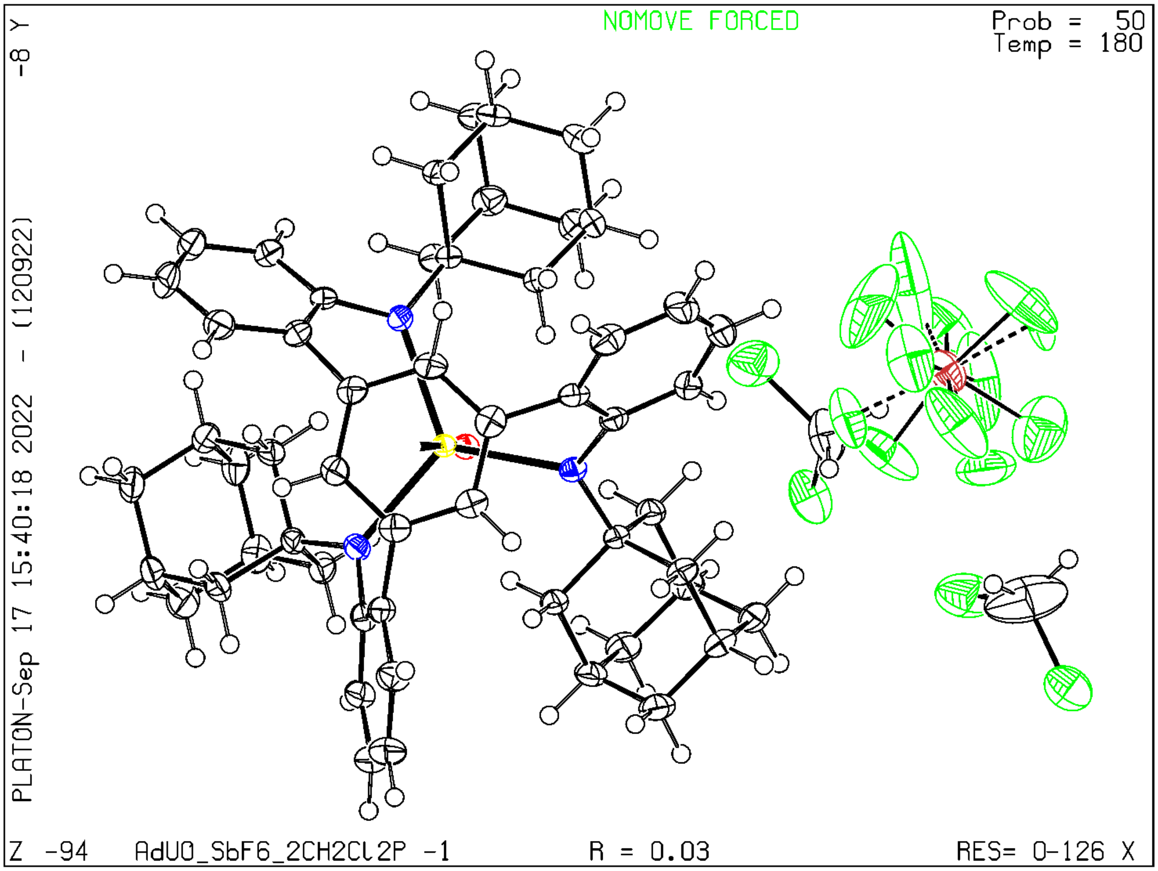

checkCIF/PLATON report

Structure factors have been supplied for datablock(s) AdUF

THIS REPORT IS FOR GUIDANCE ONLY. IF USED AS PART OF A REVIEW PROCEDURE FOR PUBLICATION, IT SHOULD NOT REPLACE THE EXPERTISE OF AN EXPERIENCED CRYSTALLOGRAPHIC REFEREE.

No syntax errors found.   CIF dictionary   Interpreting this report

Datablock: AdUF

Bond precision: C-C = 0.0049 A   Wavelength=0.71073

Cell:	a=10.9969(2)	b=18.5897(4)	c=21.2481(4)
	alpha=90	beta=101.153(2)	gamma=90
Temperature:	180 K		

	Calculated	Reported
Volume	4261.70(15)	4261.69(15)
Space group	P 21/c	P 1 21/c 1
Hall group	-P 2ybc	-P 2ybc
Moiety formula	C54 H60 F N3 U	C54 H60 F N3 U
Sum formula	C54 H60 F N3 U	C54 H60 F N3 U
Mr	1008.08	1008.08
Dx,g cm-3	1.571	1.571
Z	4	4
Mu (mm-1)	3.855	3.855
F000	2024.0	2024.0
F000’	1986.16	
h,k,lmax	15,25,29	14,25,29
Nref	11770	10710
Tmin,Tmax	0.412,0.463	
Tmin’	0.302	

Correction method= Not given

Data completeness= 0.910   Theta(max)= 29.414

R(reflections)= 0.0289(9164)   wR2(reflections)= 0.0648(10710)

S = 1.042   Npar= 532

```
The following ALERTS were generated. Each ALERT has the format
       test-name_ALERT_alert-type_alert-level.
Click on the hyperlinks for more details of the test.
```

Alert level B

```
PLAT094_ALERT_2_B Ratio of Maximum / Minimum Residual Density ....       5.94 Report
```

Author Response: High residual density near heavy metal or incomplete absorption correction

```
PLAT971_ALERT_2_B Check Calcd Resid. Dens.  2.16Ang From C46             3.48 eA-3
```

Author Response: Near heavy metal or incomplete absorption correction

Alert level C

```
PLAT057_ALERT_3_C Correction for Absorption Required   RT(exp) ...       1.12 Do !
PLAT906_ALERT_3_C Large K Value in the Analysis of Variance ......      2.997 Check
```

Alert level G

```
PLAT083_ALERT_2_G SHELXL Second Parameter in WGHT  Unusually Large       6.29 Why ?
PLAT883_ALERT_1_G No Info/Value for _atom_sites_solution_primary .     Please Do !
PLAT910_ALERT_3_G Missing # of FCF Reflection(s) Below Theta(Min).          3 Note
PLAT912_ALERT_4_G Missing # of FCF Reflections Above STh/L=  0.600       1027 Note
PLAT933_ALERT_2_G Number of HKL-OMIT Records in Embedded .res File          1 Note
PLAT941_ALERT_3_G Average HKL Measurement Multiplicity ..........         3.0 Low
PLAT978_ALERT_2_G Number C-C Bonds with Positive Residual Density.          7 Info
```

```
   0 ALERT level A = Most likely a serious problem - resolve or explain
   2 ALERT level B = A potentially serious problem, consider carefully
   2 ALERT level C = Check. Ensure it is not caused by an omission or oversight
   7 ALERT level G = General information/check it is not something unexpected

   1 ALERT type 1 CIF construction/syntax error, inconsistent or missing data
   5 ALERT type 2 Indicator that the structure model may be wrong or deficient
   4 ALERT type 3 Indicator that the structure quality may be low
   1 ALERT type 4 Improvement, methodology, query or suggestion
   0 ALERT type 5 Informative message, check
```

Publication of your CIF

You should attempt to resolve as many as possible of the alerts in all categories. Often the minor alerts point to easily fixed oversights, errors and omissions in your CIF or refinement strategy, so attention to these fine details can be worthwhile. In order to resolve some of the more serious problems it may be necessary to carry out additional measurements or structure refinements. However, the nature of your study may justify the reported deviations from journal submission requirements and the more serious of these should be commented upon in the discussion or experimental section of a paper or in the "special_details" fields of the CIF. *checkCIF* was carefully designed to identify outliers and unusual parameters, but every test has its limitations and alerts that are not important in a particular case may appear. Conversely, the absence of alerts does not guarantee there are no aspects of the results needing attention. It is up to the individual to critically assess their own results and, if necessary, seek expert advice.

If you wish to submit your CIF for publication in Acta Crystallographica Section C or E, you should upload your CIF via the web. If you wish to submit your CIF for publication in IUCrData you should upload your CIF via the web. If your CIF is to form part of a submission to another IUCr journal, you will be asked, either during electronic submission or by the Co-editor handling your paper, to upload your CIF via our web site.

PLATON version of 12/09/2022; check.def file version of 09/08/2022

Datablock AdUF - ellipsoid plot

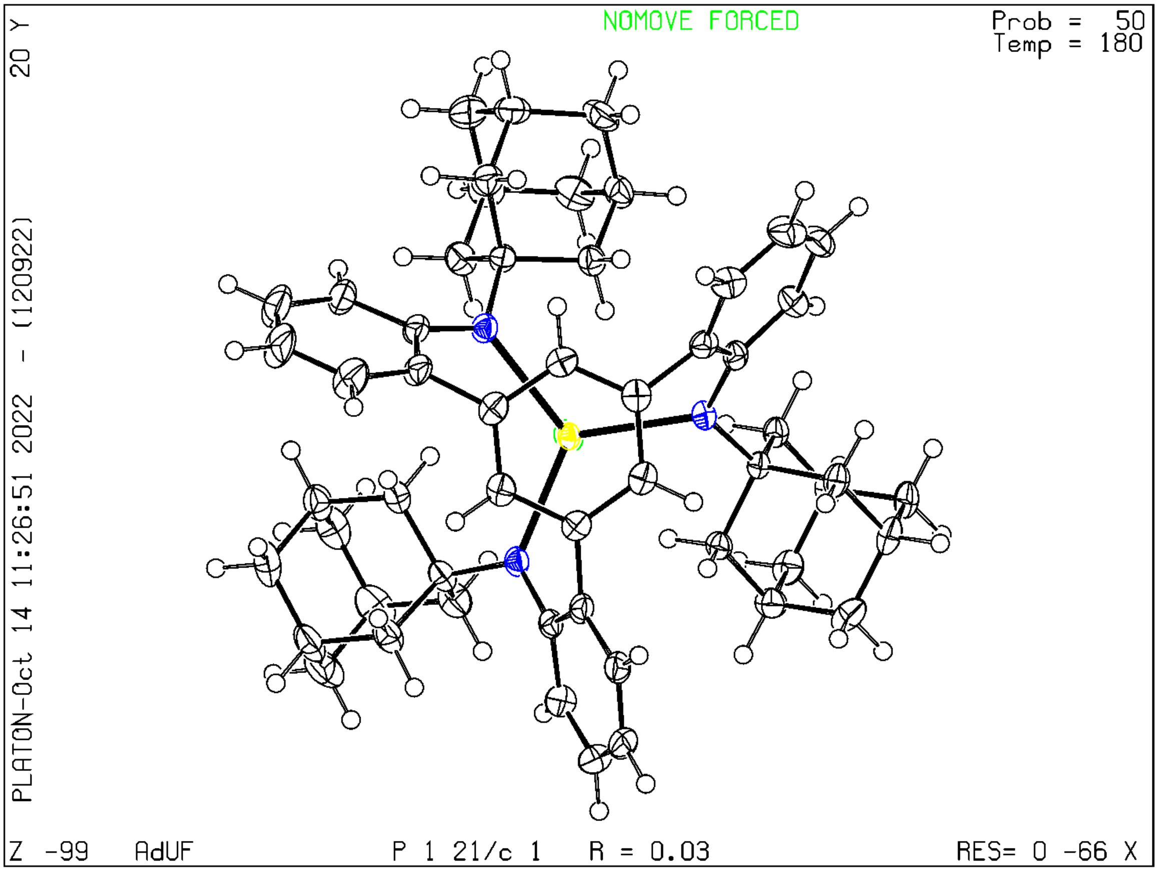

checkCIF/PLATON report

Structure factors have been supplied for datablock(s) AdUI

THIS REPORT IS FOR GUIDANCE ONLY. IF USED AS PART OF A REVIEW PROCEDURE FOR PUBLICATION, IT SHOULD NOT REPLACE THE EXPERTISE OF AN EXPERIENCED CRYSTALLOGRAPHIC REFEREE.

No syntax errors found. CIF dictionary Interpreting this report

Datablock: AdUI

```
Bond precision:   C-C = 0.0059 A          Wavelength=0.71073

Cell:             a=20.7007(5)     b=10.8830(3)       c=19.4004(5)
                  alpha=90         beta=92.280(2)     gamma=90
Temperature:      180 K

                Calculated                    Reported
Volume          4367.2(2)                     4367.2(2)
Space group     P 21/c                        P 1 21/c 1
Hall group      -P 2ybc                       -P 2ybc
Moiety formula  C54 H60 I N3 U                C54 H60 I N3 U
Sum formula     C54 H60 I N3 U                C54 H60 I N3 U
Mr              1115.98                       1115.98
Dx,g cm-3       1.697                         1.697
Z               4                             4
Mu (mm-1)       4.465                         4.465
F000            2200.0                        2200.0
F000'           2160.31
h,k,lmax        28,15,26                      28,14,26
Nref            12000                         10932
Tmin,Tmax       0.272,0.409                   0.613,1.000
Tmin'           0.201

Correction method= # Reported T Limits: Tmin=0.613 Tmax=1.000
AbsCorr = MULTI-SCAN

Data completeness= 0.911           Theta(max)= 29.353

R(reflections)= 0.0353( 8980)                    wR2(reflections)=
                                                 0.0665( 10932)
S = 1.026                    Npar= 532
```

The following ALERTS were generated. Each ALERT has the format
test-name_ALERT_alert-type_alert-level.
Click on the hyperlinks for more details of the test.

Alert level C

```
ABSTY02_ALERT_1_C  An _exptl_absorpt_correction_type has been given without
            a literature citation. This should be contained in the
            _exptl_absorpt_process_details field.
            Absorption correction given as multi-scan
PLAT906_ALERT_3_C Large K Value in the Analysis of Variance ......      2.854 Check
PLAT911_ALERT_3_C Missing FCF Refl Between Thmin & STh/L=    0.600         34 Report
PLAT971_ALERT_2_C Check Calcd Resid. Dens.  1.00Ang From U1             1.70 eA-3
PLAT971_ALERT_2_C Check Calcd Resid. Dens.  0.79Ang From U1             1.65 eA-3
PLAT972_ALERT_2_C Check Calcd Resid. Dens.  0.65Ang From U1            -1.67 eA-3
PLAT975_ALERT_2_C Check Calcd Resid. Dens.  0.81Ang From N3      .      0.65 eA-3
PLAT975_ALERT_2_C Check Calcd Resid. Dens.  1.04Ang From N2      .      0.59 eA-3
```

Alert level G

```
PLAT083_ALERT_2_G SHELXL Second Parameter in WGHT  Unusually Large      12.02 Why ?
PLAT232_ALERT_2_G Hirshfeld Test Diff (M-X)  U1       --I1       .        9.2 s.u.
PLAT883_ALERT_1_G No Info/Value for _atom_sites_solution_primary .     Please Do !
PLAT910_ALERT_3_G Missing # of FCF Reflection(s) Below Theta(Min).          1 Note
PLAT912_ALERT_4_G Missing # of FCF Reflections Above STh/L=  0.600       1031 Note
PLAT933_ALERT_2_G Number of HKL-OMIT Records in Embedded .res File          5 Note
PLAT941_ALERT_3_G Average HKL Measurement Multiplicity ..........         3.1 Low
PLAT978_ALERT_2_G Number C-C Bonds with Positive Residual Density.          1 Info
```

0 **ALERT level A** = Most likely a serious problem - resolve or explain
0 **ALERT level B** = A potentially serious problem, consider carefully
8 **ALERT level C** = Check. Ensure it is not caused by an omission or oversight
8 **ALERT level G** = General information/check it is not something unexpected

2 ALERT type 1 CIF construction/syntax error, inconsistent or missing data
9 ALERT type 2 Indicator that the structure model may be wrong or deficient
4 ALERT type 3 Indicator that the structure quality may be low
1 ALERT type 4 Improvement, methodology, query or suggestion
0 ALERT type 5 Informative message, check

Publication of your CIF

You should attempt to resolve as many as possible of the alerts in all categories. Often the minor alerts point to easily fixed oversights, errors and omissions in your CIF or refinement strategy, so attention to these fine details can be worthwhile. In order to resolve some of the more serious problems it may be necessary to carry out additional measurements or structure refinements. However, the nature of your study may justify the reported deviations from journal submission requirements and the more serious of these should be commented upon in the discussion or experimental section of a paper or in the "special_details" fields of the CIF. *checkCIF* was carefully designed to identify outliers and unusual parameters, but every test has its limitations and alerts that are not important in a particular case may appear. Conversely, the absence of alerts does not guarantee there are no aspects of the results needing attention. It is up to the individual to critically assess their own results and, if necessary, seek expert advice.

If you wish to submit your CIF for publication in Acta Crystallographica Section C or E, you should upload your CIF via the web. If you wish to submit your CIF for publication in IUCrData you should upload your CIF via the web. If your CIF is to form part of a submission to another IUCr journal, you will be asked, either during electronic submission or by the Co-editor handling your paper, to upload your CIF via our web site.

PLATON version of 12/09/2022; check.def file version of 09/08/2022

Datablock AdUI - ellipsoid plot

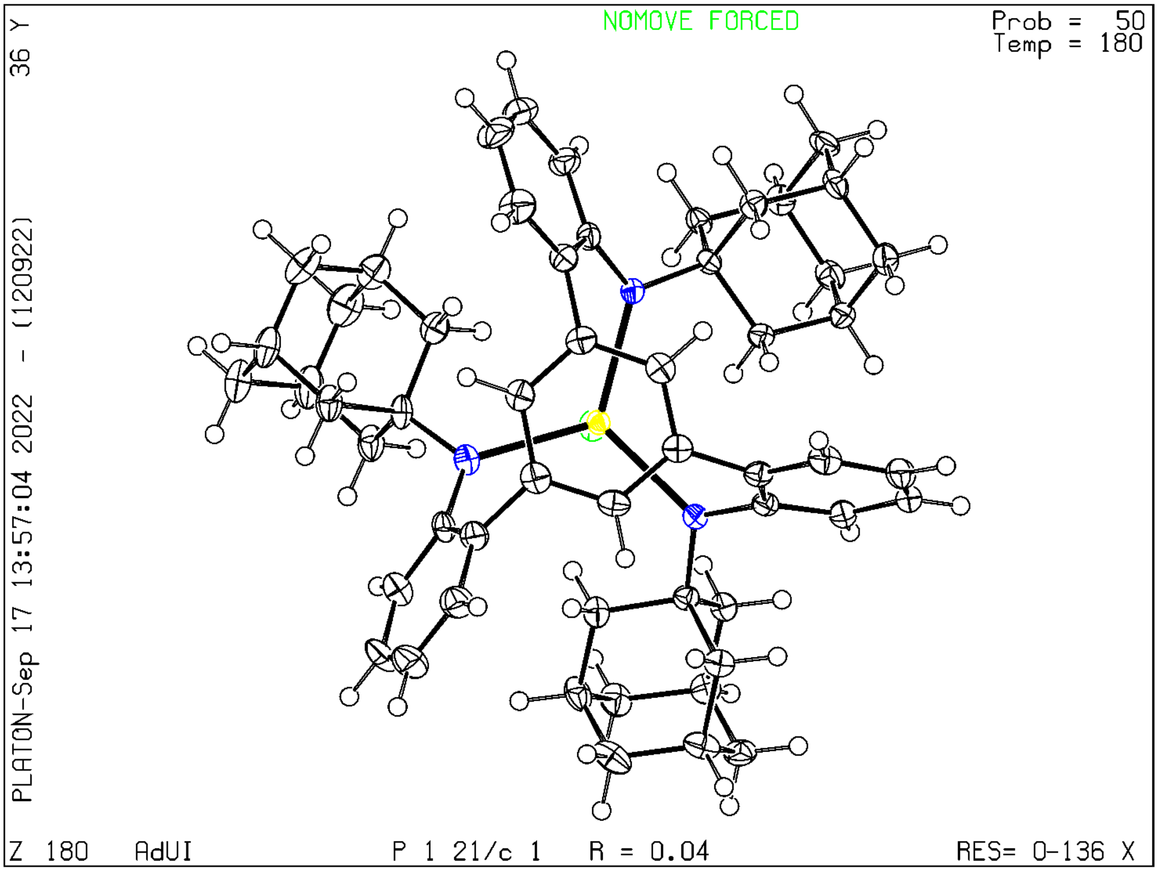

checkCIF/PLATON report

Structure factors have been supplied for datablock(s) XPhos

THIS REPORT IS FOR GUIDANCE ONLY. IF USED AS PART OF A REVIEW PROCEDURE FOR PUBLICATION, IT SHOULD NOT REPLACE THE EXPERTISE OF AN EXPERIENCED CRYSTALLOGRAPHIC REFEREE.

No syntax errors found. CIF dictionary Interpreting this report

Datablock: XPhos

Bond precision: C-C = 0.0017 A Wavelength=0.71073

Cell:	a=10.9278(3)	b=18.6308(6)	c=14.7813(5)
	alpha=90	beta=104.640(3)	gamma=90
Temperature:	180 K		

	Calculated	Reported
Volume	2911.67(16)	2911.67(16)
Space group	P 21/c	P 1 21/c 1
Hall group	-P 2ybc	-P 2ybc
Moiety formula	C33 H49 P	C33 H49 P
Sum formula	C33 H49 P	C33 H49 P
Mr	476.69	476.69
Dx,g cm-3	1.087	1.087
Z	4	4
Mu (mm-1)	0.113	0.113
F000	1048.0	1048.0
F000'	1048.73	
h,k,lmax	15,26,21	14,26,20
Nref	8797	7725
Tmin,Tmax	0.978,0.987	0.835,1.000
Tmin'	0.978	

Correction method= # Reported T Limits: Tmin=0.835 Tmax=1.000
AbsCorr = MULTI-SCAN

Data completeness= 0.878 Theta(max)= 30.396

R(reflections)= 0.0401(6573) wR2(reflections)= 0.1061(7725)

S = 1.031 Npar= 313

```
The following ALERTS were generated. Each ALERT has the format
       test-name_ALERT_alert-type_alert-level.
Click on the hyperlinks for more details of the test.
```

Alert level C

```
PLAT906_ALERT_3_C Large K Value in the Analysis of Variance ......      2.525 Check
PLAT910_ALERT_3_C Missing # of FCF Reflection(s) Below Theta(Min).          6 Note
```

Alert level G

```
PLAT883_ALERT_1_G No Info/Value for _atom_sites_solution_primary .     Please Do !
PLAT912_ALERT_4_G Missing # of FCF Reflections Above STh/L=  0.600       1005 Note
PLAT933_ALERT_2_G Number of HKL-OMIT Records in Embedded .res File          1 Note
PLAT941_ALERT_3_G Average HKL Measurement Multiplicity ...........        4.5 Low
PLAT978_ALERT_2_G Number C-C Bonds with Positive Residual Density.         21 Info
```

```
   0 ALERT level A = Most likely a serious problem - resolve or explain
   0 ALERT level B = A potentially serious problem, consider carefully
   2 ALERT level C = Check. Ensure it is not caused by an omission or oversight
   5 ALERT level G = General information/check it is not something unexpected

   1 ALERT type 1 CIF construction/syntax error, inconsistent or missing data
   2 ALERT type 2 Indicator that the structure model may be wrong or deficient
   3 ALERT type 3 Indicator that the structure quality may be low
   1 ALERT type 4 Improvement, methodology, query or suggestion
   0 ALERT type 5 Informative message, check
```

Publication of your CIF

You should attempt to resolve as many as possible of the alerts in all categories. Often the minor alerts point to easily fixed oversights, errors and omissions in your CIF or refinement strategy, so attention to these fine details can be worthwhile. In order to resolve some of the more serious problems it may be necessary to carry out additional measurements or structure refinements. However, the nature of your study may justify the reported deviations from journal submission requirements and the more serious of these should be commented upon in the discussion or experimental section of a paper or in the "special_details" fields of the CIF. *checkCIF* was carefully designed to identify outliers and unusual parameters, but every test has its limitations and alerts that are not important in a particular case may appear. Conversely, the absence of alerts does not guarantee there are no aspects of the results needing attention. It is up to the individual to critically assess their own results and, if necessary, seek expert advice.

If you wish to submit your CIF for publication in Acta Crystallographica Section C or E, you should upload your CIF via the web. If you wish to submit your CIF for publication in IUCrData you should upload your CIF via the web. If your CIF is to form part of a submission to another IUCr journal, you will be asked, either during electronic submission or by the Co-editor handling your paper, to upload your CIF via our web site.

PLATON version of 12/09/2022; check.def file version of 09/08/2022

Datablock XPhos - ellipsoid plot

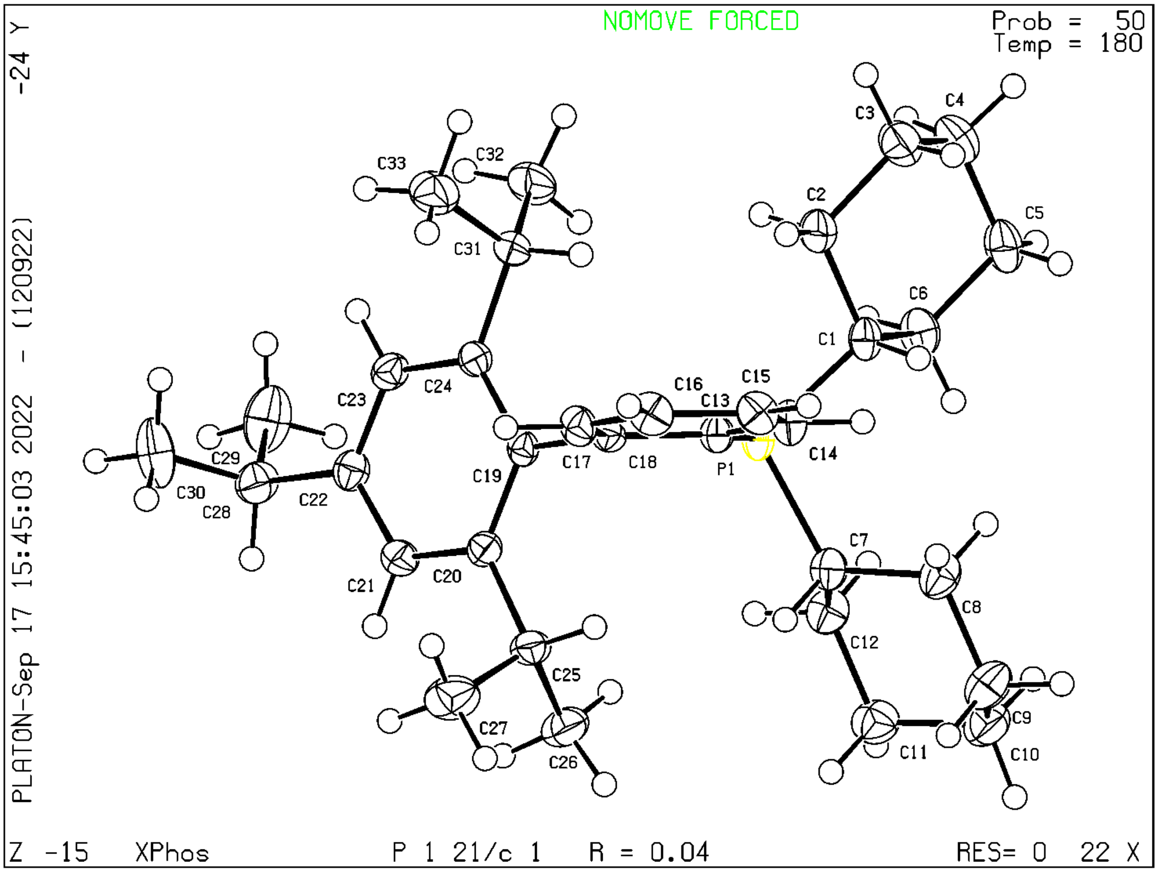

Supplement: Supplementary file 3 — Source Data [file 41467_2023_40403_MOESM3_ESM.zip › checkcifs.pdf]
